# Discovery of Novel 3-Hydroxyquinazoline-2,4(1*H*,3*H*)-Dione Derivatives: A Series of Metal Ion Chelators with Potent Anti-HCV Activities

**DOI:** 10.3390/ijms23115930

**Published:** 2022-05-25

**Authors:** Yang Cao, Abudumijiti Aimaiti, Zeyun Zhu, Lu Zhou, Deyong Ye

**Affiliations:** 1 Department of Medicinal Chemistry, School of Pharmacy, Fudan University, 826 Zhangheng Rd, Shanghai 201203, China; ycao14@fudan.edu.cn (Y.C.); traveller-zzy@outlook.com (Z.Z.); 2 Shanghai Medical College, Fudan University, 130 Dongan Rd, Shanghai 200032, China; abu@fudan.edu.cn

**Keywords:** chelator, NS5B, anti-HCV, hydroxyquinazolinedione, synthesis

## Abstract

Millions of people worldwide suffer from acute or chronic liver inflammation caused by the hepatitis C virus (HCV). Metal ion chelators have achieved widespread success in the development of antiviral drugs. Some inhibitors with metal ion chelating structures have been proven to have good inhibitory activities on non-structural protein 5B (NS5B) polymerase. However, most of the reported metal ion chelators showed poor anti-HCV potency at the cellular level. Hence, we designed and synthesized a series of 3-hydroxyquinazoline-2,4(1*H*,3*H*)-dione derivatives with novel metal ion chelating structures. Typical compounds such as **21h**, **21k,** and **21t** showed better anti-HCV activities than ribavirin with EC_50_ values less than 10 μM. **21t** is currently known as one of the metal ion chelators with the best anti-HCV potency (EC_50_ = 2.0 μM) at the cellular level and has a better therapeutic index (TI > 25) as compared to ribavirin and the reported compound **6**. In the thermal shift assay, the representative compounds **21e** and **21k** increased the melting temperature (*T*_m_) of NS5B protein solution by 1.6 °C and 2.1 °C, respectively, at the test concentration, indicating that these compounds may exert an anti-HCV effect by targeting NS5B. This speculation was also supported by our molecular docking studies and ultraviolet-visible (UV-Vis) spectrophotometry assay, in which the possibility of binding of 3-hydroxyquinazoline-2,4(1*H*,3*H*)-diones with Mg^2+^ in the NS5B catalytic center was observed.

## 1. Introduction

Hepatitis C is an acute or chronic liver inflammation caused by the hepatitis C virus (HCV), which can lead to cirrhosis or liver cancer [1]. According to statistics from the World Health Organization (WHO), about 58 million people worldwide are infected with the hepatitis C virus, with about 1.5 million new infections occurring every year [2]. In 2019, approximately 290,000 people died of hepatitis C, mainly due to primary liver cancer and cirrhosis [2]. HCV is a small positive sense, single-stranded RNA virus of the Flaviviridae family. Due to the high error rate when synthesizing RNA, HCV has a variety of subtypes. Based on the genetic differences between HCV isolates, 8 genotypes (1–8) and 93 subtypes of hepatitis C virus have been confirmed [3], in which subtypes 1a and 1b were found to be predominant [4,5]. Unlike hepatitis A and hepatitis B viruses, there is currently no effective vaccine against HCV [6], which makes the overall prevention and control of HCV very difficult.

At present, direct-acting antivirals (DAAs) mainly target non-structural proteins NS3/4A, NS5A, and NS5B in the treatment of HCV infection. Non-structural protein 5B (NS5B) is a key enzyme in the synthesis of HCV RNA strands. As an RNA-dependent RNA polymerase (RdRp), NS5B takes the original RNA chain as a template and catalyzes the polymerization of ribonucleoside triphosphates (rNTP) to synthesize the new RNA chains [7,8].

The NS5B protein consists of three domains: fingers, thumb, and palm regions [8]. The thumb and palm regions contain four allosteric sites which can regulate the conformation of the NS5B protein, thereby affecting the RNA synthesis [9,10]. Inhibitors of various structural types, such as benzothiadiazines [11] and benzofurans [12], have been identified to be capable of binding to the allosteric sites and show potent HCV inhibitory activities [10,11,12,13,14,15]. Due to the mutability and high mutation rates of the allosteric sites, NS5B allosteric inhibitors are mostly effective against only a small range of virus subtypes and are prone to drug resistance [16,17].

The active center of NS5B in the palm region is responsible for catalyzing the nucleophilic attack of the 3′-terminal hydroxyl group of the RNA extension chain to the rNTP substrates [18]. Since the NS5B polymerase active site is highly conserved, inhibitors targeting the NS5B active site have a higher genetic barrier to drug resistance and more pan-genotypic activities as compared to other HCV DAAs [17,19,20]. Nucleoside or nucleotide inhibitors target the active center of NS5B in their active form, which could be accepted as substrates for NS5B polymerase and ultimately incorporated into growing RNA strands, terminating the HCV replication cycle [8]. A variety of nucleoside or nucleotide inhibitors have been shown to have good anti-HCV activities [21,22]. However, the development of many nucleoside or nucleotide analogs was halted in clinical trials due to the widespread mitochondrial toxicity [23]. In addition, nucleoside inhibitors represented by ribavirin can also exert toxicity through the disruption of natural nucleoside triphosphate (NTP) pools [24]. These features make the development of nucleoside or nucleotide-based anti-HCV drugs more risky. Sofosbuvir (trade name Sovaldi) is currently the only nucleotide prodrug approved by the U.S. Food and Drug Administration (FDA) for the treatment of HCV infection [25]. Although the combination regimens of sofosbuvir with other DAAs have achieved high HCV clearance rates, 5–10% of patients still do not respond well to the current therapies [26]. More importantly, a growing list of HCV mutations associated with DAAs resistance has been found in clinical practice, including sofosbuvir [27,28,29,30,31]. Therefore, the development of novel HCV inhibitors is still of great importance.

**Figure 1:**
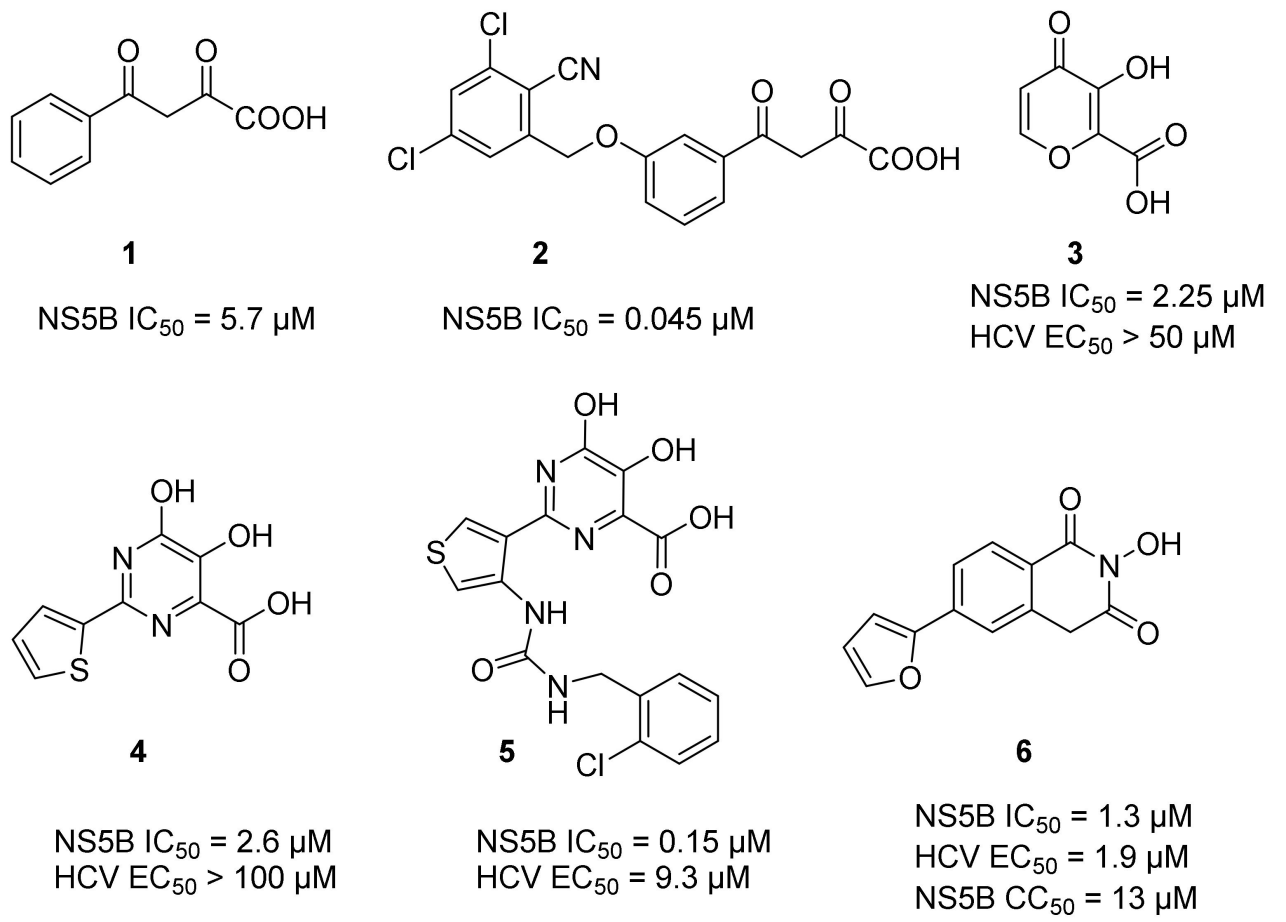
A series of representative non-structural protein 5B (NS5B) metal ion chelators.

Two Mg^2+^ ions in the active center of NS5B form a chelating complex with the conserved amino acid residues D220, D318, and D319, stabilizing the central structure of the active site [8]. In addition to nucleoside or nucleotide inhibitors, some metal ion chelators can also target the conserved active center of NS5B by chelating with the Mg^2+,^ which are essential for the polymerase active center [32]. Metal ion chelators can be treated as pyrophosphate (PPi) mimetics that block viral RNA replication by competing with the phosphate group of NTP for binding to the catalytic center of polymerases [33]. As shown in Figure 1, a variety of structural types of metal ion chelators, such as α,γ-diketo acids (compound **1** and **2**) [34], meconic acids (compound **3**) [35], 5,6-dihydroxypyrimidine-4-carboxylic acids (compound **4** and **5**) [36,37], and 2-hydroxyisoquinoline-1,3-diones (compound **6**) [38] have been identified to have potent inhibitory activities against NS5B. Molecular simulations also suggested that these compounds might bind to the two Mg^2+^ ions in the active center of NS5B through a “tridentate” chelation mode [37,38]. However, the anti-HCV activities of most reported metal ion chelators at the cellular level did not reach expectations, probably due to the low membrane permeability of the compounds caused by the carboxyl-containing metal-chelating functional groups. Interestingly, the representative compound **6** of the 2-hydroxyisoquinoline-1,3-diones with no carboxyl group possesses a good cellular-level anti-HCV activity (EC_50_ = 1.9 μM). Nevertheless, the therapeutic index of such compounds still needs optimization [38].

Magnesium ions play a central role as metal cofactors in a variety of enzymes, especially those involved in nucleic acid biochemistry [39,40]. In addition to HCV NS5B polymerase, the representative ones are HIV-1 integrase, HIV-1 ribonuclease H (RNase H), and influenza virus endonuclease. Targeting these metal cofactor-containing viral proteins to design and develop a series of metal ion chelators has been shown to be a practical and effective antiviral strategy [41,42,43,44,45,46,47]. To date, five HIV-1 integrase inhibitors (raltegravir, elvitegravir, dolutegravir, bictegravir, and cabotegravir) containing metal ion chelating structures have been approved by the FDA [48]. These encouraging results confirm that the metal ion chelation strategy has a broad prospect in the development of HCV DAAs.

Herein, we designed and synthesized a series of 3-hydroxyquinazoline-2,4(1*H*,3*H*)-dione derivatives acting as metal ion chelators. The cellular-level anti-HCV activities of the compounds were evaluated based on the HCV replicon model. In addition, the binding of representative compounds to the NS5B protein was determined using the thermal shift assay (TSA) to validate the targeted protein of the compounds. The in vitro binding properties of the preferred compound with Mg^2+^ were studied by ultraviolet-visible (UV-Vis) spectrophotometry. Molecular modeling was conducted to explore the binding mode of this series of metal ion chelators to the active center of NS5B.

## 2. Results and Discussion

### 2.1. Chemistry

**Scheme 1:**
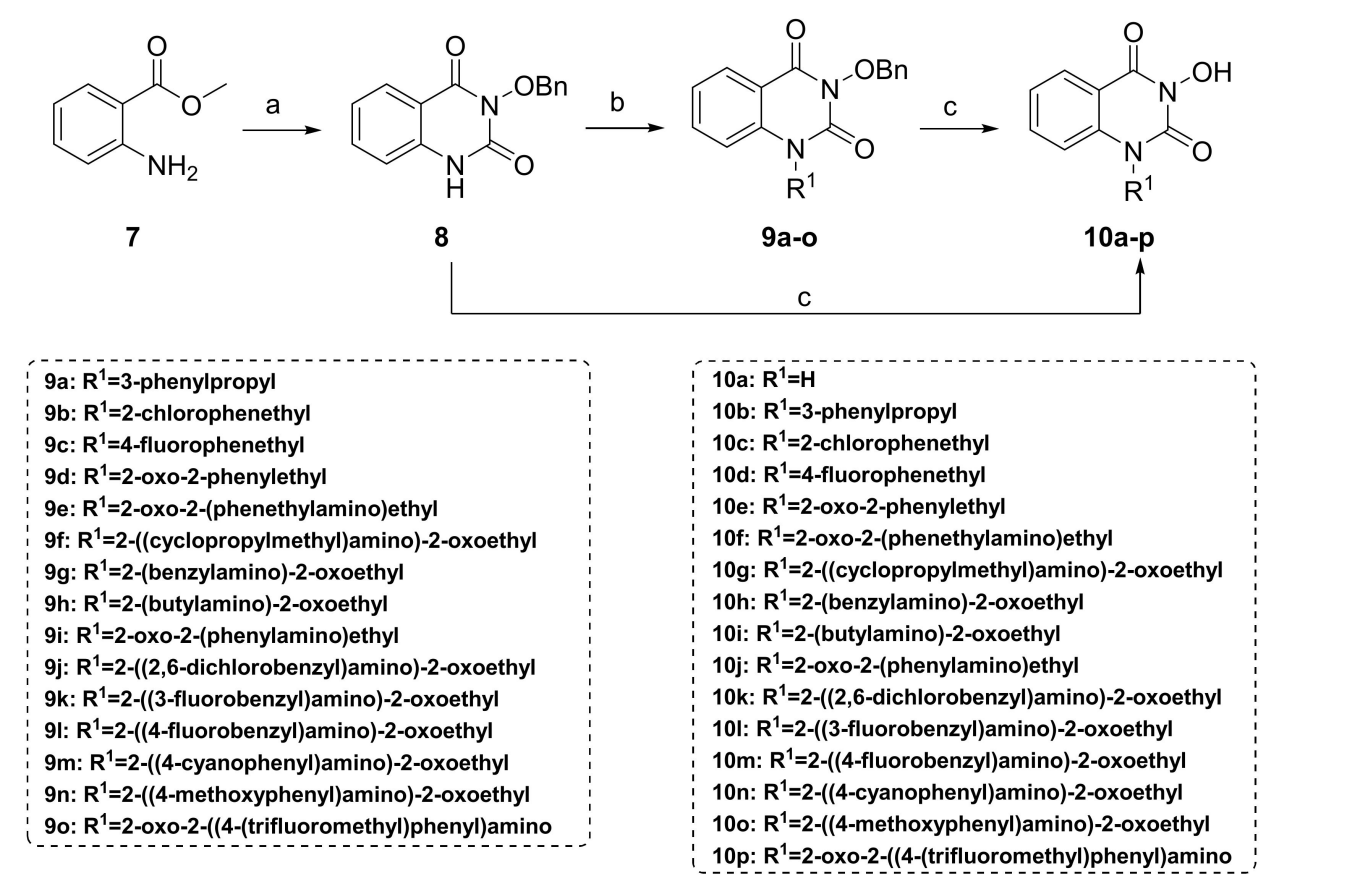
Synthesis of N-1 substituted 3-hydroxy-quinazoline-2,4(1*H*,3*H*)-diones. Reagents and conditions: (**a**) (i) CDI/toluene, reflux, 2 h, then NH_2_OBn, reflux, 4 h (ii) NaOH/H_2_O/EtOH, reflux, 2 h; (**b**) R^1^X, K_2_CO_3_, DMF, 80 °C, 2 h; (**c**) 48% HBr, AcOH, reflux, 2 h; or H_2_, 10% Pd/C, THF/MeOH, room temperature (r.t.), 4–12 h.

**Scheme 2:**
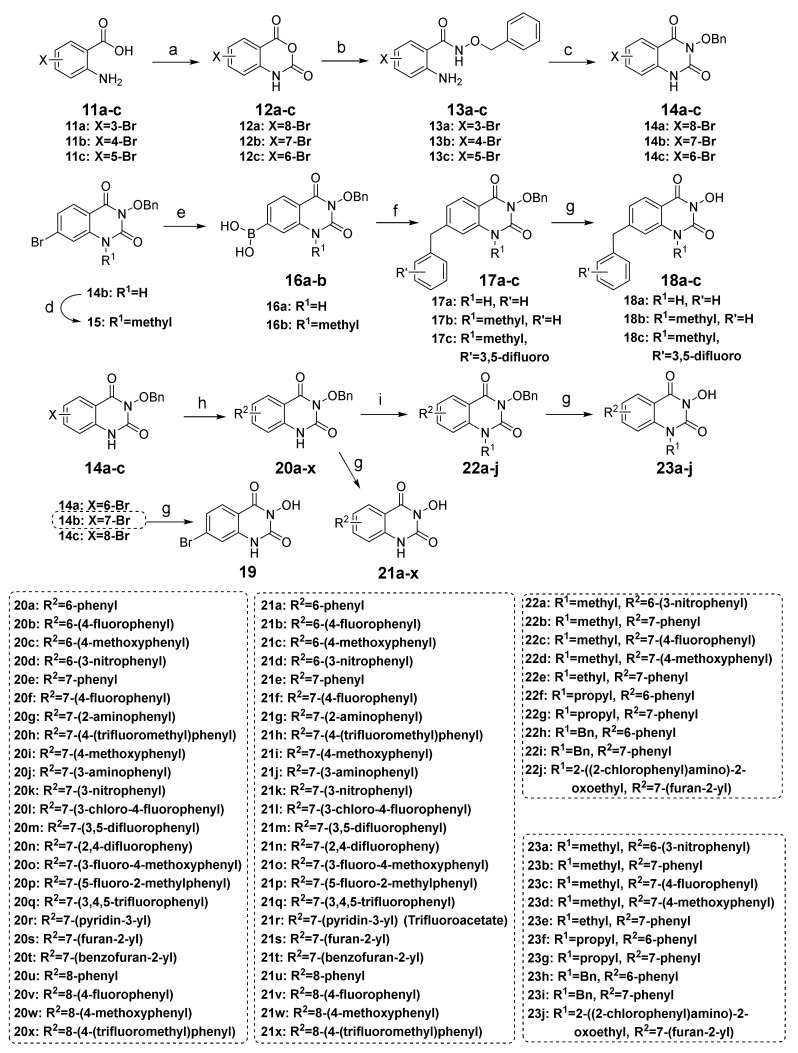
Synthesis of C-6/C-7/C-8 substituted 3-hydroxyquinazoline-2,4(1*H*,3*H*)-diones and the key intermediates. Reagents and conditions: (**a**) triphosgene, 1,4-dioxane, reflux, 6 h; (**b**) NH_2_OBn, triethylamine, EtOH, reflux, 3 h; (**c**) triphosgene, triethylamine, THF, r.t., 2 h; (**d**) CH_3_I, K_2_CO_3_, DMF, r.t., 2 h; (**e**) bis(pinacolato)diboron, Pd(dppf)Cl_2_, KOAc, 1,4-dioxane, 100 °C, 8 h; (**f**) BnBr or substituted BnBr, Pd(dppf)Cl_2_, KOAc, dioxane/H_2_O, 100 °C, 3 h; (**g**) 48% HBr, AcOH, reflux, 2 h; or H_2_, 10% Pd/C, THF/MeOH, 4–12 h; or TFA, reflux, 12–16 h; or TiCl_4_/potassium sodium tartrate, DCM, 1.5 h; (**h**) R^2^B(OH)_2_, K_2_CO_3_, Pd(PPh_3_)_4_, dioxane/H_2_O, 100 °C, 12 h; (**i**) R^1^Br, K_2_CO_3_, DMF, 80 °C, 2 h; or R^1^I, K_2_CO_3_, DMF, r.t., 2 h.

In this study, we synthesized a series of novel metal ion chelators with 3-hydroxyquinazoline-2,4(1*H*,3*H*)-dione parent nucleus. Based on the strategy of partition structure modification, we first tried different substituents on the 1-nitrogen of hydroxyquinazolinedione core to explore the preliminary structure-activity relationship (SAR) (Scheme 1). Further, structural modifications at the phenyl ring region of the parent nucleus were implemented (Scheme 2). As shown in Scheme 1, the starting material, methyl anthranilate **7**, underwent a one-pot two-step reaction to give the key intermediate 3-(benzyloxy)quinazoline-2,4(1*H*,3*H*)-dione (**8**). Initially, methyl anthranilate **7** was condensed with 1,1′-carbonyldiimidazole (CDI) and benzyloxyamine successively to introduce the carbonyl fragment. Then the intermediate **8** was obtained after the intramolecular cyclization reaction under a strongly alkaline environment. In step b, the intermediate **8** and the corresponding halide underwent an alkylation reaction with various substituents introduced on the N-1 position to yield compounds **9a**–**o**. Finally, to obtain the target compounds N-1 substituted 3-hydroxyquinazoline-2,4(1*H*,3*H*)-diones (**10a**–**p**), the benzyl groups in **8** and **9a**–**o** were removed using different conditions of deprotection, i.e., heating in hydrobromic acid/acetic acid mixture under reflux, or palladium-carbon catalyzed hydrogenation.

The synthetic route depicted in Scheme 2 produced the C-6/C-7/C-8 substituted 3-hydroxyquinazoline-2,4(1*H*,3*H*)-diones. Bromoanthranilic acid (**11a**–**c**) and triphosgene were refluxed in dioxane to afford the oxazinedione intermediates **12a**–**c,** which were treated with benzyloxyamine to produce **13a**–**c** through the nucleophilic reaction. Then, the aniline groups of **13a**–**c** were amidated by reacting with triphosgene, and subsequently, the products underwent an intramolecular cyclization reaction to yield the key intermediates **14a**–**c** possessing the quinazolinedione parent nucleus. Next, we tried to introduce benzyl substitution on the phenyl ring of the parent nucleus. The C-7 brominated compound **14b** and its N-1 methylated product **15** were treated with bis(pinacolato)diboron to achieve the boron intermediates **16a** and **16b**, respectively, which were then subjected to the Pd(dppf)Cl_2_ catalyzed Suzuki coupling reaction with benzyl bromide or substituted benzyl bromide to obtain compounds **17a**–**c**. Finally, under the catalysis of palladium-carbon, the benzyl groups of **17a**–**c** were removed by hydrogenation reaction to yield three target compounds **18a**–**c** substituted by various benzyl groups at the C-7 position.

In order to fully study the SAR on the phenyl ring of the parent nucleus, we also prepared a series of C-6/C-7/C-8 substituted 3-hydroxyquinazoline-2,4(1*H*,3*H*)-diones according to the synthetic route in Scheme 2. The key intermediates **14a**–**c** were subjected to Suzuki coupling reaction with different arylboronic acids under the catalysis of Pd(PPh_3_)_4_ to obtain intermediates **20a**–**x**, which were then debenzylated by different conditions to yield the target compounds **21a**–**x**. The C-7 brominated compound **14b** was directly debenzylated in the mixture of hydrobromic acid/acetic acid to give the target compound **19**. On the other hand, alkylation of the N-1 position of **20a**–**x** by different halides under the condition of inorganic base afforded intermediates **22a**–**j**, which were then debenzylated to produce the target compounds **23a**–**j** using the conditions as described in converting **20a**–**x** to **21a**–**x**. In the selection of debenzylation conditions, the more environmentally friendly palladium-carbon catalyzed hydrogenation is preferred. Strong acids such as trifluoroacetic acid, hydrobromic acid, and acetic acid are used for debenzylation unless the hydrogenation conditions are not applicable. As for compound **20s**, the debenzylation reaction was performed using titanium tetrachloride (TiCl_4_) in dichloromethane (DCM) to obtain the target compound **21s** in high purity. The structures of various target compounds were confirmed by ESI-MS, ^1^H NMR, and ^13^C NMR spectral data, which are shown in the Materials and Methods section.

### 2.2. Anti-HCV Assay

The HCV replicons were validated as convenient and effective models for testing the anti-HCV activity [49]. Since HCV subtype 1b is one of the major subtypes worldwide, especially in China [4,5], the replicon model from the Huh-7.5.1 cell line integrating HCV 1b genome encoding the nonstructural proteins was chosen to evaluate the anti-HCV activities of 3-hydroxyquinazoline-2,4(1*H*,3*H*)-diones. The inhibitory rates of the compounds on HCV replicon cells were determined at concentrations of 25 μM and 10 μM, respectively. The broad-spectrum antiviral drug ribavirin (RBV) and the NS5B nucleoside inhibitor 2′-*C*-methyladenosine (2CMA) were selected as positive controls. The cytotoxicity of these compounds on replicon cells was determined using the cell counting kit-8 (CCK-8) assay.

As shown in Table 1, the EC_50_ values of ribavirin and 2CMA measured under the experimental conditions were 20.0 μM and 0.36 μM, respectively, which were close to the reported 14 μM [38] and 0.3 μM [50], indicating the reliability of the test method in this study. As compared to 3-hydroxyquinazoline-2,4(1*H*,3*H*)-dione (**10a**), the introduction of phenylpropyl group (**10b**) and substituted phenethyl groups (**10c**, **10d**) at the N-1 position can increase the HCV inhibitory rate at 25 μM and 10 μM. The EC_50_ value of **10d** reaches 13.3 μM, while compounds **10c** and **10d** both show some cytotoxicity. The ketone carbonyl group or different types of amide fragments were further introduced into the N-1 position nitrogen of the parent nucleus. The results indicated that when there is no aromatic group in the N-1 substituents (**10g**, **10i**), the compounds inhibited HCV by less than 30% at the tested concentrations. Compared with **10j**, the compounds with longer N-1 substituted chain (**10f**, **10h**) bearing aryl groups had lower HCV inhibitory activities. The inhibitory rate of **10f** and **10h** did not exceed 50% at 25 μM; however, **10j** had the corresponding value of 62.7%. When halogen (**10k**, **10l**, **10m**), methoxy (**10o**), cyano (**10n**), and trifluoromethyl (**10p**) groups were introduced into the phenyl ring of the substituent at the N-1 position, the activity was not significantly improved. In general, **10n** with cyano-substituted on the phenyl ring of the amide fragment had the best anti-HCV activity, with an EC_50_ value of 6.4 μM. Similar to ribavirin, these compounds have certain cytotoxicity, with therapeutic indexes (TI) of about 1.7–1.9, which is comparable to the tested TI of ribavirin (TI = 2.3). Among the synthesized N-1 substituted 3-hydroxyquinazoline-2,4(1*H*,3*H*)-diones, compounds **10n** and **10p** showed anti-HCV EC_50_ values less than 10 μM, which were more potent than ribavirin (EC_50_ = 20.0 μM). These results preliminarily validated the anti-HCV potential of 3-hydroxyquinazoline-2,4(1*H*,3*H*)-dione derivatives.

Further optimization of the anti-HCV activity and therapeutic index was conducted by exploring the SAR on the phenyl ring of the quinazolinedione parent nucleus. As listed in Table 2, C-6/C-7/C-8 aryl or benzyl groups substituted 3-hydroxyquinazoline-2,4(1*H*,3*H*)-diones were subjected to an anti-HCV assay based on the HCV 1b replicon model. The C-7 benzyl substituted compound **18a** (inhibitory rate = 59.8% at 25 μM) had a higher inhibitory rate of HCV than that of the unsubstituted **10a** (inhibitory rate = 36.0% at 25 μM). However, the introduction of a methyl group (**18b**) at the N-1 position of **18a** failed to effectively improve the anti-HCV potency. Compared with the C-7 brominated compound **19**, the inhibitory rates of **18a**–**c** did not exceed 40% at 10 μM, indicating that the benzyl substituents at the phenyl ring of the hydroxyquinazolinedione core may not be dominant in enhancing the inhibitory activity of HCV.

**Figure 2:**
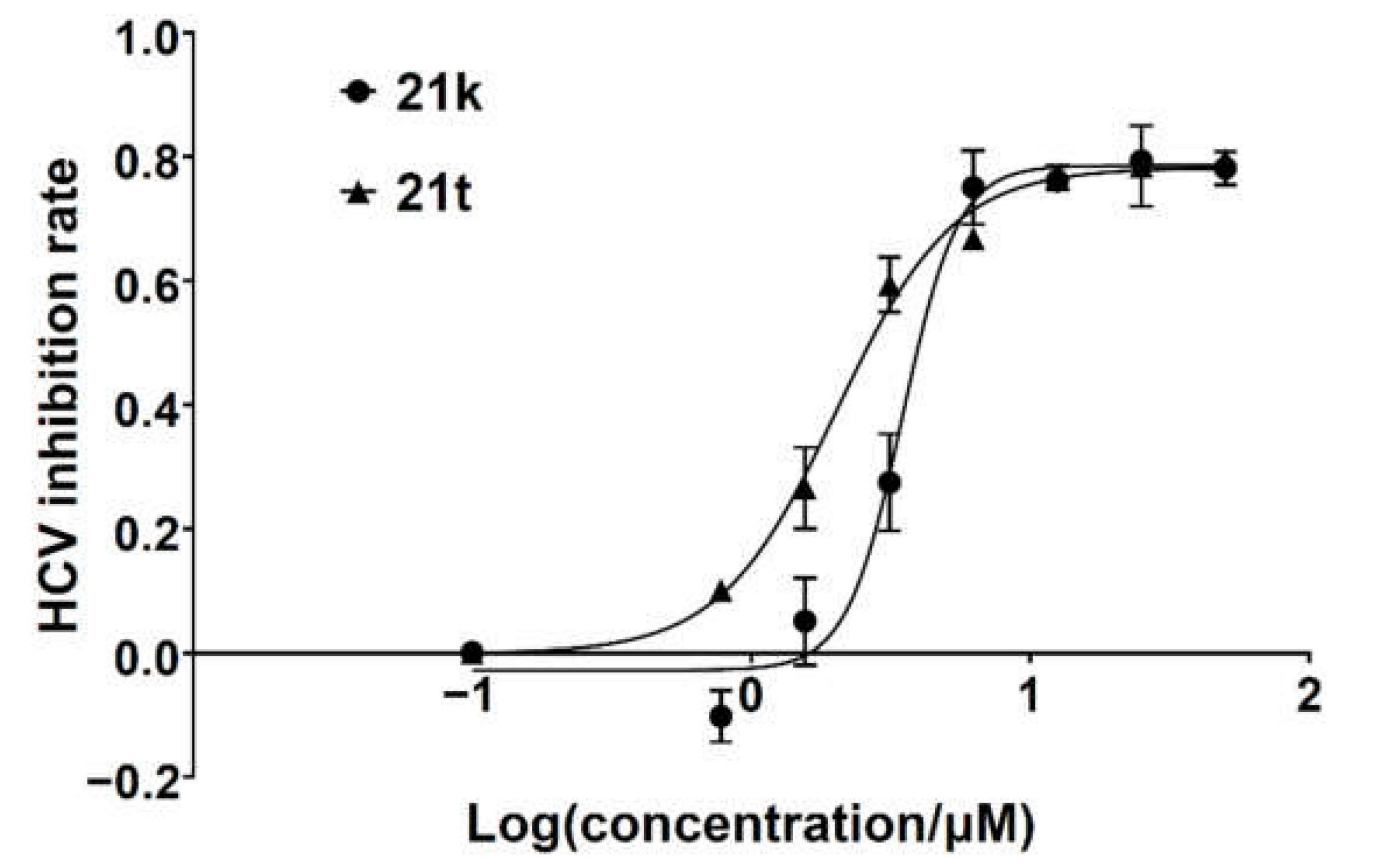
HCV inhibition fitting curves of compounds **21k** and **21t**.

Unlike the benzyl substituents, when the C-7 position of the parent nucleus was substituted with phenyl (**21e**), the anti-HCV activity of the compound was significantly improved (78.1% inhibitory rate at 10 μM). Based on these results, various aryl substituents at the C-6, C-7, or 8-position of the hydroxyquinazolinedione core were introduced to observe their effects on inhibiting HCV replication. It should be noted that compounds **21a**–**d** with C-6 aryl groups showed significant cytotoxicity. For instance, the therapeutic index of **21d** with *m*-nitrophenyl at the C-6 position was only 1.4. Interestingly, as compared to **21d**, the C-7 regioisomer **21k** had better HCV inhibitory activity (EC_50_ = 3.5 μM) and significantly improved therapeutic index (TI = 11.7). These encouraging results led to the further exploration of different C-7 aryl groups on the parent nucleus. Most of the compounds **21e**–**p** substituted with phenyl groups at the C-7 position showed good antiviral activities with EC_50_ values below 10 μM. Among the C-7 substituents, disubstituted phenyl groups had no advantage over monosubstituted ones for anti-HCV potency, while the C-7 trisubstituted phenyl led to the near loss of activity (**21q**). It is remarkable that, in comparison with an electron-donating group, when the C-7 aromatic substituent contains an electron-withdrawing group, the compound is less toxic to the tested cells and has a better therapeutic index. For example, no cytotoxicity was observed in the tested concentrations of **21h** with trifluoromethyl at the C-7 position (TI > 5.2). In the cases of **21k** bearing nitro group and **21l** bearing chlorine group, the therapeutic indexes were 11.7 and 5.2, respectively. The cytotoxicity profiles of compounds **21h**, **21k**, and **21l** were distinctly better than those of **21g**, **21i**, **21j**, and **21o**, in which C-7 phenyl substituents contain electron-donating groups such as amino and methoxy (TI = 1.1–1.8). Compared with ribavirin (TI = 2.3, EC_50_ = 20.0 μM) tested under the same condition, **21h**, **21k**, **21l**, and **21n** all have better therapeutic windows and HCV inhibitory activities. At the C-7 position of the parent nucleus, we also tried some heteroaryl groups, such as the pyridine group of **21r**, the furan group of **21s**, and the benzofuran group of **21t**. The HCV inhibitory rate of **21r** at 10 μM turned out to be 26.9%, which showed no advantage over other compounds possessing C-7 aryl substituents. The reason could be that the introduction of the pyridine group increased the hydrophilicity of the compound and thus reduced the cell membrane permeability. The activity of C-7 furyl substituted **21s** (EC_50_ = 4.0 μM) is comparable to that of C-7 phenyl substituted **21e** (EC_50_ = 3.2 μM). Intriguingly, **21s** has a therapeutic index of 4.1, which is better than **21e** (TI = 1.7), suggesting that the furyl group is beneficial in reducing the cytotoxicity. This result was also confirmed by compound **21t,** which contains a benzofuranyl group at the C-7 position. No cytotoxic effect was observed for **21t** at the tested concentrations. On the other hand, the HCV inhibitory activity of **21t** (EC_50_ = 2.0 μM) is also superior to other synthesized compounds, rendering the therapeutic index of **21t** greater than 25. It is worth mentioning that **21t** showed a comparable EC_50_ value to compound **6** (EC_50_ = 1.9 μM), which is one of the reported metal ion chelators with the best anti-HCV activity at the cellular level. More importantly, no obvious cytotoxic effect was observed at the tested concentration of **21t** (TI > 25), leading to a better therapeutic index (TI > 25) than that of compound **6** (TI = 6.8) [38]. The EC_50_ fitting curves of representative compounds **21k** and **21t** measured using HCV 1b replicon cells are depicted in Figure 2. The fitting graph validated that the HCV inhibitory rate of the compounds has a gradient increasing relationship with the concentration.

Compared with the unsubstituted **10a**, C-8 aryl-substituted compounds exhibited no significant improvement in the HCV inhibitory activity. For example, the HCV inhibitory rates of compounds **21u**–**x** at 25 μM did not exceed 50%, whether the introduced C-8 substituents had an electron-donating group (**21w**) or an electron-withdrawing group (**21x**). Methylation of **21d**–**f** and **21i** at the N-1 position gave the compounds **23a**–**d**, while the EC_50_ values did not change significantly. However, the cytotoxicity profiles of the methylated products **23a**–**d** were slightly better. The introduction of the ethyl group at the N-1 position of **21e** (EC_50_ = 3.2 μM**)** greatly reduced the anti-HCV potency, which is exemplified by compound **23e** (EC_50_ = 26.8 μM). The same situation occurred in **23f**–**i**, in which the N-1 position is substituted by a propyl or benzyl group. These results indicated that when the phenyl ring of the hydroxyquinazolinedione core is substituted by aryl groups, sterically large substituents may not be preferred in improving the anti-HCV activity. However, compound **23j** is an exception to this rule. One possible reason is that **23j** adopts a different binding mode with the target. Overall, the SAR study elucidated the characteristics of the HCV inhibitory profile of 3-hydroxyquinazoline-2,4(1*H*,3*H*)-diones. These findings also verified the feasibility of the metal ion chelation strategy in the development of novel anti-HCV drugs.

### 2.3. Thermal Shift Assay

**Figure 3:**
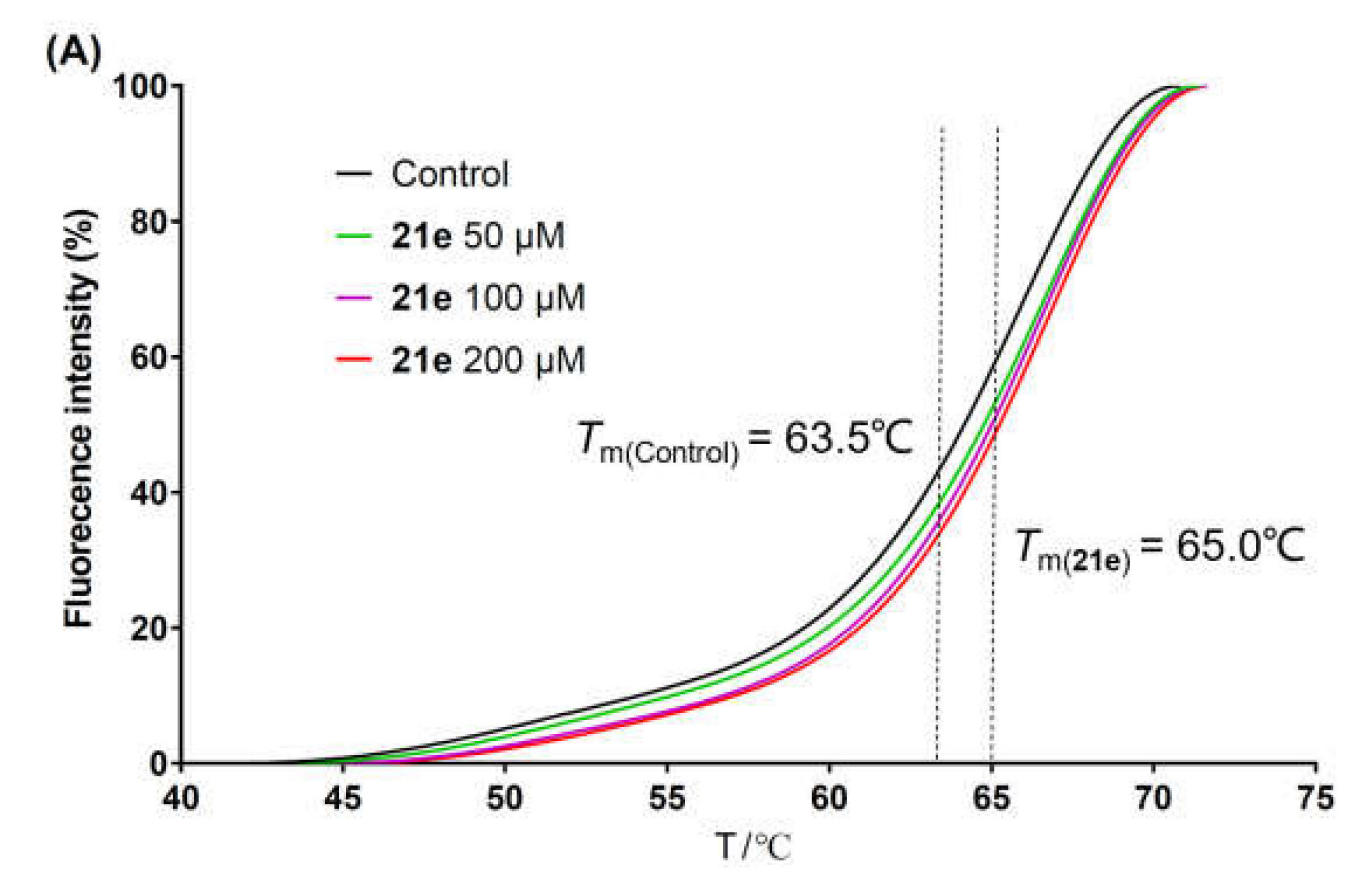

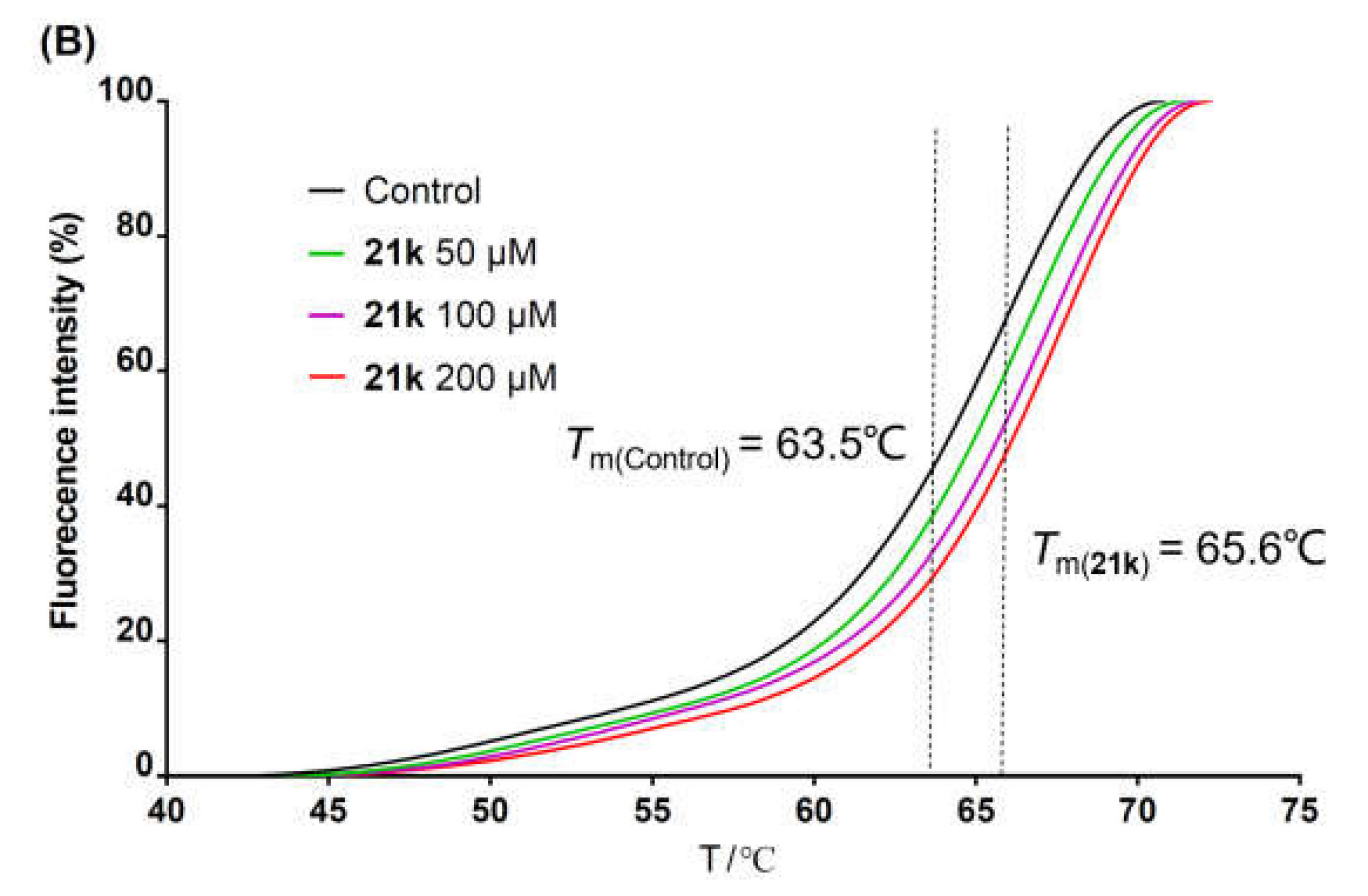
Fluorescence intensity change versus temperature of NS5B complexed with different concentrations of **21e** (**A**) or **21k** (**B**).

The stability of a protein system is enhanced after binding to its specific ligand, leading to an increase in the melting temperature (*T*_m_). Taking advantage of this feature, the thermal shift assay was extensively used to assess the binding of small molecules to the protein targets [51,52,53]. The metal ion chelators were thought to exert an anti-HCV effect by chelating with Mg^2+^ in the catalytic center of NS5B [34,36,38]. Herein, to evaluate the binding of 3-hydroxyquinazoline-2,4(1*H*,3*H*)-dione derivatives with NS5B protein, representative compounds **21e** and **21k** with superior anti-HCV activities or good therapeutic indexes were selected and subjected to the thermal shift assay. C-terminal His-tagged NS5BΔ21 from HCV 1b subtype was used as the protein in the experiment. Compared with the blank control (DMSO), the fluorescence–temperature curves of the NS5B system in the presence of **21e** (Figure 3A) and **21k** (Figure 3B) at 50 μM, 100 μM, and 200 μM all shifted to the right of the axis. The *T*_m_ values of **21e** and **21k** increased in a concentration-dependent manner, presenting higher values than that of the blank control (*T*_m_ = 63.5 °C) at all tested concentrations. The addition of 200 μM **21e** or **21k** to the NS5B protein solution resulted in a shift in the melting temperature (Δ*T*_m_) with a value of 1.5 °C or 2.1 °C, respectively (Table 3), validating the binding abilities of **21e** and **21k** to NS5B. The co-crystal structure of NS5B complexed with ADP (PDB code: 4WTD) [8] proved that ADP could bind to the catalytic center of NS5B. Hence, ADP was chosen as the positive control in the thermal shift assay. As listed in Table 3, under the same experimental conditions, the Δ*T*_m_ values of protein–ligand complexes containing ADP at the concentrations of 50 μM, 100 μM, and 200 μM were 0.44 °C, 0.62 °C, and 0.87 °C, respectively, which were significantly lower than the corresponding concentration groups of compounds **21e** and **21k**. These data indicated that the affinities of **21e** and **21k** to NS5B protein might be better than that of ADP. The thermal shift assay suggested that the metal ion chelators with 3-hydroxyquinazoline-2,4(1*H*,3*H*)-dione parent nucleus could inhibit the replication of HCV by binding to NS5B protein.

### 2.4. Molecular Docking

In order to provide detailed insights into the possible binding mode of 3-hydroxyquinazoline-2,4(1*H*,3*H*)-diones with HCV NS5B polymerase and to interpret the potential reasons for the structure–activity relationship of the compounds, the 7-phenyl substituted compound **21e** was docked into the crystal structure of NS5B 1b subtype (PDB code: 1GX6) [9] using the Schrödinger software package [54]. Before the docking studies, the original ligand UTP in 1GX6 was removed. In addition, the structures of NS5B protein and **21e** were optimized by Schrödinger. The best scoring docking model was subjected to subsequent binding analysis.

**Figure 4:**
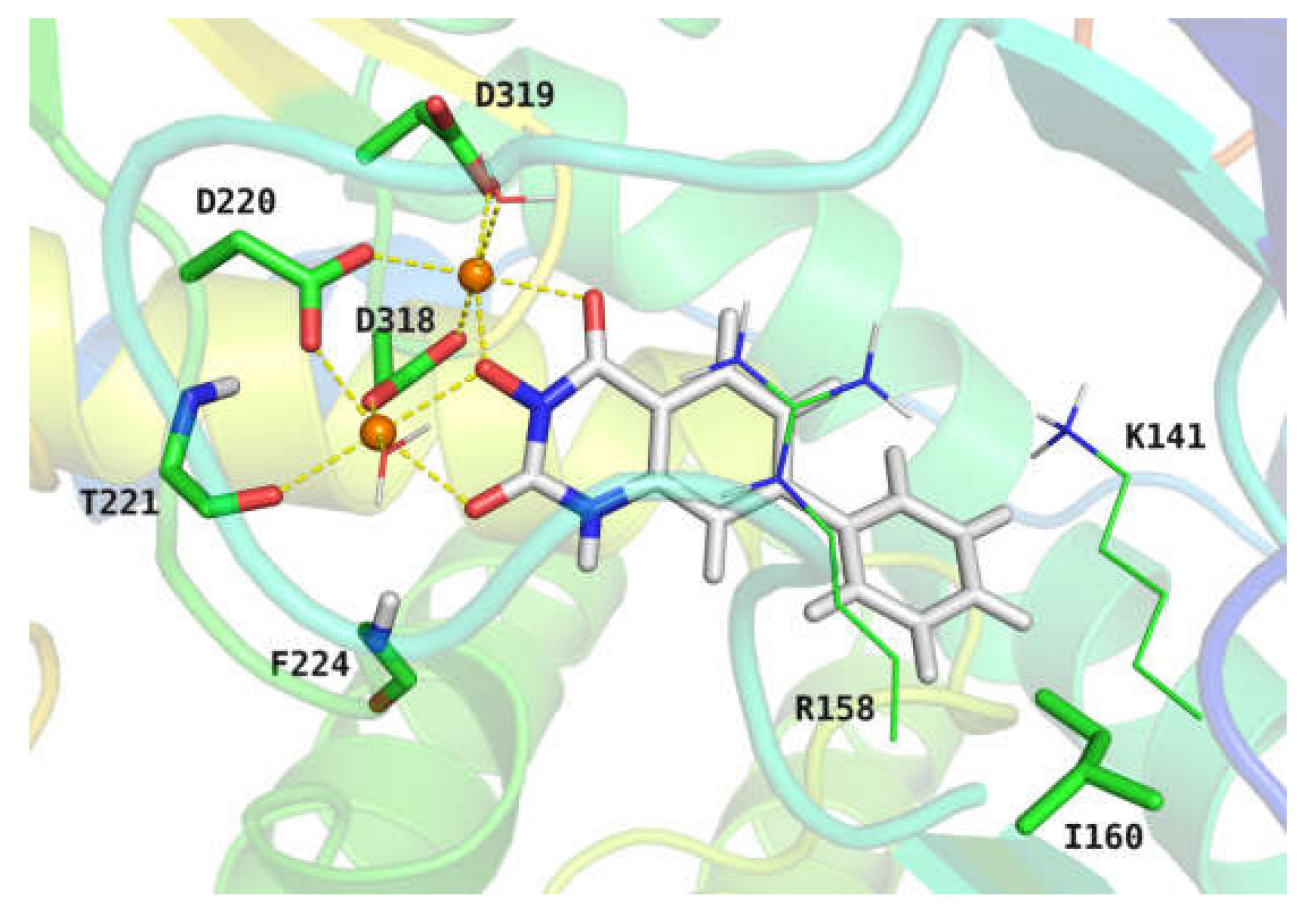
Binding model of compound **21e** with NS5B protein from molecular docking study. The central two Mg^2+^ ions are shown in orange spheres. The ligand molecule is shown in sticks with carbon atoms and hydrogen atoms colored grey. The key amino acid residues and two water molecules in the catalytic center are presented in sticks or lines with non-polar hydrogen atoms hidden for clarity. The chelation interactions of Mg^2+^ with the ligand, water molecules, and central amino acid residues are shown in golden dash lines.

From the docking model (Figure 4), it can be seen that the two Mg^2+^ ions in the NS5B protein maintain the core conformation of the catalytic center in a “hexadentate coordination” mode. The side chain of amino acid residues D318, D319, and D220, the main chain of T221, and two water molecules together form a chelation complex with the central metal ions. The oxygen at the N-3 position and the diketone carbonyl group of compound **21e** occupy the remaining coordination space of Mg^2+^. The distance between Mg^2+^ and the oxygen atoms involved in chelation in the simulation model is 2.2–2.4 Å, which is very close to the corresponding distance (2.3 Å) between the phosphate group of UTP and the metal ions in the co-crystal structure (1GX6). In addition, one of the carbonyl oxygen atoms of **21e** chelated with Mg^2+^ also forms a hydrogen bond with the main chain of residue F224, maintaining the chelation stability of the compound in the active center of NS5B. It is worth noting that the positive charge center of the side chain of R158 is in the vertical direction of the hydroxyquinazolinedione core of **21e**, contributing a cation–π interaction with the nuclear parent of **21e**. Interestingly, the cation–π interaction could also be observed between the positively charged amino in the side chain of K141 and the 7-phenyl ring of compound **21e**. In fact, such interactions were widely observed among structures in the Protein Data Bank (PDB) [55]. In the aforementioned anti-HCV SAR studies of 3-hydroxyquinazoline-2,4(1*H*,3*H*)-diones, we found that the C-6 or C-7 aryl substituents are beneficial to the improvement of HCV inhibitory activity. This finding could be attributed to the cation–π interactions observed in the docking model. Meanwhile, the docking results showed that the C-7 phenyl group of the compound also had a van der Waals interaction with the hydrophobic residue I160. As shown in Table 2, compound **21t** with benzofuranyl at the C-7 position had the best HCV inhibitory activity (EC_50_ = 2.0 μM) and therapeutic index (TI > 25) among the tested compounds. According to the docking model, the superior performance of **21t** might be because the substitution of a larger aromatic ring at the C-7 position is advantageous to improving the cation–π interaction with K141 and the van der Waals interaction with I160. The docking conformation also suggested that the C-8 position of the hydroxyquinazolinedione core is spatially farther from the pocket formed by K141 and I160 as compared to the C-7 position, which might be the reason why C-8 aromatic substituents have no apparent contribution to the HCV inhibitory activities of 3-hydroxyquinazoline-2,4(1*H*,3*H*)-dione derivatives. The molecular simulation results are in accordance with the SAR studies from multiple perspectives, indicating the mechanism by which the compounds exert their anti-HCV efficacy.

### 2.5. Metal Ion Chelation Assay Using UV-Vis Spectrophotometry

**Figure 5:**
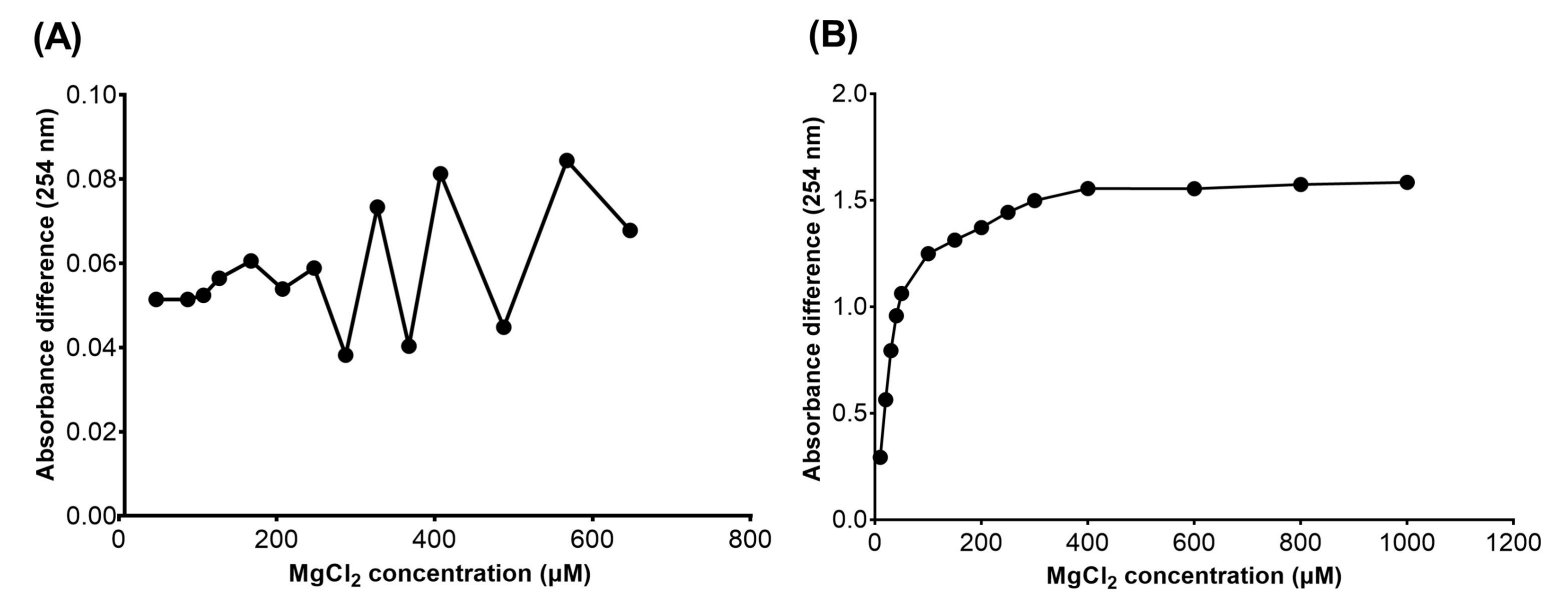
Absorbance increasement at 254 nm of the methanol solution containing 50 μM **21e** in the presence (**A**) or absence (**B**) of NaOAc caused by the addition of ascending concentrations of MgCl_2_. (**A**) Absorbance difference at 254 nm obtained by subtracting the absorbance of the control group (50 μM **21e**) from that of the sample group (50 μM **21e**, 40–640 μM MgCl_2_) containing MgCl_2_. (**B**) Absorbance difference at 254 nm obtained by subtracting the absorbance of the control group (50 μM **21e**, 5 mm NaOAc) from that of the sample group (50 μM **21e**, 5 mm NaOAc, 10–1000 μM MgCl_2_) containing MgCl_2_.

The chelation rules of the 3-hydroxyquinazoline-2,4(1*H*,3*H*)-diones with magnesium ions were explored by UV-Vis spectrophotometry, which was widely used to study the binding properties of compounds with metal ions in vitro [56,57,58]. For the sake of eliminating the interference of heteroaryl groups on the determination of metal ion chelation, the representative compound **21e** with phenyl group substituted at the C-7 position was selected to study its chelation ability with Mg^2+^ under different conditions. As illustrated in Figure 5A, the increase in absorbance of **21e** methanol solution at 254 nm after the addition of ascending concentrations of MgCl_2_ was analyzed. The results revealed that with the increase in Mg^2+^ concentration, the value of absorbance difference at 254 nm between the control group and sample group was always no more than 0.1 even if the concentration of MgCl_2_ was up to 640 μM, and no concentration dependence was observed. This is probably because compound **21e** mainly exists in the free molecular state with the N-hydroxyl group protonated in methanol, which is difficult to chelate with Mg^2+^. Intriguingly, after the introduction of 5 mm NaOAc to the methanol solution containing **21e**, the effect of MgCl_2_ on the absorbance of the system totally changed (Figure 5B). Compared to the control group containing 50 μM **21e** and 5 mm NaOAc, the sample group, which had additional MgCl_2_, showed significantly higher absorbance. Remarkably, merely 20 μM MgCl_2_ was able to increase the absorbance by more than 0.5. In the presence of NaOAc, higher concentrations of MgCl_2_ resulted in larger changes in absorbance at 254 nm, with equilibrium reached at about 400 μM Mg^2+^. A reasonable logic is that the addition of NaOAc raised the pH of the methanol solution, resulting in partial ionization of compound **21e** at the N-hydroxyl group. Subsequently, **21e** in the ionic state formed a chelation complex with Mg^2+^, which increased the absorbance of the solution.

**Figure 6:**
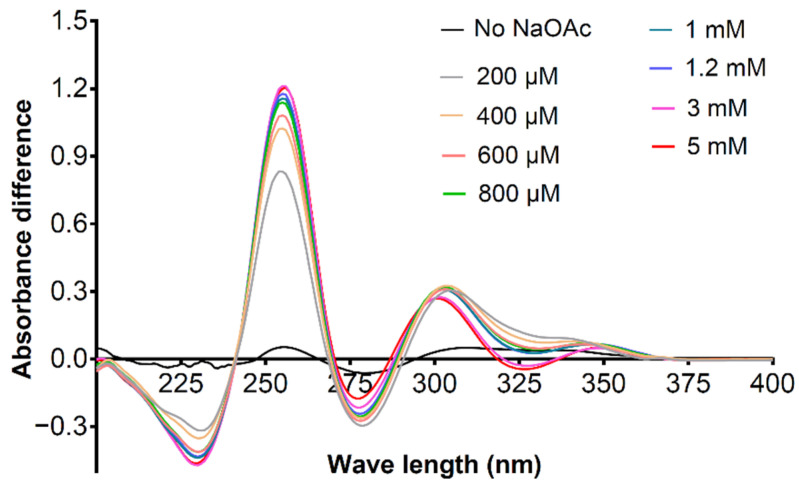
The differential ultraviolet-visible (UV-Vis) spectra caused by the addition of 80 μM MgCl_2_ in the presence of 0–5 mm NaOAc. The differential spectra were obtained by subtracting the absorbance of the control group (50 μM **21e**, 0–5 mm NaOAc) from that of the sample group (50 μM **21e**, the same concentration of NaOAc as in the control group, 80 μM MgCl_2_) at the corresponding wavelength (200–400 nm). Methanol was used as the solvent.

With the aim of verifying whether **21e** has the ability to chelate with Mg^2+^ in the ionic state, we further investigated the influence of various concentrations of NaOAc on the differential UV-Vis spectra caused by the addition of MgCl_2_. As shown in Figure 6, when the system contained no NaOAc, 80 μM MgCl_2_ could not effectively change the UV-Vis spectrum of the solution containing **21e**. Nevertheless, in the presence of ascending concentrations of NaOAc (from 200 μM to 5 mm), compared with the control group (50 μM **21e**, 200 μM to 5 mm NaOAc), the UV spectra of the sample group (50 μM **21e,** the same concentration of NaOAc as in the control group, 80 μM MgCl_2_) containing MgCl_2_ changed significantly, with the absorbance at 254 nm increased by 0.8–1.2. Moreover, the magnitude of the variation in the spectrum is proportional to the concentration of NaOAc. These findings demonstrated that with the increase of NaOAc concentration in methanol solution, the concentration of N-hydroxyl deprotonated **21e** increased synchronously, leading to a higher amount of chelation complex with Mg^2+^. The comparison between different UV-Vis spectra also verified that compound **21e** could chelate with Mg^2+^ in the N-hydroxyl deprotonated form rather than the free molecular state. The UV-Vis spectra studies illustrated the metal ion chelating properties of 3-hydroxyquinazoline-2,4(1*H*,3*H*)-diones represented by compound **21e**, suggesting that these compounds in the ionic state may have the ability to chelate the metal ions in the catalytic center of NS5B.

## 3. Materials and Methods

### 3.1. Chemistry

The reagents used in the chemical experiments were purchased from qualified chemical sellers, with purity greater than 95%. Most of the solvents used in the synthesis experiments were obtained from the China National Pharmaceutical Group Corporation. Unless otherwise specified, all solvents used in the reactions are of analytical grade and have not been further processed. Anhydrous solvents such as DMF, DCM, THF, etc., were purchased from the Innovative Technology Solvent Purification System (Innovative Technology Ltd., Hong Kong, China). Some reaction products were purified by flash column chromatography (CombiFlash^®^ EZ Prep, Teledyne ISCO, Lincoln, NE, USA) with 200–300 mesh silica gel (Qingdao Haiyang Chemical Co., Ltd., Qingdao, China).

The nuclear magnetic resonance spectra (^1^H NMR, ^13^C NMR) of the synthesized compounds were recorded by Varian Mercury Plus 400 MHz and Bruker Ascend^TM^ 600 MHz spectrometers with TMS as an internal standard to calibrate the chemical shifts (δ). Deuterated DMSO or Deuterated trifluoroacetic acid purchased from J&K Scientific was used as the solvent for NMR spectroscopy. The purities and molecular weights of the compounds were determined using an Agilent 1100s mass spectrometer and an Agilent 1260 LC-Agilent 6120 MS liquid chromatography-mass spectrometer with an ESI ion source. The mobile phase was a chromatographically pure water/methanol mixture.

#### 3.1.1. Synthesis of Compounds **8** and **10a**

3-(Benzyloxy)quinazoline-2,4(1*H*,3*H*)-dione (**8**). To *O*-benzylhydroxylamine hydrochloride (6.25 g, 39.1 mmol, 1.5 equiv.) was added 5% NaOH aqueous solution (80 mL) and ether (230 mL), and the mixture was stirred at room temperature for 2 h. The layers were left to stand, and the ether layer was washed three times with saturated brine. The ether layer was dried by Na_2_SO_4_ and concentrated to give *O*-benzylhydroxylamine (4.8 g, 100%), which was stored at 4 °C for later use. A suspension of methyl anthranilate (3.95 g, 26.1 mmol, 1.0 equiv.) and CDI (5.3 g, 32.6 mmol, 1.25 equiv.) in toluene (240 mL) was heated under reflux for 2 h. After the reaction mixture was cooled, the above prepared *O*-benzylhydroxylamine (4.8 g, 39.1 mmol, 1.5 equiv.) was added, and the suspension was heated under reflux for 4 h and then evaporated. Subsequently, ethanol (70 mL) and 2 mol/L NaOH aqueous solution (18 mL) were added to the flask, and the mixture was refluxed for 2 h. After cooling, 15% volume fraction of acetic acid aqueous solution (240 mL) was slowly added to the reaction mixture. The white precipitate was filtrated and recrystallized with methanol (40 mL) to obtain **8** as a white solid (4.2 g, 60% in all steps). MS (ESI) *m*/*z*: 269.1 [M + H]^+^. ^1^H NMR (400 MHz, DMSO-*d*_6_) δ 8.08 (dd, *J* = 8.7, 1.5 Hz, 1H), 7.54 (td, *J* = 7.9, 1.5 Hz, 1H), 7.38–7.31 (m, 4H), 7.35–7.17 (m, 4H), 5.01 (s, 2H).

3-Hydroxyquinazoline-2,4(1*H*,3*H*)-dione (**10a**). To compound **8** (100 mg, 0.373 mmol, 1.0 equiv.) was added 10 wt.% loading palladium–carbon catalyst (10% of the mass of **8**), THF (4 mL), and methanol (1 mL). The solution was stirred under hydrogen (1 atm) at room temperature for 8 h until the raw material **8** was completely converted. The palladium–carbon catalyst was removed by filtration. The filtrate was evaporated to dryness, recrystallized with methanol to give **10a** as a white solid (38 mg, 57%). MS (ESI) *m*/*z*: 177.1 [M−H]^−^. ^1^H NMR (400 MHz, DMSO-*d*_6_) δ 11.51 (s, 1H), 10.56 (s, 1H), 7.95 (d, *J* = 8.0 Hz, 1H), 7.66 (t, *J* = 7.8 Hz, 1H), 7.27–7.12 (m, 2H). ^13^C NMR (151 MHz, DMSO-*d*_6_) δ 159.23, 148.58, 138.18, 134.53, 126.90, 122.36, 115.10, 113.97.

The ESI-MS, ^1^H NMR, and ^13^C NMR spectra of **10a** are shown in Supplementary Materials (Figures S1–S4).

#### 3.1.2. Synthesis of Compounds **10b**–**p**

A suspension of compound **8** (300 mg, 1.12 mmol, 1.0 equiv.), K_2_CO_3_ (309.6 mg, 2.24 mmol, 2.0 equiv.), the corresponding halide (1.34 mmol, 1.2 equiv.) was stirred in DMF (3 mL) at 80 °C for 2 h. After cooling, the reaction mixture was poured into water. The precipitate was washed with water and ether and dried to give the crude product **9a**–**o**, respectively. Debenzylation reactions of **9a**–**o** were subsequently conducted using two different conditions to obtain **10b**–**p**, respectively.

For **10b**–**e**: to compound **9a**–**d** (0.388 mmol, 1.0 equiv.) was added 48% hydrobromic acid (1.5 mL) and glacial acetic acid (1.5 mL). The solution was refluxed for 2 h until the complete conversion of **9a**–**d**. The reaction mixture was cooled to 0 °C under an ice bath and neutralized with 1 mol/L sodium hydroxide aqueous solution. The precipitate was collected by filtration, washed with water and diethyl ether, dried, and re-slurried or recrystallized with a mixture of ethyl acetate/methanol to obtain **10b**–**e** as a solid, respectively.

For **10f**–**p**: A suspension of compound **9e**–**o** (0.233 mmol, 1.0 equiv.), 10 wt.% loading palladium–carbon catalyst (10% of the mass of **9e**–**o**), THF (4 mL), and methanol (1 mL) was stirred under hydrogen (1 atm) at room temperature for 4–12 h until the raw material **9e**–**o** was completely consumed. The catalyst was removed by filtration. The filtrate was evaporated to dryness, recrystallized or re-slurried with methanol, and dried to give **10f**–**p** as a solid, respectively.

3-Hydroxy-1-(3-phenylpropyl)quinazoline-2,4(1*H*,3*H*)-dione (**10b**). According to the above general procedure with 1-bromo-3-phenylpropane as the halide in the reactants, the crude **9a** was obtained as a white solid. MS (ESI) *m*/*z*: 387.0 [M + H]^+^. Subsequent debenzylation in the mixture of hydrobromic acid/glacial acetic acid gave **10b** as a pale yellow solid (82.2 mg, 55% over two steps). MS (ESI) *m*/*z*: 294.9 [M−H]^−^. ^1^H NMR (400 MHz, DMSO-*d*_6_) δ 8.00 (d, *J* = 7.8 Hz, 1H), 7.66 (d, *J* = 8.0 Hz, 1H), 7.35 (d, *J* = 8.4 Hz, 1H), 7.31–7.07 (m, 6H), 4.09 (t, *J* = 7.7 Hz, 2H), 2.67 (d, *J* = 8.2 Hz, 2H), 1.89 (t, *J* = 8.6 Hz, 2H). ^13^C NMR (151 MHz, DMSO-*d*_6_) δ 158.71, 149.74, 141.08, 137.98, 134.21, 128.51, 128.25, 128.17, 128.09, 127.36, 125.74, 122.30, 115.13, 114.14, 42.74, 31.98, 28.40.

1-(2-Chlorophenethyl)-3-hydroxyquinazoline-2,4(1*H*,3*H*)-dione (**10c**). According to the above general procedure with 1-(2-bromoethyl)-2-chlorobenzene as the halide in the reactants, the crude **9b** was obtained as a white solid. MS (ESI) *m*/*z*: 406.9 [M + H]^+^. Subsequent debenzylation in the mixture of hydrobromic acid/glacial acetic acid gave **10c** as a pale yellow solid (43 mg, 18% over two steps). MS (ESI) *m*/*z*: 315.1 [M−H]^−^. ^1^H NMR (400 MHz, DMSO-*d*_6_) δ 10.76 (s, 1H), 8.07 (d, *J* = 7.5 Hz, 1H), 7.74 (t, *J* = 7.6 Hz, 1H), 7.46 (d, *J* = 8.2 Hz, 1H), 7.40 (d, *J* = 7.2 Hz, 2H), 7.29 (dt, *J* = 14.7, 6.9 Hz, 3H), 4.37 (t, *J* = 7.5 Hz, 2H), 3.09 (t, *J* = 7.3 Hz, 2H). ^13^C NMR (151 MHz, DMSO-*d*_6_) δ 158.28, 148.91, 138.39, 135.50, 134.87, 133.10, 131.35, 129.13, 128.55, 127.67, 127.36, 122.73, 114.89, 114.25, 42.87, 30.58.

1-(4-Fluorophenethyl)-3-hydroxyquinazoline-2,4(1*H*,3*H*)-dione (**10d**). According to the above general procedure with 1-(2-bromoethyl)-4-fluorobenzene as the halide in the reactants, the crude **9c** was obtained as a white solid. MS (ESI) *m*/*z*: 391.1 [M + H]^+^. Subsequent debenzylation in the mixture of hydrobromic acid/glacial acetic acid gave **10d** as a pale yellow solid (60 mg, 65% over two steps). MS (ESI) *m*/*z*: 299.0 [M−H]^−^. ^1^H NMR (400 MHz, DMSO-*d*_6_) δ 10.76 (s, 1H), 8.08 (d, *J* = 8.0 Hz, 1H), 7.92–7.71 (m, 1H), 7.56 (d, *J* = 8.7 Hz, 1H), 7.49–7.27 (m, 3H), 7.23–7.07 (m, 2H), 4.33 (d, *J* = 7.7 Hz, 2H), 2.95 (d, *J* = 7.4 Hz, 2H). ^13^C NMR (151 MHz, DMSO-*d*_6_) δ 161.74, 160.14, 158.29, 148.90, 138.32, 134.98, 134.03, 130.66, 130.61, 127.64, 122.78, 115.12, 114.98, 114.86, 114.67, 44.34, 31.94.

3-Hydroxy-1-(2-oxo-2-phenylethyl)quinazoline-2,4(1*H*,3*H*)-dione (**10e**). According to the above general procedure with 2-bromo-1-phenylethan-1-one as the halide in the reactants, the crude **9d** was obtained as a white solid. MS (ESI) *m*/*z*: 386.9 [M + H]^+^. Subsequent debenzylation in the mixture of hydrobromic acid/glacial acetic acid gave **10e** as a pale yellow solid (30 mg, 27% over two steps). MS (ESI) *m*/*z*: 295.0 [M−H]^−^. ^1^H NMR (400 MHz, DMSO-*d*_6_) δ 10.90 (s, 1H), 8.14 (d, *J* = 7.2 Hz, 3H), 7.85–7.57 (m, 4H), 7.43–7.26 (m, 2H), 5.82 (s, 2H). ^13^C NMR (151 MHz, DMSO-*d*_6_) δ 192.91, 158.44, 149.36, 139.06, 135.01, 134.15, 128.85 (2C), 128.17 (2C), 127.58, 123.02, 114.75, 114.61, 50.12.

2-(3-Hydroxy-2,4-dioxo-3,4-dihydroquinazolin-1(2*H*)-yl)-*N*-phenethylacetamide (**10f**). According to the above general procedure with 2-bromo-*N*-phenethylacetamide as the halide in the reactants, the crude **9e** was obtained as a white solid. MS (ESI) *m*/*z*: 430.2 [M + H]^+^. Subsequent debenzylation by catalytic hydrogenation gave **10f** as a white solid (36.3 mg, 35% over two steps). MS (ESI) *m*/*z*: 338.1 [M−H]^−^. ^1^H NMR (400 MHz, DMSO-*d*_6_) δ 10.89 (s, 1H), 8.36 (t, *J* = 5.9 Hz, 1H), 8.05 (d, *J* = 7.5 Hz, 1H), 7.70 (t, *J* = 7.7 Hz, 1H), 7.28 (t, *J* = 8.0 Hz, 3H), 7.20 (t, *J* = 8.6 Hz, 3H), 7.11 (d, *J* = 7.9 Hz, 1H), 4.72 (d, *J* = 4.9 Hz, 2H), 3.32 (d, *J* = 7.3 Hz, 2H), 2.71 (dd, *J* = 10.0, 4.6 Hz, 2H). ^13^C NMR (151 MHz, DMSO-*d*_6_) δ 166.20, 158.55, 149.50, 139.12, 138.95, 134.68, 128.55 (2C), 128.20 (2C), 127.38, 125.99, 122.79, 114.92, 114.30, 45.89, 40.21, 34.89.

*N*-(Cyclopropylmethyl)-2-(3-hydroxy-2,4-dioxo-3,4-dihydroquinazolin-1(2*H*)-yl)acetamide (**10g**). According to the above general procedure with 2-bromo-*N*-(cyclopropylmethyl)acetamide as the halide in the reactants, the crude **9f** was obtained as a white solid. MS (ESI) *m*/*z*: 380.2 [M + H]^+^. Subsequent debenzylation by catalytic hydrogenation gave **10g** as a white solid (85 mg, 79% over two steps). MS (ESI) *m*/*z*: 288.1 [M−H]^−^. ^1^H NMR (400 MHz, DMSO-*d*_6_) δ 8.33 (s, 1H), 8.00 (d, *J* = 6.8 Hz, 1H), 7.69 (s, 1H), 7.26 (d, *J* = 7.5 Hz, 1H), 7.17 (d, *J* = 8.1 Hz, 1H), 4.73 (s, 2H), 2.97 (s, 2H), 2.50 (s, 2H), 0.88 (s, 1H), 0.38 (d, *J* = 5.7 Hz, 2H), 0.13 (s, 2H). ^13^C NMR (151 MHz, DMSO-*d*_6_) δ 166.23, 158.80, 149.91, 138.78, 134.23, 127.21, 122.53, 115.06, 114.16, 45.84, 42.81, 10.58, 3.06 (2C).

*N*-Benzyl-2-(3-hydroxy-2,4-dioxo-3,4-dihydroquinazolin-1(2*H*)-yl)acetamide (**10h**). According to the above general procedure with *N*-benzyl-2-bromoacetamide as the halide in the reactants, the crude **9g** was obtained as a white solid. MS (ESI) *m*/*z*: 416.1 [M + H]^+^. Subsequent debenzylation by catalytic hydrogenation gave **10h** as a pale yellow solid (90 mg, 81% over two steps). MS (ESI) *m*/*z*: 324.1 [M−H]^−^. ^1^H NMR (400 MHz, DMSO-*d*_6_) δ 10.95 (s, 1H), 8.78 (s, 1H), 8.06 (d, *J* = 7.6 Hz, 1H), 7.73 (s, 1H), 7.31 (t, *J* = 8.0 Hz, 3H), 7.27–7.19 (m, 4H), 4.84 (s, 2H), 4.30 (s, 2H). ^13^C NMR (151 MHz, DMSO-*d*_6_) δ 166.49, 158.61, 149.57, 138.90, 134.63, 128.14, 127.38 (2C), 126.99 (2C), 126.71, 122.84, 114.98, 114.41, 46.03, 42.02.

*N*-Butyl-2-(3-hydroxy-2,4-dioxo-3,4-dihydroquinazolin-1(2*H*)-yl)acetamide (**10i**). According to the above general procedure with 2-bromo-*N*-butylacetamide as the halide in the reactants, the crude **9h** was obtained as a white solid. MS (ESI) *m*/*z*: 382.2 [M + H]^+^. Subsequent debenzylation by catalytic hydrogenation gave **10i** as a white solid (19 mg, 11% over two steps). MS (ESI) *m*/*z*: 290.2 [M−H]^−^. ^1^H NMR (400 MHz, DMSO-*d*_6_) δ 10.82 (s, 1H), 8.20 (d, *J* = 5.7 Hz, 1H), 8.07 (d, *J* = 7.9 Hz, 1H), 7.73 (t, *J* = 7.8 Hz, 1H), 7.31 (t, *J* = 7.7 Hz, 1H), 7.19 (d, *J* = 7.8 Hz, 1H), 4.73 (s, 2H), 3.06 (p, *J* = 5.6, 5.0 Hz, 2H), 1.37 (t, *J* = 8.0 Hz, 2H), 1.24 (q, *J* = 7.5 Hz, 2H), 0.90–0.78 (m, 3H). ^13^C NMR (151 MHz, DMSO-*d*_6_) δ 166.03, 158.51, 149.41, 139.05, 134.74, 127.43, 122.84, 114.90, 114.37, 45.92, 38.18, 30.97, 19.33, 13.52.

2-(3-Hydroxy-2,4-dioxo-3,4-dihydroquinazolin-1(2*H*)-yl)-*N*-phenylacetamide (**10j**). According to the above general procedure with 2-bromo-*N*-phenylacetamide as the halide in the reactants, the crude **9i** was obtained as a white solid. MS (ESI) *m*/*z*: 402.1 [M + H]^+^. Subsequent debenzylation by catalytic hydrogenation gave **10j** as a white solid (13 mg, 7% over two steps). MS (ESI) *m*/*z*: 310.1 [M−H]^−^. ^1^H NMR (400 MHz, DMSO-*d*_6_) δ 10.89 (s, 1H), 10.38 (s, 1H), 8.10 (d, *J* = 7.0 Hz, 1H), 7.76 (t, *J* = 8.0 Hz, 1H), 7.57 (d, *J* = 6.8 Hz, 2H), 7.40 (d, *J* = 7.9 Hz, 1H), 7.32 (d, *J* = 6.7 Hz, 3H), 7.08 (d, *J* = 7.1 Hz, 1H), 5.00 (s, 2H). ^13^C NMR (151 MHz, DMSO-*d*_6_) δ 165.13, 158.41, 149.42, 139.15, 138.39, 134.89, 128.67 (2C), 127.45, 123.42, 122.90, 119.01 (2C), 114.62, 114.57, 46.30.

*N*-(2,6-Dichlorobenzyl)-2-(3-hydroxy-2,4-dioxo-3,4-dihydroquinazolin-1(2*H*)-yl)acetamide (**10k**). According to the above general procedure with 2-bromo-*N*-(2,6-dichlorobenzyl)acetamide as the halide in the reactants, the crude **9j** was obtained as a white solid. MS (ESI) *m*/*z*: 484.1 [M + H]^+^. Subsequent debenzylation by catalytic hydrogenation gave **10k** as a grey solid (28.4 mg, 25% over two steps). MS (ESI) *m*/*z*: 393.0 [M−H]^−^. ^1^H NMR (400 MHz, DMSO-*d*_6_) δ 10.83 (s, 1H), 8.56 (s, 1H), 8.07 (d, *J* = 8.5 Hz, 1H), 7.71 (t, *J* = 8.3 Hz, 1H), 7.50 (d, *J* = 8.2 Hz, 2H), 7.38 (d, *J* = 6.8 Hz, 1H), 7.34–7.24 (m, 1H), 7.18 (d, *J* = 8.8 Hz, 1H), 4.79 (s, 2H), 4.54 (s, 2H). ^13^C NMR (151 MHz, DMSO-*d*_6_) δ 166.11, 158.44, 149.40, 139.02, 135.46 (2C), 134.74, 132.83, 130.27, 128.50 (2C), 127.44, 122.88, 114.78, 114.41, 45.55, 38.80.

*N*-(3-Fluorobenzyl)-2-(3-hydroxy-2,4-dioxo-3,4-dihydroquinazolin-1(2*H*)-yl)acetamide (**10l**). According to the above general procedure with 2-bromo-*N*-(3-fluorobenzyl)acetamide as the halide in the reactants, the crude **9k** was obtained as a white solid. MS (ESI) *m*/*z*: 434.1 [M + H]^+^. Subsequent debenzylation by catalytic hydrogenation gave **10l** as a white solid (40 mg, 30% over two steps). MS (ESI) *m*/*z*: 342.2 [M−H]^−^. ^1^H NMR (400 MHz, DMSO-*d*_6_) δ 10.84 (s, 1H), 8.80 (d, *J* = 6.7 Hz, 1H), 8.08 (d, *J* = 7.8 Hz, 1H), 7.73 (t, *J* = 7.8 Hz, 1H), 7.39–7.24 (m, 3H), 7.05 (dd, *J* = 17.2, 9.0 Hz, 3H), 4.85 (d, *J* = 2.9 Hz, 2H), 4.32 (d, *J* = 6.0 Hz, 2H). ^13^C NMR (151 MHz, DMSO-*d*_6_) δ 166.70, 162.11 (d, *J* = 243.3 Hz), 158.55, 149.49, 142.02 (d, *J* = 7.2 Hz), 139.03, 134.76, 130.10 (d, *J* = 8.4 Hz), 127.49, 122.94, 115.01, 114.46, 113.52.

*N*-(4-Fluorobenzyl)-2-(3-hydroxy-2,4-dioxo-3,4-dihydroquinazolin-1(2*H*)-yl)acetamide (**10m**). According to the above general procedure with 2-bromo-*N*-(4-fluorobenzyl)acetamide as the halide in the reactants, the crude **9l** was obtained as a white solid. MS (ESI) *m*/*z*: 434.1 [M + H]^+^. Subsequent debenzylation by catalytic hydrogenation gave **10m** as a white solid (49.4 mg, 36% over two steps). MS (ESI) *m*/*z*: 342.1 [M−H]^−^. ^1^H NMR (400 MHz, DMSO-*d*_6_) δ 10.84 (s, 1H), 8.79 (d, *J* = 6.0 Hz, 1H), 8.08 (d, *J* = 7.7 Hz, 1H), 7.74 (t, *J* = 7.9 Hz, 1H), 7.32 (t, *J* = 8.0 Hz, 1H), 7.26 (d, *J* = 7.7 Hz, 3H), 7.19–7.09 (m, 2H), 4.83 (s, 2H), 4.28 (d, *J* = 5.7 Hz, 2H). ^13^C NMR (151 MHz, DMSO-*d*_6_) δ 166.53, 161.08 (d, *J* = 242.2 Hz), 158.54, 149.47, 139.03, 135.15, 134.79, 128.99 (d, *J* = 8.3 Hz), 127.47, 122.93, 114.97 (d, *J* = 4.4 Hz), 114.81, 114.44, 46.05, 41.33.

*N*-(4-Cyanophenyl)-2-(3-hydroxy-2,4-dioxo-3,4-dihydroquinazolin-1(2*H*)-yl)acetamide (**10n**). According to the above general procedure with 2-bromo-*N*-(4-cyanophenyl)acetamide as the halide in the reactants, the crude **9m** was obtained as a white solid. MS (ESI) *m*/*z*: 427.1 [M + H]^+^. Subsequent debenzylation by catalytic hydrogenation gave **10n** as a white solid (20 mg, 19% over two steps). MS (ESI) *m*/*z*: 335.1 [M−H]^−^. ^1^H NMR (400 MHz, DMSO-*d*_6_) δ 10.90 (s, 1H), 10.85 (s, 1H), 8.11 (dd, *J* = 7.5, 3.6 Hz, 1H), 7.78 (d, *J* = 11.4 Hz, 5H), 7.47–7.40 (m, 1H), 7.34 (td, *J* = 7.4, 3.6 Hz, 1H), 5.05 (d, *J* = 3.7 Hz, 2H). ^13^C NMR (151 MHz, DMSO-*d*_6_) δ 166.14, 158.40, 149.41, 142.57, 139.10, 134.96, 133.27, 123.00, 119.07, 118.79, 114.61, 105.23, 46.50.

2-(3-Hydroxy-2,4-dioxo-3,4-dihydroquinazolin-1(2*H*)-yl)-*N*-(4-methoxyphenyl)acetamide (**10o**). According to the above general procedure with 2-bromo-*N*-(4-methoxyphenyl)acetamide as the halide in the reactants, the crude **9n** was obtained as a white solid. MS (ESI) *m*/*z*: 432.1 [M + H]^+^. Subsequent debenzylation by catalytic hydrogenation gave **10o** as a white solid (23.8 mg, 18% over two steps). MS (ESI) *m*/*z*: 340.1 [M−H]^−^. ^1^H NMR (400 MHz, DMSO-*d*_6_) δ 10.87 (s, 1H), 10.22 (s, 1H), 8.09 (d, *J* = 7.9 Hz, 1H), 7.74 (t, *J* = 8.2 Hz, 1H), 7.47 (d, *J* = 8.6 Hz, 2H), 7.35 (dd, *J* = 20.2, 8.4 Hz, 2H), 6.88 (d, *J* = 8.4 Hz, 2H), 4.96 (s, 2H), 3.71 (s, 3H). ^13^C NMR (151 MHz, DMSO-*d*_6_) δ 164.60, 158.41, 155.22, 149.41, 139.14, 134.85, 131.47, 127.41, 122.85, 120.59, 114.63, 114.54, 113.73, 54.96, 46.17.

2-(3-Hydroxy-2,4-dioxo-3,4-dihydroquinazolin-1(2*H*)-yl)-*N*-(4-(trifluoromethyl)phenyl)acetamide (**10p**). According to the above general procedure with 2-bromo-*N*-(4-(trifluoromethyl)phenyl)acetamide as the halide in the reactants, the crude **9o** was obtained as a white solid. MS (ESI) *m*/*z*: 470.1 [M + H]^+^. Subsequent debenzylation by catalytic hydrogenation gave **10p** as a white solid (30 mg, 18% over two steps). MS (ESI) *m*/*z*: 378.1 [M−H]^−^. ^1^H NMR (400 MHz, DMSO-*d*_6_) δ 10.91 (s, 1H), 10.77 (s, 1H), 8.10 (d, *J* = 7.8 Hz, 1H), 7.78 (d, *J* = 9.0 Hz, 2H), 7.74 (d, *J* = 7.3 Hz, 1H), 7.69 (d, *J* = 8.4 Hz, 2H), 7.43 (d, *J* = 7.9 Hz, 1H), 7.33 (d, *J* = 7.3 Hz, 1H), 5.05 (s, 2H). ^13^C NMR (151 MHz, Chloroform-*d*) δ 165.95, 158.42, 149.44, 141.95, 139.12, 134.92, 127.48, 126.04, 125.04, 123.54, 123.28 (d, *J* = 12.2 Hz), 122.96, 118.96, 114.61, 46.44.

#### 3.1.3. Synthesis of Compounds **14a**–**c**

To compound **11a**–**c** (37.0 mmol, 1.0 equiv.) was added triphosgene (3.84 g, 12.9 mmol, 0.35 equiv.), and 1,4-dioxane (40 mL). The suspension was refluxed at 110 °C for 6 h and then evaporated to 1/3 of the volume. The precipitate was collected by filtration, washed with petroleum ether (50 mL) and ethyl acetate (10 mL) successively, and dried to give the crude product **12a**–**c**. A suspension of *O*-benzylhydroxylamine hydrochloride (3.72 g, 23.3 mmol, 1.02 equiv.) and triethylamine (2.36 g, 23.3 mmol, 1.02 equiv.) in ethanol (100 mL) was stirred at room temperature for 1 h, and then crude **12a**–**c** (22.9 mmol, 1.0 equiv.) was added. The reaction mixture was refluxed for 3 h, then poured into water (350 mL). The precipitate was collected by filtration, washed with water, and dried to obtain crude **13a**–**c**. Without further purification, crude **13a**–**c** (16.7 mmol, 1.0 equiv.) was stirred with triphosgene (1.98 g, 6.63 mmol, 0.4 equiv.) and triethylamine (4.05 g, 40.0 mmol, 2.4 equiv.) in THF (200 mL) at room temperature for 2 h, then quenched with water (600 mL). The precipitate was filtrated, washed with water, dried, and re-slurried with petroleum ether/ethyl acetate to give compound **14a**–**c**, respectively.

3-(Benzyloxy)-8-bromoquinazoline-2,4(1*H*,3*H*)-dione (**14a**). According to the above general procedure, cyclization of **11a** with triphosgene afforded crude **12a**. MS (ESI) *m*/*z*: 242.1 [M + H]^+^. Reaction of **12a** with *O*-benzylhydroxylamine gave crude **13a**. MS (ESI) *m*/*z*: 321.0 [M + H]^+^. Compound **14a** was finally obtained from crude **13a** as a white solid (4.52 g, 36% over three steps). MS (ESI) *m*/*z*: 347.1 [M + H]^+^.

3-(Benzyloxy)-7-bromoquinazoline-2,4(1*H*,3*H*)-dione (**14b**). According to the above general procedure, cyclization of **11b** with triphosgene afforded crude **12b**. MS (ESI) *m*/*z*: 242.1 [M + H]^+^. Reaction of **12b** with *O*-benzylhydroxylamine gave crude **13b**. MS (ESI) *m*/*z*: 321.0 [M + H]^+^. Compound **14b** was finally obtained from crude **13b** as a white solid (5.28 g, 42% over three steps). MS (ESI) *m*/*z*: 347.1 [M + H]^+^. ^1^H NMR (400 MHz, DMSO-*d*_6_) δ 11.78 (s, 1H), 7.88 (d, *J* = 8.5 Hz, 1H), 7.62–7.54 (m, 2H), 7.42 (d, *J* = 7.3 Hz, 4H), 7.37 (d, *J* = 1.8 Hz, 1H), 5.09 (s, 2H).

3-(Benzyloxy)-6-bromoquinazoline-2,4(1*H*,3*H*)-dione (**14c**). According to the above general procedure, cyclization of **11c** with triphosgene afforded crude **12c**. MS (ESI) *m*/*z*: 242.1 [M + H]^+^. Reaction of **12c** with *O*-benzylhydroxylamine gave crude **13c**. MS (ESI) *m*/*z*: 321.0 [M + H]^+^. Compound **14c** was finally obtained from crude **13c** as a white solid (1.1 g, 18% over three steps). MS (ESI) *m*/*z*: 347.1 [M + H]^+^.

The ^1^H NMR, and ^13^C NMR spectra of **10b**–**p** are shown in Supplementary Materials (Figures S5–S49).

#### 3.1.4. Synthesis of Compounds **18a**–**c** and **19**

A mixture of **14b** or **15** (2.88 mmol, 1.0 equiv.), bis(pinacolato)diboron (3.47 mmol, 1.2 equiv.), Pd(dppf)Cl_2_ (0.145 mmol, 0.05 equiv.), and KOAc (8.66 mmol, 3.0 equiv.) were heated at 100 °C under nitrogen atmosphere for 8 h. The suspension was filtered while hot to remove the catalyst and KOAc, and the filtrate was poured into water (175 mL). The precipitate was collected by filtration, washed with water and petroleum ether, and dried to give crude boron **16a** or **16b**. A portion of crude **16a** or **16b** (0.769 mmol, 1.0 equiv.) was added to benzyl bromide or substituted benzyl bromide (0.641 mmol, 1.2 equiv.), Pd(dppf)Cl_2_ (0.032 mmol, 0.05 equiv.), KOAc (1.28 mmol, 2.0 equiv.), and water (2 mL) in 1,4-dioxane (20 mL). The suspension was heated at 80 °C for 3 h and then filtered while hot. The filtrate was concentrated, and the residue was purified by chromatography on silica gel with 20–30% ethyl acetate in petroleum ether to afford **17a**–**c,** which was subsequently subjected to catalytic hydrogenation following the same procedure as described in the preparation of **10a** to obtain **18a**–**c**.

7-Benzyl-3-hydroxyquinazoline-2,4(1*H*,3*H*)-dione (**18a**). According to the above general procedure, **14b** was first converted to the boron intermediate **16a**. MS (ESI) *m*/*z*: 310.8 [M−H]^−^. The crude **16a** was then subjected to the Suzuki coupling reaction with benzyl bromide to afford crude **17a**. MS (ESI) *m*/*z*: 359.1 [M + H]^+^. Subsequent debenzylation of **17a** by catalytic hydrogenation gave **18a** as a white solid (11 mg, 10% over three steps). MS (ESI) *m*/*z*: 266.9 [M−H]^−^. ^1^H NMR (400 MHz, DMSO-*d*_6_) δ 11.43 (s, 1H), 10.50 (s, 1H), 7.86 (dd, *J* = 8.1, 2.3 Hz, 1H), 7.32 (dd, *J* = 10.9, 4.6 Hz, 2H), 7.23 (d, *J* = 6.3 Hz, 3H), 7.09 (d, *J* = 8.1 Hz, 1H), 7.00 (s, 1H), 4.01 (d, *J* = 4.1 Hz, 2H). ^13^C NMR (151 MHz, DMSO-*d*_6_) δ 159.09, 148.64, 148.55, 139.79, 138.36, 128.73 (2C), 128.45 (2C), 127.16, 126.18, 123.33, 114.66, 112.13, 40.83.

7-Benzyl-3-hydroxy-1-methylquinazoline-2,4(1*H*,3*H*)-dione (**18b**). According to the above general procedure, **15** was first converted to the boron intermediate **16b**. MS (ESI) *m*/*z*: 324.8 [M−H]^−^. The crude **16b** was then subjected to the Suzuki coupling reaction with benzyl bromide to afford crude **17b**. MS (ESI) *m*/*z*: 373.1 [M + H]^+^. Subsequent debenzylation of **17b** by catalytic hydrogenation gave **18b** as a white solid (30 mg, 18% over three steps). MS (ESI) *m*/*z*: 280.9 [M−H]^−^. ^1^H NMR (400 MHz, DMSO-*d*_6_) ^1^H NMR (400 MHz, DMSO-*d*_6_) δ 10.67 (s, 1H), 7.95 (d, *J* = 8.1 Hz, 1H), 7.41 (s, 1H), 7.30 (d, *J* = 3.5 Hz, 4H), 7.21 (p, *J* = 4.2 Hz, 1H), 7.15 (d, *J* = 7.9 Hz, 1H), 4.09 (s, 2H), 3.52 (s, 3H). ^13^C NMR (151 MHz, DMSO-*d*_6_) δ 158.21, 149.24, 148.95, 140.01, 139.42, 128.66 (2C), 128.40 (2C), 127.57, 126.14, 123.38, 114.51, 112.77, 41.11, 30.59.

7-(3,5-Difluorobenzyl)-3-hydroxy-1-methylquinazoline-2,4(1*H*,3*H*)-dione (**18c**). According to the above general procedure, **14b** was first converted to the boron intermediate **16a**. MS (ESI) *m*/*z*: 310.8 [M−H]^−^. The crude **16a** was then subjected to the Suzuki coupling reaction with 1-(bromomethyl)-3,5-difluorobenzene to afford crude **17c**. MS (ESI) *m*/*z*: 373.1 [M + H]^+^. Subsequent debenzylation of **17c** by catalytic hydrogenation gave **18c** as a white solid (30 mg, 18% over three steps). MS (ESI) *m*/*z*: 316.8 [M−H]^−^. ^1^H NMR (400 MHz, DMSO-*d*_6_) δ 10.69 (s, 1H), 7.98 (d, *J* = 8.0 Hz, 1H), 7.46 (s, 1H), 7.21 (d, *J* = 7.9 Hz, 1H), 7.14–7.00 (m, 3H), 4.11 (s, 2H), 3.54 (s, 3H). ^13^C NMR (151 MHz, DMSO-*d*_6_) δ 162.22 (dd, *J* = 246.0, 13.3 Hz, 2C), 158.18, 149.23, 147.52, 144.63, 139.51, 127.71, 123.32, 114.75, 113.07, 111.88 (d, *J* = 5.0 Hz), 111.75 (d, *J* = 4.9 Hz), 101.69 (t, *J* = 25.7 Hz), 40.41, 30.65.

7-Bromo-3-hydroxyquinazoline-2,4(1*H*,3*H*)-dione (**19**). To compound **14b** (200 mg, 0.576 mmol, 1.0 equiv.) was added 48% hydrobromic acid (1.5 mL) and glacial acetic acid (1.5 mL). The solution was refluxed for 2 h. The reaction mixture was cooled to 0 °C under an ice bath and neutralized with 1 mol/L sodium hydroxide aqueous solution. The precipitate was collected by filtration, washed with water, dried, and recrystallized with methanol to obtain **19** as a pale yellow solid (120 mg, 81%). MS (ESI) *m*/*z*: 255.1 [M−H]^−^. ^1^H NMR (400 MHz, DMSO-*d*_6_) δ 11.63 (s, 1H), 10.65 (s, 1H), 7.86 (s, 1H), 7.39 (d, *J* = 13.7 Hz, 2H). ^13^C NMR (151 MHz, DMSO-*d*_6_) δ 158.78, 148.45, 139.33, 129.04, 127.84, 125.40, 117.51, 113.36.

The ^1^H NMR, ^13^C NMR, and representative ESI-MS spectra of **18a**–**c** and **19** are shown in Supplementary Materials (Figures S50–S63).

#### 3.1.5. Synthesis of Compounds **21a**–**x**

The brominated compound **14a**, **14b**, or **14c** (0.576 mmol, 1.0 equiv.), the corresponding arylboronic acid (0.864 mmol, 1.5 equiv.), Pd(PPh_3_)_4_ (0.029 mmol, 0.05 equiv.), K_2_CO_3_ (2.88 mmol, 5.0 equiv.), and water (1.5 mL) were suspended in 1,4-dioxane (15 mL). The mixture was heated at 100 °C under nitrogen for 12 h until the complete conversion of **14a**–**c** and then filtrated while hot. The filtrate was poured into water (15 mL), and the precipitate was collected by filtration, washed with water, dried, and re-slurried with methanol or ethyl acetate (5 mL) to give the crude solid **20a**–**x**. Debenzylation reactions of **20a**–**x** were subsequently performed using four different conditions to obtain **21a**–**x**, respectively. For **20a**–**i**, **20m**–**q**, and **20t**–**x**, the same method of catalytic hydrogenation as described in the preparation of **10f**–**q** was used to give **21a**–**i**, **21m**–**q**, and **20t**–**x**, respectively. For **20j**–**l**, the same procedure of debenzylation in the mixture of hydrobromic acid/glacial acetic acid as described in the preparation of **10b**–**e** was conducted to afford **21j**–**l**, respectively. **21r** or **21s** was obtained from the debenzylation of **20r** or **20s** using trifluoroacetate (TFA) or TiCl_4_, respectively.

3-Hydroxy-6-phenylquinazoline-2,4(1*H*,3*H*)-dione (**21a**). According to the above general procedure, **14a** was first subjected to the Suzuki coupling reaction with phenylboronic acid to afford crude **20a**. MS (ESI) *m*/*z*: 343.3 [M−H]^−^. Subsequent debenzylation of **20a** by catalytic hydrogenation gave **21a** as a white solid (28 mg, 30% over two steps). MS (ESI) *m*/*z*: 253.2 [M−H]^−^. ^1^H NMR (400 MHz, DMSO-*d*_6_) δ 11.64 (s, 1H), 10.63 (s, 1H), 8.15 (s, 1H), 8.00 (d, *J* = 6.4 Hz, 1H), 7.69 (d, *J* = 5.4 Hz, 2H), 7.48 (d, *J* = 6.0 Hz, 2H), 7.38 (t, *J* = 6.8 Hz, 1H), 7.30 (d, *J* = 6.9 Hz, 1H). ^13^C NMR (151 MHz, DMSO-*d*_6_) δ 159.24, 148.51, 138.47, 137.53, 134.31, 133.04, 128.95 (2C), 127.44, 126.25 (2C), 124.23, 115.97, 114.43.

6-(4-Fluorophenyl)-3-hydroxyquinazoline-2,4(1*H*,3*H*)-dione (**21b**). According to the above general procedure, **14a** was first subjected to the Suzuki coupling reaction with (4-fluorophenyl)boronic acid to afford crude **20b**. MS (ESI) *m*/*z*: 361.1 [M−H]^−^. Subsequent debenzylation of **20b** by catalytic hydrogenation gave **21b** as a white solid (38.9 mg, 29% over two steps). MS (ESI) *m*/*z*: 271.1 [M−H]^−^. ^1^H NMR (400 MHz, DMSO-*d*_6_) δ 11.64 (s, 1H), 10.63 (s, 1H), 8.13 (s, 1H), 8.01–7.90 (m, 1H), 7.74 (dd, *J* = 7.7, 4.3 Hz, 2H), 7.30 (t, *J* = 6.8 Hz, 3H). ^13^C NMR (151 MHz, DMSO-*d*_6_) δ 161.75 (d, *J* = 244.8 Hz), 159.19, 148.49, 137.49, 134.98, 133.32, 132.98, 128.35, 128.30, 124.22, 115.97, 115.79, 115.65, 114.42.

3-Hydroxy-6-(4-methoxyphenyl)quinazoline-2,4(1*H*,3*H*)-dione (**21c**). According to the above general procedure, **14a** was first subjected to the Suzuki coupling reaction with (4-methoxyphenyl)boronic acid to afford crude **20c**. MS (ESI) *m*/*z*: 373.1 [M−H]^−^. Subsequent debenzylation of **20c** by catalytic hydrogenation gave **21c** as a white solid (25 mg, 18% over two steps). MS (ESI) *m*/*z*: 283.2 [M−H]^−^. ^1^H NMR (400 MHz, DMSO-*d*_6_) δ 11.58 (s, 1H), 10.61 (s, 1H), 8.10 (s, 1H), 7.95 (d, *J* = 8.6 Hz, 1H), 7.64 (d, *J* = 8.4 Hz, 2H), 7.27 (d, *J* = 8.6 Hz, 1H), 7.04 (d, *J* = 8.3 Hz, 2H), 3.81 (s, 3H). ^13^C NMR (151 MHz, DMSO-*d*_6_) δ 159.27, 158.83, 148.49, 136.96, 134.10, 132.62, 130.83, 127.39 (2C), 123.47, 115.88, 114.39, 114.35 (2C), 55.03.

3-Hydroxy-6-(3-nitrophenyl)quinazoline-2,4(1*H*,3*H*)-dione (**21d**). According to the above general procedure, **14a** was first subjected to the Suzuki coupling reaction with (3-nitrophenyl)boronic acid to afford crude **20d**. MS (ESI) *m*/*z*: 388.1 [M−H]^−^. Subsequent debenzylation of **20d** by catalytic hydrogenation gave **21d** as a white solid (25 mg, 29% over two steps). MS (ESI) *m*/*z*: 298.2 [M−H]^−^. ^1^H NMR (400 MHz, DMSO-*d*_6_) δ 11.72 (s, 1H), 10.68 (s, 1H), 8.49–8.42 (m, 1H), 8.30–8.16 (m, 3H), 8.12 (dd, *J* = 8.7, 2.3 Hz, 1H), 7.77 (t, *J* = 8.0 Hz, 1H), 7.33 (d, *J* = 8.5 Hz, 1H). ^13^C NMR (151 MHz, DMSO-*d*_6_) δ 159.15, 148.54, 148.38, 140.12, 138.39, 133.26, 132.91, 131.92, 130.53, 125.05, 122.13, 120.76, 116.26, 114.64.

3-Hydroxy-7-phenylquinazoline-2,4(1*H*,3*H*)-dione (**21e**). According to the above general procedure, **14b** was first subjected to the Suzuki coupling reaction with phenylboronic acid to afford crude **20e**. MS (ESI) *m*/*z*: 343.1 [M−H]^−^. Subsequent debenzylation of **20e** by catalytic hydrogenation gave **21e** as an off-white solid (42 mg, 42% over two steps). MS (ESI) *m*/*z*: 253.2 [M−H]^−^. ^1^H NMR (400 MHz, DMSO-*d*_6_) δ 11.58 (s, 1H), 10.63 (s, 1H), 8.02 (d, *J* = 8.2 Hz, 1H), 7.68 (d, *J* = 7.6 Hz, 2H), 7.53 (d, *J* = 7.6 Hz, 3H), 7.48 (d, *J* = 6.7 Hz, 1H), 7.41 (d, *J* = 6.2 Hz, 1H). ^13^C NMR (151 MHz, DMSO-*d*_6_) δ 159.14, 148.77, 146.17, 138.75, 138.55, 129.14, 128.69, 127.76, 126.90, 121.23, 113.07, 112.83.

7-(4-Fluorophenyl)-3-hydroxyquinazoline-2,4(1*H*,3*H*)-dione (**21f**). According to the above general procedure, **14b** was first subjected to the Suzuki coupling reaction with (4-fluorophenyl)boronic acid to afford crude **20f**. MS (ESI) *m*/*z*: 361.1 [M−H]^−^. Subsequent debenzylation of **20f** by catalytic hydrogenation gave **21f** as a white solid (20 mg, 15% over two steps). MS (ESI) *m*/*z*: 271.1 [M−H]^−^. ^1^H NMR (400 MHz, DMSO-*d*_6_) δ 11.61 (s, 1H), 10.61 (s, 1H), 8.01 (d, *J* = 7.6 Hz, 1H), 7.73 (dd, *J* = 8.6, 5.0 Hz, 2H), 7.50 (d, *J* = 8.3 Hz, 1H), 7.36 (d, *J* = 10.6 Hz, 3H). ^13^C NMR (151 MHz, DMSO-*d*_6_) δ 162.36 (d, *J* = 246.2 Hz), 158.99, 148.63, 144.96, 138.62, 134.91, 128.97, 128.92, 127.67, 121.06, 115.98, 115.84, 112.92, 112.65.

7-(2-Aminophenyl)-3-hydroxyquinazoline-2,4(1*H*,3*H*)-dione (**21g**). According to the above general procedure, **14b** was first subjected to the Suzuki coupling reaction with (2-aminophenyl)boronic acid to afford crude **20g**. MS (ESI) *m*/*z*: 360.1 [M + H]^+^. Subsequent debenzylation of **20g** by catalytic hydrogenation gave **21g** as a white solid (34.5 mg, 37% over two steps). MS (ESI) *m*/*z*: 268.1 [M−H]^−^. ^1^H NMR (400 MHz, DMSO-*d*_6_) δ 11.50 (s, 1H), 10.65 (s, 1H), 7.98 (d, *J* = 8.6 Hz, 1H), 7.26 (d, *J* = 5.8 Hz, 2H), 7.09 (t, *J* = 7.0 Hz, 1H), 7.01 (d, *J* = 7.7 Hz, 1H), 6.77 (d, *J* = 8.2 Hz, 1H), 6.65 (t, *J* = 7.3 Hz, 1H), 4.97 (s, 2H). ^13^C NMR (151 MHz, DMSO-*d*_6_) δ 159.18, 148.77, 146.12, 145.13, 138.57, 129.66, 129.03, 127.41, 123.82, 123.22, 116.56, 115.41, 114.99, 112.44.

3-Hydroxy-7-(4-(trifluoromethyl)phenyl)quinazoline-2,4(1*H*,3*H*)-dione (**21h**). According to the above general procedure, **14b** was first subjected to the Suzuki coupling reaction with (4-(trifluoromethyl)phenyl)boronic acid to afford crude **20h**. MS (ESI) *m*/*z*: 413.1 [M + H]^+^. Subsequent debenzylation of **20h** by catalytic hydrogenation gave **21h** as a white solid (21 mg, 12% over two steps). MS (ESI) *m*/*z*: 321.1 [M−H]^−^. ^1^H NMR (400 MHz, DMSO-*d*_6_) δ 11.62 (s, 1H), 10.67 (s, 1H), 8.05 (dt, *J* = 10.8, 5.2 Hz, 1H), 7.88 (d, *J* = 8.3 Hz, 4H), 7.57 (d, *J* = 8.7 Hz, 1H), 7.44 (d, *J* = 10.0 Hz, 1H). ^13^C NMR (151 MHz, DMSO-*d*_6_) δ 158.97, 148.65, 144.41, 142.52, 138.72, 128.78 (d, *J* = 31.9 Hz), 127.90, 127.76, 125.94 (d, *J* = 3.9 Hz), 124.03 (d, *J* = 271.8 Hz), 121.34, 113.75, 113.33.

3-Hydroxy-7-(4-methoxyphenyl)quinazoline-2,4(1*H*,3*H*)-dione (**21i**). According to the above general procedure, **14b** was first subjected to the Suzuki coupling reaction with (4-methoxyphenyl)boronic acid to afford crude **20i**. MS (ESI) *m*/*z*: 373.1 [M−H]^−^. Subsequent debenzylation of **20i** by catalytic hydrogenation gave **21i** as a white solid (14.2 mg, 17% over two steps). MS (ESI) *m*/*z*: 283.2 [M−H]^−^. ^1^H NMR (400 MHz, DMSO-*d*_6_) δ 11.56 (s, 1H), 10.57 (s, 1H), 8.07–7.89 (m, 1H), 7.69–7.60 (m, 2H), 7.54–7.45 (m, 1H), 7.36 (d, *J* = 2.1 Hz, 1H), 7.14–7.05 (m, 2H), 3.82 (s, 3H). ^13^C NMR (151 MHz, DMSO-*d*_6_) δ 159.82, 159.16, 148.78, 145.80, 138.77, 130.66, 128.11 (2C), 127.68, 120.74, 114.57 (2C), 112.43, 111.96, 55.19.

7-(3-Aminophenyl)-3-hydroxyquinazoline-2,4(1*H*,3*H*)-dione (**21j**). According to the above general procedure, **14b** was first subjected to the Suzuki coupling reaction with (3-aminophenyl)boronic acid to afford crude **20j**. MS (ESI) *m*/*z*: 358.1 [M−H]^−^. Subsequent debenzylation of **20j** in the mixture of hydrobromic acid/glacial acetic acid gave **21j** as a white solid (32.4 mg, 42% over two steps). MS (ESI) *m*/*z*: 268.2 [M−H]^−^. ^1^H NMR (400 MHz, DMSO-*d*_6_) δ 11.59 (s, 1H), 10.58 (s, 1H), 7.98 (d, *J* = 7.5 Hz, 1H), 7.41 (d, *J* = 8.2 Hz, 1H), 7.33 (s, 1H), 7.16 (t, *J* = 7.0 Hz, 1H), 6.86 (s, 1H), 6.79 (d, *J* = 7.4 Hz, 1H), 6.66 (d, *J* = 7.8 Hz, 1H), 5.42 (s, 2H). ^13^C NMR (151 MHz, DMSO-*d*_6_) δ 159.11, 148.85, 148.73, 147.08, 139.23, 138.59, 129.56, 127.53, 121.00, 114.50, 114.34, 112.76, 112.43, 112.08.

3-Hydroxy-7-(3-nitrophenyl)quinazoline-2,4(1*H*,3*H*)-dione (**21k**). According to the above general procedure, **14b** was first subjected to the Suzuki coupling reaction with (3-nitrophenyl)boronic acid to afford crude **20k**. MS (ESI) *m*/*z*: 388.1 [M−H]^−^. Subsequent debenzylation of **20k** in the mixture of hydrobromic acid/glacial acetic acid gave **21k** as a white solid (16 mg, 13% over two steps). MS (ESI) *m*/*z*: 298.1 [M−H]^−^. ^1^H NMR (400 MHz, DMSO-*d*_6_) δ 11.63 (s, 1H), 10.65 (s, 1H), 8.45 (s, 1H), 8.31 (s, 1H), 8.16 (s, 1H), 8.07 (d, *J* = 8.1 Hz, 1H), 7.84 (s, 1H), 7.64 (d, *J* = 7.6 Hz, 1H), 7.50 (s, 1H). ^13^C NMR (151 MHz, DMSO-*d*_6_) δ 159.00, 148.67, 148.33, 143.60, 140.06, 138.81, 133.44, 130.78, 128.07, 123.31, 121.41, 121.35, 113.90, 113.37.

7-(3-Chloro-4-fluorophenyl)-3-hydroxyquinazoline-2,4(1*H*,3*H*)-dione (**21l**). According to the above general procedure, **14b** was first subjected to the Suzuki coupling reaction with (3-chloro-4-fluorophenyl)boronic acid to afford crude **20l**. MS (ESI) *m*/*z*: 395.0 [M−H]^−^. Subsequent debenzylation of **20l** in the mixture of hydrobromic acid/glacial acetic acid gave **21l** as a white solid (34 mg, 42% over two steps). MS (ESI) *m*/*z*: 306.8 [M + H]^+^. ^1^H NMR (400 MHz, DMSO-*d*_6_) δ 8.00 (s, 1H), 7.90 (s, 1H), 7.69 (s, 1H), 7.56 (d, *J* = 18.8 Hz, 2H), 7.37 (s, 1H). ^13^C NMR (151 MHz, DMSO-*d*_6_) δ 158.97, 157.37 (d, *J* = 248.7 Hz), 148.68, 143.61, 138.68, 136.40, 128.97, 128.07–126.56 (m), 121.21, 120.19 (d, *J* = 17.7 Hz), 117.59, 117.45, 113.40, 113.10.

7-(3,5-Difluorophenyl)-3-hydroxyquinazoline-2,4(1*H*,3*H*)-dione (**21m**). According to the above general procedure, **14b** was first subjected to the Suzuki coupling reaction with (3,5-difluorophenyl)boronic acid to afford crude **20m**. MS (ESI) *m*/*z*: 379.1 [M−H]^−^. Subsequent debenzylation of **20m** by catalytic hydrogenation gave **21m** as a white solid (30 mg, 48% over two steps). MS (ESI) *m*/*z*: 290.8 [M + H]^+^. ^1^H NMR (400 MHz, DMSO-*d*_6_) δ 11.58 (s, 1H), 10.67 (s, 1H), 8.02 (d, *J* = 8.3 Hz, 1H), 7.61–7.52 (m, 1H), 7.48–7.31 (m, 4H). ^13^C NMR (151 MHz, DMSO-*d*_6_) δ 162.67 (dd, *J* = 246.4, 13.4 Hz, 2C), 158.91, 148.62, 143.43, 142.17, 138.62, 127.78, 121.28, 113.88, 113.34, 110.30 (d, *J* = 5.8 Hz), 110.17 (d, *J* = 5.7 Hz), 103.91 (t, *J* = 25.8 Hz).

7-(2,4-Difluorophenyl)-3-hydroxyquinazoline-2,4(1*H*,3*H*)-dione (**21n**). According to the above general procedure, **14b** was first subjected to the Suzuki coupling reaction with (2,4-difluorophenyl)boronic acid to afford crude **20n**. MS (ESI) *m*/*z*: 379.1 [M−H]^−^. Subsequent debenzylation of **20n** by catalytic hydrogenation gave **21n** as a white solid (39.5 mg, 62% over two steps). MS (ESI) *m*/*z*: 290.8 [M + H]^+^. ^1^H NMR (400 MHz, DMSO-*d*_6_) δ 11.62 (s, 1H), 10.63 (s, 1H), 8.03 (d, *J* = 7.8 Hz, 1H), 7.64 (q, *J* = 8.2 Hz, 1H), 7.44 (t, *J* = 10.3 Hz, 1H), 7.38 (d, *J* = 8.3 Hz, 1H), 7.33 (s, 1H), 7.25 (t, *J* = 8.8 Hz, 1H). ^13^C NMR (151 MHz, DMSO-*d*_6_) δ 163.18–161.23 (m), 158.99 (dd, *J* = 249.7, 12.7 Hz), 158.96, 148.64, 140.04, 138.33, 134.90–130.51 (m), 127.40, 123.26 (d, *J* = 12.9 Hz), 122.90, 115.08, 113.37, 112.31 (d, *J* = 21.2 Hz), 104.69 (t, *J* = 26.4 Hz).

7-(3-Fluoro-4-methoxyphenyl)-3-hydroxyquinazoline-2,4(1*H*,3*H*)-dione (**21o**). According to the above general procedure, **14b** was first subjected to the Suzuki coupling reaction with (3-fluoro-4-methoxyphenyl)boronic acid to afford crude **20o**. MS (ESI) *m*/*z*: 391.1 [M−H]^−^. Subsequent debenzylation of **20o** by catalytic hydrogenation gave **21o** as a white solid (20.6 mg, 32% over two steps). MS (ESI) *m*/*z*: 302.8 [M + H]^+^. ^1^H NMR (400 MHz, DMSO-*d*_6_) δ 11.56 (s, 1H), 10.60 (s, 1H), 7.98 (d, *J* = 8.4 Hz, 1H), 7.63–7.54 (m, 1H), 7.54–7.46 (m, 2H), 7.40–7.35 (m, 1H), 7.33 (t, *J* = 8.4 Hz, 1H), 3.91 (s, 3H). ^13^C NMR (151 MHz, DMSO-*d*_6_) δ 159.02, 151.56 (d, *J* = 244.3 Hz), 148.68, 147.54 (d, *J* = 10.5 Hz), 144.51, 138.67, 131.16 (d, *J* = 6.2 Hz), 127.64, 123.19, 120.79, 114.26 (d, *J* = 6.6 Hz), 114.15, 112.79, 112.25, 55.97.

7-(5-Fluoro-2-methylphenyl)-3-hydroxyquinazoline-2,4(1*H*,3*H*)-dione (**21p**). According to the above general procedure, **14b** was first subjected to the Suzuki coupling reaction with (5-fluoro-2-methylphenyl)boronic acid to afford crude **20p**. MS (ESI) *m*/*z*: 375.1 [M−H]^−^. Subsequent debenzylation of **20p** by catalytic hydrogenation gave **21p** as a white solid (22.6 mg, 39% over two steps). MS (ESI) *m*/*z*: 286.8 [M + H]^+^. ^1^H NMR (400 MHz, DMSO-*d*_6_) δ 11.60 (s, 1H), 10.62 (s, 1H), 8.00 (dd, *J* = 8.1, 2.4 Hz, 1H), 7.38 (t, *J* = 7.0 Hz, 1H), 7.26–7.14 (m, 2H), 7.10 (d, *J* = 12.7 Hz, 2H), 2.19 (s, 3H). ^13^C NMR (151 MHz, DMSO-*d*_6_) δ 160.20 (d, *J* = 242.5 Hz), 159.03, 148.65, 146.07, 141.31 (d, *J* = 7.7 Hz), 138.15, 132.21 (d, *J* = 7.9 Hz), 130.66, 127.04, 123.28, 115.51 (d, *J* = 21.7 Hz), 115.24, 114.68 (d, *J* = 20.3 Hz), 113.03, 19.05.

3-Hydroxy-7-(3,4,5-trifluorophenyl)quinazoline-2,4(1*H*,3*H*)-dione (**21q**). According to the above general procedure, **14b** was first subjected to the Suzuki coupling reaction with (3,4,5-trifluorophenyl)boronic acid to afford crude **20q**. MS (ESI) *m*/*z*: 397.1 [M−H]^−^. Subsequent debenzylation of **20q** by catalytic hydrogenation gave **21q** as a white solid (30.5 mg, 37% over two steps). MS (ESI) *m*/*z*: 307.0 [M−H]^−^. ^1^H NMR (400 MHz, DMSO-*d*_6_) δ 11.64 (s, 1H), 10.64 (s, 1H), 8.06–7.97 (m, 1H), 7.68 (td, *J* = 6.9, 2.8 Hz, 2H), 7.54 (dd, *J* = 8.4, 1.9 Hz, 1H), 7.36 (d, *J* = 1.9 Hz, 1H). ^13^C NMR (151 MHz, DMSO-*d*_6_) δ 158.89, 150.47 (d, *J* = 246.6 Hz), 148.61, 142.81, 138.90 (d, *J* = 251.3 Hz), 138.58, 135.37, 127.76, 121.25, 113.77, 113.31, 111.86 (d, *J* = 4.6 Hz), 111.75 (d, *J* = 4.4 Hz).

3-Hydroxy-7-(pyridin-3-yl)quinazoline-2,4(1*H*,3*H*)-dione (**21r**). According to the above general procedure, **14b** was first subjected to the Suzuki coupling reaction with pyridin-3-ylboronic acid to afford crude **20r**. MS (ESI) *m*/*z*: 344.1 [M−H]^−^. To **20r** (90 mg, 0.261 mmol, 1.0 equiv.) was added TFA (3 mL) and the suspension was refluxed at 80 °C for 12 h. The reaction mixture was evaporated to dryness and diethyl ether (5 mL) was added. The suspension was well stirred for 15 min and then filtrated. The filtrate was discarded and the residue was washed with diethyl ether (20 mL), dried, and recrystallized with methanol to give the trifluoroacetate **21r** as a white solid (20 mg, 10% over two steps). MS (ESI) *m*/*z*: 254.1 [M−H]^−^. ^1^H NMR (400 MHz, DMSO-*d*_6_) δ 11.70 (s, 1H), 8.98 (d, *J* = 7.9 Hz, 1H), 8.74 (d, *J* = 6.0 Hz, 1H), 8.28 (d, *J* = 9.2 Hz, 1H), 8.07 (d, *J* = 8.6 Hz, 1H), 7.70 (d, *J* = 6.7 Hz, 1H), 7.60 (d, *J* = 8.6 Hz, 1H), 7.45 (d, *J* = 7.9 Hz, 1H). ^13^C NMR (151 MHz, DMSO-*d*_6_) δ 163.70, 153.37, 152.43, 150.72 (d, *J* = 33.6 Hz), 146.95, 143.48, 141.35, 139.63, 132.70, 129.49, 126.06, 118.55, 118.06 (d, *J* = 4.7 Hz).

7-(Furan-2-yl)-3-hydroxyquinazoline-2,4(1*H*,3*H*)-dione (**21s**). According to the above general procedure, **14b** was first subjected to the Suzuki coupling reaction with furan-2-ylboronic acid to afford crude **20s**. MS (ESI) *m*/*z*: 333.1 [M−H]^−^. To a suspension of **20s** (100 mg, 0.299 mmol, 1.0 equiv.) in anhydrous DCM (5 mL) was added a solution of TiCl_4_ (75 μL) in anhydrous DCM (750 μL) dropwise at 0 °C, and the mixture was stirred at 0 °C for 1 h. 1 mol/L potassium sodium tartrate aqueous solution (3 mL) was subsequently added, and the mixture was stirred for 30 min at room temperature. After completion of the reaction, DCM was evaporated, and water (10 mL) was added. The resulting suspension was extracted with ethyl acetate (4 × 10 mL). The combined organic layer was dried by Na_2_SO_4_, filtrated, concentrated, and recrystallized in petroleum ether/ethyl acetate to obtain **21s** as a pale yellow solid (9.7 mg, 10% over two steps). MS (ESI) *m*/*z*: 243.1 [M−H]^−^. ^1^H NMR (400 MHz, DMSO-*d*_6_) δ 11.60 (s, 1H), 10.57 (s, 1H), 7.95 (d, *J* = 8.0 Hz, 1H), 7.88 (s, 1H), 7.57 (d, *J* = 8.2 Hz, 1H), 7.46 (s, 1H), 7.16 (d, *J* = 3.8 Hz, 1H), 6.68 (d, *J* = 3.8 Hz, 1H). ^13^C NMR (151 MHz, DMSO-*d*_6_) δ 158.98, 151.32, 148.73, 144.57, 138.92, 135.39, 127.79, 117.95, 112.62, 112.51, 109.12, 108.63.

7-(Benzofuran-2-yl)-3-hydroxyquinazoline-2,4(1*H*,3*H*)-dione (**21t**). According to the above general procedure, **14b** was first subjected to the Suzuki coupling reaction with benzofuran-2-ylboronic acid to afford crude **20t**. MS (ESI) *m*/*z*: 383.1 [M−H]^−^. Subsequent debenzylation of **20t** by catalytic hydrogenation gave **21t** as a white solid (21 mg, 21% over two steps). MS (ESI) *m*/*z*: 293.1 [M−H]^−^. ^1^H NMR (400 MHz, DMSO-*d*_6_) δ 11.67 (s, 1H), 10.64 (s, 1H), 8.03 (d, *J* = 8.4 Hz, 1H), 7.79 (d, *J* = 8.1 Hz, 1H), 7.73 (d, *J* = 7.3 Hz, 1H), 7.69 (t, *J* = 3.6 Hz, 2H), 7.66–7.62 (m, 1H), 7.40 (t, *J* = 7.3 Hz, 1H), 7.32 (t, *J* = 7.3 Hz, 1H). ^13^C NMR (151 MHz, DMSO-*d*_6_) δ 158.93, 154.48, 153.28, 148.71, 138.91, 134.94, 128.34, 127.87, 125.62, 123.51, 121.72, 118.96, 113.72, 111.25, 110.17, 105.10.

3-Hydroxy-8-phenylquinazoline-2,4(1*H*,3*H*)-dione (**21u**). According to the above general procedure, **14c** was first subjected to the Suzuki coupling reaction with phenylboronic acid to afford crude **20u**. MS (ESI) *m*/*z*: 342.8 [M−H]^−^. Subsequent debenzylation of **20u** by catalytic hydrogenation gave **21u** as a white solid (17.6 mg, 23% over two steps). MS (ESI) *m*/*z*: 253.0 [M−H]^−^. ^1^H NMR (400 MHz, DMSO-*d*_6_) δ 10.64 (s, 1H), 10.16 (s, 1H), 8.01 (dd, *J* = 7.4, 2.2 Hz, 1H), 7.57–7.47 (m, 3H), 7.47–7.38 (m, 3H), 7.31 (t, *J* = 7.8 Hz, 1H). ^13^C NMR (151 MHz, DMSO-*d*_6_) δ 159.08, 148.36, 136.10, 135.72, 135.28, 129.07 (2C), 128.89, 128.81 (2C), 127.93, 126.41, 122.59, 114.93.

8-(4-Fluorophenyl)-3-hydroxyquinazoline-2,4(1*H*,3*H*)-dione (**21v**). According to the above general procedure, **14c** was first subjected to the Suzuki coupling reaction with (4-fluorophenyl)boronic acid to afford crude **20v**. MS (ESI) *m*/*z*: 361.1 [M−H]^−^. Subsequent debenzylation of **20v** by catalytic hydrogenation gave **21v** as a white solid (18 mg, 32% over two steps). MS (ESI) *m*/*z*: 270.9 [M−H]^−^. ^1^H NMR (400 MHz, DMSO-*d*_6_) δ 10.62 (d, *J* = 8.1 Hz, 1H), 10.34 (d, *J* = 6.9 Hz, 1H), 8.00 (d, *J* = 8.1 Hz, 1H), 7.55–7.48 (m, 1H), 7.45 (ddd, *J* = 8.4, 5.7, 2.3 Hz, 2H), 7.36–7.21 (m, 3H). ^13^C NMR (151 MHz, DMSO-*d*_6_) δ 162.06 (d, *J* = 244.0 Hz), 159.04, 148.45, 135.73, 135.57, 132.52, 131.36, 131.31, 128.01, 126.52, 122.48, 115.69, 115.55, 114.92.

3-Hydroxy-8-(4-methoxyphenyl)quinazoline-2,4(1*H*,3*H*)-dione (**21w**). According to the above general procedure, **14c** was first subjected to the Suzuki coupling reaction with (4-methoxyphenyl)boronic acid to afford crude **20w**. MS (ESI) *m*/*z*: 373.1 [M−H]^−^. Subsequent debenzylation of **20w** by catalytic hydrogenation gave **21w** as a white solid (45.1 mg, 61% over two steps). MS (ESI) *m*/*z*: 282.9 [M−H]^−^. ^1^H NMR (400 MHz, DMSO-*d*_6_) δ 10.63 (s, 1H), 10.07 (s, 1H), 8.07–7.92 (m, 1H), 7.57–7.47 (m, 1H), 7.35 (d, *J* = 8.8 Hz, 2H), 7.29 (t, *J* = 7.8 Hz, 1H), 7.13–7.03 (m, 2H), 3.82 (s, 3H). ^13^C NMR (151 MHz, DMSO-*d*_6_) δ 159.12, 159.06, 148.33, 135.60, 135.41, 130.30 (2C), 128.70, 128.23, 126.00, 122.55, 114.84, 114.30 (2C), 55.03.

3-Hydroxy-8-(4-(trifluoromethyl)phenyl)quinazoline-2,4(1*H*,3*H*)-dione (**21x**). According to the above general procedure, **14c** was first subjected to the Suzuki coupling reaction with (4-(trifluoromethyl)phenyl)boronic acid to afford crude **20x**. MS (ESI) *m*/*z*: 411.1 [M−H]^−^. Subsequent debenzylation of **20x** by catalytic hydrogenation gave **21x** as a white solid (28.5 mg, 20% over two steps). MS (ESI) *m*/*z*: 320.8 [M−H]^−^. ^1^H NMR (400 MHz, DMSO-*d*_6_) δ 10.65 (s, 1H), 10.58 (s, 1H), 8.13–7.98 (m, 1H), 7.84 (d, *J* = 7.9 Hz, 2H), 7.64 (d, *J* = 8.1 Hz, 2H), 7.60–7.51 (m, 1H), 7.34 (dd, *J* = 9.4, 6.4 Hz, 1H). ^13^C NMR (151 MHz, DMSO-*d*_6_) δ 158.97, 148.53, 140.51, 135.67, 135.50, 130.16, 128.33 (d, *J* = 31.5 Hz), 127.57, 127.10, 125.59 (d, *J* = 4.1 Hz), 124.26 (d, *J* = 272.0 Hz), 122.54, 115.07.

The ^1^H NMR, ^13^C NMR, and representative ESI-MS spectra of **21a**–**x** are shown in Supplementary Materials (Figures S64–S151).

#### 3.1.6. Synthesis of Compounds **23a**–**j**

To compound **20a**, **20d**–**f**, **20i** or **20s** (0.436 mmol, 1.0 equiv.) was added K_2_CO_3_ (0.872 mmol, 2.0 equiv.), the corresponding halide (0.872 mmol, 2.0 equiv.), and DMF (4 mL). The suspension was heated at 80 °C for 2 h or stirred at room temperature for 2 h when the halide was iodomethane. After cooling, the reaction was quenched with water (10 mL), and the precipitate was collected by filtration, washed with water, and dried to give the crude product **22a**–**j**. Debenzylation reactions of **22a**–**j** were subsequently conducted using three different conditions to obtain **23a**–**j**, respectively. For **22a**, the same method of debenzylation using TFA as described in the preparation of **21r** was performed to give **23a**. For **22b** or **22c**, the same procedure of debenzylation in the mixture of hydrobromic acid/glacial acetic acid as described in the preparation of **10b**–**e** was conducted to afford **23b** or **23c**, respectively. For **22d**–**j**, the method of catalytic hydrogenation described in the preparation of **10f**–**q** was used to give **23d**–**j**, respectively.

3-Hydroxy-1-methyl-6-(3-nitrophenyl)quinazoline-2,4(1*H*,3*H*)-dione (**23a**). According to the above general procedure, **20d** was subjected to the alkylation reaction with iodomethane to afford crude **22a**. MS (ESI) *m*/*z*: 404.1 [M + H]^+^. Subsequent debenzylation of **22a** in TFA gave **23a** as a grey solid (53.4 mg, 56% over two steps). MS (ESI) *m*/*z*: 312.1 [M−H]^−^. ^1^H NMR (400 MHz, DMSO-*d*_6_) δ 10.80 (s, 1H), 8.61 (s, 1H), 8.31 (d, *J* = 8.2 Hz, 2H), 8.16 (d, *J* = 8.1 Hz, 1H), 7.90–7.80 (m, 1H), 7.76 (s, 1H), 7.71 (d, *J* = 8.0 Hz, 1H), 3.66 (s, 3H). ^13^C NMR (151 MHz, DMSO-*d*_6_) δ 158.06, 149.24, 148.30, 143.98, 140.26, 139.81, 133.95, 130.45, 128.24, 123.21, 121.87, 121.60, 114.47, 113.30, 30.80.

3-Hydroxy-1-methyl-7-phenylquinazoline-2,4(1*H*,3*H*)-dione (**23b**). According to the above general procedure, **20e** was subjected to the alkylation reaction with iodomethane to afford crude **22b**. MS (ESI) *m*/*z*: 359.1 [M + H]^+^. Subsequent debenzylation of **22b** in the mixture of hydrobromic acid/glacial acetic acid gave **23b** as a grey solid (209 mg, 73% over two steps). MS (ESI) *m*/*z*: 267.1 [M−H]^−^. ^1^H NMR (400 MHz, DMSO-*d*_6_) δ 10.74 (s, 1H), 8.12 (d, *J* = 8.1 Hz, 1H), 7.83 (d, *J* = 7.4 Hz, 2H), 7.61 (d, *J* = 7.6 Hz, 2H), 7.54 (t, *J* = 7.4 Hz, 2H), 7.47 (t, *J* = 7.2 Hz, 1H), 3.64 (s, 3H). ^13^C NMR (151 MHz, DMSO-*d*_6_) δ 158.19, 149.30, 146.46, 139.73, 138.65, 128.93 (2C), 128.63, 128.01, 127.27 (2C), 121.36, 113.65, 112.61, 30.65.

7-(4-Fluorophenyl)-3-hydroxy-1-methylquinazoline-2,4(1*H*,3*H*)-dione (**23c**). According to the above general procedure, **20f** was subjected to the alkylation reaction with iodomethane to afford crude **22c**. MS (ESI) *m*/*z*: 377.1 [M + H]^+^. Subsequent debenzylation of **22c** in the mixture of hydrobromic acid/glacial acetic acid gave **23c** as a white solid (30.8 mg, 45% over two steps). MS (ESI) *m*/*z*: 285.2 [M−H]^−^. ^1^H NMR (400 MHz, DMSO-*d*_6_) δ 10.75 (s, 1H), 8.11 (d, *J* = 8.0 Hz, 1H), 7.98–7.85 (m, 2H), 7.60 (d, *J* = 9.4 Hz, 2H), 7.37 (t, *J* = 8.4 Hz, 2H), 3.64 (s, 3H). ^13^C NMR (151 MHz, DMSO-*d*_6_) δ 163.36, 161.73, 158.21, 149.35, 145.39, 139.79, 135.15, 129.56, 129.50, 128.09, 121.34, 115.91, 115.76, 113.68, 112.64, 30.73.

3-Hydroxy-7-(4-methoxyphenyl)-1-methylquinazoline-2,4(1*H*,3*H*)-dione (**23d**). According to the above general procedure, **20i** was subjected to the alkylation reaction with iodomethane to afford crude **22d**. MS (ESI) *m*/*z*: 389.2 [M + H]^+^. Subsequent debenzylation of **22d** by catalytic hydrogenation gave **23d** as a white solid (20 mg, 19% over two steps). MS (ESI) *m*/*z*: 297.2 [M−H]^−^. ^1^H NMR (400 MHz, DMSO-*d*_6_) δ 10.72 (s, 1H), 8.08 (s, 1H), 7.81 (s, 2H), 7.58 (s, 2H), 7.09 (s, 2H), 3.83 (s, 3H), 3.64 (s, 3H). ^13^C NMR (151 MHz, DMSO-*d*_6_) δ 159.86, 158.27, 149.39, 146.15, 139.80, 130.86, 128.58 (2C), 128.00, 120.89, 114.40 (2C), 113.06, 111.80, 55.21, 39.94, 39.81, 39.68, 39.54, 39.40, 39.26, 39.12, 38.98, 30.68.

1-Ethyl-3-hydroxy-7-phenylquinazoline-2,4(1*H*,3*H*)-dione (**23e**). According to the above general procedure, **20e** was subjected to the alkylation reaction with bromoethane to afford crude **22e**. MS (ESI) *m*/*z*: 373.2 [M + H]^+^. Subsequent debenzylation of **22e** by catalytic hydrogenation gave **23e** as a white solid (6.8 mg, 8% over two steps). MS (ESI) *m*/*z*: 281.2 [M−H]^−^. ^1^H NMR (400 MHz, DMSO-*d*_6_) δ 10.76 (s, 1H), 8.14 (d, *J* = 8.7 Hz, 1H), 7.82 (d, *J* = 7.2 Hz, 2H), 7.56 (td, *J* = 30.5, 26.6, 9.5 Hz, 5H), 4.40–4.18 (m, 2H), 1.28 (dt, *J* = 11.9, 5.8 Hz, 3H). ^13^C NMR (151 MHz, DMSO-*d*_6_) δ 158.11, 148.86, 146.71, 138.68, 138.62, 128.93 (2C), 128.62, 128.40, 127.36 (2C), 121.43, 113.92, 112.13, 38.24, 12.40.

3-Hydroxy-6-phenyl-1-propylquinazoline-2,4(1*H*,3*H*)-dione (**23f**). According to the above general procedure, **20a** was subjected to the alkylation reaction with 1-bromopropane to afford crude **22f**. MS (ESI) *m*/*z*: 387.0 [M + H]^+^. Subsequent debenzylation of **22f** by catalytic hydrogenation gave **23f** as a white solid (22.3 mg, 41% over two steps). MS (ESI) *m*/*z*: 295.1 [M−H]^−^. ^1^H NMR (400 MHz, DMSO-*d*_6_) δ 10.80 (s, 1H), 8.28 (s, 1H), 8.08 (d, *J* = 8.0 Hz, 1H), 7.73 (d, *J* = 8.1 Hz, 2H), 7.62 (d, *J* = 7.2 Hz, 1H), 7.51 (t, *J* = 7.3 Hz, 2H), 7.41 (d, *J* = 7.1 Hz, 1H), 4.12 (t, *J* = 7.5 Hz, 2H), 1.69 (q, *J* = 7.6 Hz, 2H), 0.97 (t, *J* = 6.8 Hz, 3H). ^13^C NMR (151 MHz, DMSO-*d*_6_) δ 158.26, 148.91, 138.08, 137.74, 134.42, 133.08, 128.99 (2C), 127.59, 126.30 (2C), 124.87, 115.50, 115.36, 44.60, 20.14, 10.70.

3-Hydroxy-7-phenyl-1-propylquinazoline-2,4(1*H*,3*H*)-dione (**23g**). According to the above general procedure, **20e** was subjected to the alkylation reaction with 1-bromopropane to afford crude **22g**. MS (ESI) *m*/*z*: 387.2 [M + H]^+^. Subsequent debenzylation of **22g** by catalytic hydrogenation gave **23g** as a white solid (25 mg, 25% over two steps). MS (ESI) *m*/*z*: 295.1 [M−H]^−^. ^1^H NMR (400 MHz, DMSO-*d*_6_) δ 10.76 (s, 1H), 8.19–8.10 (m, 1H), 7.80 (d, *J* = 4.7 Hz, 2H), 7.66–7.57 (m, 2H), 7.57–7.51 (m, 2H), 7.52–7.43 (m, 1H), 4.23 (t, *J* = 8.0 Hz, 2H), 1.78–1.61 (m, 2H), 0.96 (t, *J* = 6.3 Hz, 3H). ^13^C NMR (151 MHz, DMSO-*d*_6_) δ 158.08, 149.18, 146.65, 138.90, 138.71, 128.96 (2C), 128.60, 128.35, 127.33 (2C), 121.48, 113.85, 112.35, 44.32, 20.16, 10.82.

1-Benzyl-3-hydroxy-6-phenylquinazoline-2,4(1*H*,3*H*)-dione (**23h**). According to the above general procedure, **20a** was subjected to the alkylation reaction with benzyl bromide to afford crude **22h**. MS (ESI) *m*/*z*: 435.0 [M + H]^+^. Subsequent debenzylation of **22h** by catalytic hydrogenation gave **23h** as a white solid (20 mg, 33% over two steps). MS (ESI) *m*/*z*: 343.1 [M−H]^−^. ^1^H NMR (400 MHz, DMSO-*d*_6_) δ 10.92 (s, 1H), 8.31 (d, *J* = 8.6 Hz, 1H), 7.99 (d, *J* = 9.1 Hz, 1H), 7.70 (d, *J* = 8.0 Hz, 2H), 7.48 (t, *J* = 7.6 Hz, 2H), 7.45–7.22 (m, 7H), 5.44 (s, 2H). ^13^C NMR (151 MHz, DMSO-*d*_6_) δ 158.41, 149.61, 138.05, 137.87, 136.00, 134.78, 133.09, 129.04 (2C), 128.66 (2C), 127.69, 127.25, 126.38 (2C), 126.32 (2C), 124.93, 115.90, 115.62, 46.40.

1-Benzyl-3-hydroxy-7-phenylquinazoline-2,4(1*H*,3*H*)-dione (**23i**). According to the above general procedure, **20e** was subjected to the alkylation reaction with benzyl bromide to afford crude **22i**. MS (ESI) *m*/*z*: 435.1 [M + H]^+^. Subsequent debenzylation of **22i** by catalytic hydrogenation gave **23i** as a white solid (26 mg, 29% over two steps). MS (ESI) *m*/*z*: 345.2 [M + H]^+^. ^1^H NMR (400 MHz, DMSO-*d*_6_) δ 10.91 (d, *J* = 20.9 Hz, 1H), 8.24–8.10 (m, 1H), 7.74–7.20 (m, 12H), 5.57 (d, *J* = 21.3 Hz, 2H). ^13^C NMR (151 MHz, DMSO-*d*_6_) δ 158.23, 149.87, 146.26, 138.95, 138.49, 136.27, 129.03 (2C), 128.72 (2C), 128.67, 128.39, 127.25, 127.07 (2C), 126.42 (2C), 121.61, 114.09, 112.96, 46.18.

*N*-(2-Chlorophenyl)-2-(7-(furan-2-yl)-3-hydroxy-2,4-dioxo-3,4-dihydroquinazolin-1(2*H*)-yl)acetamide (**23j**). According to the above general procedure, **20s** was subjected to the alkylation reaction with 2-chloro-*N*-(2-chlorophenyl)acetamide to afford crude **22j**. MS (ESI) *m*/*z*: 502.1 [M + H]^+^. Subsequent debenzylation of **22j** by catalytic hydrogenation gave **23j** as a white solid (26 mg, 13% over two steps). MS (ESI) *m*/*z*: 410.0 [M−H]^−^. ^1^H NMR (400 MHz, DMSO-*d*_6_) δ 10.89 (s, 1H), 10.10 (s, 1H), 8.11 (d, *J* = 9.0 Hz, 1H), 7.88 (s, 1H), 7.67 (d, *J* = 8.2 Hz, 1H), 7.65–7.56 (m, 2H), 7.51 (d, *J* = 7.7 Hz, 1H), 7.32 (t, *J* = 6.5 Hz, 1H), 7.27 (s, 1H), 7.24 (d, *J* = 10.8 Hz, 1H), 6.70 (s, 1H), 5.17 (s, 2H). ^13^C NMR (151 MHz, DMSO-*d*_6_) δ 165.98, 158.16, 151.37, 149.59, 139.66, 135.80, 134.22, 129.52, 128.45, 127.47, 127.22, 126.89, 126.46, 118.21, 113.40, 112.63, 112.54, 109.56, 108.37, 46.22.

The ^1^H NMR, ^13^C NMR, and representative ESI-MS spectra of **23a**–**j** are shown in Supplementary Materials (Figures S152–S185).

### 3.2. Anti-HCV Assay

HCV 1b replicon cells (Huh-7.5.1) containing luciferase reporter gene were kindly provided by Prof. Zhenghong Yuan (Key Laboratory of Medical Molecular Virology, Fudan University, Shanghai, China). The replicon cells were plated at 1.5 × 10^4^ cells per well in a 48-well plate (Corning Inc., New York, NY, USA) at 5% CO_2_/37 °C. The culture medium was Dulbecco’s Modified Eagle Medium (DMEM, pH 7.3, Shanghai XP Biomed Ltd., Shanghai, China) containing 10% fetal bovine serum (Shanghai XP Biomed Ltd., Shanghai, China), 10^5^ U/L penicillin, 0.1 g/L streptomycin, and 0.5 μg/mL blasticidin. After 12 h, the culture medium in each well was replaced with 300 μL of pre-prepared fresh culture medium containing test compound (dissolved in DMSO with specific concentration) or blank DMSO. The final concentration of DMSO in the culture medium in the plate was 0.1%. Each group was tested at least twice in parallel. Ribavirin and 2CMA were used as the positive controls. After incubating the drug-added plate at 5% CO_2_/37 °C for 72 h, the medium was removed, and each well was washed with 300 μL of phosphate buffer solution (PBS, Shanghai XP Biomed Ltd., Shanghai, China). The cells in each well were then lysed with 50 μL of 1× lysis buffer (E2820, Promega, Madison, WI, USA). Subsequently, 50 μL of 1× luciferase assay substrate (E2820, Promega, Madison, WI, USA) was mixed with 20 μL of the harvested cell lysates. The sample was immediately placed in an ultra-weak luminescence analyzer (BPCL-GP21Q, Guangzhou Weiguang Technology Co., Ltd., Guangzhou, China), and the sum of emitted photons in the first 10 s was measured, which was represented by *L*. The inhibitory rate of a compound in a given concentration on HCV replicon cells was calculated using the following equation, in which *L*_compound_ or *L*_blank_ represents the measured signal of the compound group or the blank control (DMSO), respectively:

inhibitory rate = (1−*L*_compound_/*L*_blank_) × 100% (1)

Compounds that showed good inhibitory rates at 10 μM or 25 μM were subjected to further anti-HCV EC_50_ (50% effective concentration) determination. The inhibitory rates under at least six different concentration gradients were imported into GraphPad Prism 7.0 software to calculate the anti-HCV EC_50_ values of the compounds using the nonlinear fitting method.

### 3.3. Cytotoxicity Assay

The HCV 1b replicon cells were incubated and treated with test compounds or blank DMSO in a 48-well plate using the same materials and methods as described in the experimental procedure of anti-HCV assay. The final concentration of DMSO in each well was 0.1%. After 72 h of drug treatment, the culture medium in the well plate was removed, and cells in each well were washed with 300 μL of PBS. A total of 300 μL of pre-prepared fresh culture medium containing 30 μL CCK-8 reagent (Donjindo, Kumamoto, Japan) was added to each well, and the plate was incubated at 5% CO_2_/37 °C for 2 h. The OD (optical density) value of each well at 450 nm was measured using a multi-function microplate reader (SpectraMax M5, Molecular Devices, San Jose, CA, USA). The death ratio of each well was calculated using the following equation, in which *OD*_compound_ or *OD*_blank_ represents the measured OD value of the compound group or the blank control (DMSO), respectively:

death ratio = (1−*OD*_compound_/*OD*_blank_) × 100% (2)

The cell death data under at least six different concentration gradients were imported into GraphPad Prism 7.0.0 software (GraphPad Software Inc., San Diego, CA, USA), and the CC_50_ (50% cytotoxic concentration) values were calculated using the nonlinear fitting method.

### 3.4. Thermal Shift Assay

C-terminal His-tagged NS5BΔ21 from HCV 1b subtype was expressed from pET28a HCR6 plasmid in our lab and dissolved in a Tris-HCl buffer (20 mm, pH 7.2) containing 150 mm NaCl. Then, 1 μL of corresponding concentration (1 mm, 2 mm, or 4 mm) of the test compound in DMSO or 1 μL DMSO alone was added to each well of a 96-well PCR plate (Shanghai Sangon Biotech Co., Ltd., Shanghai, China). Subsequently, 19 μL of pre-prepared PBS solution containing 10 mm MgCl_2_, 10× SYPRO^®^ orange protein gel stain (Sigma-Aldrich, St. Louis, MO, USA), and 5 μM NS5B protein was added to each well and mixed. The final concentration of DMSO in each well was 5%. The PCR plate was covered with a high-permeable film, incubated at room temperature for 10 min, and then placed on the Applied Biosystems^TM^ 7500 Real-Time PCR System (Thermo Fisher Scientific, Waltham, MA, USA). The temperature was increased to 90 °C gradually, and the change curve of fluorescence intensity versus system temperature was recorded.

### 3.5. UV-Vis spectrophotometry Assay

2 mL of methanol solution containing the components in the sample or control group (Table 4) was added to a quartz cuvette (BQ-114-2, Chongqing Xinweier Glass Co., Ltd., Chongqing, China). The absorbance at 200–400 nm was scanned by a UV-Vis spectrophotometer (Agilent Cary 60, Agilent Technologies, Santa Clara, CA, USA), and the blank methanol was used for baseline calibration. The absorbance of the control group was subtracted from that of the sample group at the corresponding wavelength to obtain the differential UV-Vis spectra, which were used to analyze the chelating property of the compound with Mg^2+^.

### 3.6. Molecular Docking

Molecular docking simulations were performed using the Schrödinger software package [54]. The protein structure was obtained from the co-crystal structure of NS5B 1b subtype in complex with UTP (PDB code: 1GX6) [9]. Except for the water molecules interacting with the metal ions in the catalytic center, all other water molecules in the co-crystal structure were deleted. The protein structure was automatically hydrogenated and optimized by limiting the heavy atoms within the range of RMSD = 0.3 Å using the OPLS3 force field. The structure of the ligand was built in the 3D Builder module with an ionization state generated by Epik and optimized under an OPLS3 force field in the LigPrep program. Finally, the ligand was docked into the catalytic center of NS5B protein using the Glide module at standard precision (SP). Among the five docking models in the output, the conformation of the ligand–protein complex with the best Glide score (the lowest value) was selected for subsequent binding mode analysis.

## 4. Conclusions

In this study, we designed and synthesized a series of 3-hydroxyquinazoline-2,4(1*H*,3*H*)-dione derivatives with metal ion chelating structures. The anti-HCV assay using the HCV replicon model showed that most compounds possessed potent HCV inhibitory activities at the cellular level. Some compounds, represented by **21h**, **21k,** and **21t**, exhibited superior activities and therapeutic indexes to ribavirin. Among them, **21t** is currently known as one of the metal ion chelators with the best anti-HCV activity (EC_50_ = 2.0 μM) at the cellular level, and no obvious cytotoxic effect at the tested concentrations (TI > 25) was observed. Thermal shift assay and molecular docking studies suggested that 3-hydroxyquinazoline-2,4(1*H*,3*H*)-diones may exert an anti-HCV effect by binding to NS5B. The UV-Vis spectrophotometry assay demonstrated that compound **21e** with the 3-OH-quinazolinedione core could chelate Mg^2+^ in the N-hydroxyl deprotonated form, supporting the design idea of inhibiting HCV by targeting the metal ions in the catalytic center of NS5B. These findings verified the feasibility of developing novel anti-HCV inhibitors based on metal ion chelating structures and provided a structural reference for further research on metal ion chelators in the field of anti-HCV.

## Figures and Tables

**Table 1:**
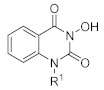
Anti-HCV assay of N-1 substituted 3-hydroxy-quinazoline-2,4(1*H*,3*H*)-diones.

Comp.	R^1^ Substituent	HCV Inhibition ^a^		EC_50_/μM ^c^	CC_50_/μM ^d^	TI ^e^
		25 μM ^b^	10 μM ^b^			
**10a**	H	36.0%	−3.0%	–	–	
**10b**	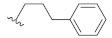	40.9%	28.6%	–	–	
**10c**	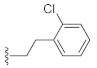	67.8%	33.4%	>12.2	12.2	
**10d**	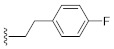	75.7%	36.4%	13.3 ± 0.9	22.2 ± 4.6	1.7
**10e**	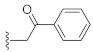	68.0%	46.1%	11.3 ± 3.0	13.1 ± 1.7	1.2
**10f**	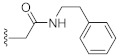	36.9%	13.3%	–	–	
**10g**	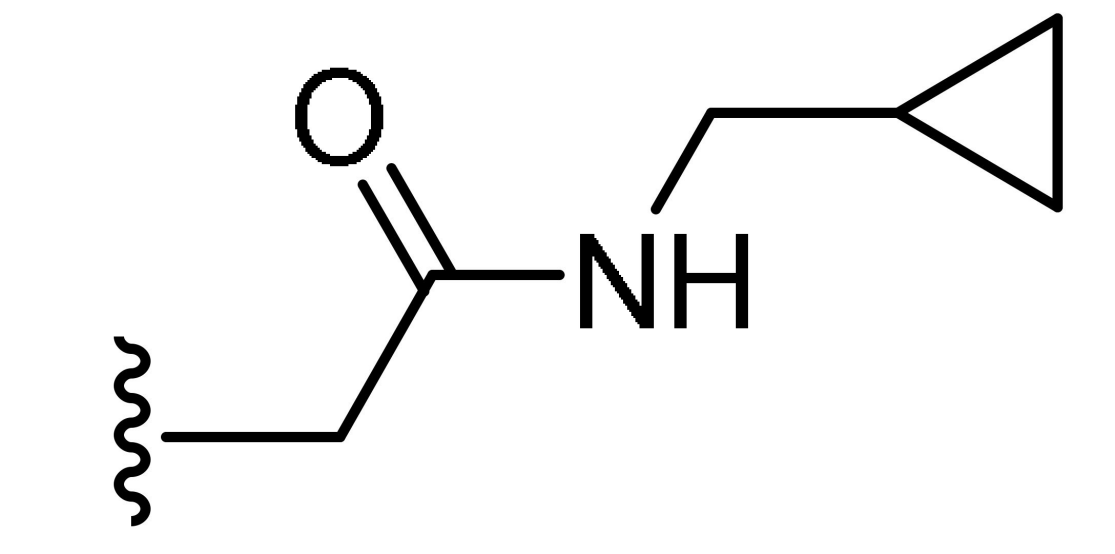	15%	10.6%	–	–	
**10h**	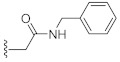	48.6%	1.3%	–	–	
**10i**	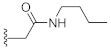	22.9%	26.5%	–	–	
**10j**	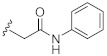	62.7%	30.1%	–	–	
**10k**	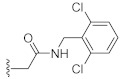	54.7%	43.7%	–	–	
**10l**	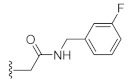	45.5%	22.4%	–	–	
**10m**	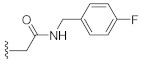	58.4%	12.7%	–	–	
**10n**	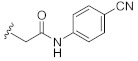	66.0%	58.0%	6.4 ± 0.3	10.6 ± 0.3	1.7
**10o**	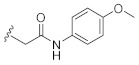	67.5%	16.9%	–	–	
**10p**	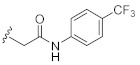	64.9%	41.4%	8.1 ± 0.1	15.3 ± 0.7	1.9
RBV		53.2%	–	20.0 ± 4.1	45.5 ± 2.6	2.3
2CMA		–	–	0.36 ± 0.03	>10	>27

^a^ The inhibitory rate was derived from the mean value of at least two independent experiments. ^b^ The inhibitory rate at the specific concentration of test compounds. ^c^ 50% effective concentration, represented as mean value ± standard deviation, generated from at least six different concentration gradients. ^d^ 50% cytotoxic concentration, generated from at least six different concentration gradients. ^e^ Therapeutic index, calculated as CC_50_/EC_50_.

**Table 2:**
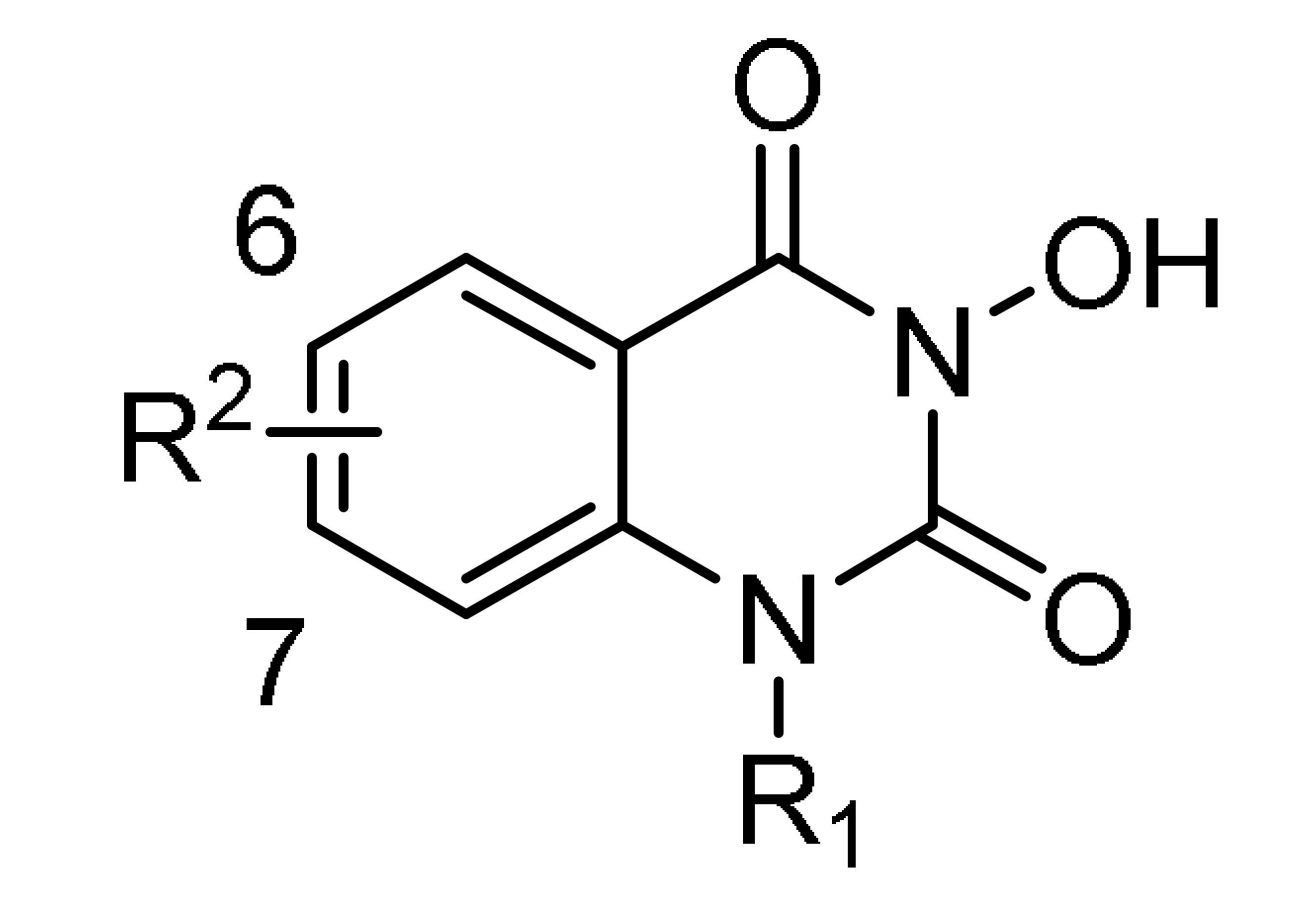
Anti-HCV assay of C-6/C-7/C-8 substituted 3-hydroxy-quinazoline-2,4(1*H*,3*H*)-diones.

Comp.	Substituent			HCV Inhibition ^a^			EC_50_ /μM ^c^	CC_50_ /μM ^d^	TI ^e^
	R^1^	R^2^		25 μM ^b^	10 μM ^b^	5 μM ^b^			
		Pos.	Group						
**10a**	H		H	36.0%	−3.0%	–	–	–	
**18a**	H	7	Bn	59.8%	28.6%	–	–	–	
**18b**	Me	7	Bn	–	23.0%	–	–	–	
**18c**	Me	7	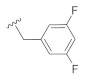	–	39.5%	–	–	–	
**19**	H	7	Br	72.0%	41.1%	23.5%	19.0 ± 1.8	37.6 ± 2.6	2.0
**21a**	H	6	Phenyl	–	65.4%	35.6%	4.3 ± 0.7	5.5 ± 1.0	1.3
**21b**	H	6	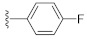	–	79.7%	47.7%	6.1 ± 0.1	8.0 ± 2.2	1.3
**21c**	H	6	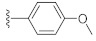	–	48.2%	11.1%	7.2 ± 0.8	14.8 ± 5.3	2.1
**21d**	H	6	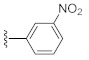	–	78.5%	71.1%	4.8 ± 0.8	6.6 ± 1.5	1.4
**21e**	H	7	Phenyl	–	78.1%	39.5%	3.2 ± 0.04	5.4 ± 0.6	1.7
**21f**	H	7	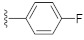	–	78.8%	73.4%	3.1 ± 0.1	4.1 ± 0.6	1.3
**21g**	H	7	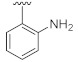	–	64.7%	24.7%	8.8 ± 1.8	16.2 ± 0.7	1.8
**21h**	H	7	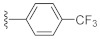	–	53.8%	5.0%	9.6 ± 2.7	>50	>5.2
**21i**	H	7	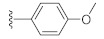	–	64.7%	–	3.4 ± 0.1	5.6 ± 0.5	1.6
**21j**	H	7	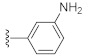	–	51.0%	16.1%	7.3 ± 1.3	13.0 ± 0.8	1.8
**21k**	H	7	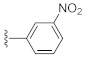	–	70.6%	55%	3.5 ± 0.3	40.9 ± 3.5	11.7
**21l**	H	7	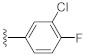	–	49.7%	25.9%	7.9 ± 1.0	40.7 ± 3.9	5.2
**21m**	H	7	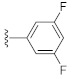	–	48.2%	–	–	–	
**21n**	H	7	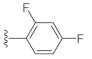	–	69.7%	–	3.9 ± 0.3	11.2 ± 1.4	2.9
**21o**	H	7	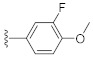	–	71.3%	–	3.9 ± 2.2	4.3 ± 0.4	1.1
**21p**	H	7	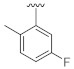	–	44.9%	–	9.9 ± 0.6	13.4 ± 5.2	1.4
**21q**	H	7	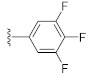	5.4%	26.8%	–	–	–	
**21r**	H	7	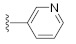	65.4%	26.9%	−5.5%	–	–	
**21s**	H	7	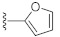	–	72.6%	56.2%	4.0 ± 0.5	16.3 ± 0.6	4.1
**21t**	H	7	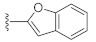	78.5%	68.3%	–	2.0 ± 0.2	>50	>25
**21u**	H	8	Phenyl	40.9%	27.5%	1.8%	–	–	
**21v**	H	8	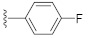	44.5%	23.5%	–	–	–	
**21w**	H	8	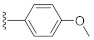	36.4%	11.5%	–	–	–	
**21x**	H	8	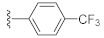	47.5%	16.4%	–	–	–	
**23a**	Me	6	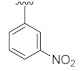	–	66.5%	–	2.3 ± 0.1	5.2 ± 0.1	2.3
**23b**	Me	7	Phenyl	–	79.5%	39.2%	3.9 ± 1.0	6.7 ± 0.3	1.7
**23c**	Me	7	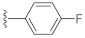	–	79.1%	40.6%	4.6 ± 0.5	8.1 ± 0.2	1.8
**23d**	Me	7	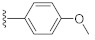	–	73.3%	–	3.3 ± 0.2	9.3 ± 0.4	2.8
**23e**	Ethyl	7	Phenyl	77.5%	24.6%	–	26.8 ± 2.5	39.5 ± 2.3	1.5
**23f**	Propyl	6	Phenyl	28.0%	26.7%	–	–	–	
**23g**	Propyl	7	Phenyl	67.4%	−2.2%	–	–	–	
**23h**	Bn	6	Phenyl	2.0%	7.8%	–	–	–	
**23i**	Bn	7	Phenyl	4.8%	−6.5%	–	–	–	
**23j**	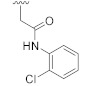	7	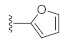	80.0%	47.8%	–	5.2 ± 1.9	12.2 ± 3.3	2.3
RBV				53.2%	–	–	20.0 ± 4.1	45.5 ± 2.6	2.3
2CMA				–	–	–	0.36 ± 0.03	>10	>27

^a^ The inhibitory rate was derived from the mean value of at least two independent experiments. ^b^ The inhibitory rate at the specific concentration of test compounds. ^c^ 50% effective concentration, represented as mean value ± standard deviation, generated from at least six different concentration gradients. ^d^ 50% cytotoxic concentration, generated from at least six different concentration gradients. ^e^ Therapeutic index, calculated as CC_50_/EC_50_.

**Table 3:** Thermal shifts of the melting temperatures of NS5B complexed with tested compounds.

Comp.	Δ*T*_m_^a^			EC_50_^b^
	50 μM	100 μM	200 μM	
**21e**	0.60 ± 0.15	0.85 ± 0.02	1.5 ± 0.01	3.2 ± 0.04
**21k**	0.87 ± 0.12	1.6 ± 0.08	2.1 ± 0.44	3.5 ± 0.3
ADP	0.44 ± 0.11	0.62 ± 0.05	0.88 ± 0.06	–

^a^ The value calculated as the differences between the *T*_m_ of the compound group and the *T*_m_ of the blank control, represented as mean value ± standard deviation from three independent experiments. ^b^ 50% effective concentration, represented as mean value ± standard deviation.

**Table 4:** Sample and control groups with different components in methanol.

No.	Sample	Control
1	50 μM **21e**, 40–640 μM MgCl_2_	50 μM **21e**
2	50 μM **21e**, 80 μL MgCl_2_, 0–5 mm NaOAc	50 μM **21e**, 0–5 mm NaOAc
3	50 μM **21e**, 10–1000 μM MgCl_2_, 5 mm NaOAc	50 μM **21e**, 5 mmh5 NaOAc

## Data Availability

Not applicable.

## Supplementary Materials

The following supporting information can be downloaded at: https://www.mdpi.com/article/10.3390/ijms23115930/s1: ^1^H NMR, ^13^C NMR, and representative ESI-MS spectra of the target compounds.

## Abbreviations

2CMA: 2′-*C*-methyladenosine; 3D: three dimension; ADP: adenosine diphosphate; CC_50_: 50% cytotoxic concentration; CCK-8: cell counting kit-8; CDI: 1,1′-carbonyldiimidazole; Comp.: compound; DAA: direct-acting antiviral; DCM: dichloromethane; DMEM: Dulbecco’s Modified Eagle Medium; DMF: *N*,*N*-dimethylformamide; DMSO: dimethyl sulfoxide; EC_50_: 50% effective concentration; equiv.: equivalent; ESI: electrospray ionization; FDA: U.S. Food and Drug Administration; HCV: hepatitis C virus; HIV: human immunodeficiency virus; IC_50_: 50% inhibitory concentration; MS: mass spectrometry; NMR: nuclear magnetic resonance; NS3/4A: nonstructural protein 3/4A; NS5A: nonstructural protein 5A; NS5B: non-structural protein 5B; NTP: nucleoside triphosphate; OD: optical density; PBS: phosphate buffered saline; PCR: polymerase chain reaction; PDB: Protein Data Bank; Pos.: position; PPi: pyrophosphate; r.t.: room temperature; RBV: ribavirin; RdRp: RNA-dependent RNA polymerase; RMSD: root-mean-square deviation; RNA: ribonucleic acid; RNase H: ribonuclease H; rNTP: ribonucleoside triphosphate; SAR: structure-activity relationship; SP: standard precision; TFA: trifluoroacetate; THF: tetrahydrofuran; TI: therapeutic index; *T*_m_: melting temperature; TMS: tetramethylsilane; Tris-HCl: tris(hydroxymethyl)aminomethane hydrochloride; TSA: thermal shift assay; UTP: uridine triphosphate; UV-Vis: ultraviolet-visible; WHO: World Health Organization.
