# Supplementary material for: Discovery of Novel 3-Hydroxyquinazoline-2,4(1H,3H)-Dione Derivatives: A Series of Metal Ion Chelators with Potent Anti-HCV Activities
Source: Int J Mol Sci. 2022 May 25;23(11):5930. doi: 10.3390/ijms23115930 (PMC9180926; doi:10.3390/ijms23115930)
Supplement: Supplementary file 1 [file ijms-23-05930-s001.zip › ijms-1738365-supplementary.pdf]

Discovery of Novel 3-Hydroxyquinazoline-2,4(1*H*,3*H*)-dione Derivatives: A Series of Metal Ion Chelators with Potent Anti-HCV Activities

Yang Cao [1], Abudumijiti Aimaiti [2], Zeyun Zhu [1], Lu Zhou [1,*] and Deyong Ye [1,*]

[1] Department of Medicinal Chemistry, School of Pharmacy, Fudan University, 826 Zhangheng Rd, Shanghai 201203, P.R. China; ycao14@fudan.edu.cn (Y.C.); traveller-zzy@outlook.com (Z.Z.)

[2] Shanghai Medical College, Fudan University, 130 Dongan Rd, Shanghai 200032, P.R. China; abu@fudan.edu.cn

* Correspondence: dyye@shmu.edu.cn (D.Y.); zhoulu@fudan.edu.cn (L.Z.)

Table of Contents

Figure S1. ^{1}H NMR (400 MHz, DMSO-d_6) spectrum of **10a** ... 7
Figure S2. Magnified ^{1}H NMR (400 MHz, DMSO-d_6) spectrum fragments of **10a** ... 7
Figure S3. ^{13}C NMR (151 MHz, DMSO-d_6) spectrum of **10a** ... 8
Figure S4. Mass spectrum (negative ionization) of **10a** ... 9
Figure S5. ^{1}H NMR (400 MHz, DMSO-d_6) spectrum of **10b** ... 10
Figure S6. Magnified ^{1}H NMR (400 MHz, DMSO-d_6) spectrum fragments of **10b** ... 10
Figure S7. ^{13}C NMR (151 MHz, DMSO-d_6) spectrum of **10b** ... 11
Figure S8. ^{1}H NMR (400 MHz, DMSO-d_6) spectrum of **10c** ... 11
Figure S9. Magnified ^{1}H NMR (400 MHz, DMSO-d_6) spectrum fragments of **10c** ... 12
Figure S10. ^{13}C NMR (151 MHz, DMSO-d_6) spectrum of **10c** ... 12
Figure S11. ^{1}H NMR (400 MHz, DMSO-d_6) spectrum of **10d** ... 13
Figure S12. Magnified ^{1}H NMR (400 MHz, DMSO-d_6) spectrum fragments of **10d** ... 13
Figure S13. ^{13}C NMR (151 MHz, DMSO-d_6) spectrum of **10d** ... 14
Figure S14. ^{1}H NMR (400 MHz, DMSO-d_6) spectrum of **10e** ... 14
Figure S15. Magnified ^{1}H NMR (400 MHz, DMSO-d_6) spectrum fragments of **10e** ... 15
Figure S16. ^{13}C NMR (151 MHz, DMSO-d_6) spectrum of **10e** ... 15
Figure S17. ^{1}H NMR (400 MHz, DMSO-d_6) spectrum of **10f** ... 16
Figure S18. Magnified ^{1}H NMR (400 MHz, DMSO-d_6) spectrum fragments of **10f** ... 16
Figure S19. ^{13}C NMR (151 MHz, DMSO-d_6) spectrum of **10f** ... 17
Figure S20. ^{1}H NMR (400 MHz, DMSO-d_6) spectrum of **10g** ... 17
Figure S21. Magnified ^{1}H NMR (400 MHz, DMSO-d_6) spectrum fragments of **10g** ... 18
Figure S22. ^{13}C NMR (151 MHz, DMSO-d_6) spectrum of **10g** ... 18
Figure S23. ^{1}H NMR (400 MHz, DMSO-d_6) spectrum of **10h** ... 19
Figure S24. Magnified ^{1}H NMR (400 MHz, DMSO-d_6) spectrum fragments of **10h** ... 19
Figure S25. ^{13}C NMR (151 MHz, DMSO-d_6) spectrum of **10h** ... 20
Figure S26. ^{1}H NMR (400 MHz, DMSO-d_6) spectrum of **10i** ... 20
Figure S27. Magnified ^{1}H NMR (400 MHz, DMSO-d_6) spectrum fragments of **10i** ... 21
Figure S28. ^{13}C NMR (151 MHz, DMSO-d_6) spectrum of **10i** ... 21
Figure S29. ^{1}H NMR (400 MHz, DMSO-d_6) spectrum of **10j** ... 22
Figure S30. Magnified ^{1}H NMR (400 MHz, DMSO-d_6) spectrum fragments of **10j** ... 22
Figure S31. ^{13}C NMR (151 MHz, DMSO-d_6) spectrum of **10j** ... 23
Figure S32. ^{1}H NMR (400 MHz, DMSO-d_6) spectrum of **10k** ... 23
Figure S33. Magnified ^{1}H NMR (400 MHz, DMSO-d_6) spectrum fragments of **10k** ... 24
Figure S34. ^{13}C NMR (151 MHz, DMSO-d_6) spectrum of **10k** ... 24
Figure S35. ^{1}H NMR (400 MHz, DMSO-d_6) spectrum of **10l** ... 25
Figure S36. Magnified ^{1}H NMR (400 MHz, DMSO-d_6) spectrum fragments of **10l** ... 25
Figure S37. ^{13}C NMR (151 MHz, DMSO-d_6) spectrum of **10l** ... 26
Figure S38. ^{1}H NMR (400 MHz, DMSO-d_6) spectrum of **10m** ... 26
Figure S39. Magnified ^{1}H NMR (400 MHz, DMSO-d_6) spectrum fragments of **10m** ... 27
Figure S40. ^{13}C NMR (151 MHz, DMSO-d_6) spectrum of **10m** ... 27
Figure S41. ^{1}H NMR (400 MHz, DMSO-d_6) spectrum of **10n** ... 28
Figure S42. Magnified ^{1}H NMR (400 MHz, DMSO-d_6) spectrum fragments of **10n** ... 28

Figure S43. ^{13}C NMR (151 MHz, DMSO-d_6) spectrum of **10n** 29
Figure S44. ^{1}H NMR (400 MHz, DMSO-d_6) spectrum of **10o** 29
Figure S45. Magnified ^{1}H NMR (400 MHz, DMSO-d_6) spectrum fragments of **10o** 30
Figure S46. ^{13}C NMR (151 MHz, DMSO-d_6) spectrum of **10o** 30
Figure S47. ^{1}H NMR (400 MHz, DMSO-d_6) spectrum of **10p** 31
Figure S48. Magnified ^{1}H NMR (400 MHz, DMSO-d_6) spectrum fragments of **10p** 31
Figure S49. ^{13}C NMR (151 MHz, DMSO-d_6) spectrum of **10p** 32
Figure S50. ^{1}H NMR (400 MHz, DMSO-d_6) spectrum of **18a** 32
Figure S51. Magnified ^{1}H NMR (400 MHz, DMSO-d_6) spectrum fragments of **18a** 33
Figure S52. ^{13}C NMR (151 MHz, DMSO-d_6) spectrum of **18a** 33
Figure S53. Mass spectrum (negative ionization) of **18a** 34
Figure S54. ^{1}H NMR (400 MHz, DMSO-d_6) spectrum of **18b** 35
Figure S55. Magnified ^{1}H NMR (400 MHz, DMSO-d_6) spectrum fragments of **18b** 35
Figure S56. ^{13}C NMR (151 MHz, DMSO-d_6) spectrum of **18b** 36
Figure S57. ^{1}H NMR (400 MHz, DMSO-d_6) spectrum of **18c** 36
Figure S58. Magnified ^{1}H NMR (400 MHz, DMSO-d_6) spectrum fragments of **18c** 37
Figure S59. ^{13}C NMR (151 MHz, DMSO-d_6) spectrum of **18c** 37
Figure S60. Mass spectrum (negative ionization) of **18c** 38
Figure S61. ^{1}H NMR (400 MHz, DMSO-d_6) spectrum of **19** 39
Figure S62. ^{13}C NMR (151 MHz, DMSO-d_6) spectrum of **19** 39
Figure S63. Mass spectrum (negative ionization) of **19** 40
Figure S64. ^{1}H NMR (400 MHz, DMSO-d_6) spectrum of **21a** 41
Figure S65. Magnified ^{1}H NMR (400 MHz, DMSO-d_6) spectrum fragments of **21a** 41
Figure S66. ^{13}C NMR (151 MHz, DMSO-d_6) spectrum of **21a** 42
Figure S67. Mass spectrum (negative ionization) of **21a** 43
Figure S68. ^{1}H NMR (400 MHz, DMSO-d_6) spectrum of **21b** 44
Figure S69. Magnified ^{1}H NMR (400 MHz, DMSO-d_6) spectrum fragments of **21b** 44
Figure S70. ^{13}C NMR (151 MHz, DMSO-d_6) spectrum of **21b** 45
Figure S71. ^{1}H NMR (400 MHz, DMSO-d_6) spectrum of **21c** 45
Figure S72. Magnified ^{1}H NMR (400 MHz, DMSO-d_6) spectrum fragments of **21c** 46
Figure S73. ^{13}C NMR (151 MHz, DMSO-d_6) spectrum of **21c** 46
Figure S74. Mass spectrum (negative ionization) of **21c** 47
Figure S75. ^{1}H NMR (400 MHz, DMSO-d_6) spectrum of **21d** 48
Figure S76. Magnified ^{1}H NMR (400 MHz, DMSO-d_6) spectrum fragments of **21d** 48
Figure S77. ^{13}C NMR (151 MHz, DMSO-d_6) spectrum of **21d** 49
Figure S78. Mass spectrum (negative ionization) of **21d** 50
Figure S79. ^{1}H NMR (400 MHz, DMSO-d_6) spectrum of **21e** 51
Figure S80. Magnified ^{1}H NMR (400 MHz, DMSO-d_6) spectrum fragments of **21e** 51
Figure S81. ^{13}C NMR (151 MHz, DMSO-d_6) spectrum of **21e** 52
Figure S82. Mass spectrum (negative ionization) of **21e** 53
Figure S83. ^{1}H NMR (400 MHz, DMSO-d_6) spectrum of **21f** 54
Figure S84. Magnified ^{1}H NMR (400 MHz, DMSO-d_6) spectrum fragments of **21f** 54
Figure S85. ^{13}C NMR (151 MHz, DMSO-d_6) spectrum of **21f** 55
Figure S86. Mass spectrum (negative ionization) of **21f** 56

Figure S87. ^{1}H NMR (400 MHz, DMSO-d_6) spectrum of **21g** 57
Figure S88. Magnified ^{1}H NMR (400 MHz, DMSO-d_6) spectrum fragments of **21g** 57
Figure S89. ^{13}C NMR (151 MHz, DMSO-d_6) spectrum of **21g** 58
Figure S90. Mass spectrum (negative ionization) of **21g** 59
Figure S91. ^{1}H NMR (400 MHz, DMSO-d_6) spectrum of **21h** 60
Figure S92. Magnified ^{1}H NMR (400 MHz, DMSO-d_6) spectrum fragments of **21h** 60
Figure S93. ^{13}C NMR (151 MHz, DMSO-d_6) spectrum of **21h** 61
Figure S94. ^{1}H NMR (400 MHz, DMSO-d_6) spectrum of **21i** 61
Figure S95. Magnified ^{1}H NMR (400 MHz, DMSO-d_6) spectrum fragments of **21i** 62
Figure S96. ^{13}C NMR (151 MHz, DMSO-d_6) spectrum of **21i** 62
Figure S97. Mass spectrum (negative ionization) of **21i** 63
Figure S98. ^{1}H NMR (400 MHz, DMSO-d_6) spectrum of **21j** 64
Figure S99. Magnified ^{1}H NMR (400 MHz, DMSO-d_6) spectrum fragments of **21j** 64
Figure S100. ^{13}C NMR (151 MHz, DMSO-d_6) spectrum of **21j** 65
Figure S101. Mass spectrum (negative ionization) of **21j** 66
Figure S102. ^{1}H NMR (400 MHz, DMSO-d_6) spectrum of **21k** 67
Figure S103. Magnified ^{1}H NMR (400 MHz, DMSO-d_6) spectrum fragments of **21k** 67
Figure S104. ^{13}C NMR (151 MHz, DMSO-d_6) spectrum of **21k** 68
Figure S105. Mass spectrum (negative ionization) of **21k** 69
Figure S106. ^{1}H NMR (400 MHz, DMSO-d_6) spectrum of **21l** 70
Figure S107. Magnified ^{1}H NMR (400 MHz, DMSO-d_6) spectrum fragments of **21l** 70
Figure S108. ^{13}C NMR (151 MHz, DMSO-d_6) spectrum of **21l** 71
Figure S109. ^{1}H NMR (400 MHz, DMSO-d_6) spectrum of **21m** 71
Figure S110. Magnified ^{1}H NMR (400 MHz, DMSO-d_6) spectrum fragments of **21m** 72
Figure S111. ^{13}C NMR (151 MHz, DMSO-d_6) spectrum of **21m** 72
Figure S112. Mass spectrum (positive ionization) of **21m** 73
Figure S113. ^{1}H NMR (400 MHz, DMSO-d_6) spectrum of **21n** 74
Figure S114. Magnified ^{1}H NMR (400 MHz, DMSO-d_6) spectrum fragments of **21n** 74
Figure S115. ^{13}C NMR (151 MHz, DMSO-d_6) spectrum of **21n** 75
Figure S116. Mass spectrum (positive ionization) of **21n** 76
Figure S117. ^{1}H NMR (400 MHz, DMSO-d_6) spectrum of **21o** 77
Figure S118. Magnified ^{1}H NMR (400 MHz, DMSO-d_6) spectrum fragments of **21o** 77
Figure S119. ^{13}C NMR (151 MHz, DMSO-d_6) spectrum of **21o** 78
Figure S120. Mass spectrum (positive ionization) of **21o** 79
Figure S121. ^{1}H NMR (400 MHz, DMSO-d_6) spectrum of **21p** 80
Figure S122. Magnified ^{1}H NMR (400 MHz, DMSO-d_6) spectrum fragments of **21p** 80
Figure S123. ^{13}C NMR (151 MHz, DMSO-d_6) spectrum of **21p** 81
Figure S124. Mass spectrum (positive ionization) of **21p** 82
Figure S125. ^{1}H NMR (400 MHz, DMSO-d_6) spectrum of **21q** 83
Figure S126. Magnified ^{1}H NMR (400 MHz, DMSO-d_6) spectrum fragments of **21q** 83
Figure S127. ^{13}C NMR (151 MHz, DMSO-d_6) spectrum of **21q** 84
Figure S128. ^{1}H NMR (400 MHz, DMSO-d_6) spectrum of **21r** 84
Figure S129. Magnified ^{1}H NMR (400 MHz, DMSO-d_6) spectrum fragments of **21r** 85
Figure S130. ^{13}C NMR (151 MHz, DMSO-d_6) spectrum of **21r** 85

Figure S131. ^{1}H NMR (400 MHz, DMSO-d_6) spectrum of **21s** 86
Figure S132. Magnified ^{1}H NMR (400 MHz, DMSO-d_6) spectrum fragments of **21s** 86
Figure S133. ^{13}C NMR (151 MHz, DMSO-d_6) spectrum of **21s** 87
Figure S134. ^{1}H NMR (400 MHz, DMSO-d_6) spectrum of **21t** 87
Figure S135. Magnified ^{1}H NMR (400 MHz, DMSO-d_6) spectrum fragments of **21t** 88
Figure S136. ^{13}C NMR (151 MHz, DMSO-d_6) spectrum of **21t** 88
Figure S137. Mass spectrum (negative ionization) of **21t** 89
Figure S138. ^{1}H NMR (400 MHz, DMSO-d_6) spectrum of **21u** 90
Figure S139. Magnified ^{1}H NMR (400 MHz, DMSO-d_6) spectrum fragments of **21u** 90
Figure S140. ^{13}C NMR (151 MHz, DMSO-d_6) spectrum of **21u** 91
Figure S141. ^{1}H NMR (400 MHz, DMSO-d_6) spectrum of **21v** 91
Figure S142. Magnified ^{1}H NMR (400 MHz, DMSO-d_6) spectrum fragments of **21v** 92
Figure S143. ^{13}C NMR (151 MHz, DMSO-d_6) spectrum of **21v** 92
Figure S144. ^{1}H NMR (400 MHz, DMSO-d_6) spectrum of **21w** 93
Figure S145. Magnified ^{1}H NMR (400 MHz, DMSO-d_6) spectrum fragments of **21w** 93
Figure S146. ^{13}C NMR (151 MHz, DMSO-d_6) spectrum of **21w** 94
Figure S147. Mass spectrum (negative ionization) of **21w** 95
Figure S148. ^{1}H NMR (400 MHz, DMSO-d_6) spectrum of **21x** 96
Figure S149. Magnified ^{1}H NMR (400 MHz, DMSO-d_6) spectrum fragments of **21x** 96
Figure S150. ^{13}C NMR (151 MHz, DMSO-d_6) spectrum of **21x** 97
Figure S151. Mass spectrum (negative ionization) of **21x** 98
Figure S152. ^{1}H NMR (400 MHz, DMSO-d_6) spectrum of **23a** 99
Figure S153. Magnified ^{1}H NMR (400 MHz, DMSO-d_6) spectrum fragments of **23a** 99
Figure S154. ^{13}C NMR (151 MHz, DMSO-d_6) spectrum of **23a** 100
Figure S155. Mass spectrum (negative ionization) of **23a** 101
Figure S156. ^{1}H NMR (400 MHz, DMSO-d_6) spectrum of **23b** 102
Figure S157. Magnified ^{1}H NMR (400 MHz, DMSO-d_6) spectrum fragments of **23b** 102
Figure S158. ^{13}C NMR (151 MHz, DMSO-d_6) spectrum of **23b** 103
Figure S159. ^{1}H NMR (400 MHz, DMSO-d_6) spectrum of **23c** 103
Figure S160. Magnified ^{1}H NMR (400 MHz, DMSO-d_6) spectrum fragments of **23c** 104
Figure S161. ^{13}C NMR (151 MHz, DMSO-d_6) spectrum of **23c** 104
Figure S162. Mass spectrum (negative ionization) of **23c** 105
Figure S163. ^{1}H NMR (400 MHz, DMSO-d_6) spectrum of **23d** 106
Figure S164. ^{13}C NMR (151 MHz, DMSO-d_6) spectrum of **23d** 106
Figure S165. Mass spectrum (negative ionization) of **23d** 107
Figure S166. ^{1}H NMR (400 MHz, DMSO-d_6) spectrum of **23e** 108
Figure S167. Magnified ^{1}H NMR (400 MHz, DMSO-d_6) spectrum fragments of **23e** 108
Figure S168. ^{13}C NMR (151 MHz, DMSO-d_6) spectrum of **23e** 109
Figure S169. Mass spectrum (negative ionization) of **23e** 110
Figure S170. ^{1}H NMR (400 MHz, DMSO-d_6) spectrum of **23f** 111
Figure S171. Magnified ^{1}H NMR (400 MHz, DMSO-d_6) spectrum fragments of **23f** 111
Figure S172. ^{13}C NMR (151 MHz, DMSO-d_6) spectrum of **23f** 112
Figure S173. ^{1}H NMR (400 MHz, DMSO-d_6) spectrum of **23g** 112
Figure S174. Magnified ^{1}H NMR (400 MHz, DMSO-d_6) spectrum fragments of **23g** 113

Figure S175. ^{13}C NMR (151 MHz, DMSO-d_6) spectrum of **23g** 113
Figure S176. ^{1}H NMR (400 MHz, DMSO-d_6) spectrum of **23h** 114
Figure S177. Magnified ^{1}H NMR (400 MHz, DMSO-d_6) spectrum fragments of **23h** 114
Figure S178. ^{13}C NMR (151 MHz, DMSO-d_6) spectrum of **23h** 115
Figure S179. ^{1}H NMR (400 MHz, DMSO-d_6) spectrum of **23i** 115
Figure S180. Magnified ^{1}H NMR (400 MHz, DMSO-d_6) spectrum fragments of **23i** 116
Figure S181. ^{13}C NMR (151 MHz, DMSO-d_6) spectrum of **23i** 116
Figure S182. Mass spectrum (positive ionization) of **23i** 117
Figure S183. ^{1}H NMR (400 MHz, DMSO-d_6) spectrum of **23j** 118
Figure S184. Magnified ^{1}H NMR (400 MHz, DMSO-d_6) spectrum fragments of **23j** 118
Figure S185. ^{13}C NMR (151 MHz, DMSO-d_6) spectrum of **23j** 119

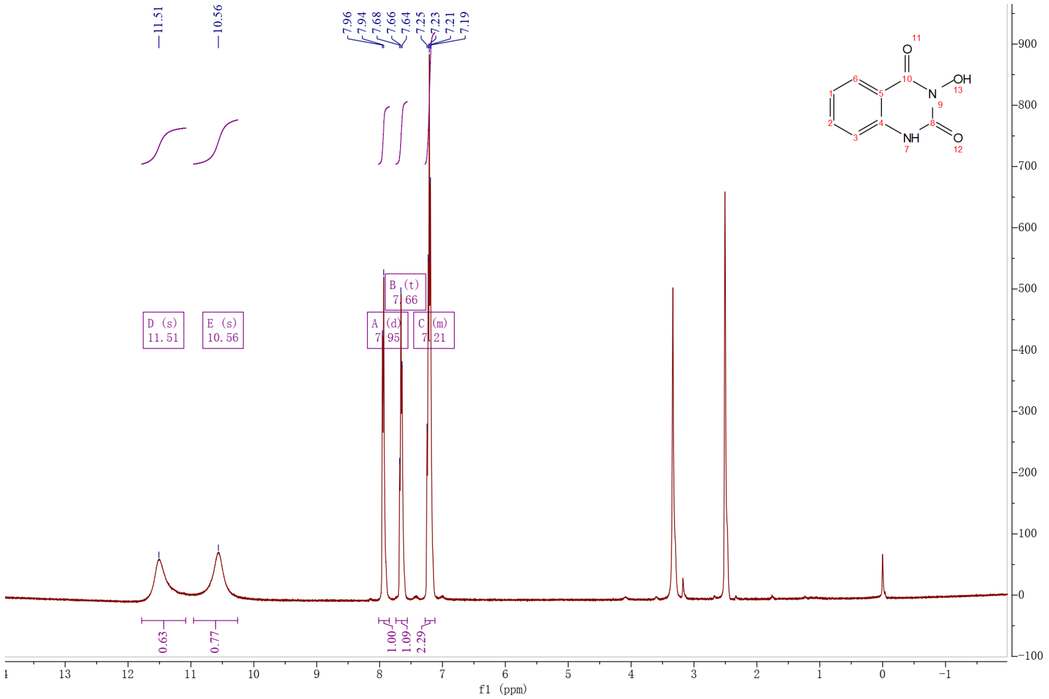

Figure S1. ^{1}H NMR (400 MHz, DMSO-d_6) spectrum of **10a**

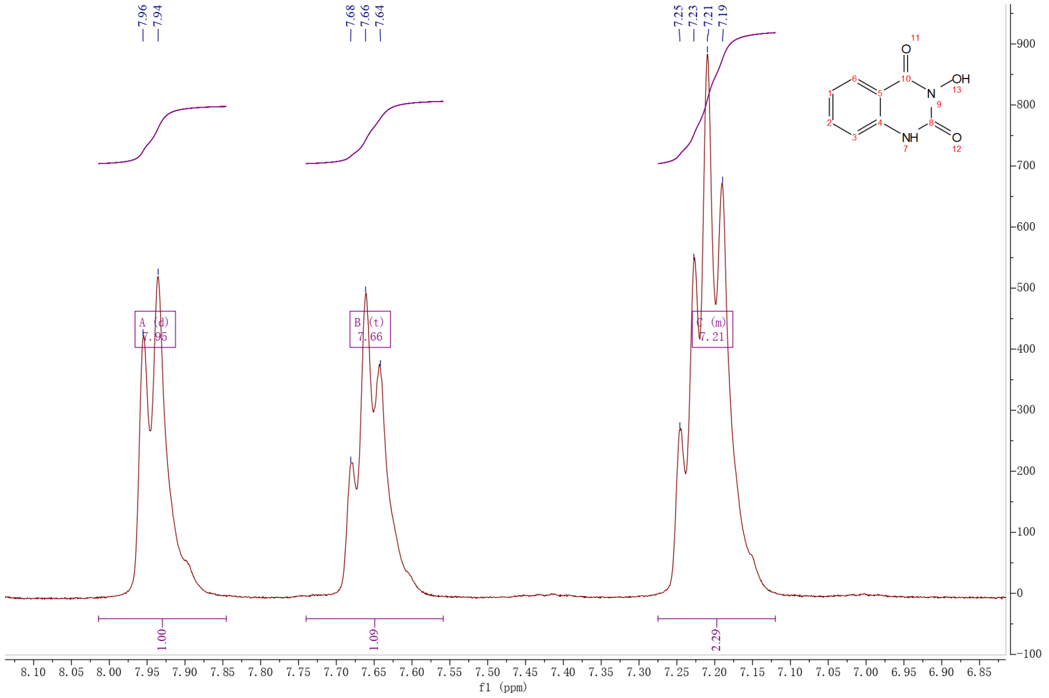

Figure S2. Magnified ^{1}H NMR (400 MHz, DMSO-d_6) spectrum fragments of **10a**

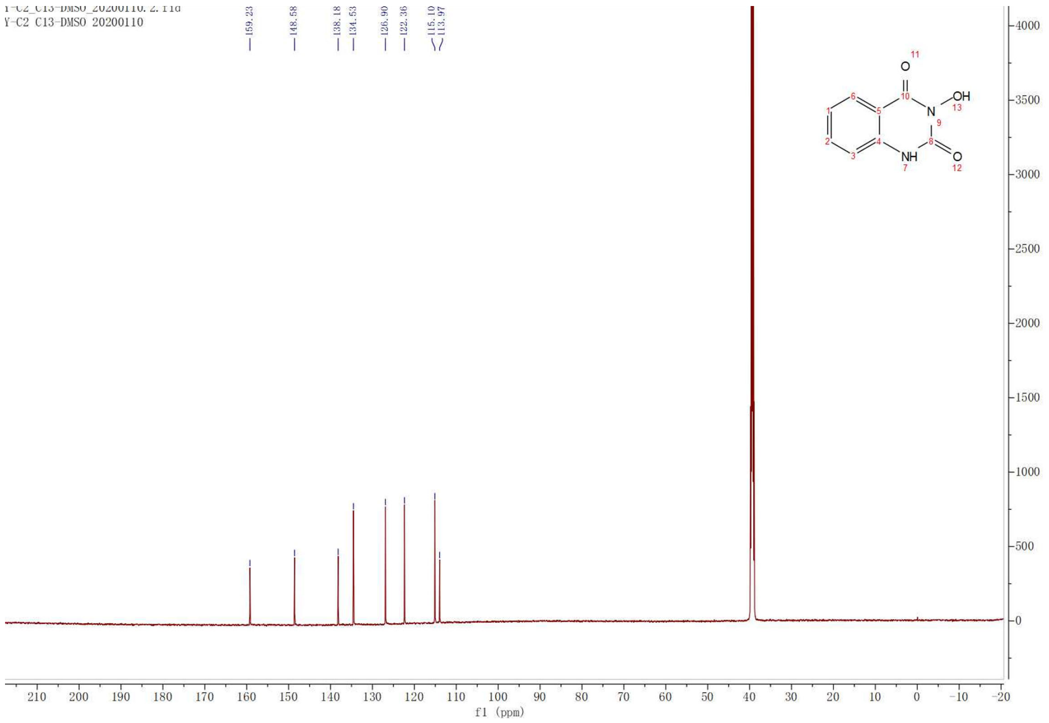

Figure S3. ^{13}C NMR (151 MHz, DMSO-d_6) spectrum of **10a**

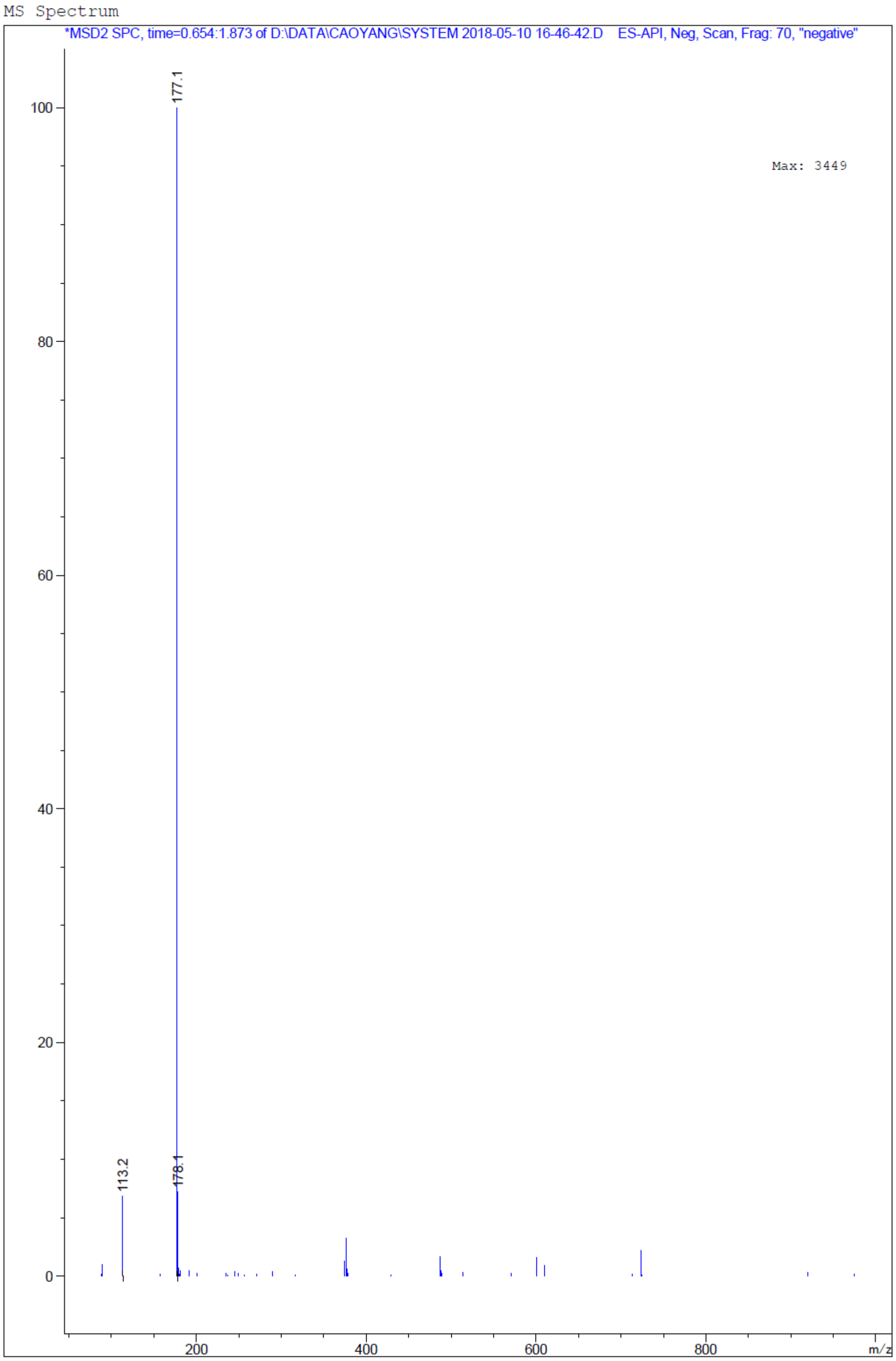

Figure S4. Mass spectrum (negative ionization) of **10a**

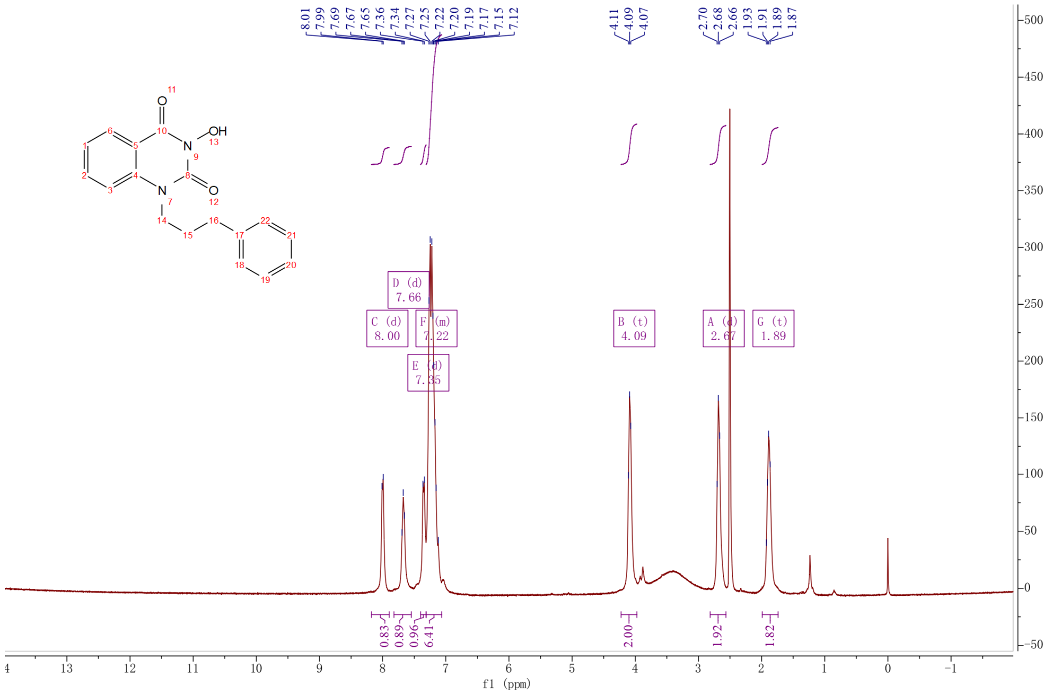

Figure S5. ^{1}H NMR (400 MHz, DMSO-d_6) spectrum of **10b**

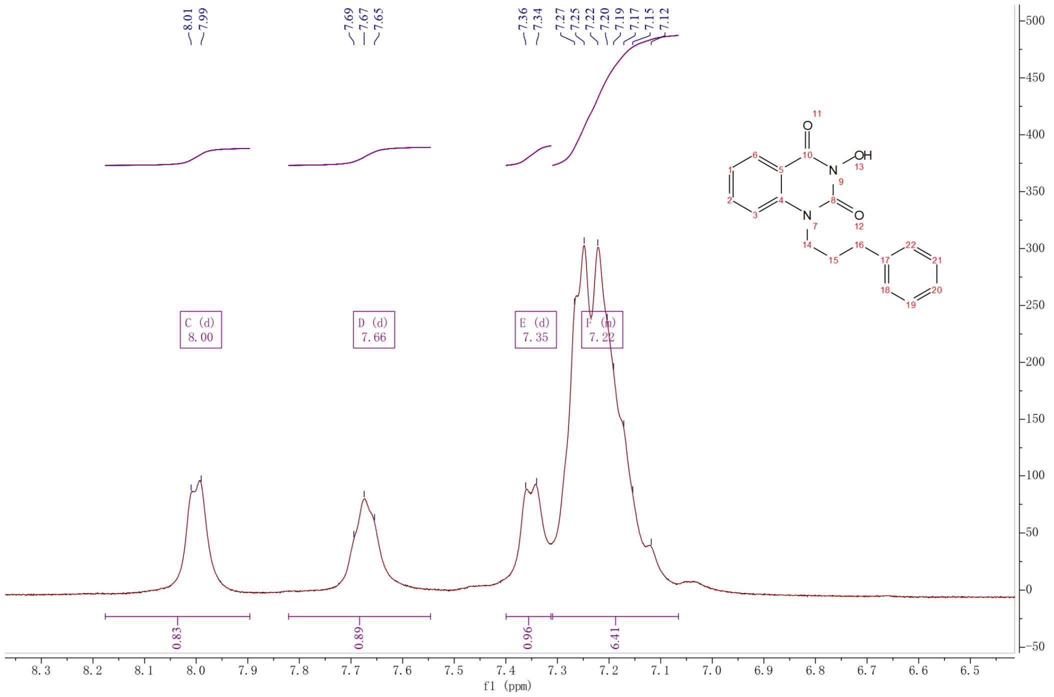

Figure S6. Magnified ^{1}H NMR (400 MHz, DMSO-d_6) spectrum fragments of **10b**

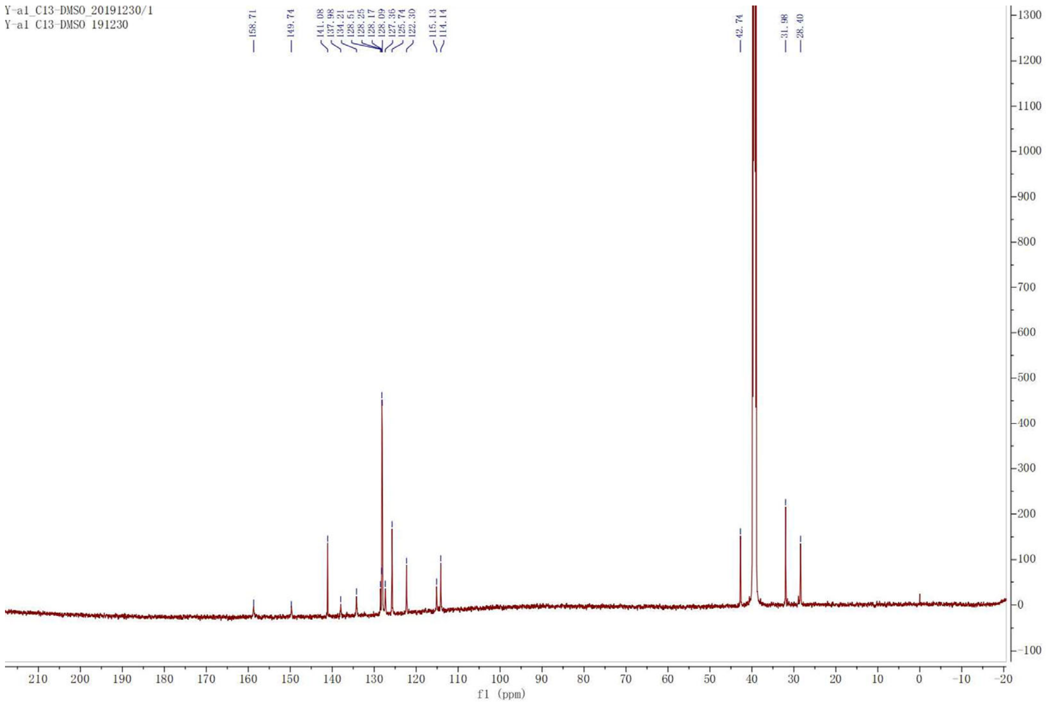

Figure S7. ^{13}C NMR (151 MHz, DMSO-d_6) spectrum of **10b**

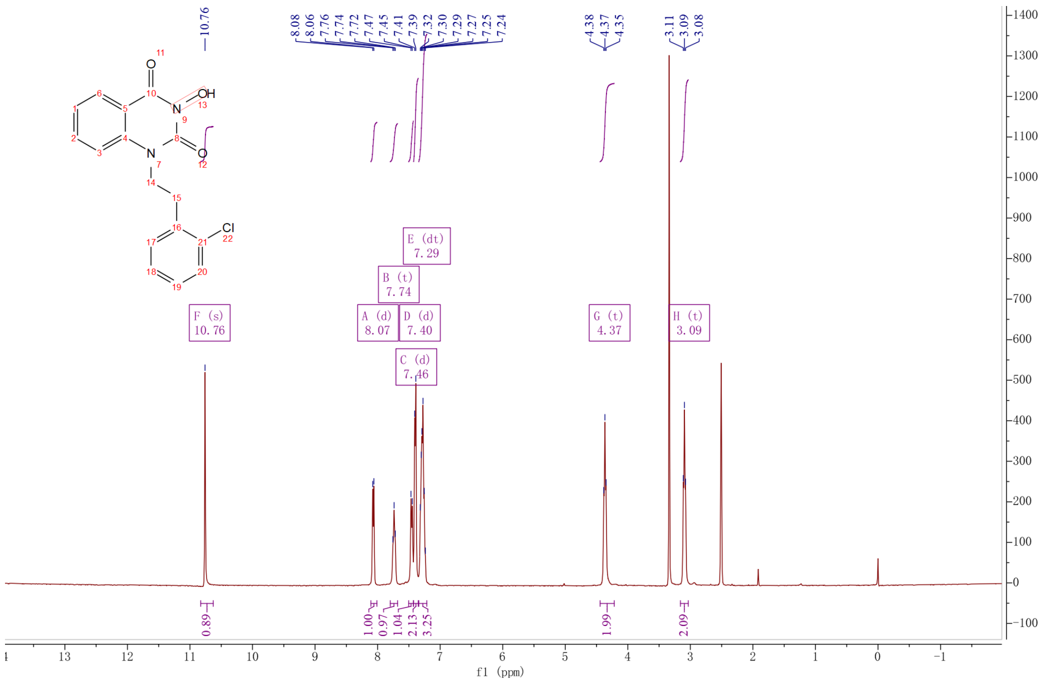

Figure S8. ^{1}H NMR (400 MHz, DMSO-d_6) spectrum of **10c**

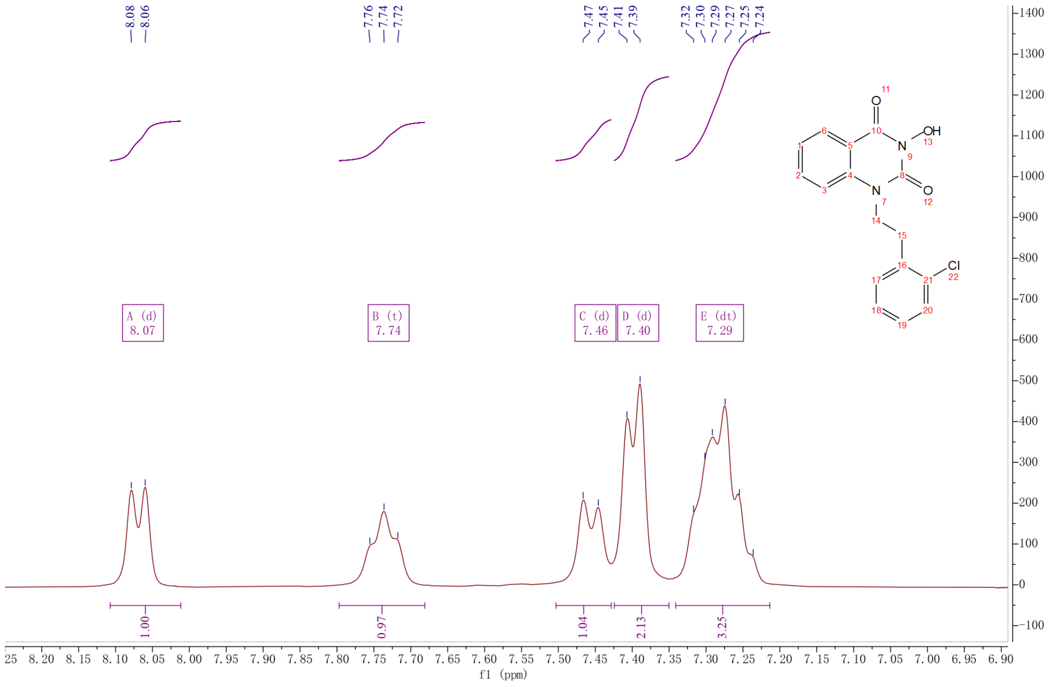

Figure S9. Magnified ^{1}H NMR (400 MHz, DMSO-d_6) spectrum fragments of **10c**

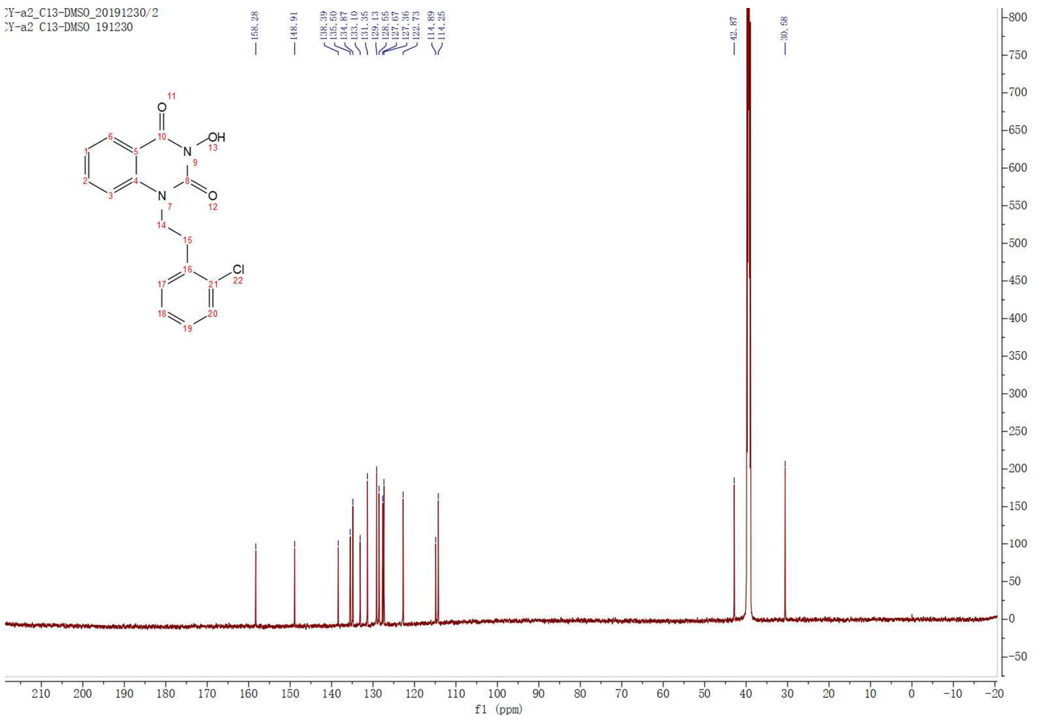

Figure S10. ^{13}C NMR (151 MHz, DMSO-d_6) spectrum of **10c**

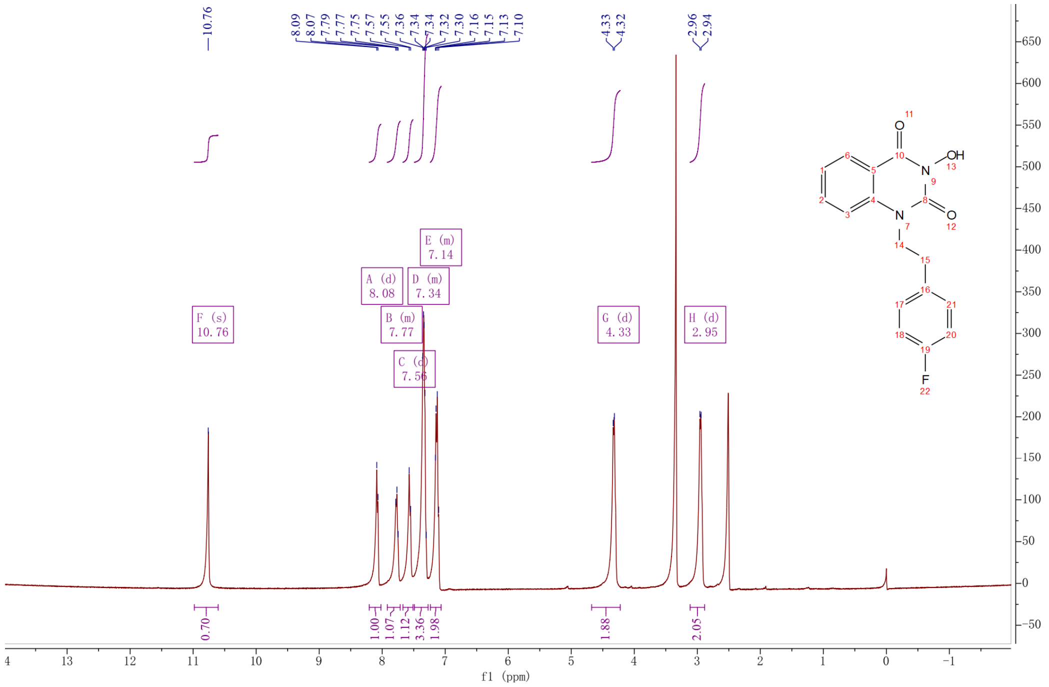

Figure S11. ^{1}H NMR (400 MHz, DMSO-d_6) spectrum of **10d**

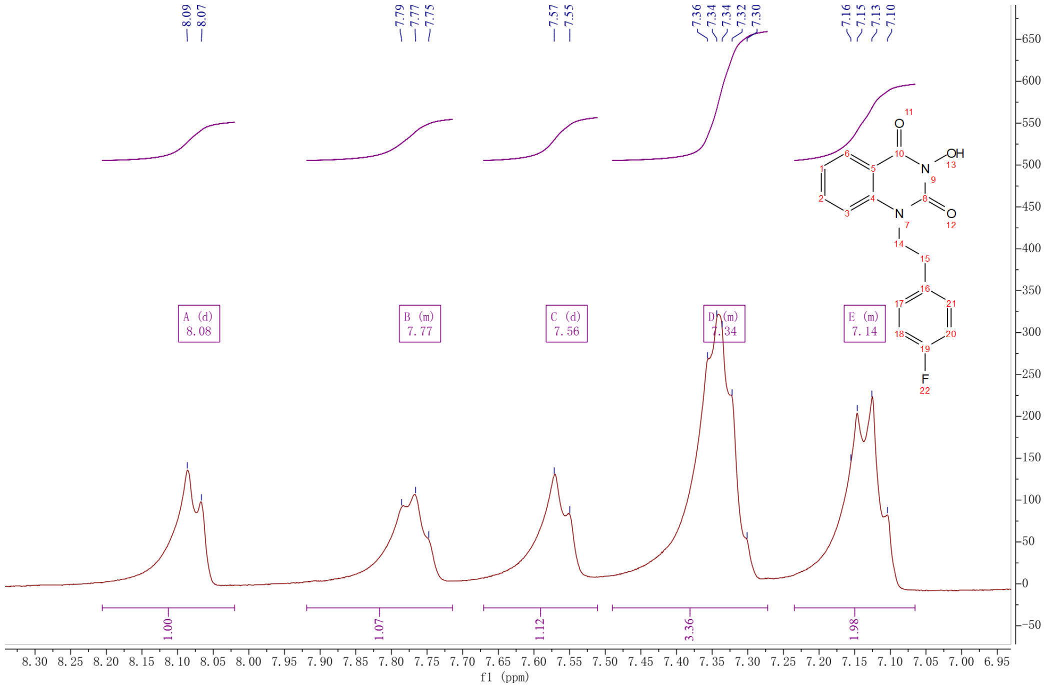

Figure S12. Magnified ^{1}H NMR (400 MHz, DMSO-d_6) spectrum fragments of **10d**

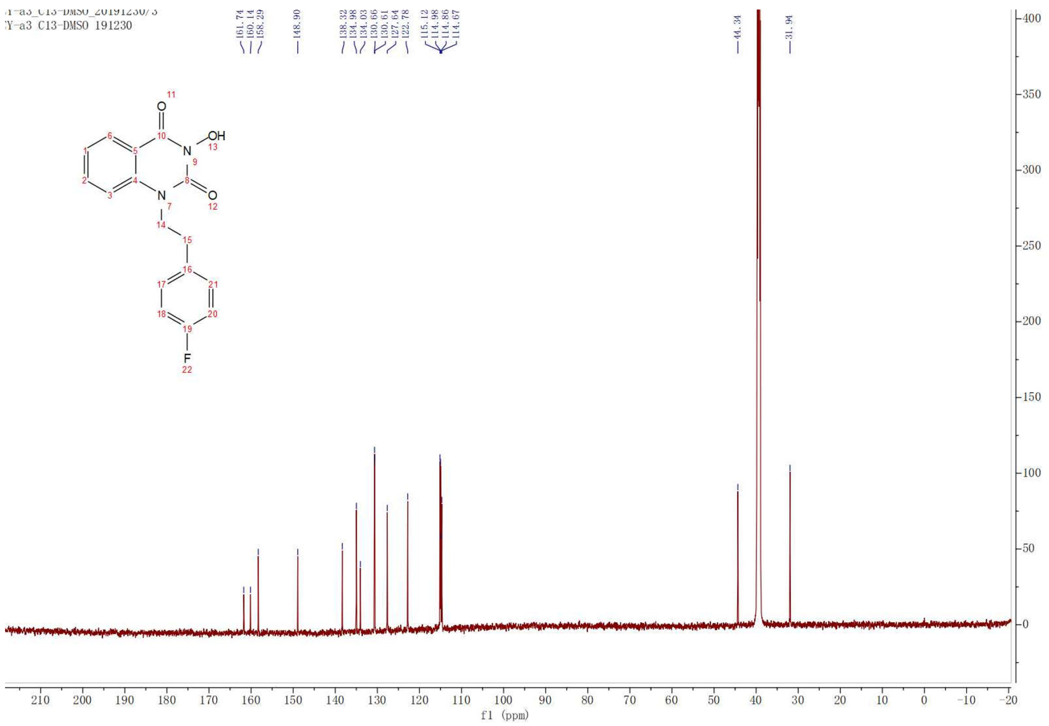

Figure S13. ^{13}C NMR (151 MHz, DMSO-d_6) spectrum of **10d**

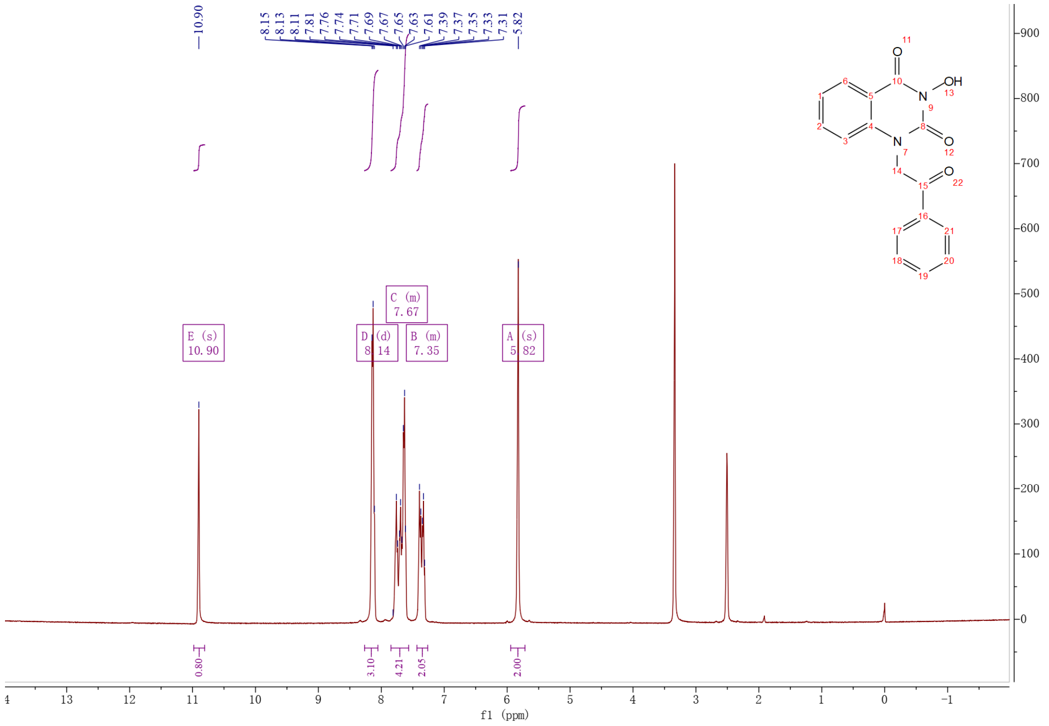

Figure S14. ^1H NMR (400 MHz, DMSO-d_6) spectrum of **10e**

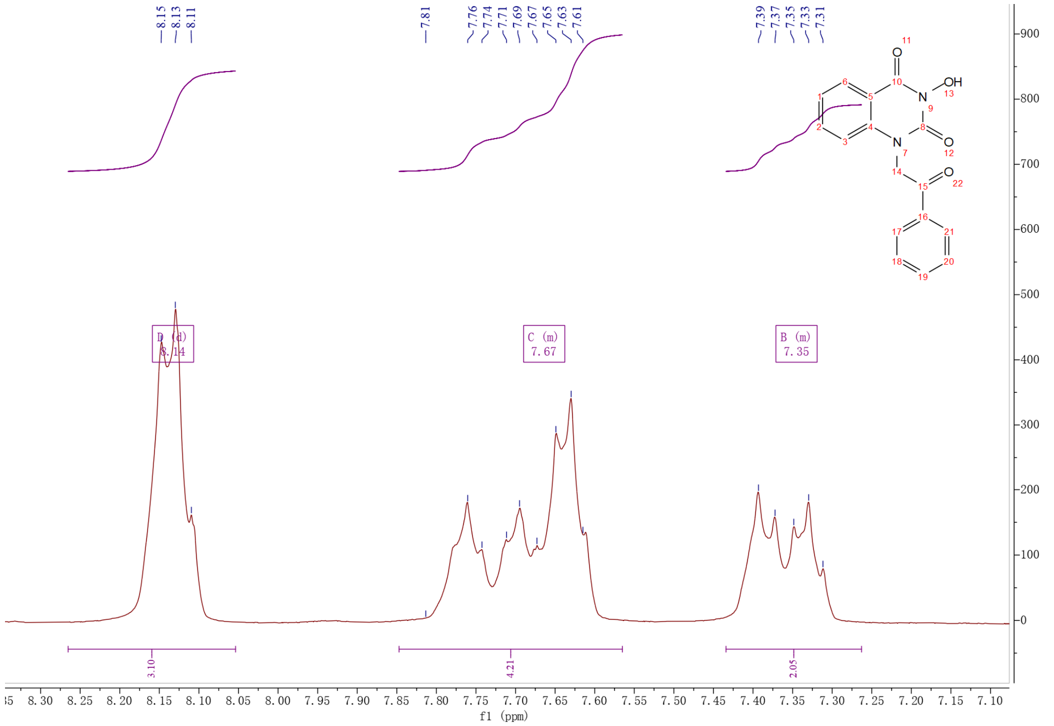

Figure S15. Magnified ^{1}H NMR (400 MHz, DMSO-d_6) spectrum fragments of **10e**

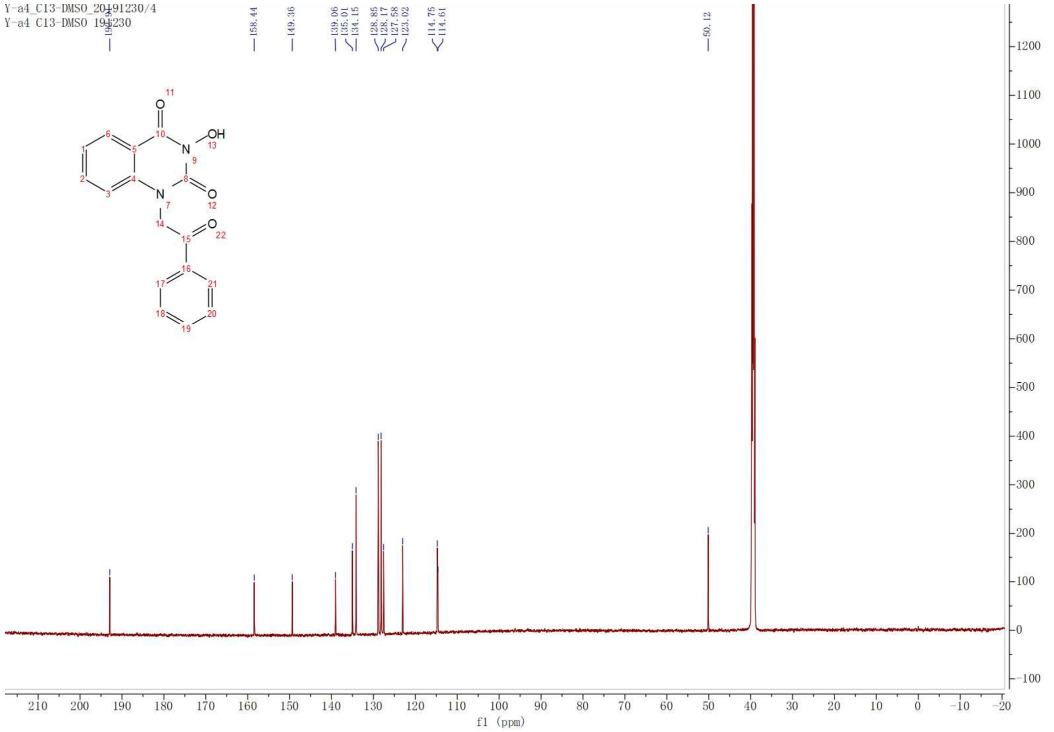

Figure S16. ^{13}C NMR (151 MHz, DMSO-d_6) spectrum of **10e**

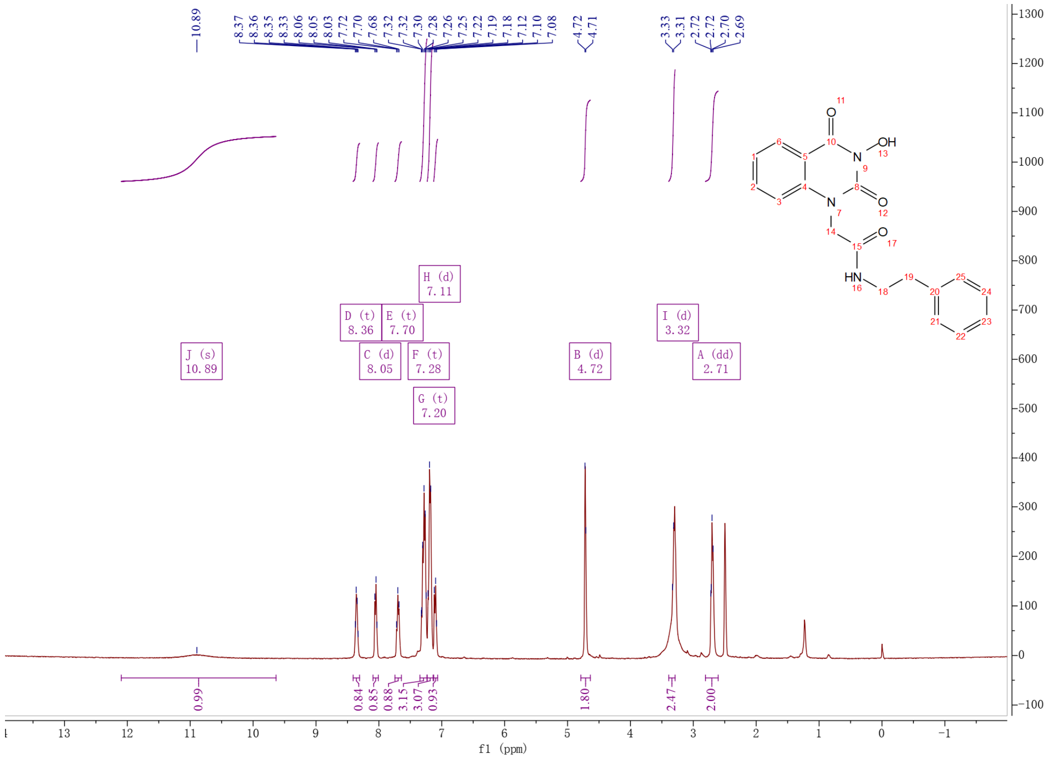

Figure S17. ^{1}H NMR (400 MHz, DMSO-d_6) spectrum of **10f**

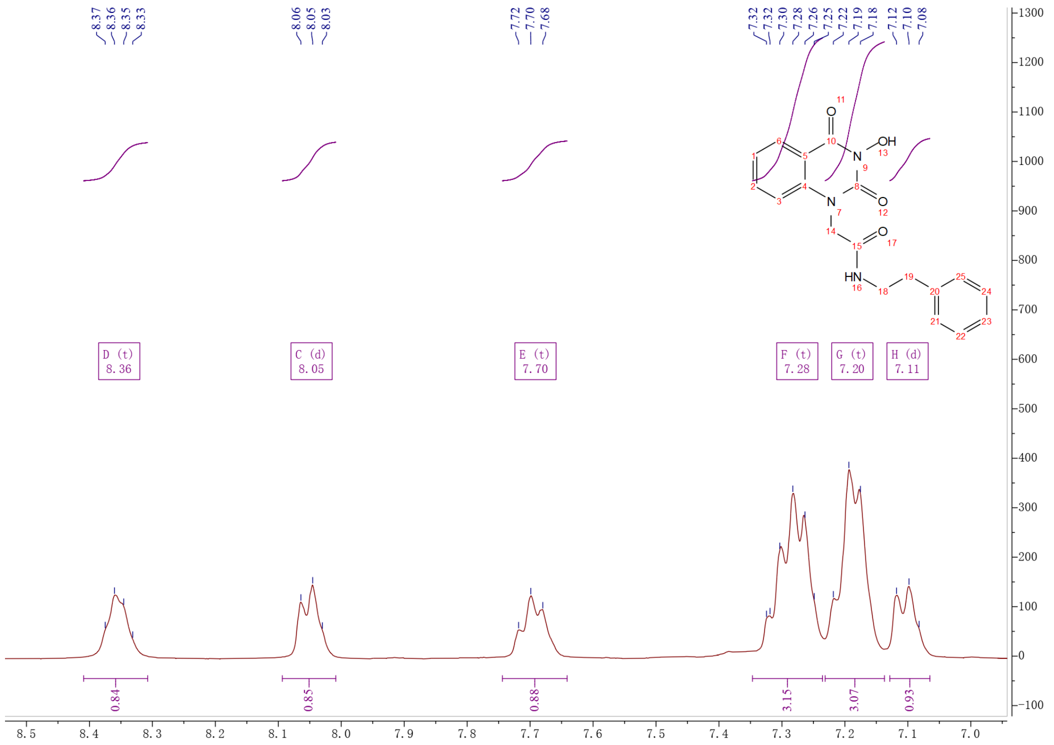

Figure S18. Magnified ^{1}H NMR (400 MHz, DMSO-d_6) spectrum fragments of **10f**

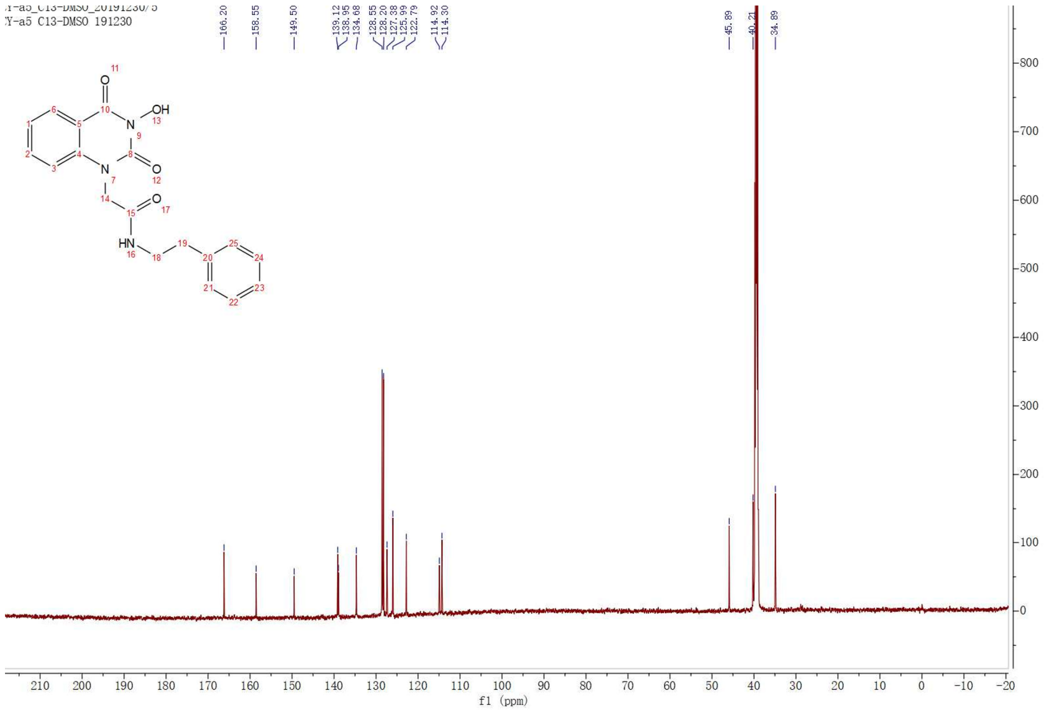

Figure S19. ^{13}C NMR (151 MHz, DMSO-d_6) spectrum of **10f**

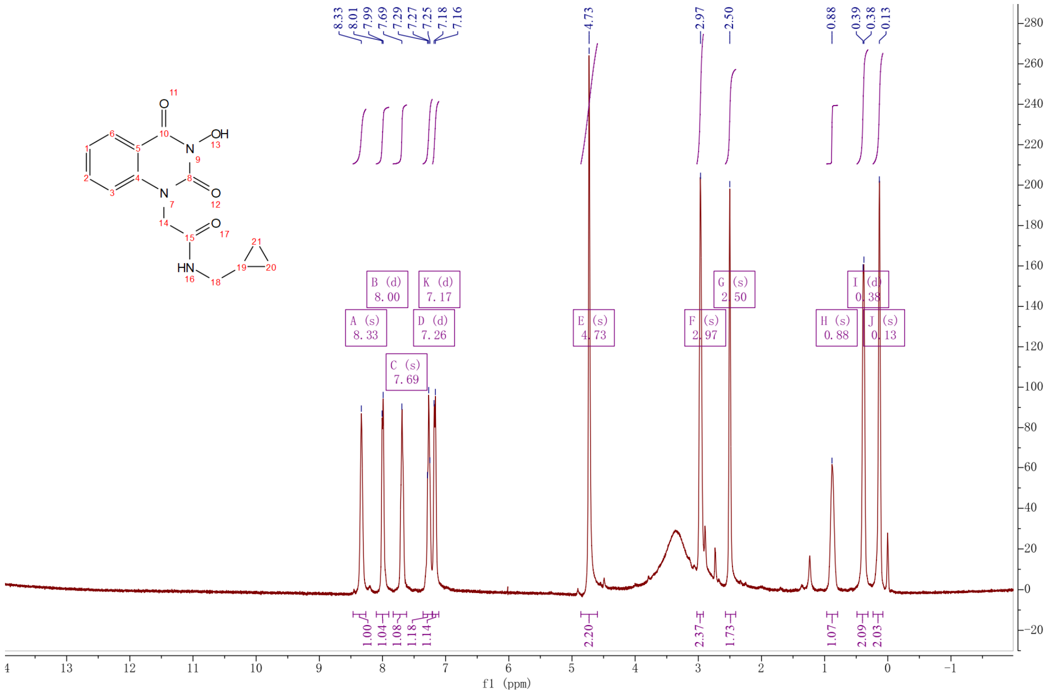

Figure S20. ^{1}H NMR (400 MHz, DMSO-d_6) spectrum of **10g**

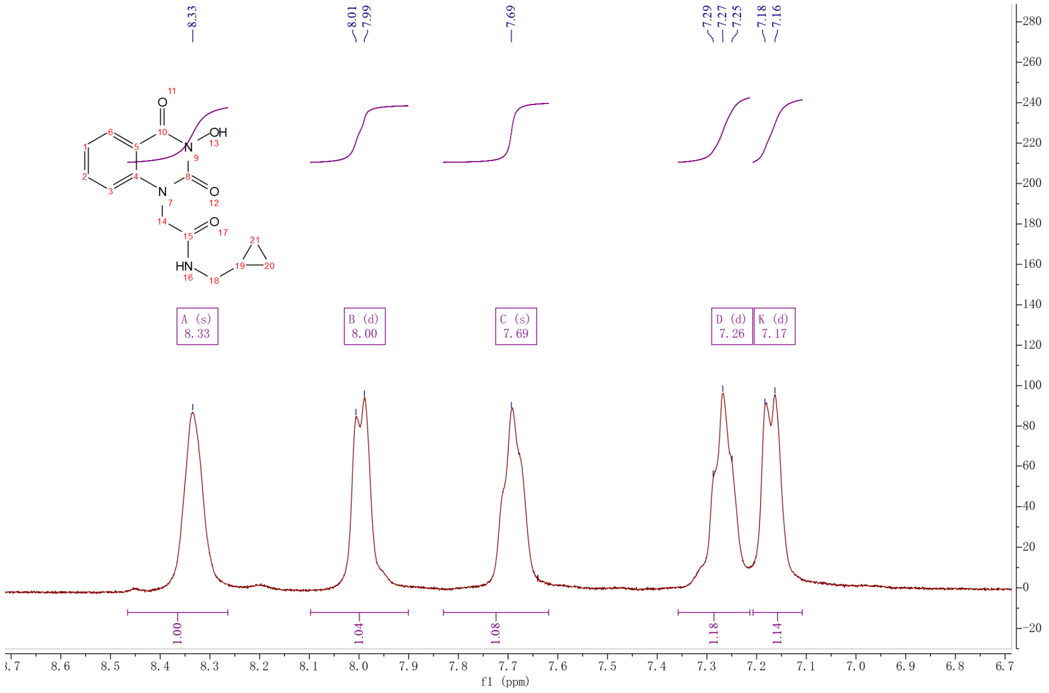

Figure S21. Magnified ^{1}H NMR (400 MHz, DMSO-d_6) spectrum fragments of **10g**

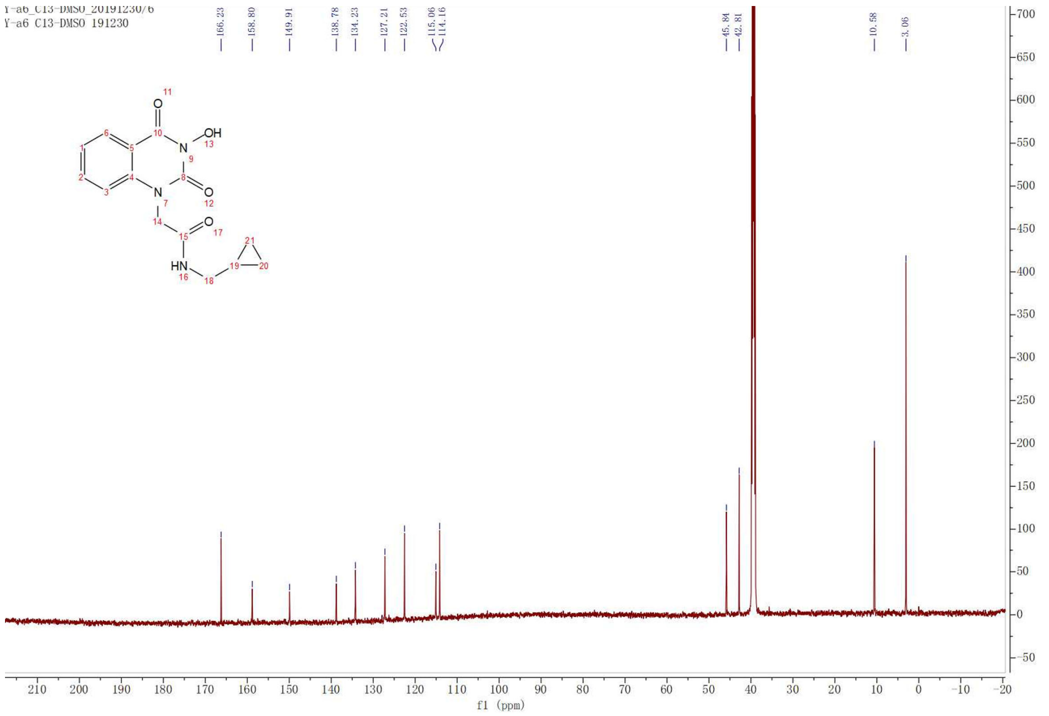

Figure S22. ^{13}C NMR (151 MHz, DMSO-d_6) spectrum of **10g**

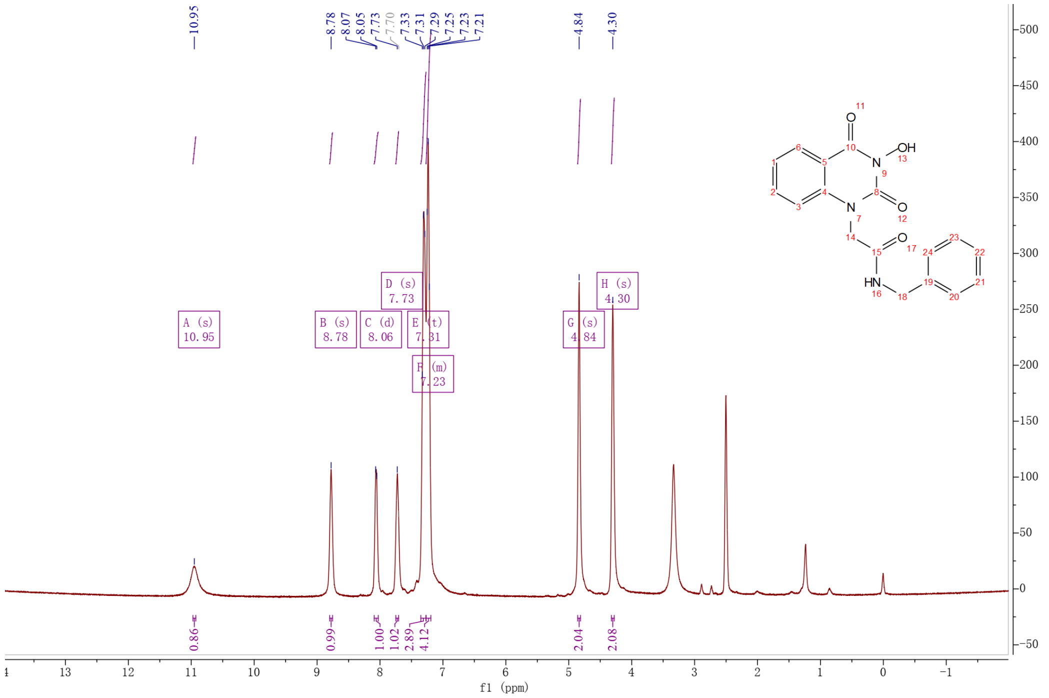

Figure S23. ^{1}H NMR (400 MHz, DMSO-d_6) spectrum of **10h**

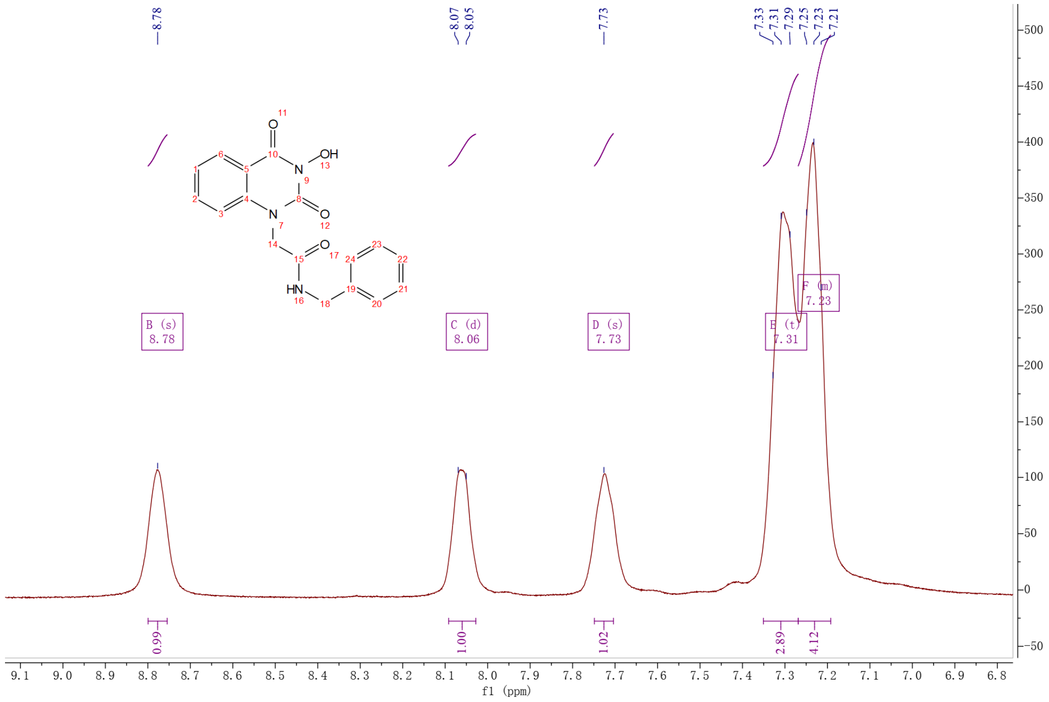

Figure S24. Magnified ^{1}H NMR (400 MHz, DMSO-d_6) spectrum fragments of **10h**

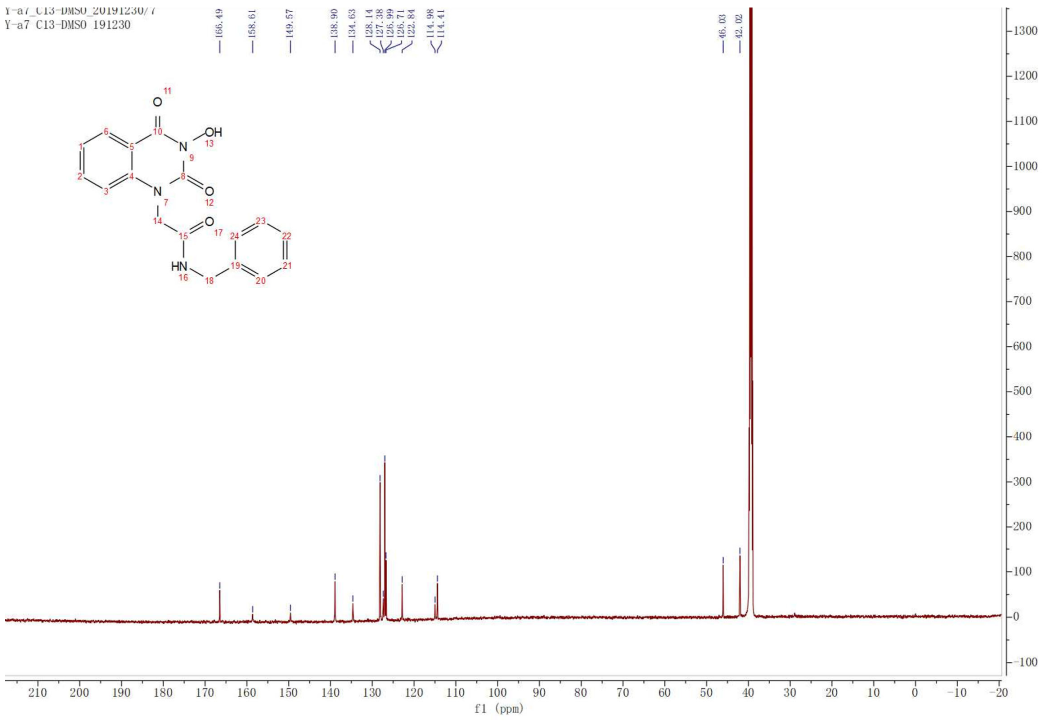

Figure S25. ^{13}C NMR (151 MHz, DMSO-d_6) spectrum of **10h**

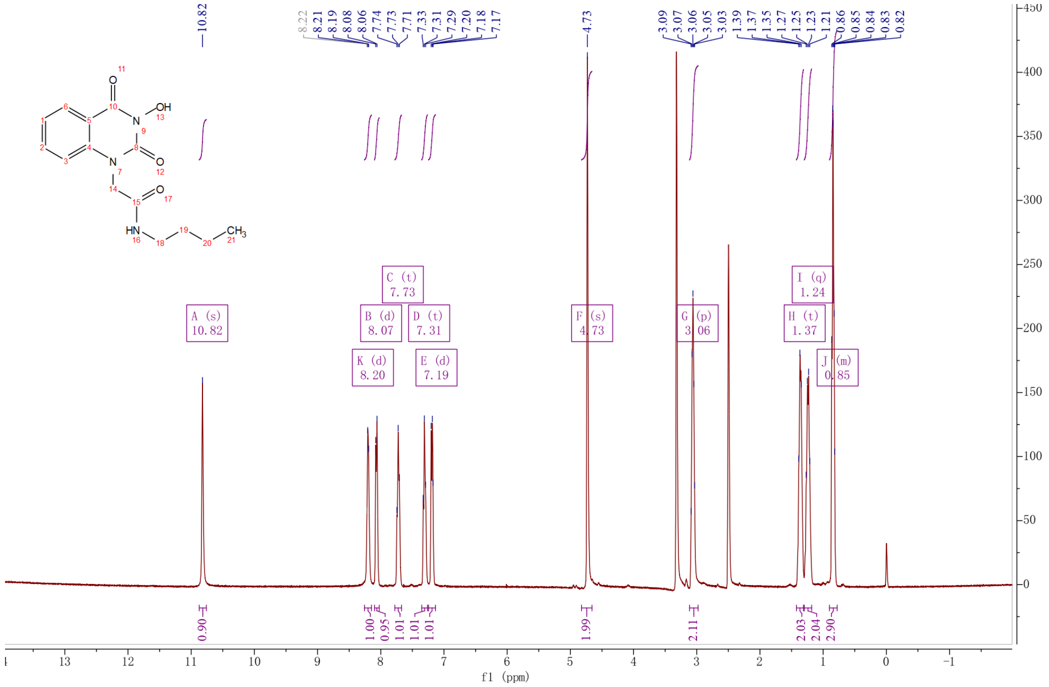

Figure S26. ^{1}H NMR (400 MHz, DMSO-d_6) spectrum of **10i**

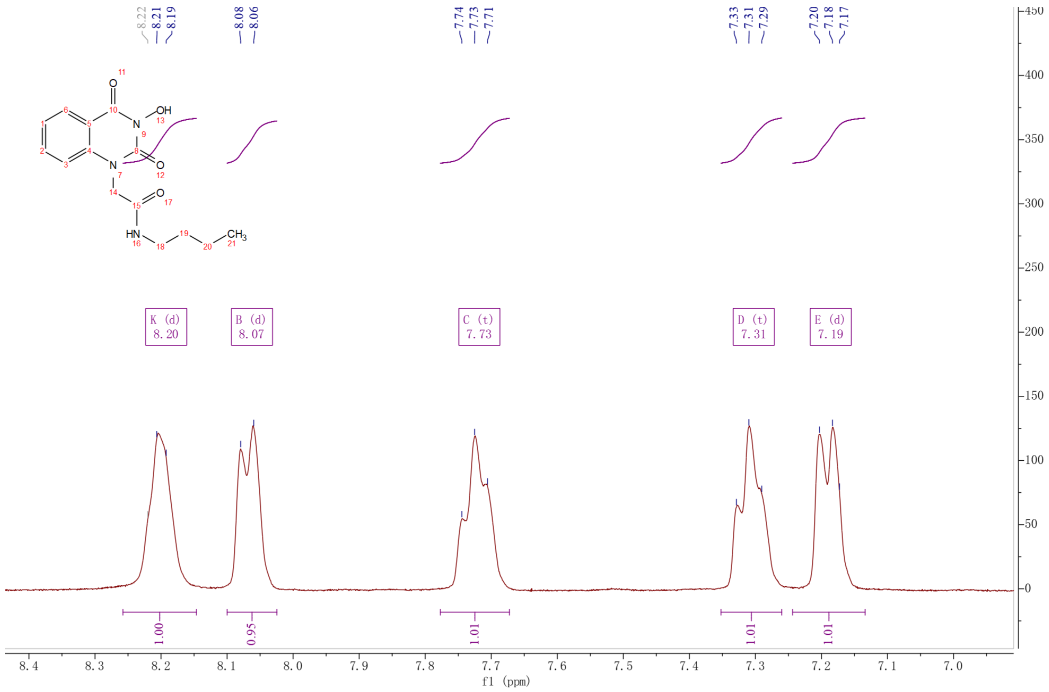

Figure S27. Magnified ^{1}H NMR (400 MHz, DMSO-d_6) spectrum fragments of **10i**

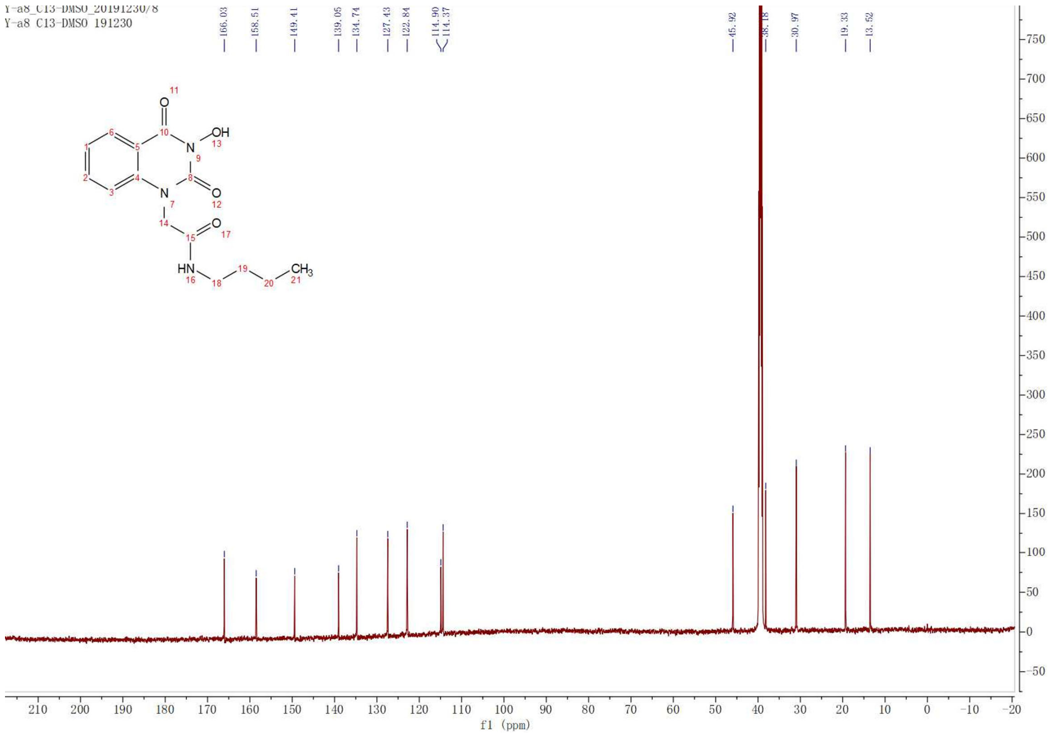

Figure S28. ^{13}C NMR (151 MHz, DMSO-d_6) spectrum of **10i**

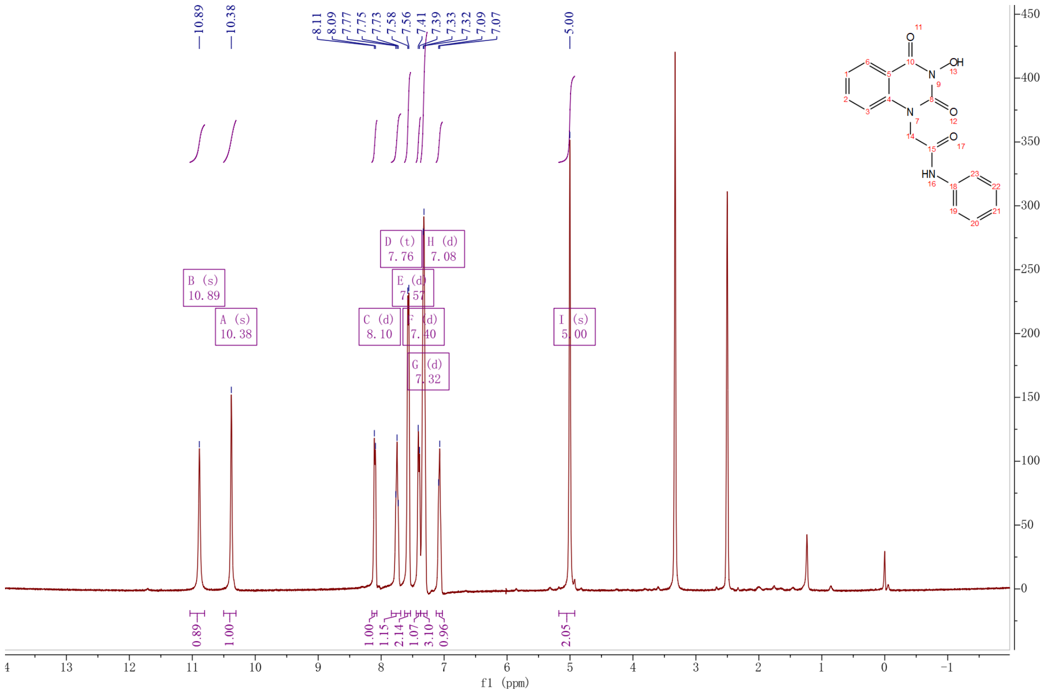

Figure S29. ^{1}H NMR (400 MHz, DMSO-d_6) spectrum of **10j**

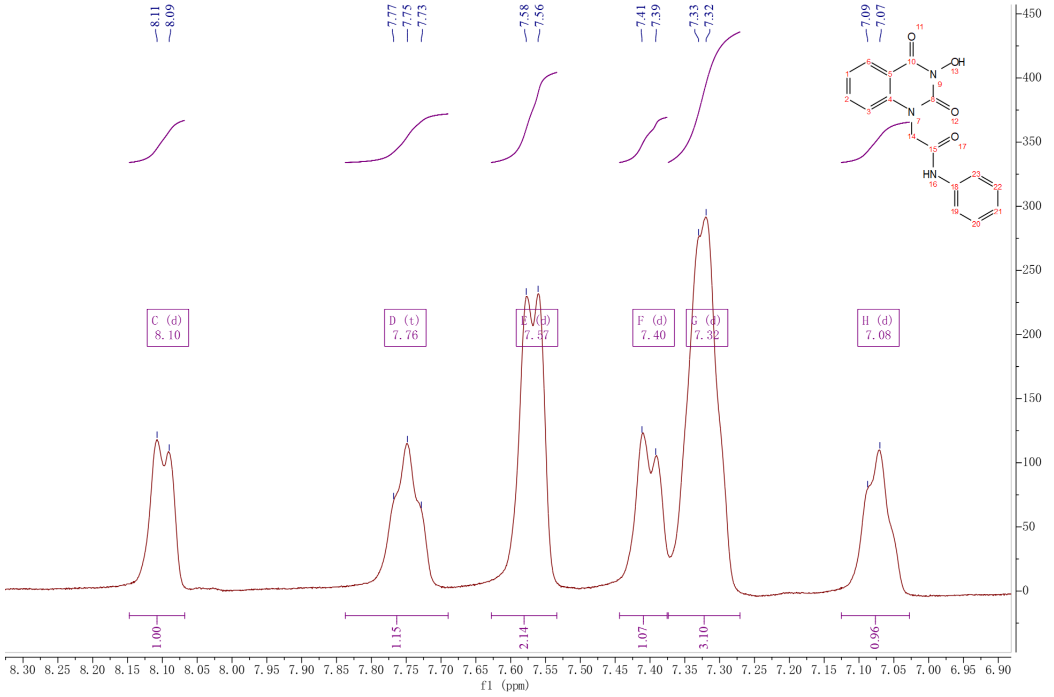

Figure S30. Magnified ^{1}H NMR (400 MHz, DMSO-d_6) spectrum fragments of **10j**

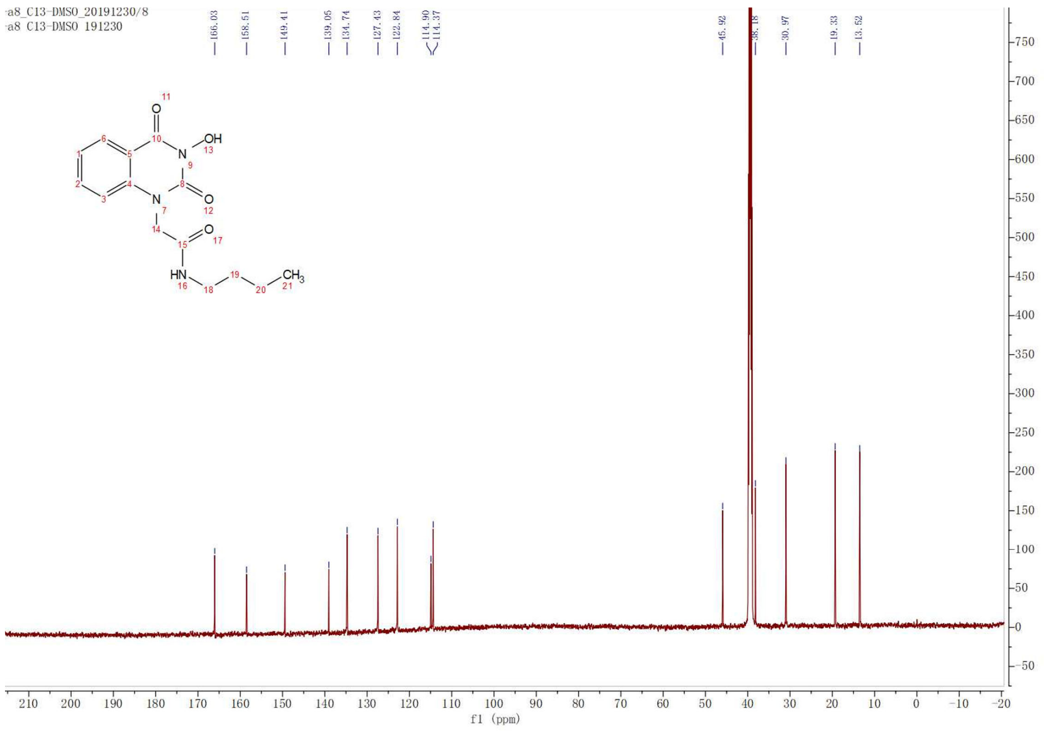

Figure S31. ^{13}C NMR (151 MHz, DMSO-d_6) spectrum of **10j**

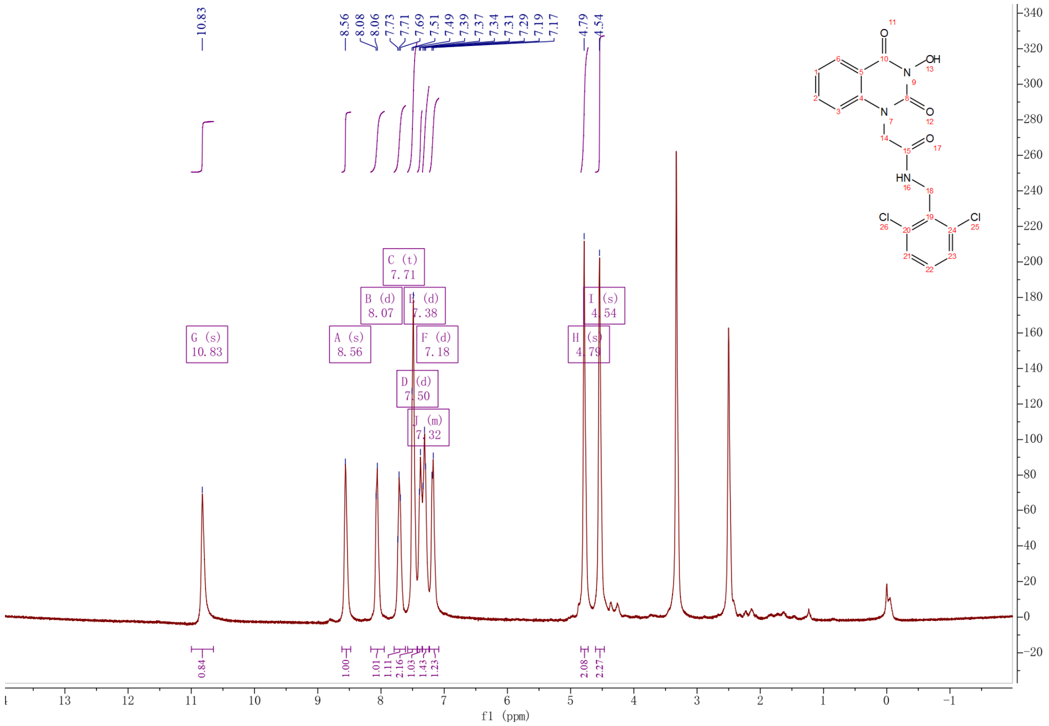

Figure S32. ^{1}H NMR (400 MHz, DMSO-d_6) spectrum of **10k**

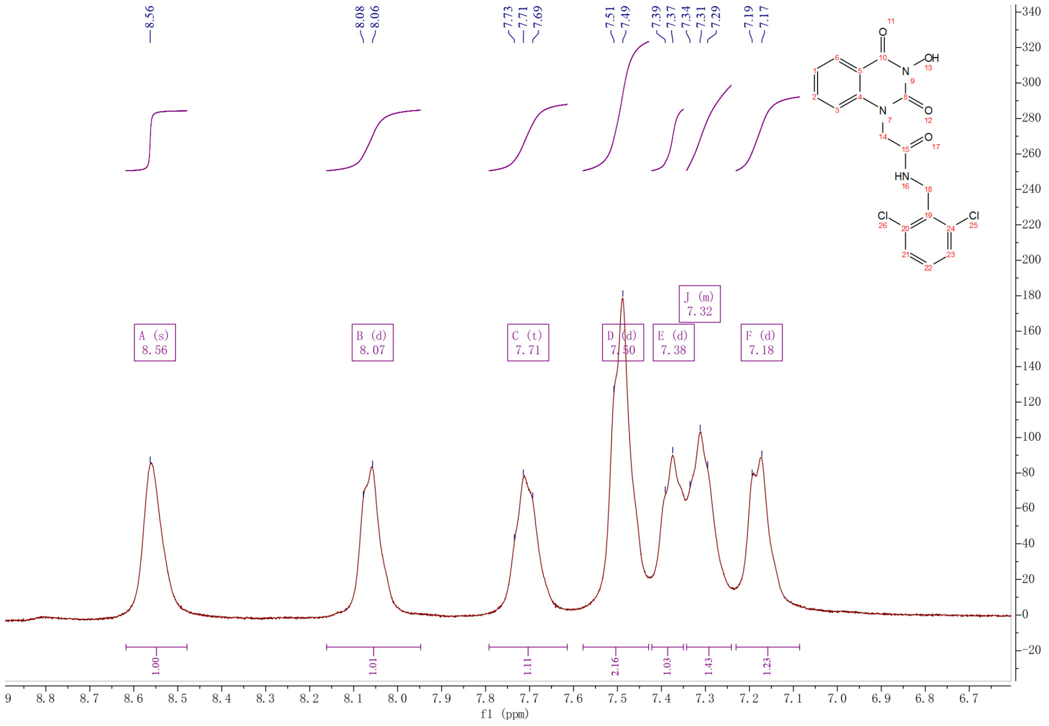

Figure S33. Magnified ^{1}H NMR (400 MHz, DMSO-d_6) spectrum fragments of **10k**

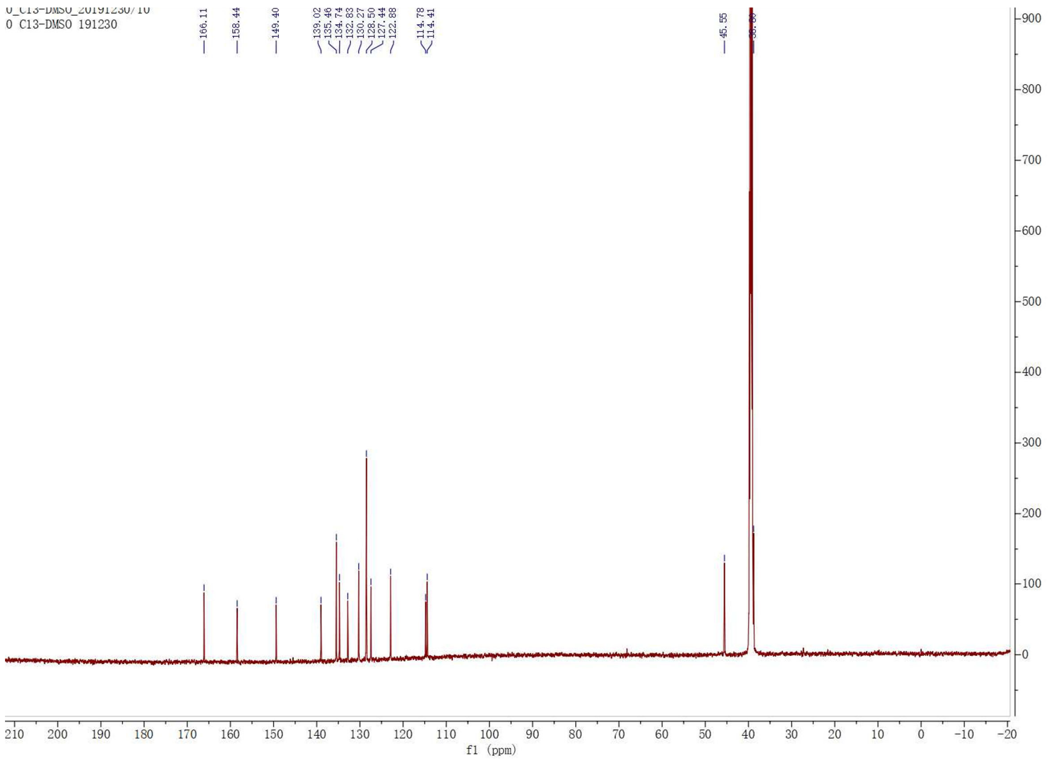

Figure S34. ^{13}C NMR (151 MHz, DMSO-d_6) spectrum of **10k**

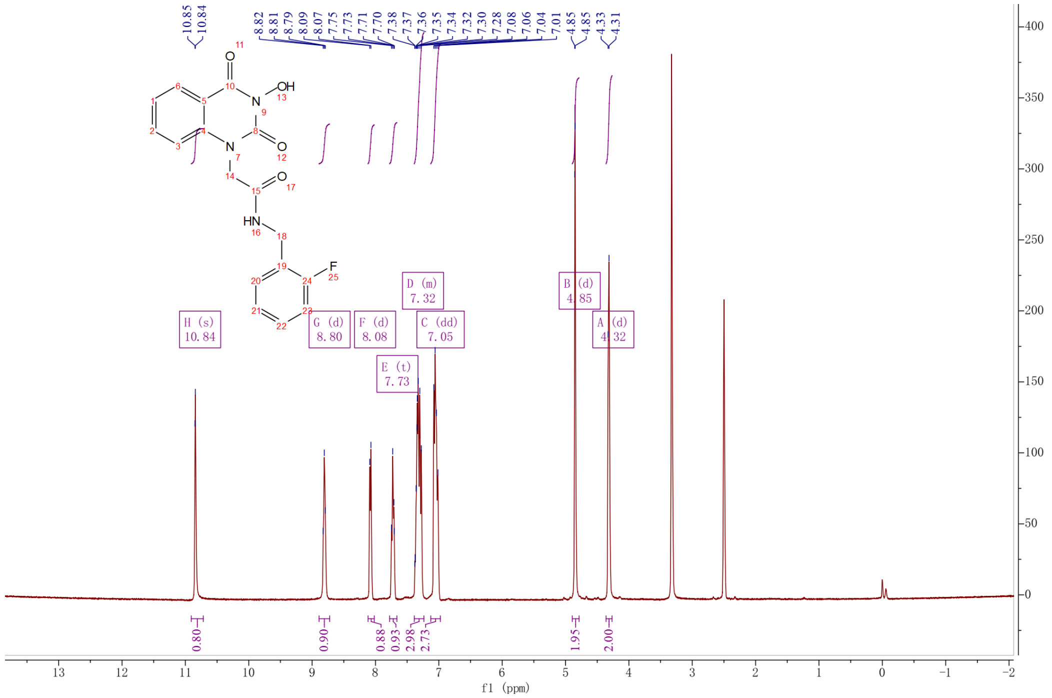

Figure S35. ^{1}H NMR (400 MHz, DMSO-d_6) spectrum of **10l**

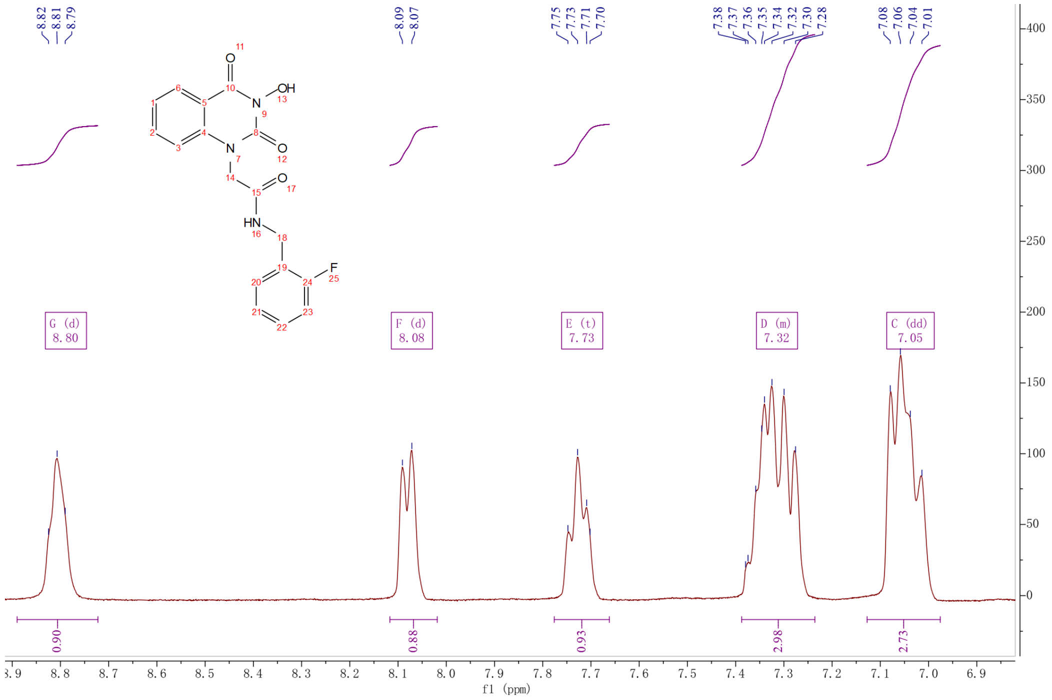

Figure S36. Magnified ^{1}H NMR (400 MHz, DMSO-d_6) spectrum fragments of **10l**

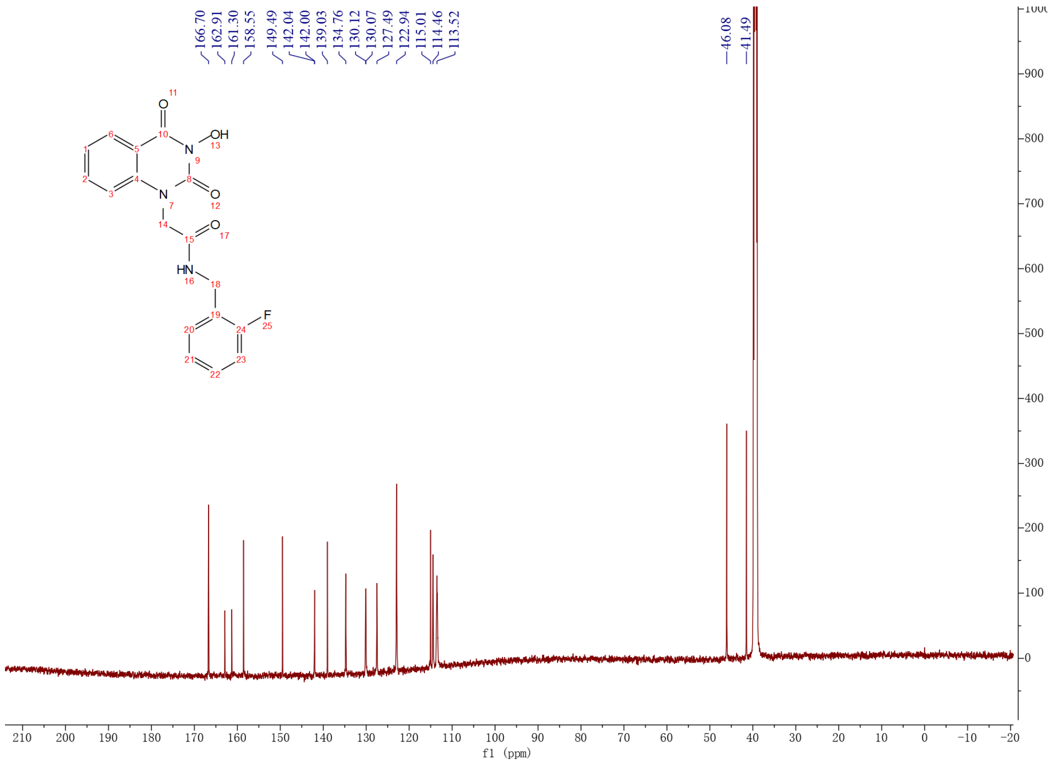

Figure S37. ^{13}C NMR (151 MHz, DMSO-d_6) spectrum of **10l**

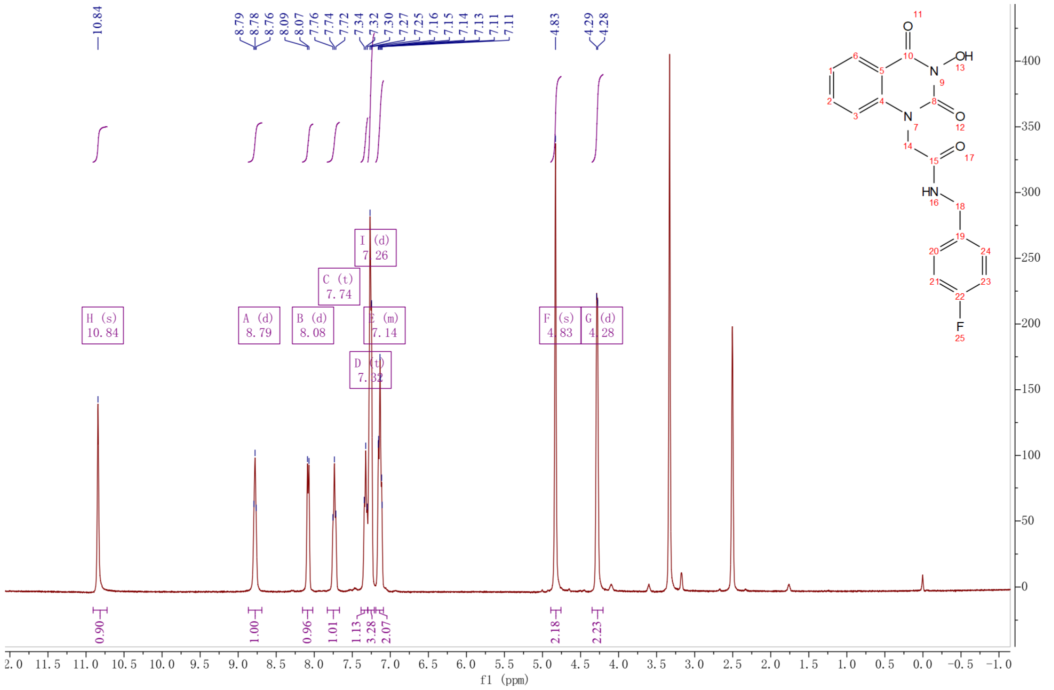

Figure S38. ^{1}H NMR (400 MHz, DMSO-d_6) spectrum of **10m**

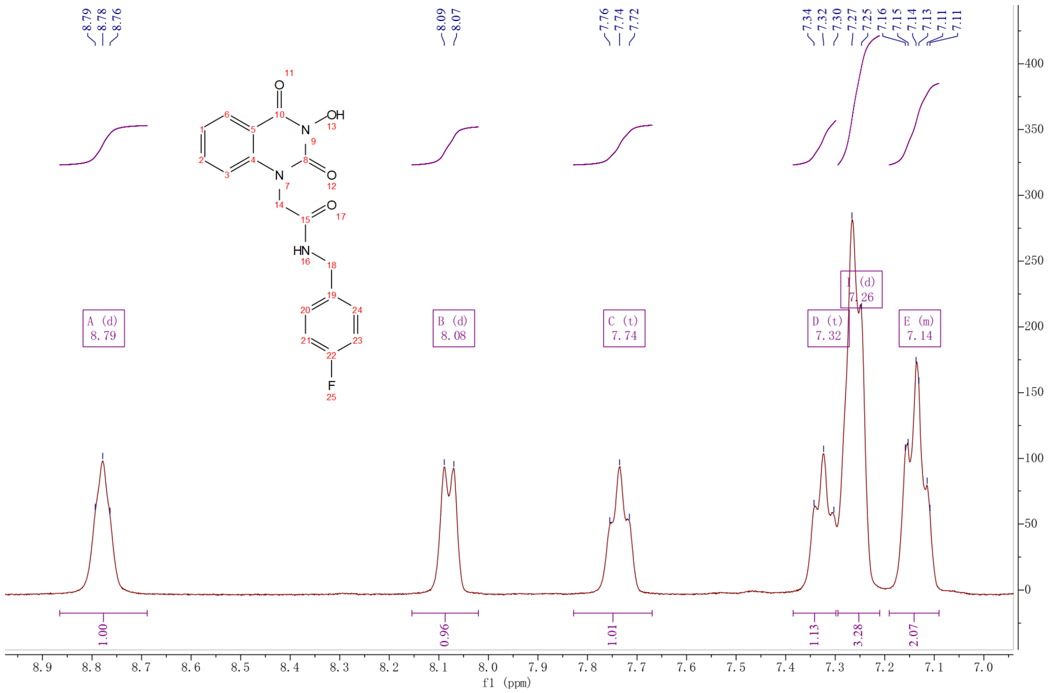

Figure S39. Magnified ^{1}H NMR (400 MHz, DMSO-d_6) spectrum fragments of **10m**

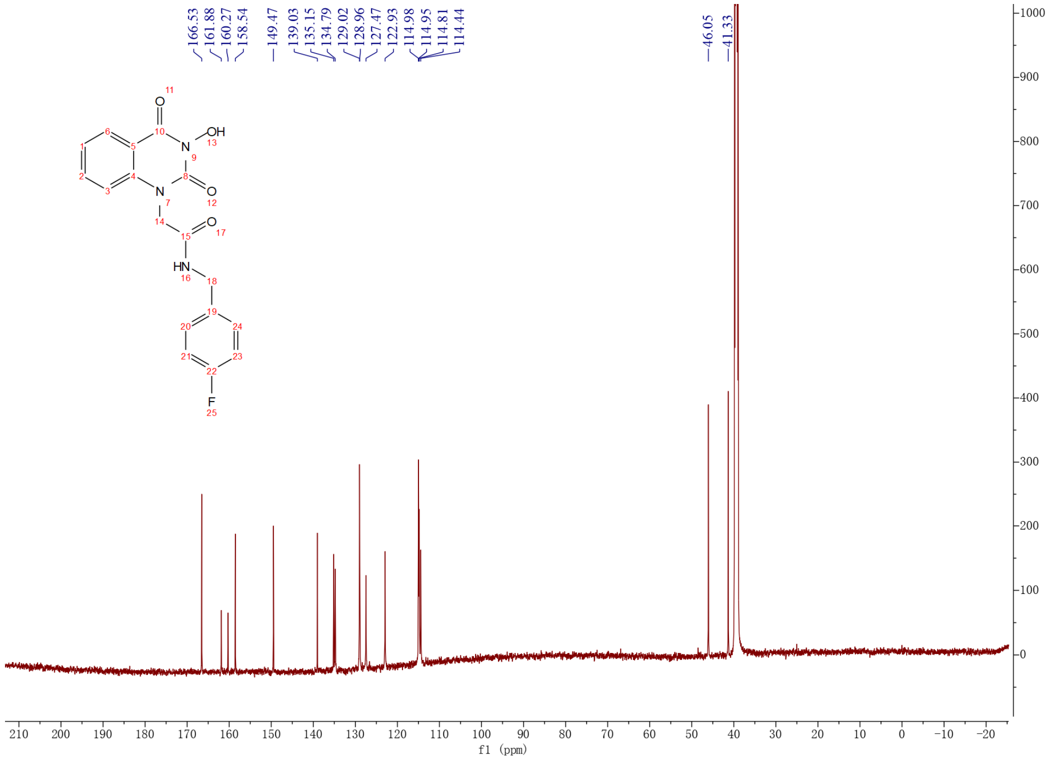

Figure S40. ^{13}C NMR (151 MHz, DMSO-d_6) spectrum of **10m**

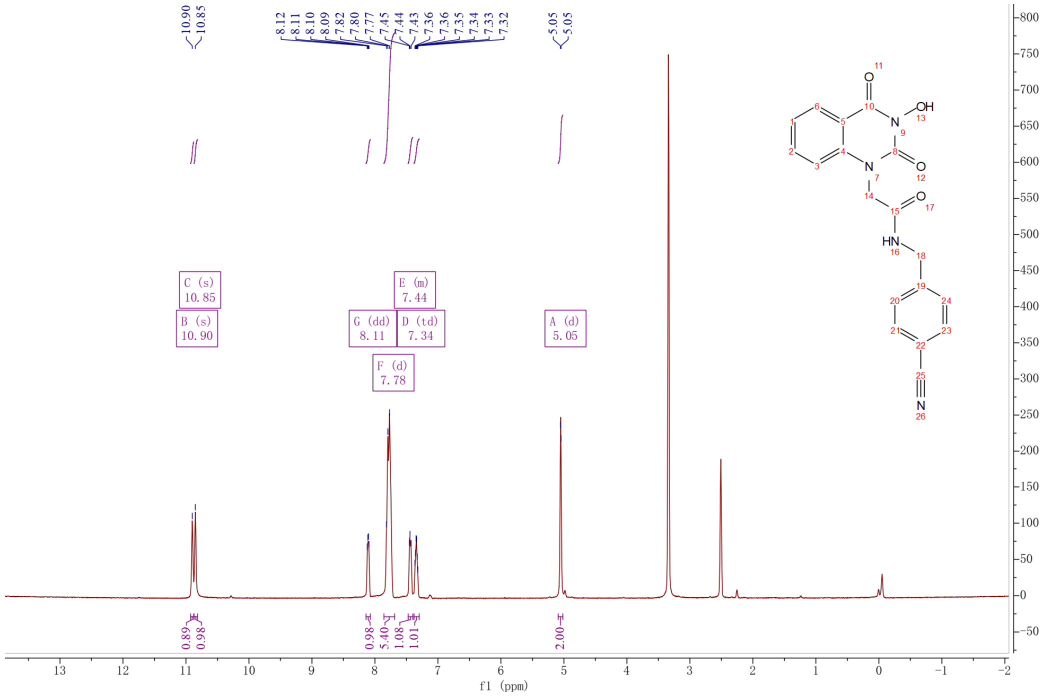

Figure S41. ^{1}H NMR (400 MHz, DMSO-d_6) spectrum of **10n**

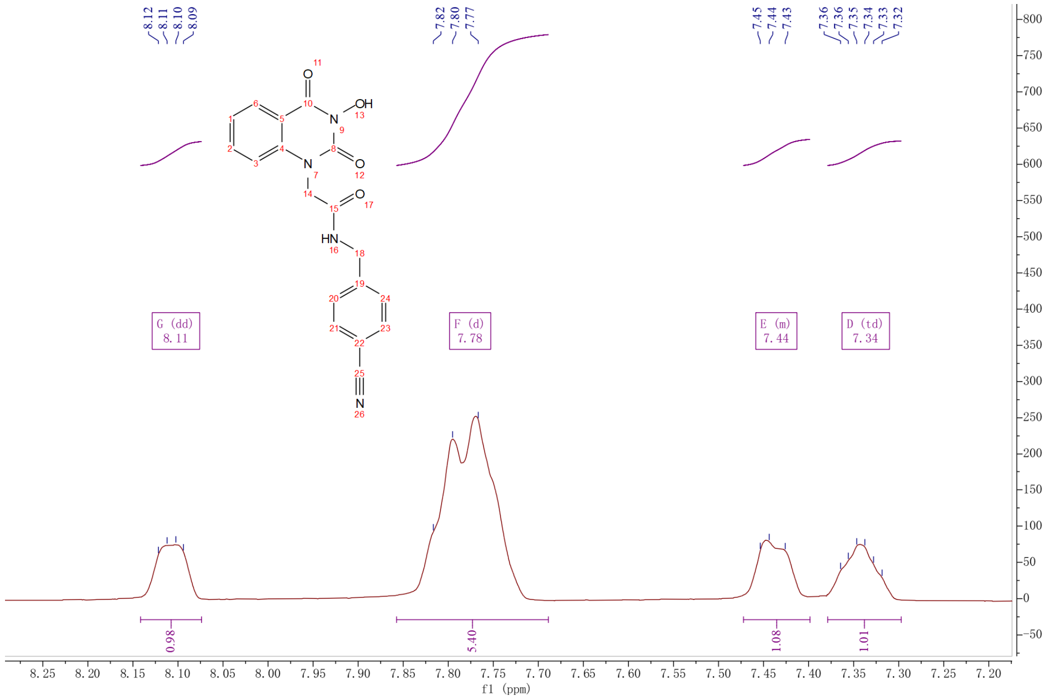

Figure S42. Magnified ^{1}H NMR (400 MHz, DMSO-d_6) spectrum fragments of **10n**

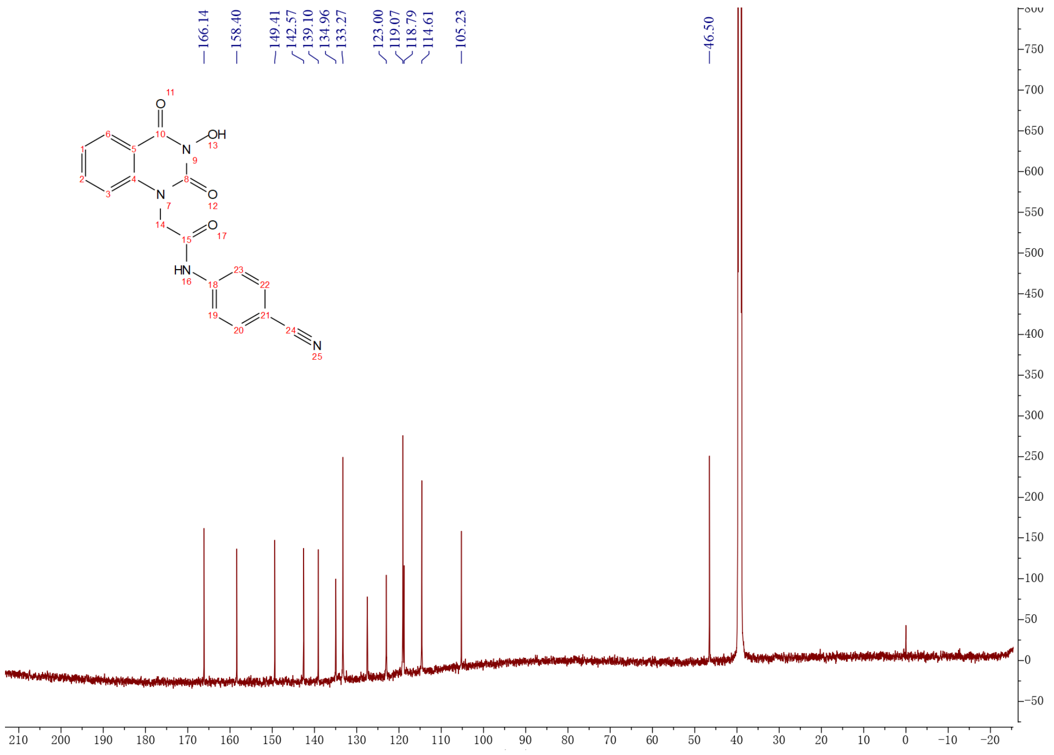

Figure S43. ^{13}C NMR (151 MHz, DMSO-d_6) spectrum of **10n**

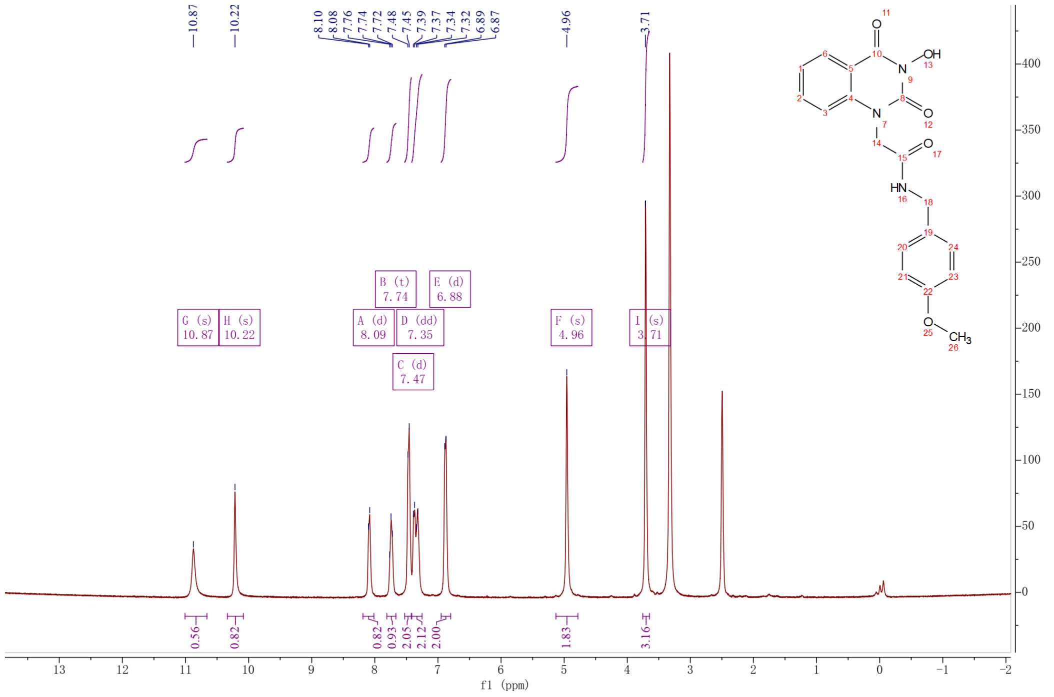

Figure S44. ^{1}H NMR (400 MHz, DMSO-d_6) spectrum of **10o**

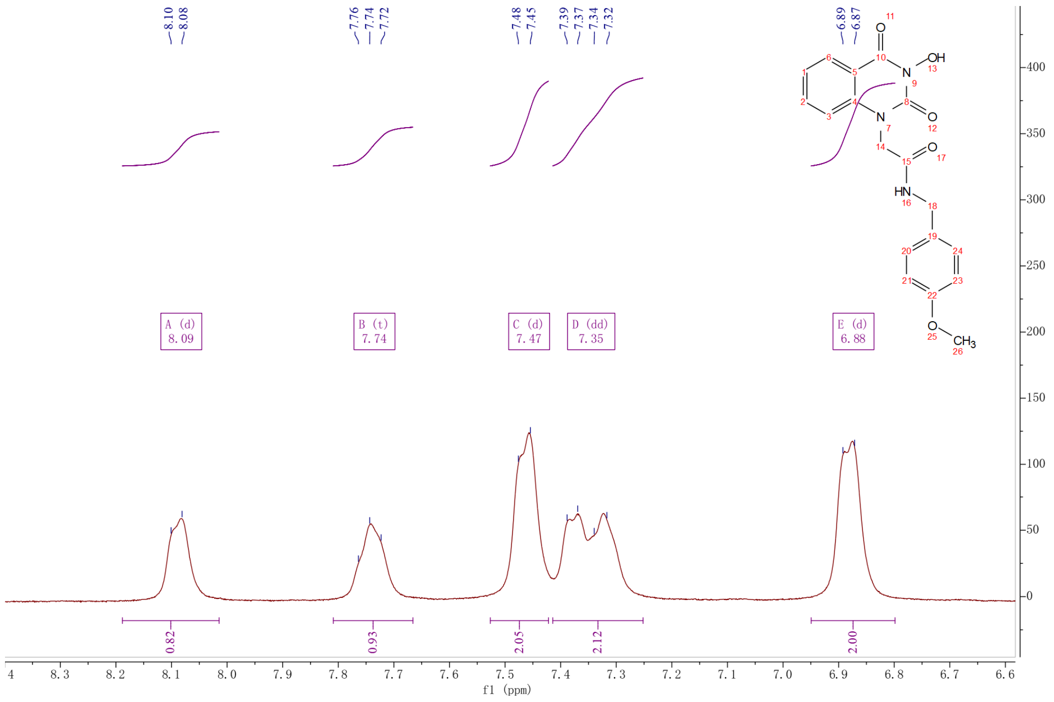

Figure S45. Magnified ^{1}H NMR (400 MHz, DMSO-d_6) spectrum fragments of **10o**

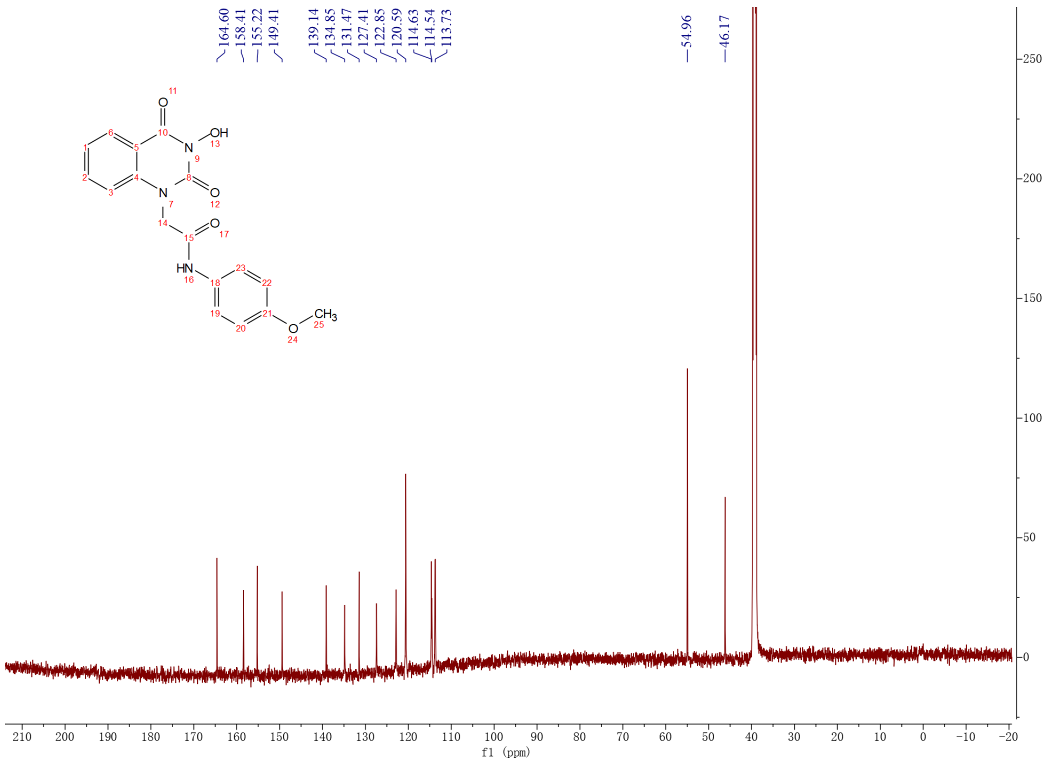

Figure S46. ^{13}C NMR (151 MHz, DMSO-d_6) spectrum of **10o**

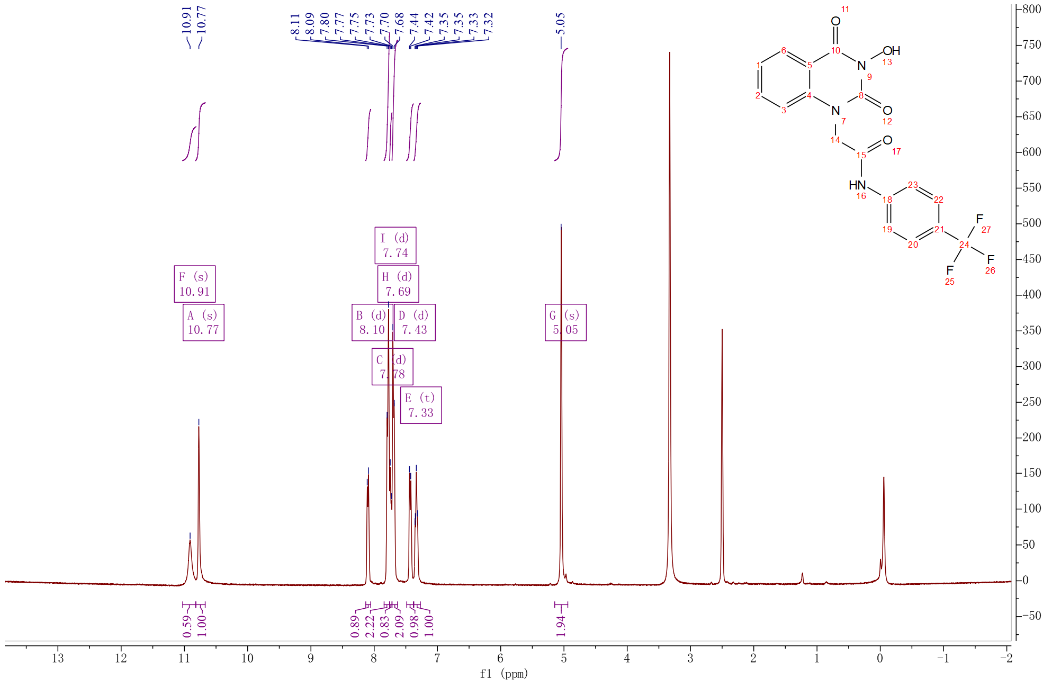

Figure S47. ^{1}H NMR (400 MHz, DMSO-d_6) spectrum of **10p**

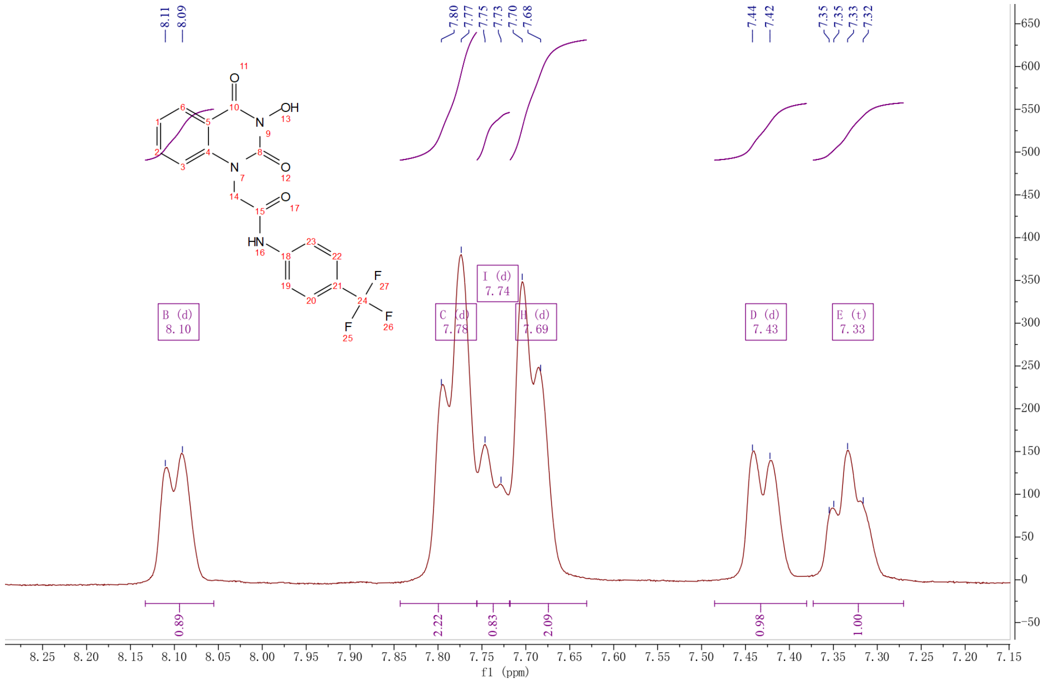

Figure S48. Magnified ^{1}H NMR (400 MHz, DMSO-d_6) spectrum fragments of **10p**

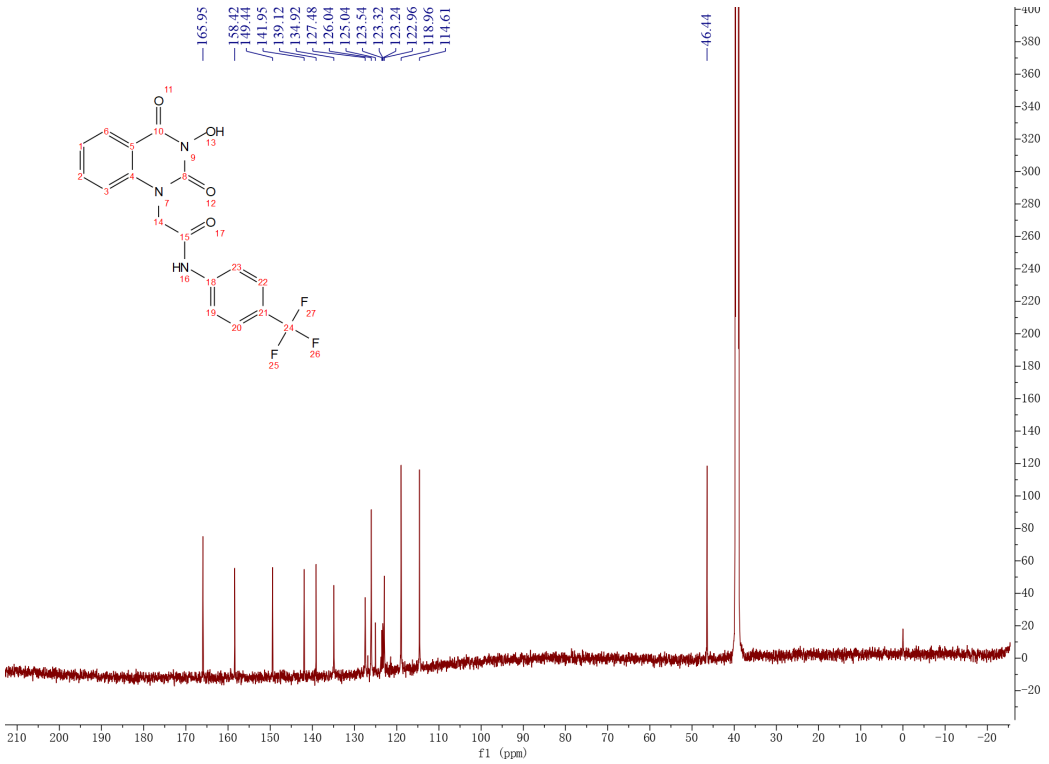

Figure S49. ^{13}C NMR (151 MHz, DMSO-d_6) spectrum of **10p**

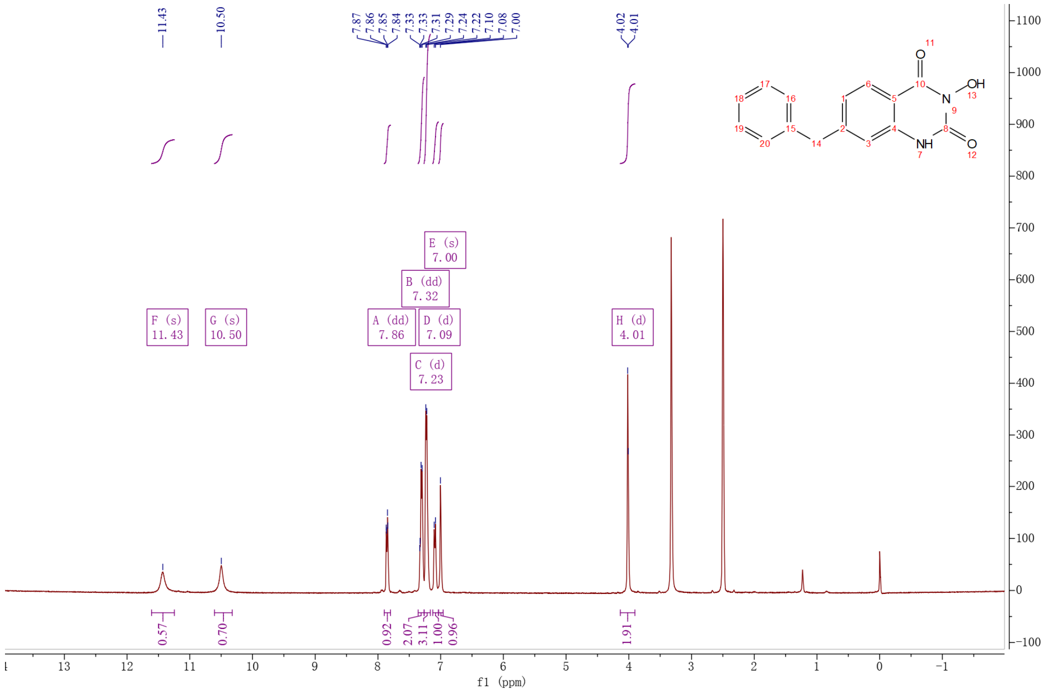

Figure S50. ^{1}H NMR (400 MHz, DMSO-d_6) spectrum of **18a**

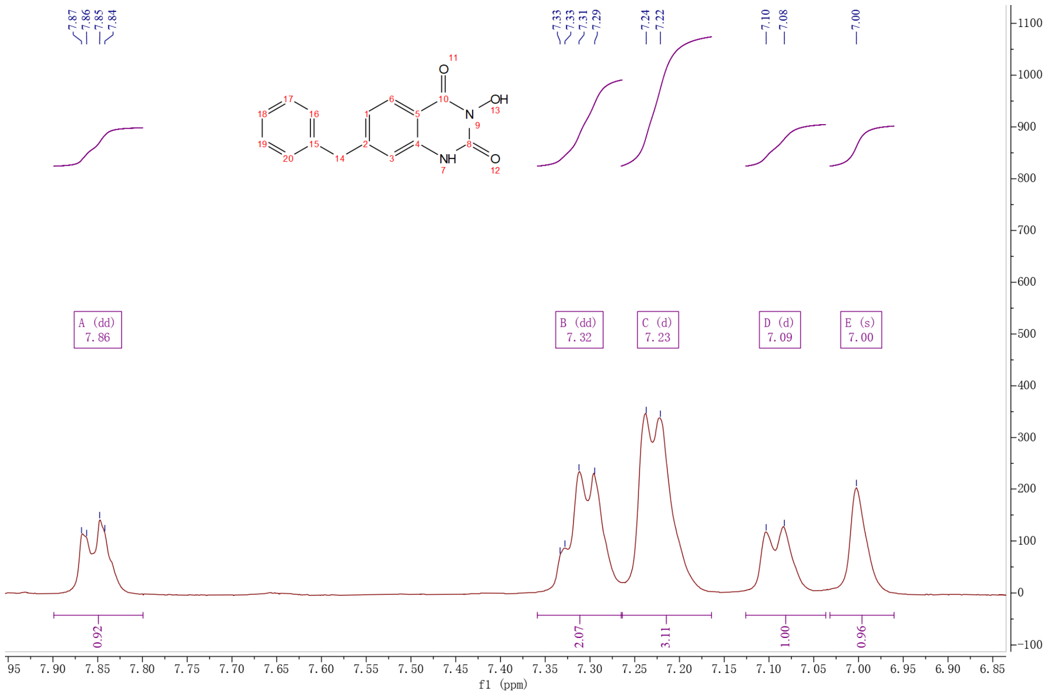

Figure S51. Magnified ^{1}H NMR (400 MHz, DMSO-d_6) spectrum fragments of **18a**

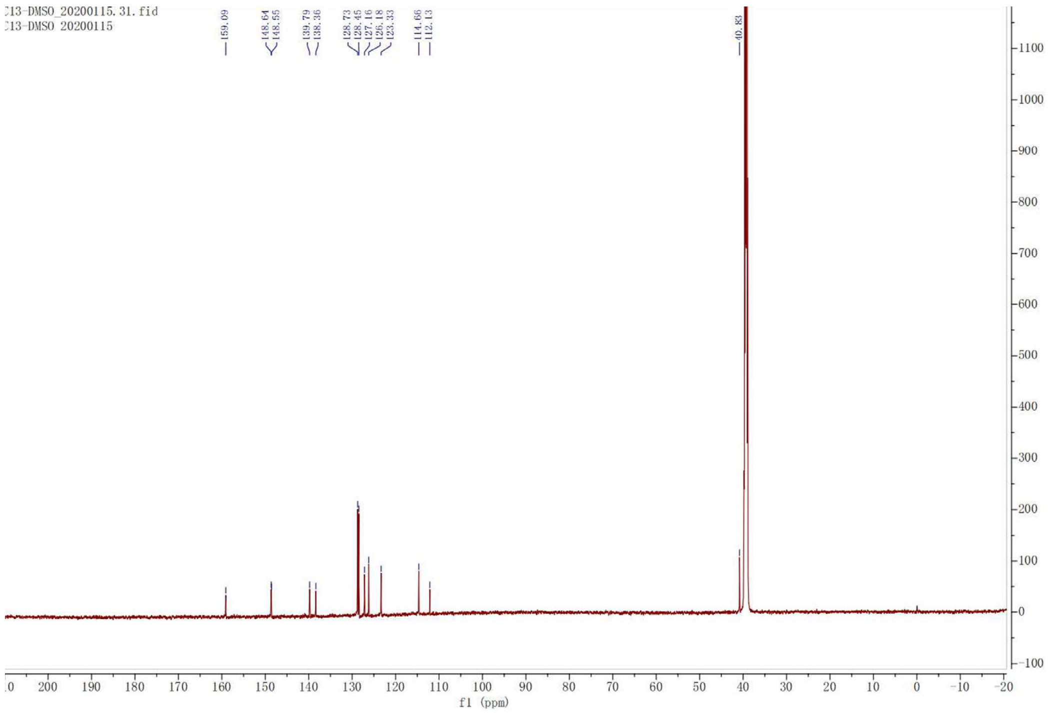

Figure S52. ^{13}C NMR (151 MHz, DMSO-d_6) spectrum of **18a**

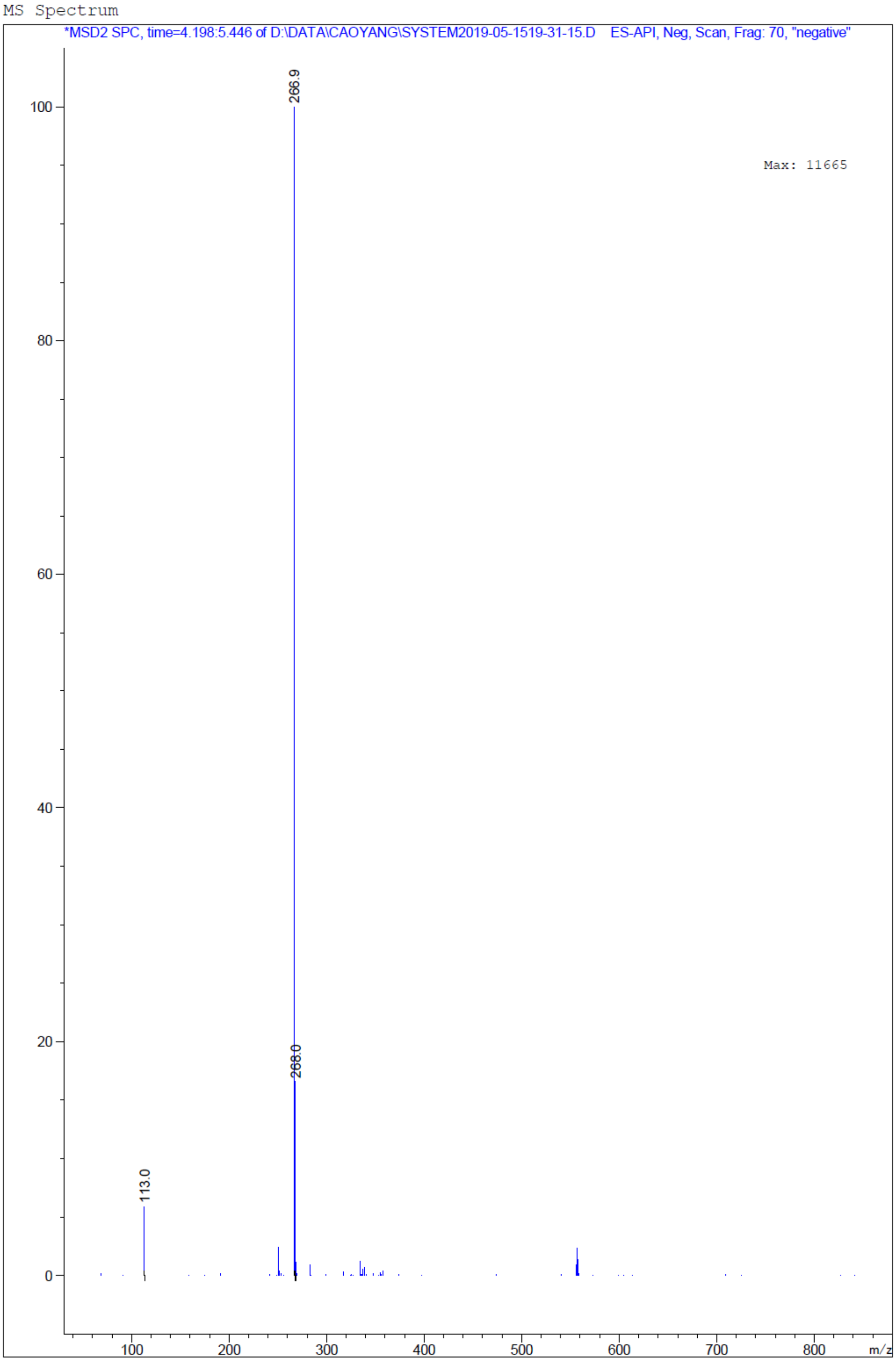

Figure S53. Mass spectrum (negative ionization) of **18a**

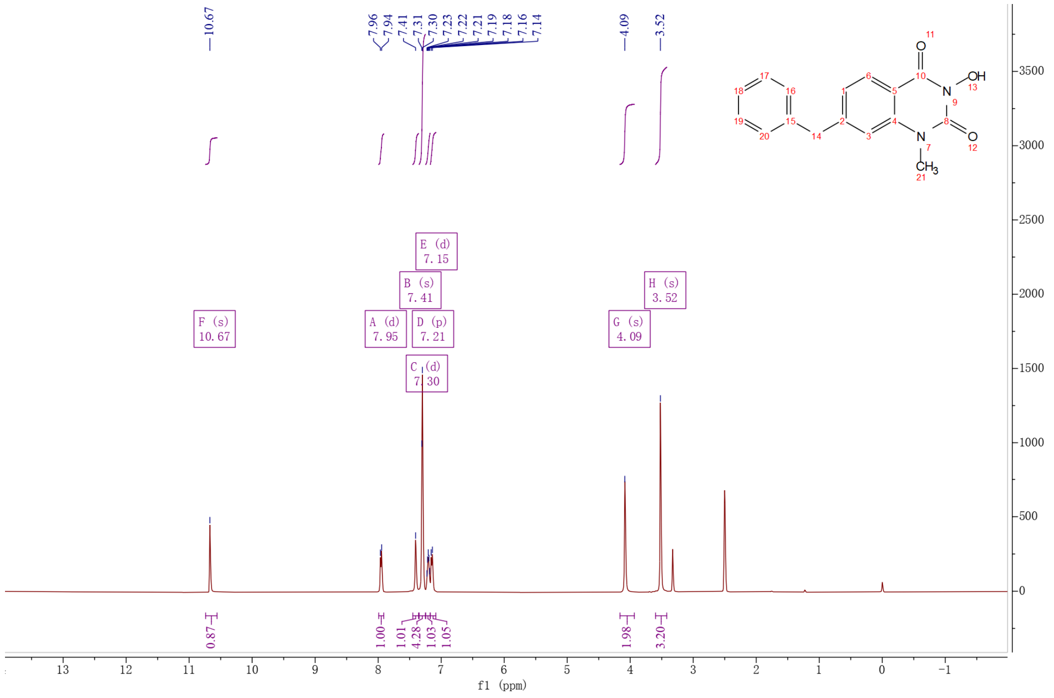

Figure S54. ^{1}H NMR (400 MHz, DMSO-d_6) spectrum of **18b**

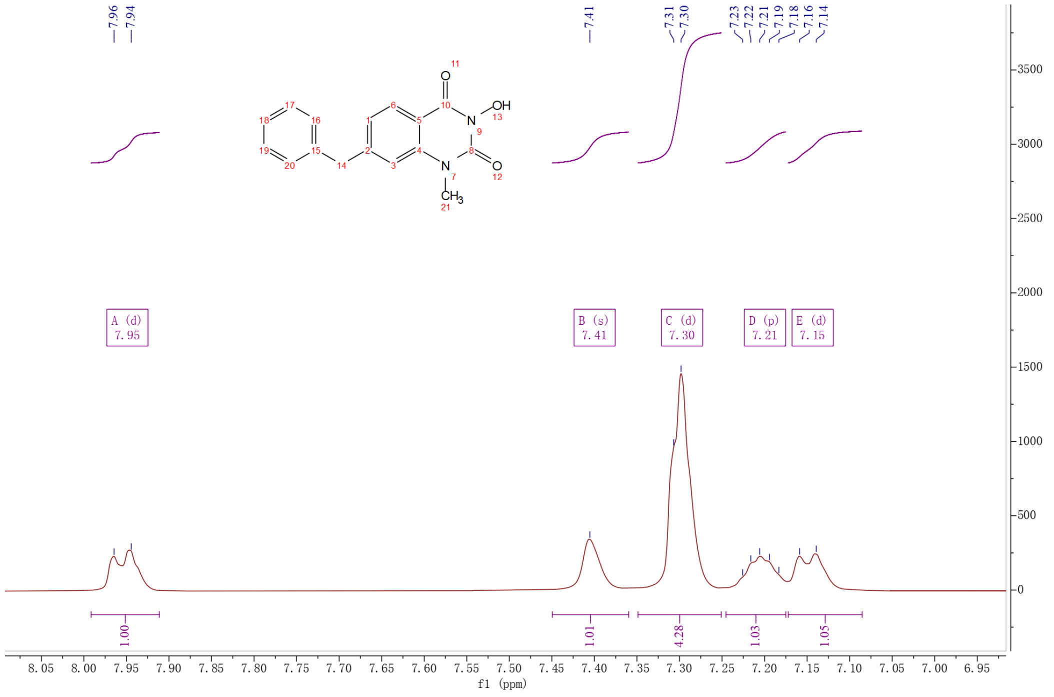

Figure S55. Magnified ^{1}H NMR (400 MHz, DMSO-d_6) spectrum fragments of **18b**

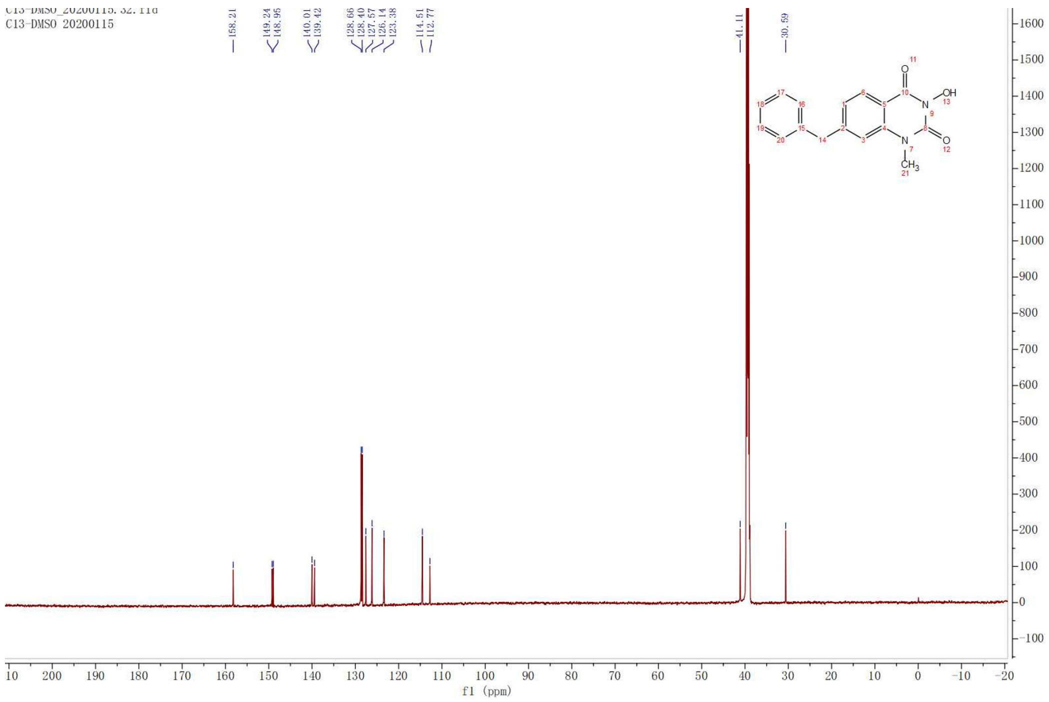

Figure S56. ^{13}C NMR (151 MHz, DMSO-d_6) spectrum of **18b**

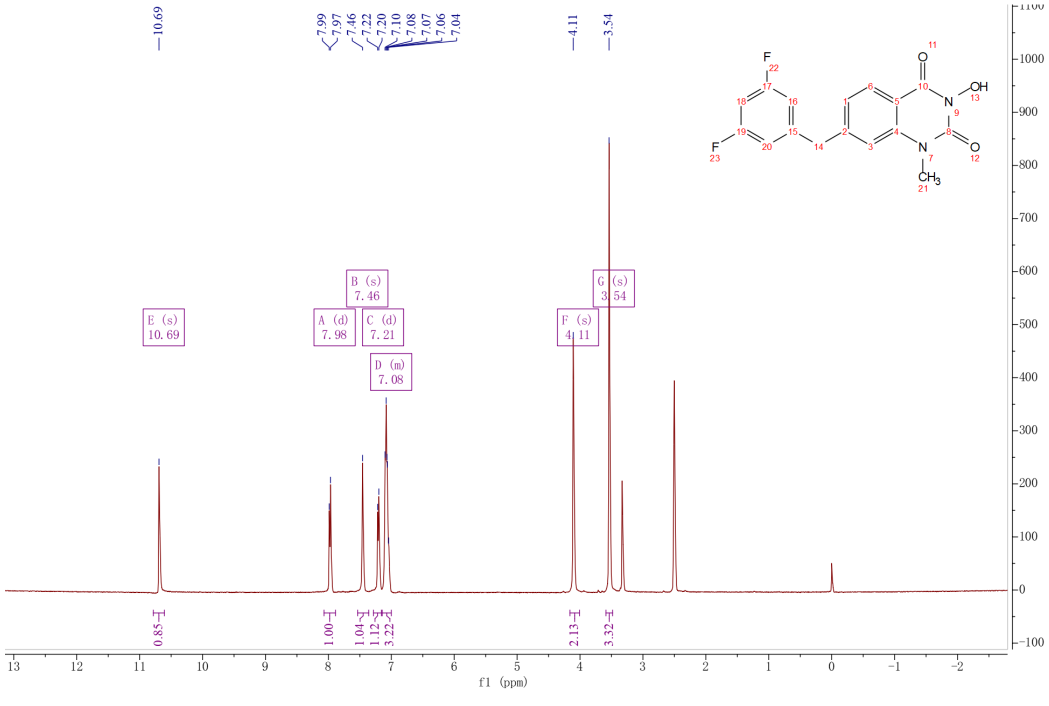

Figure S57. ^{1}H NMR (400 MHz, DMSO-d_6) spectrum of **18c**

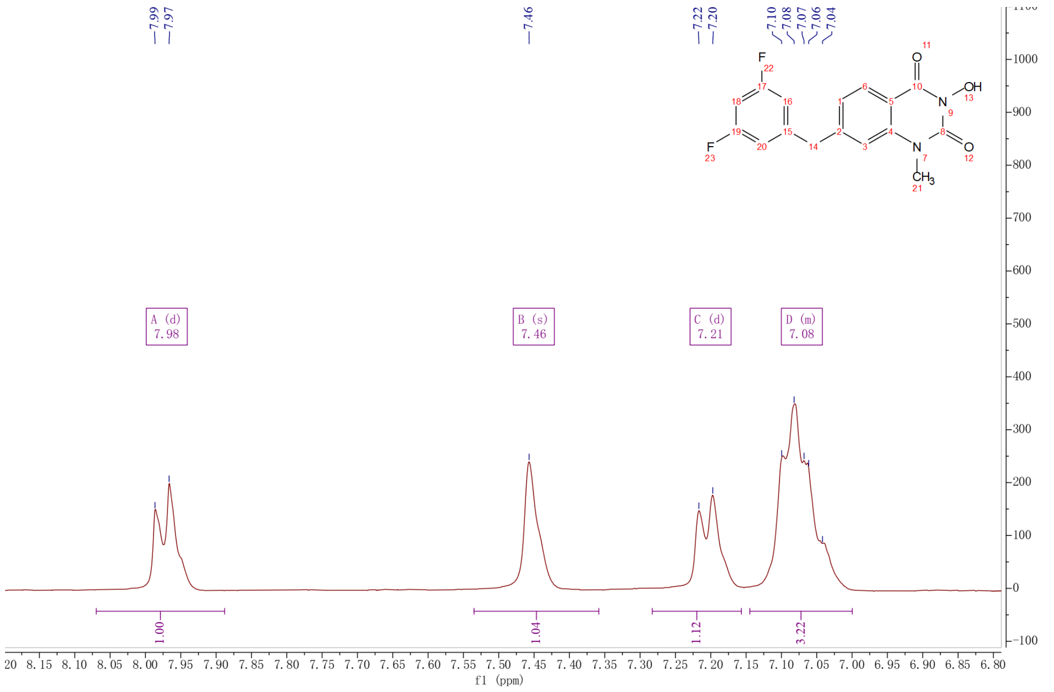

Figure S58. Magnified ^{1}H NMR (400 MHz, DMSO-d_6) spectrum fragments of **18c**

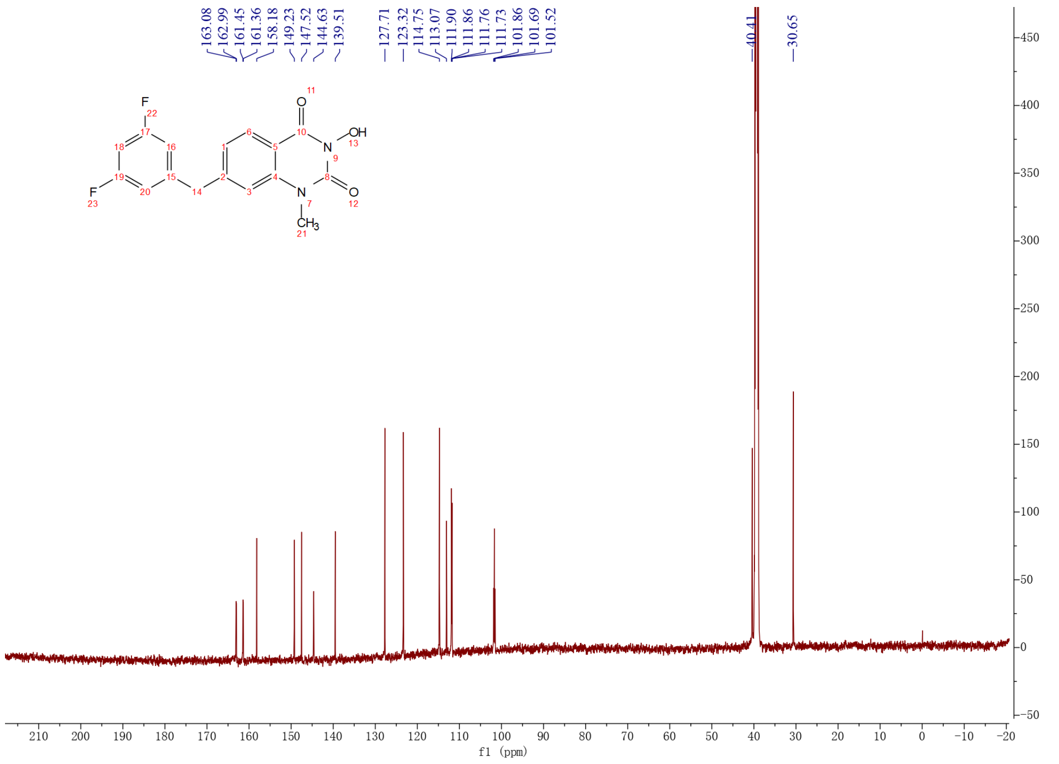

Figure S59. ^{13}C NMR (151 MHz, DMSO-d_6) spectrum of **18c**

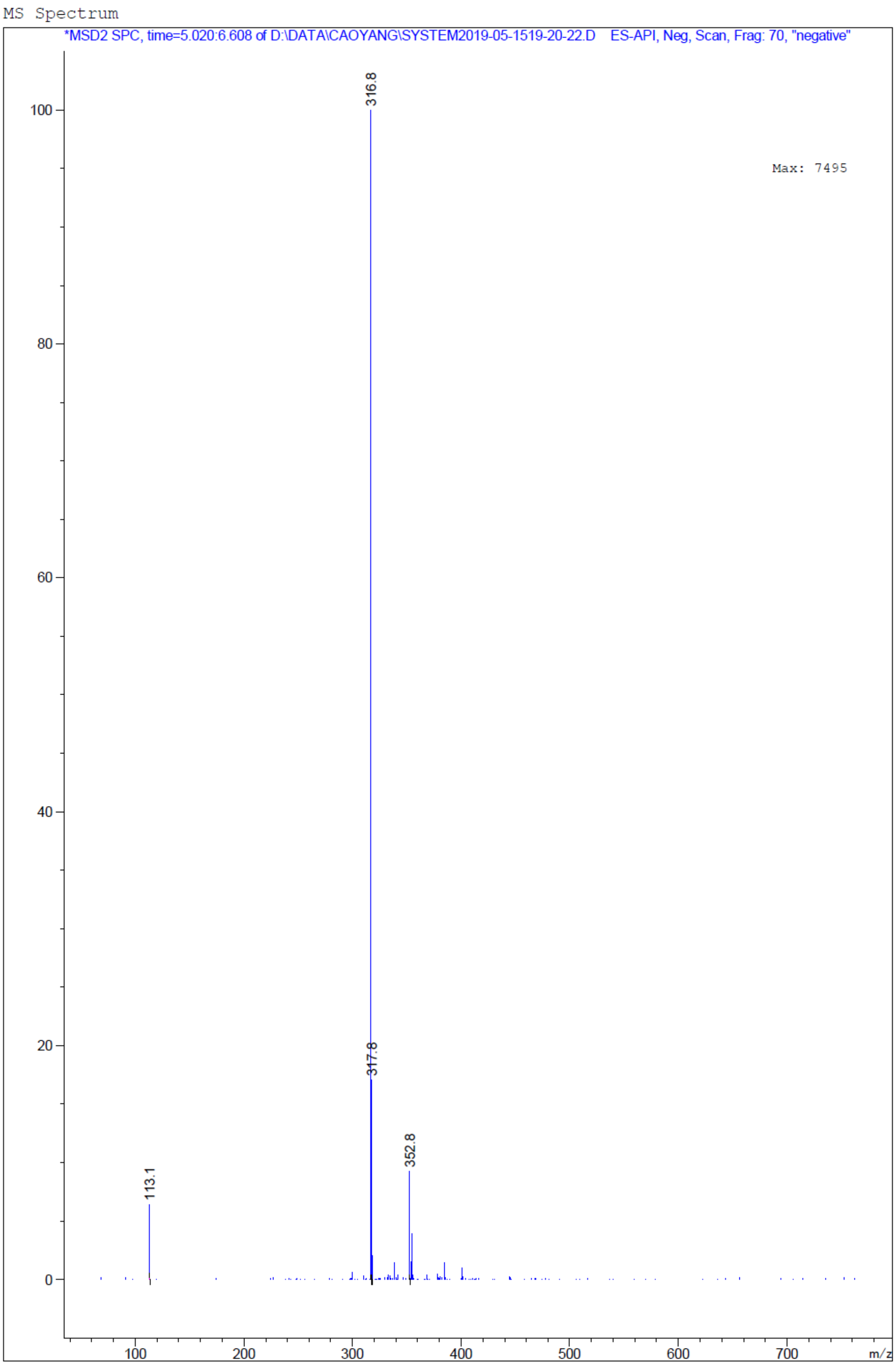

Figure S60. Mass spectrum (negative ionization) of **18c**

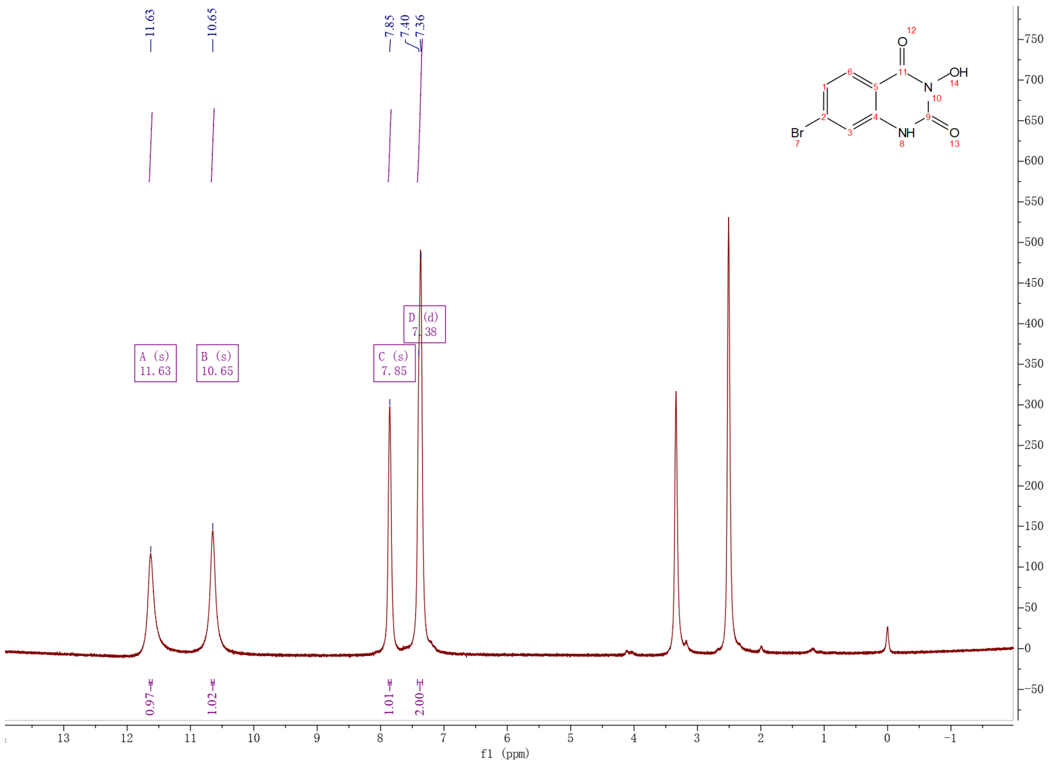

Figure S61. ^{1}H NMR (400 MHz, DMSO-d_6) spectrum of **19**

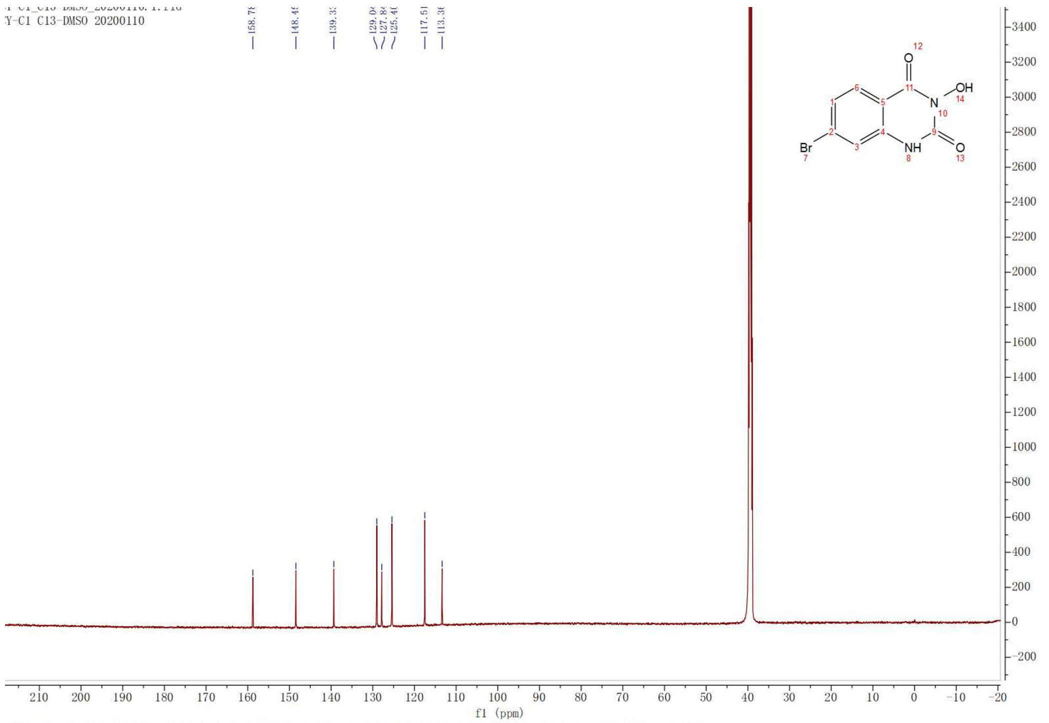

Figure S62. ^{13}C NMR (151 MHz, DMSO-d_6) spectrum of **19**

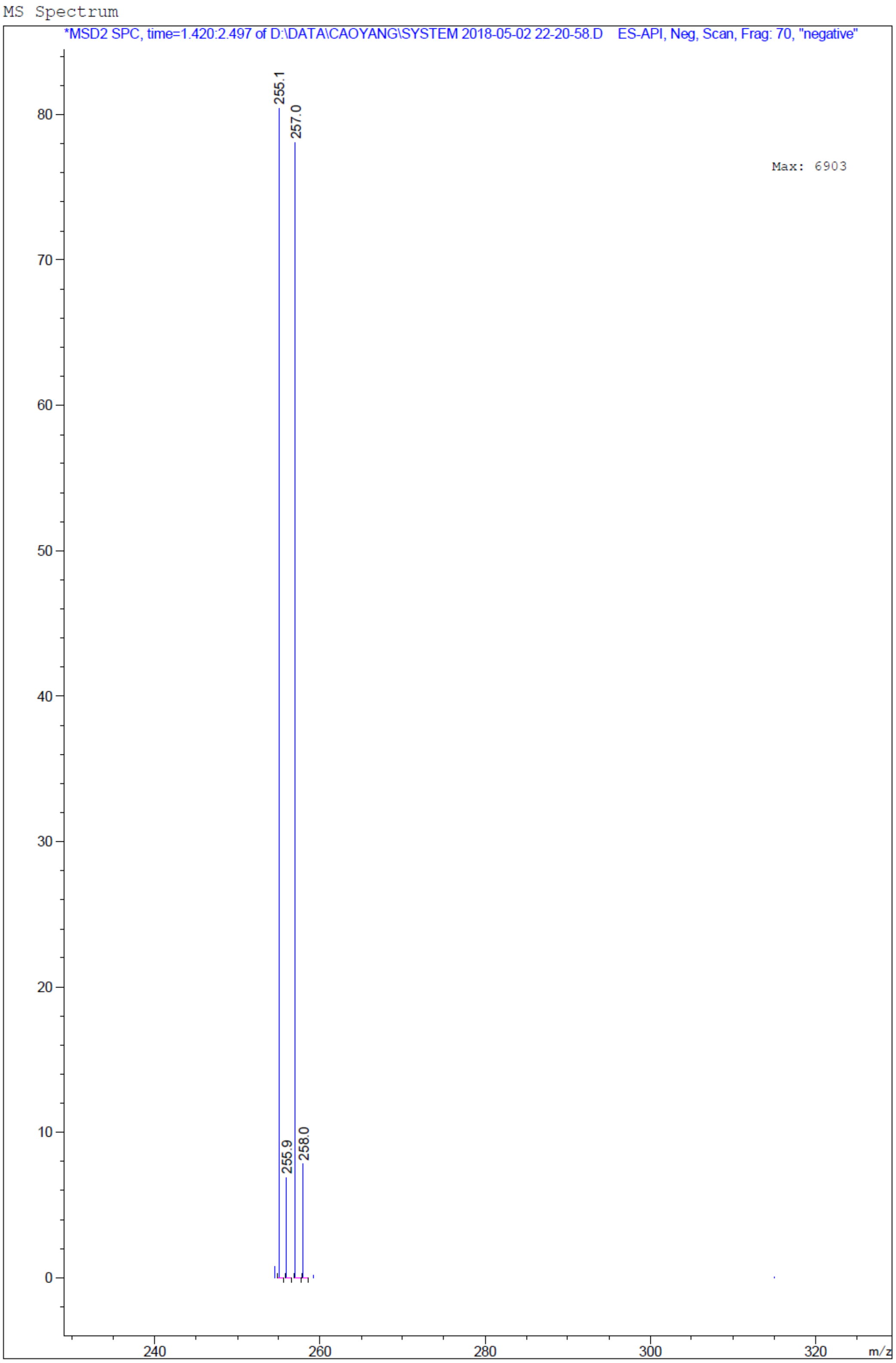

Figure S63. Mass spectrum (negative ionization) of **19**

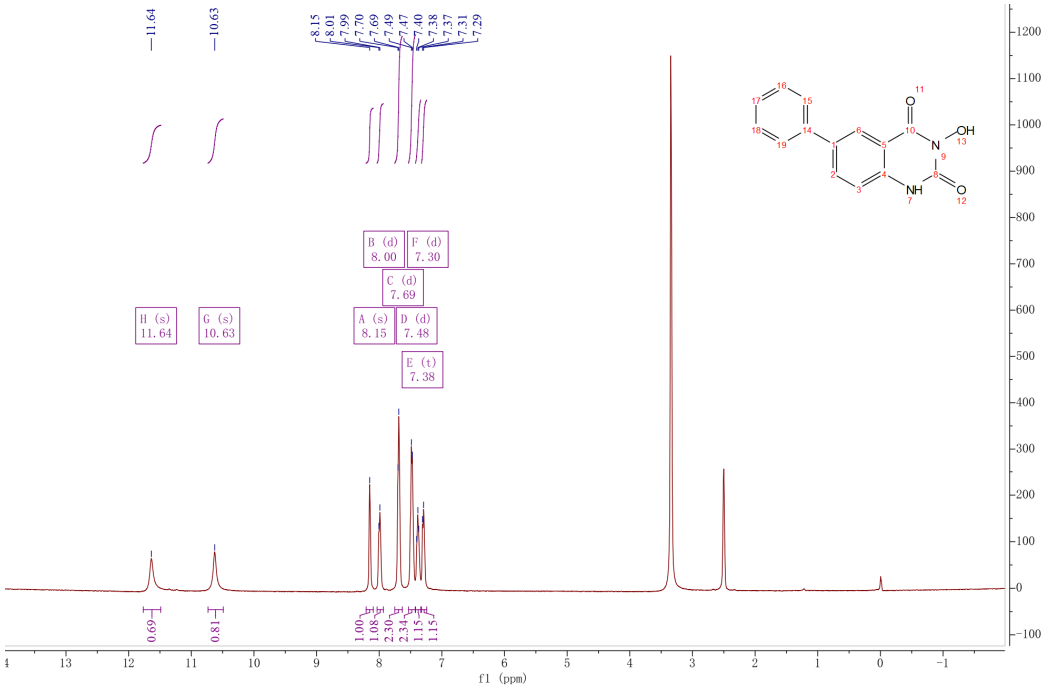

Figure S64. ^{1}H NMR (400 MHz, DMSO-d_6) spectrum of **21a**

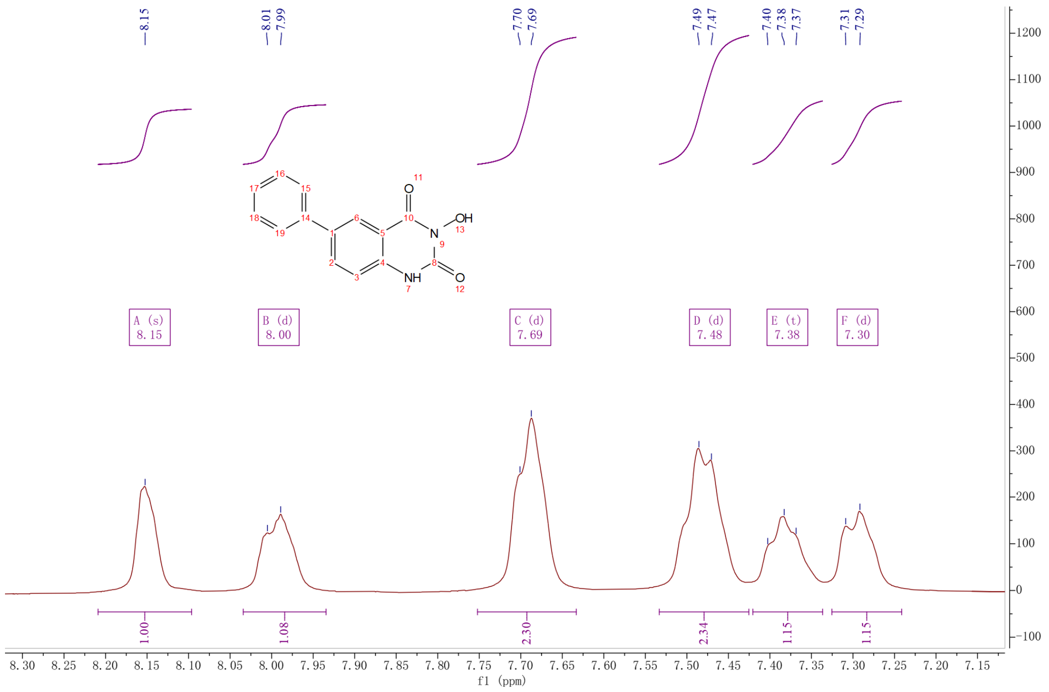

Figure S65. Magnified ^{1}H NMR (400 MHz, DMSO-d_6) spectrum fragments of **21a**

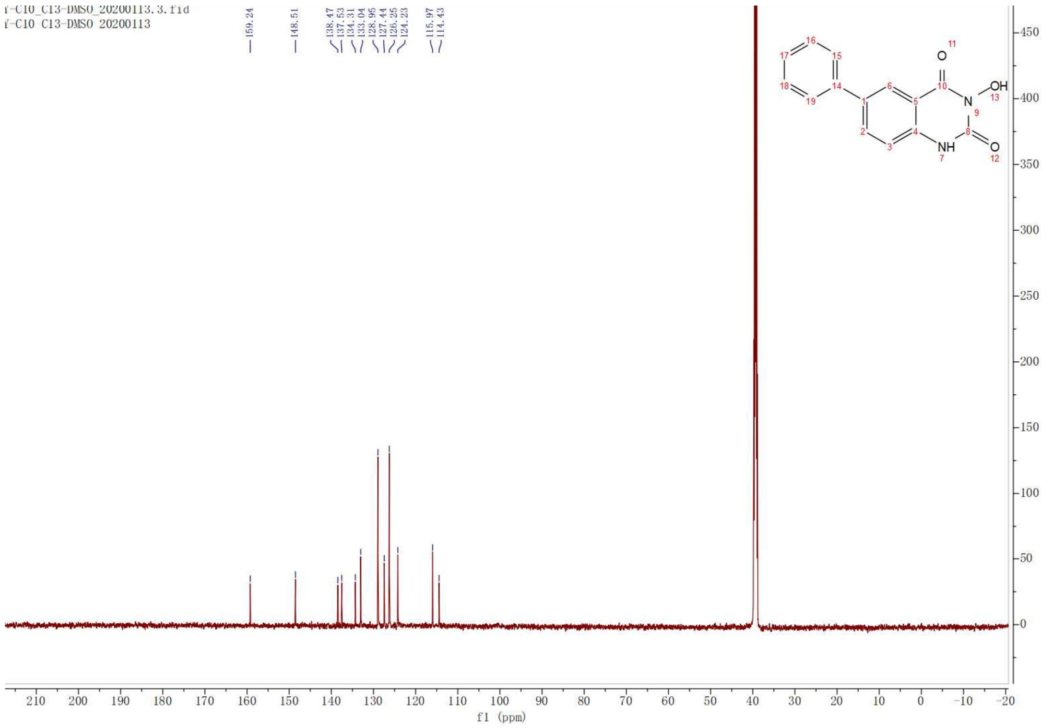

Figure S66. ^{13}C NMR (151 MHz, DMSO-d_6) spectrum of **21a**

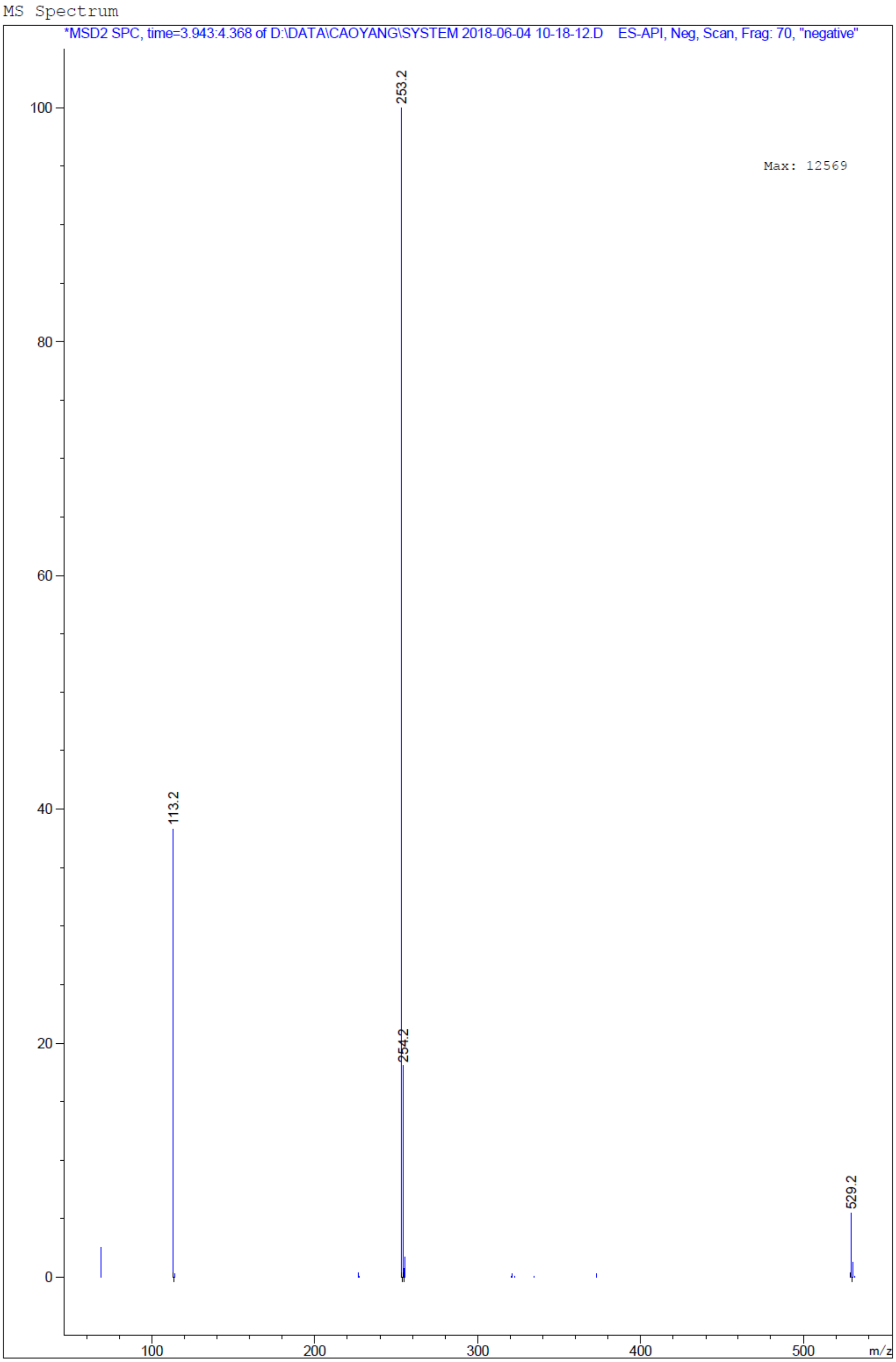

Figure S67. Mass spectrum (negative ionization) of **21a**

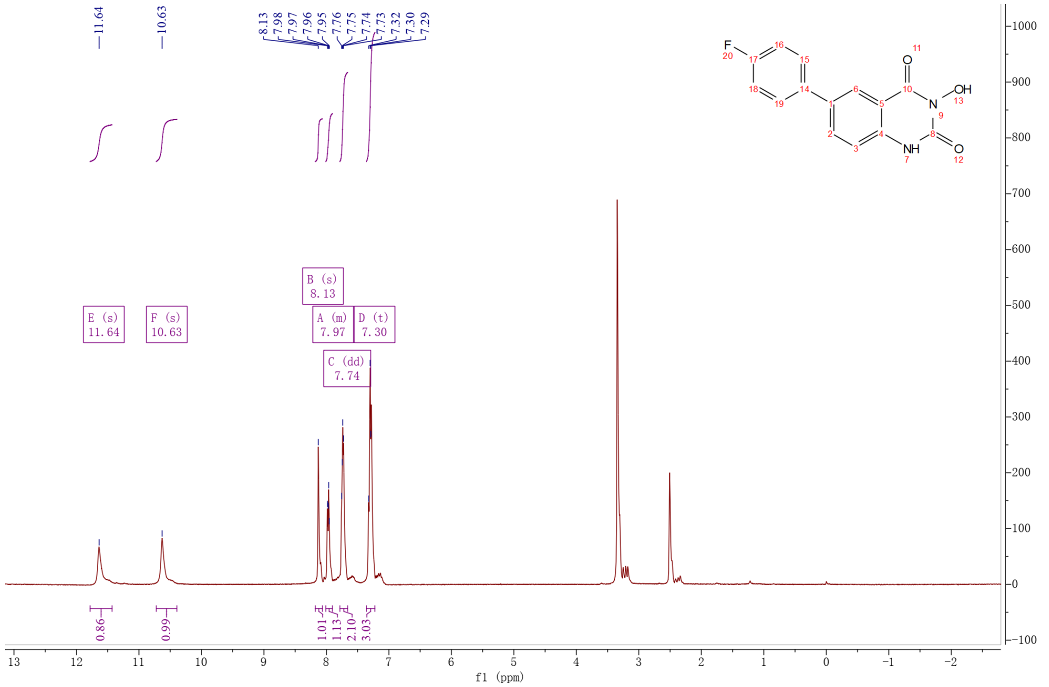

Figure S68. ^{1}H NMR (400 MHz, DMSO-d_6) spectrum of **21b**

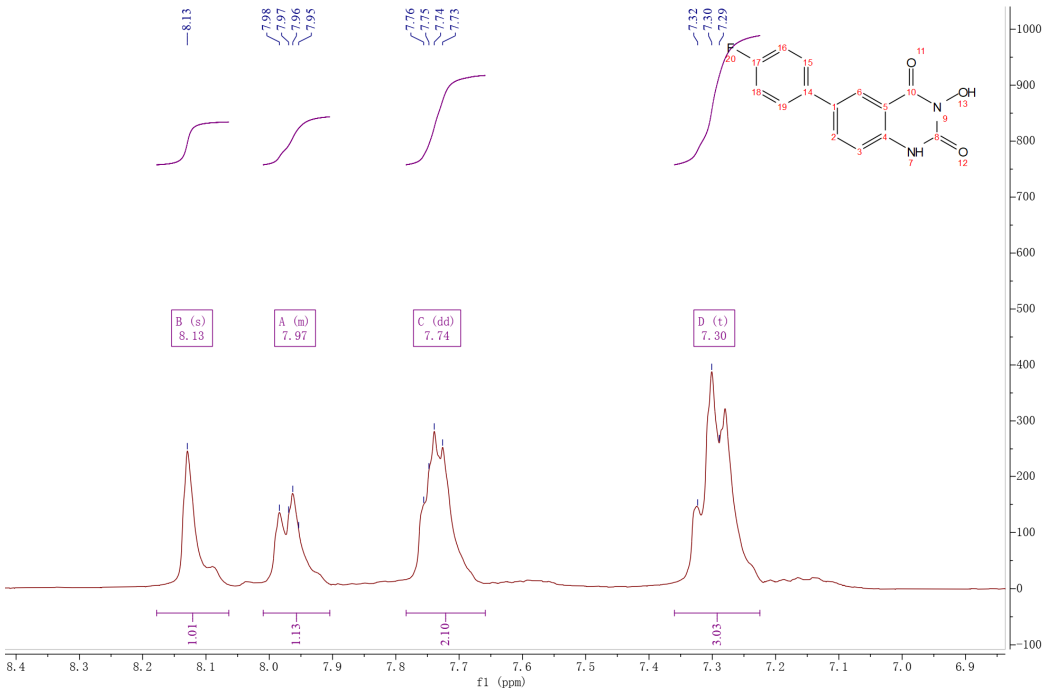

Figure S69. Magnified ^{1}H NMR (400 MHz, DMSO-d_6) spectrum fragments of **21b**

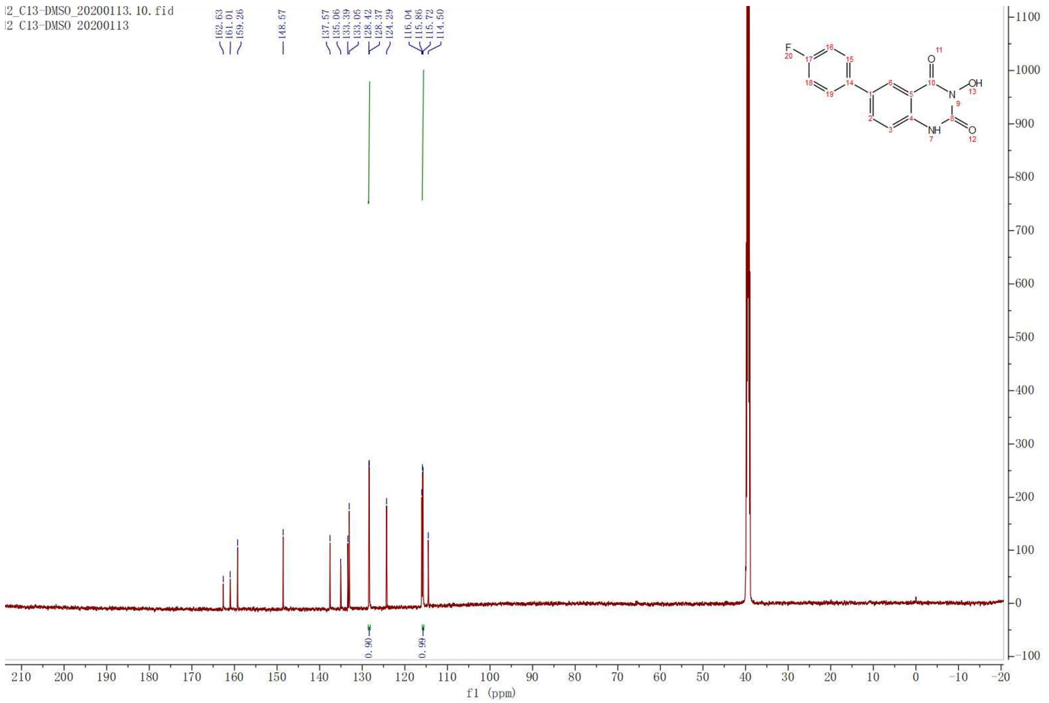

Figure S70. ^{13}C NMR (151 MHz, DMSO-d_6) spectrum of **21b**

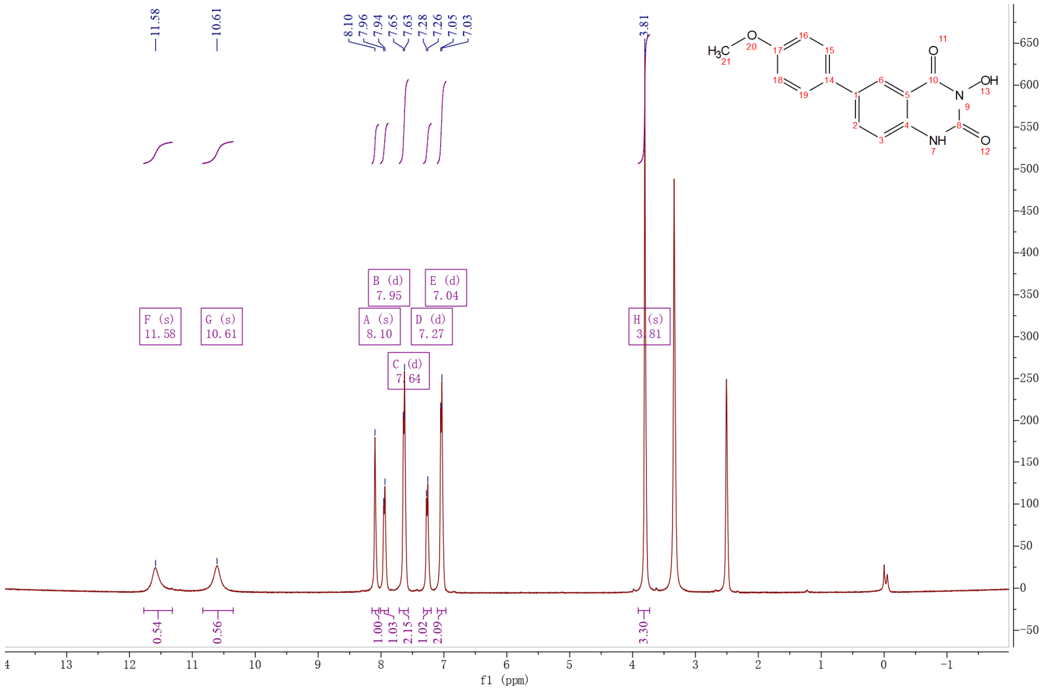

Figure S71. ^{1}H NMR (400 MHz, DMSO-d_6) spectrum of **21c**

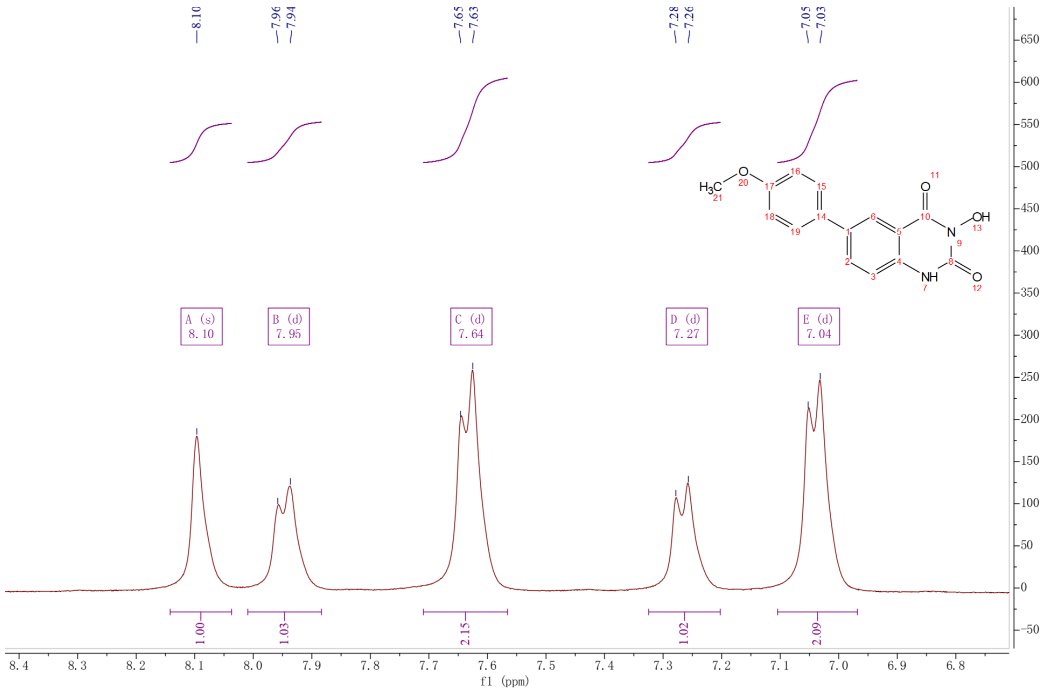

Figure S72. Magnified ^{1}H NMR (400 MHz, DMSO-d_6) spectrum fragments of **21c**

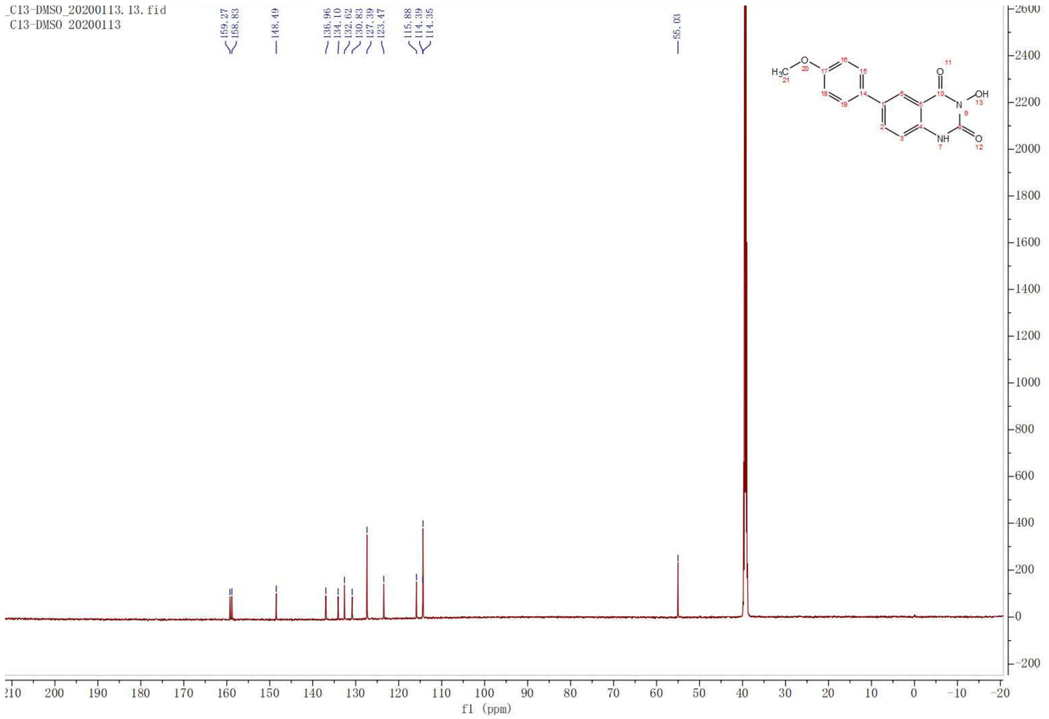

Figure S73. ^{13}C NMR (151 MHz, DMSO-d_6) spectrum of **21c**

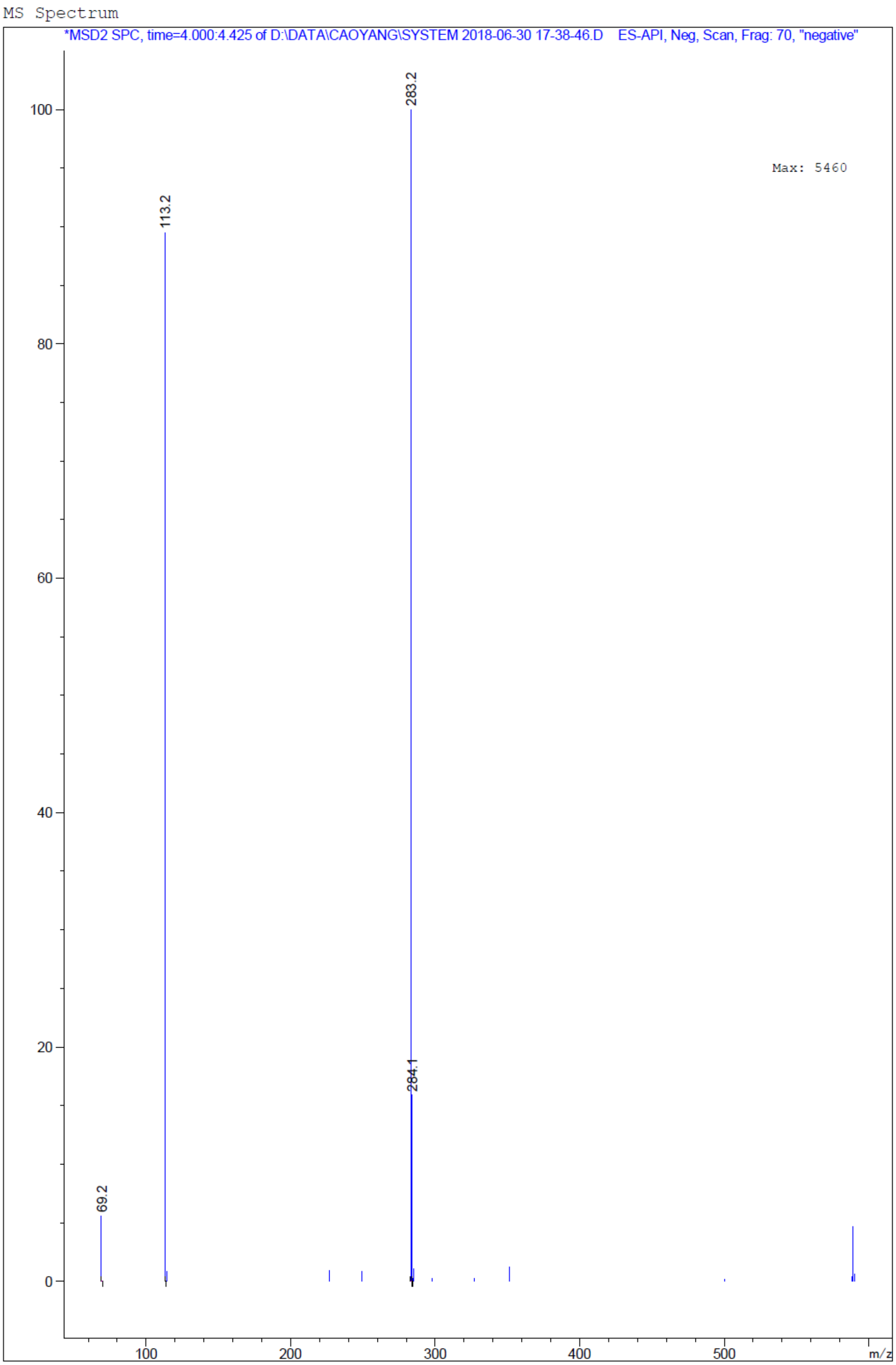

Figure S74. Mass spectrum (negative ionization) of **21c**

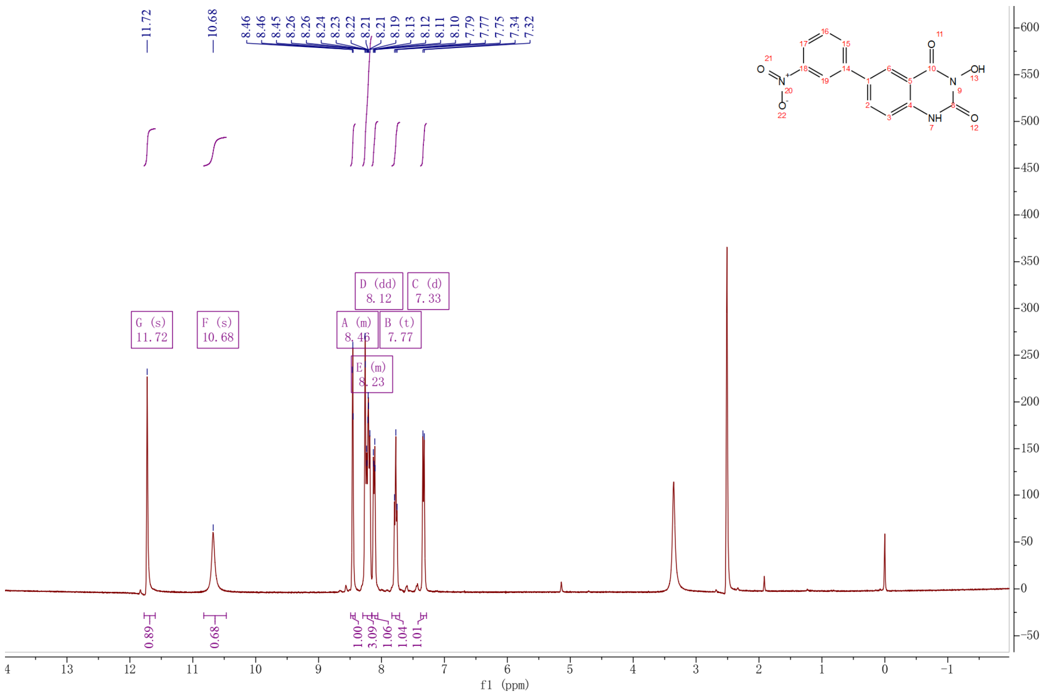

Figure S75. ^{1}H NMR (400 MHz, DMSO-d_6) spectrum of **21d**

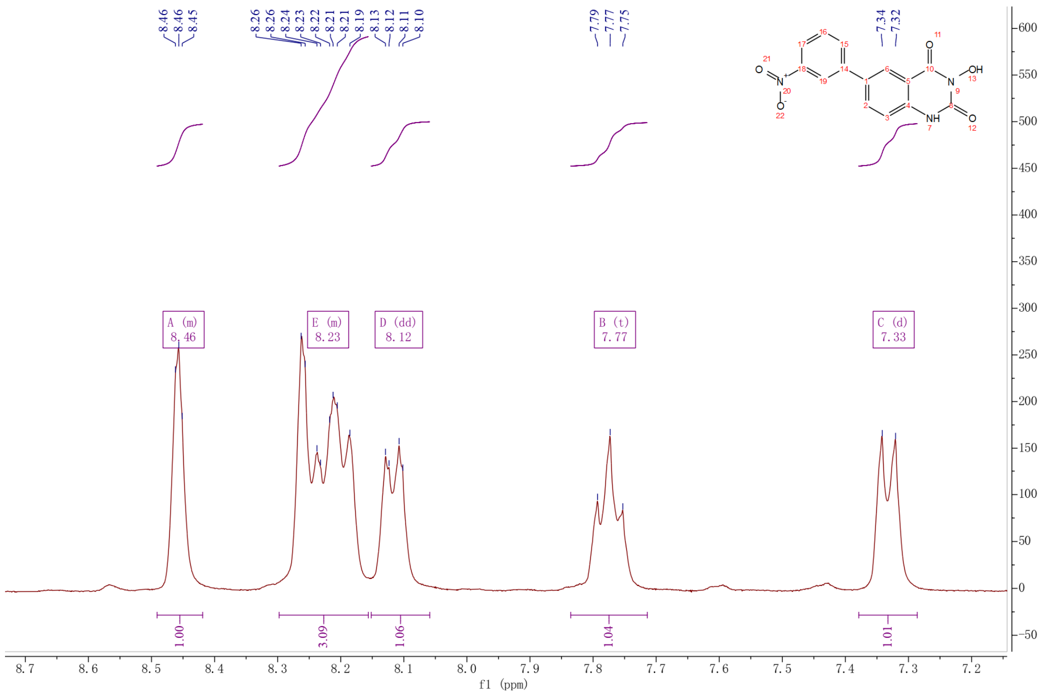

Figure S76. Magnified ^{1}H NMR (400 MHz, DMSO-d_6) spectrum fragments of **21d**

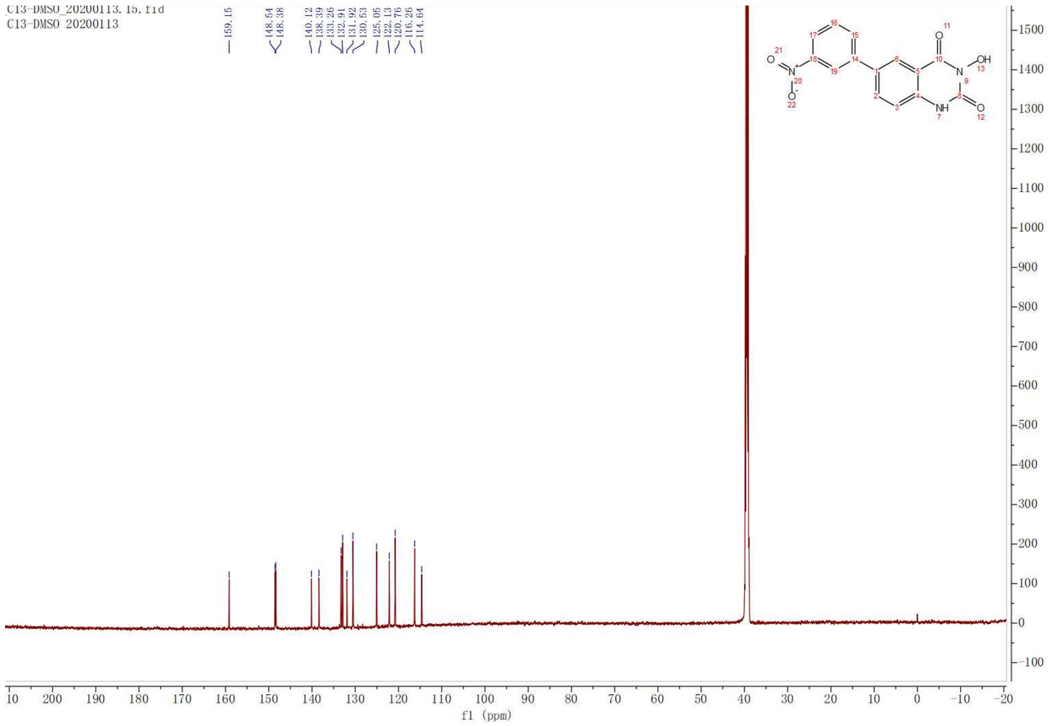

Figure S77. ^{13}C NMR (151 MHz, DMSO-d_6) spectrum of **21d**

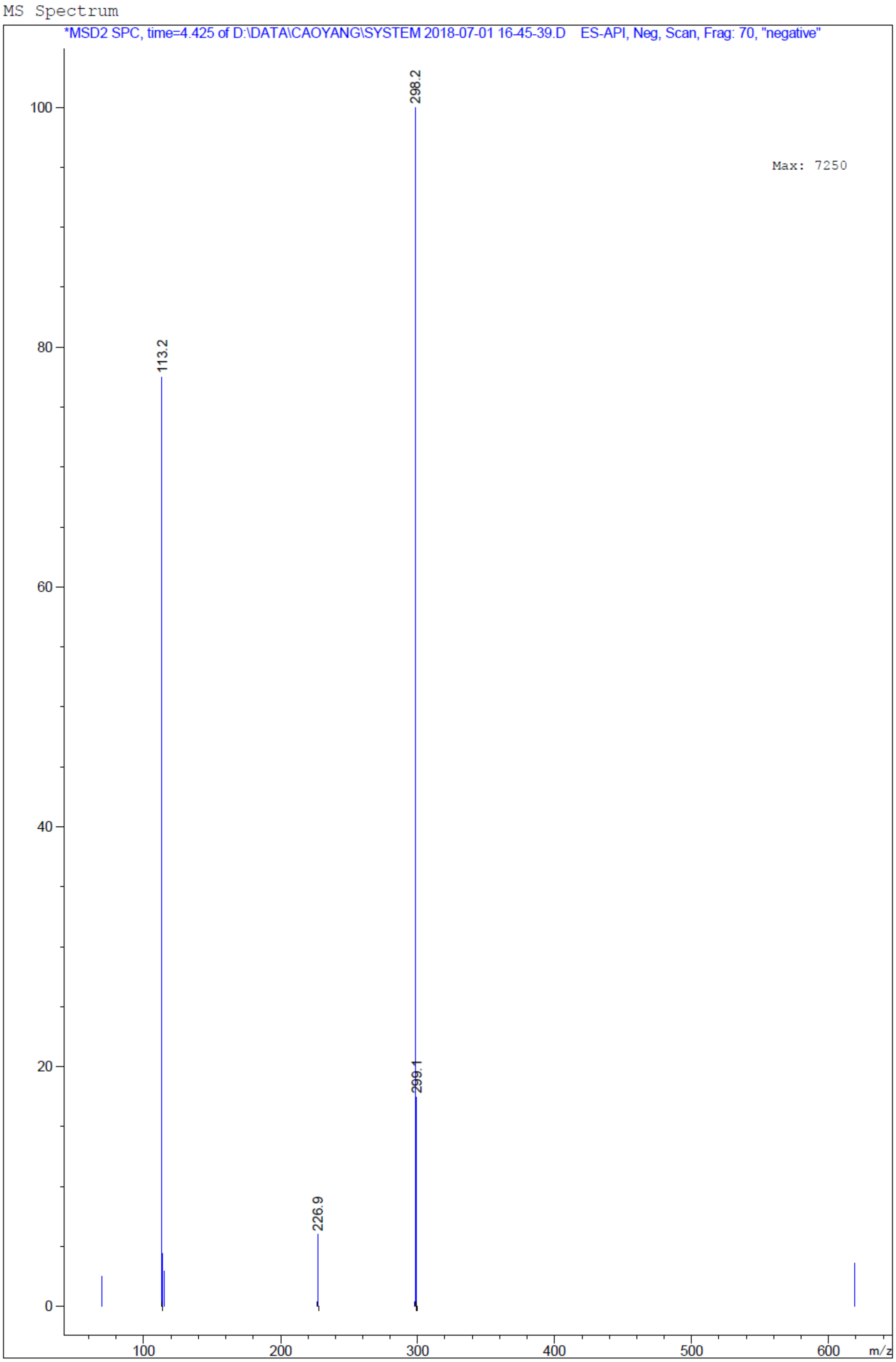

Figure S78. Mass spectrum (negative ionization) of **21d**

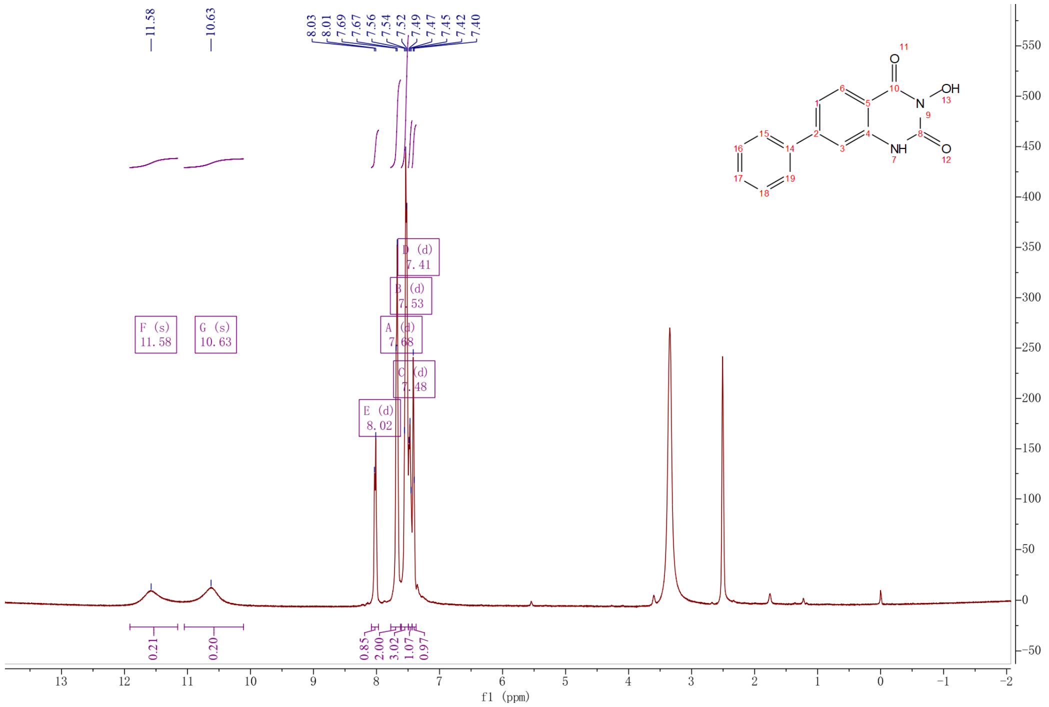

Figure S79. ^{1}H NMR (400 MHz, DMSO-d_6) spectrum of **21e**

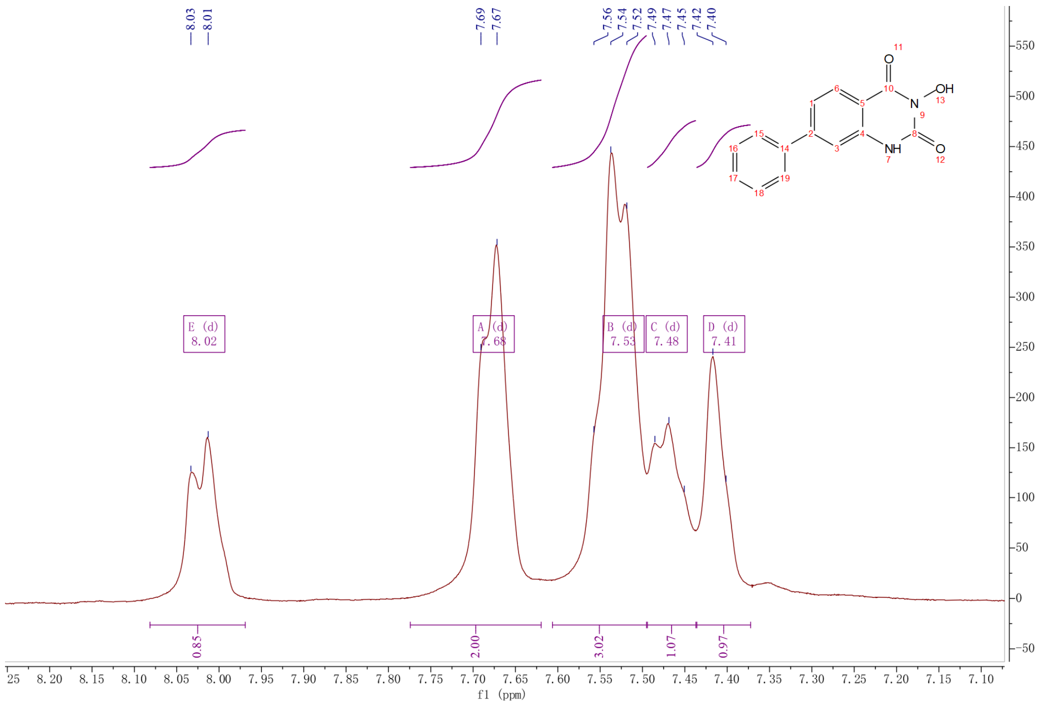

Figure S80. Magnified ^{1}H NMR (400 MHz, DMSO-d_6) spectrum fragments of **21e**

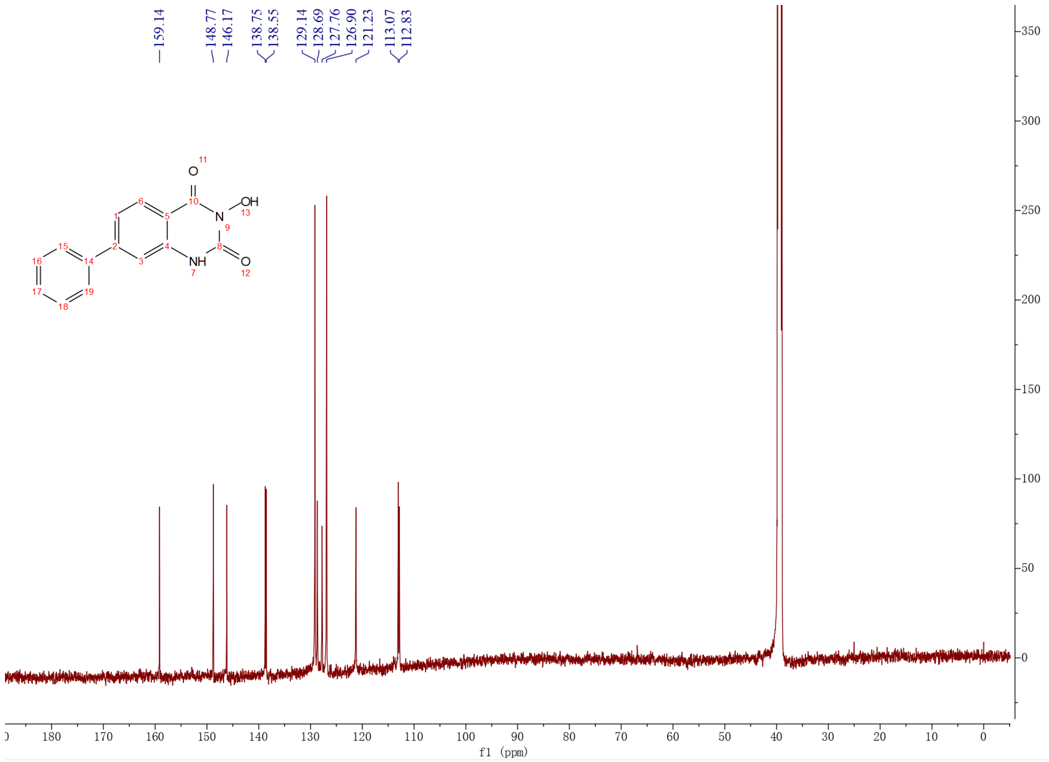

Figure S81. ^{13}C NMR (151 MHz, DMSO-d_6) spectrum of **21e**

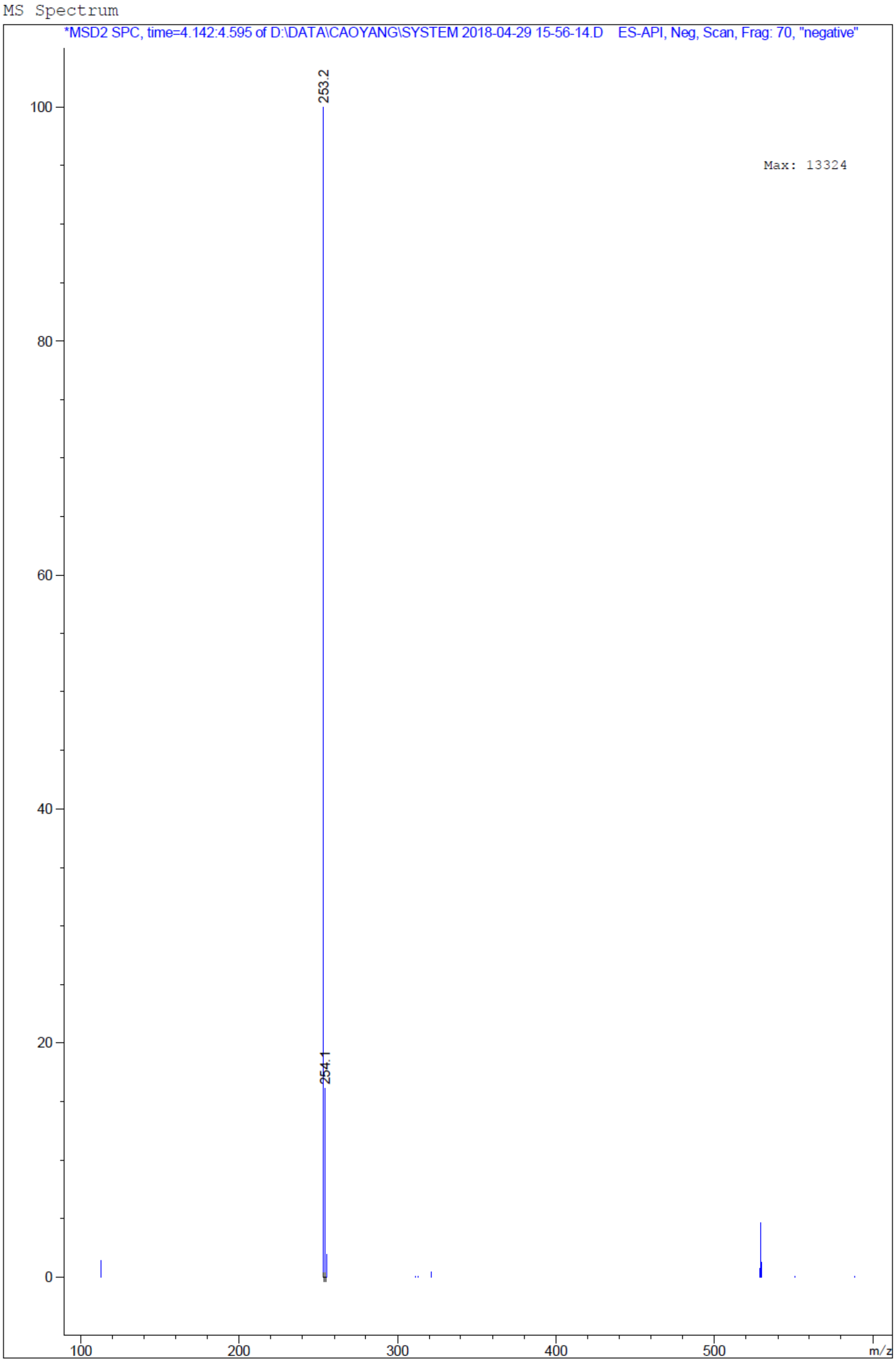

Figure S82. Mass spectrum (negative ionization) of **21e**

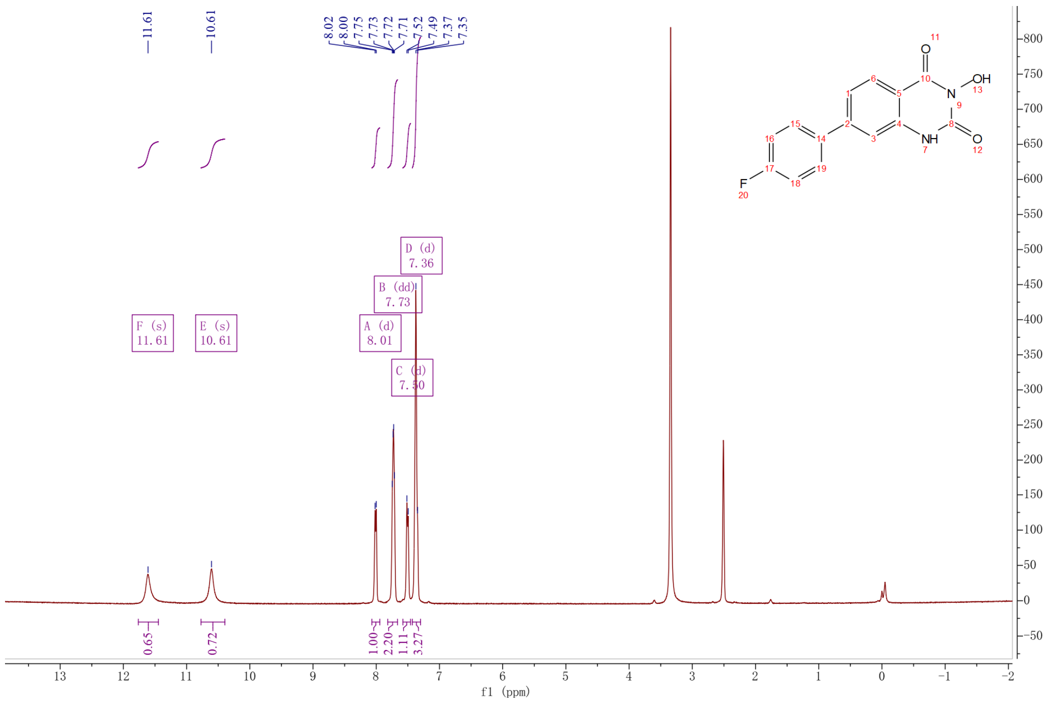

Figure S83. ^{1}H NMR (400 MHz, DMSO-d_6) spectrum of **21f**

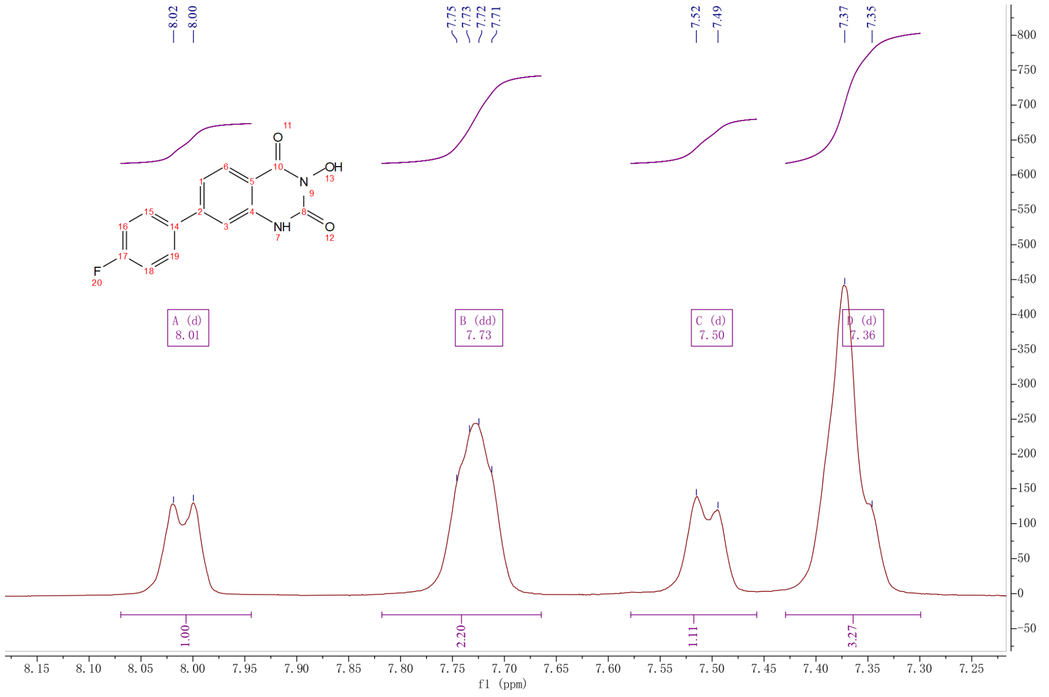

Figure S84. Magnified ^{1}H NMR (400 MHz, DMSO-d_6) spectrum fragments of **21f**

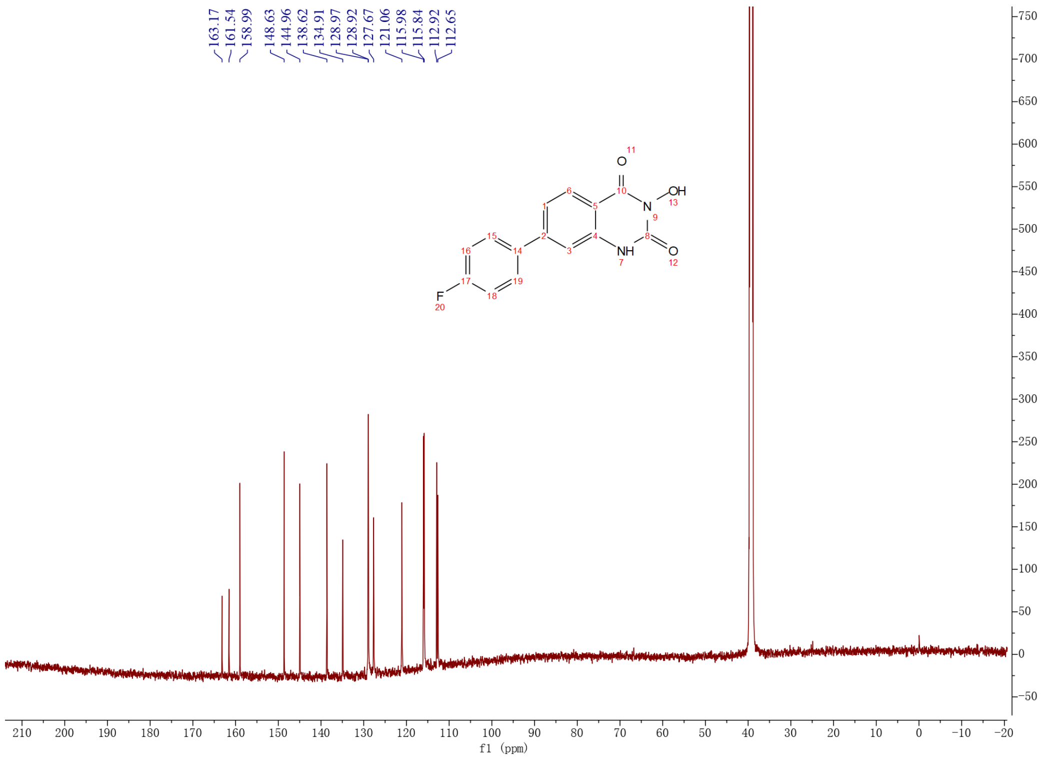

Figure S85. ^{13}C NMR (151 MHz, DMSO-d_6) spectrum of **21f**

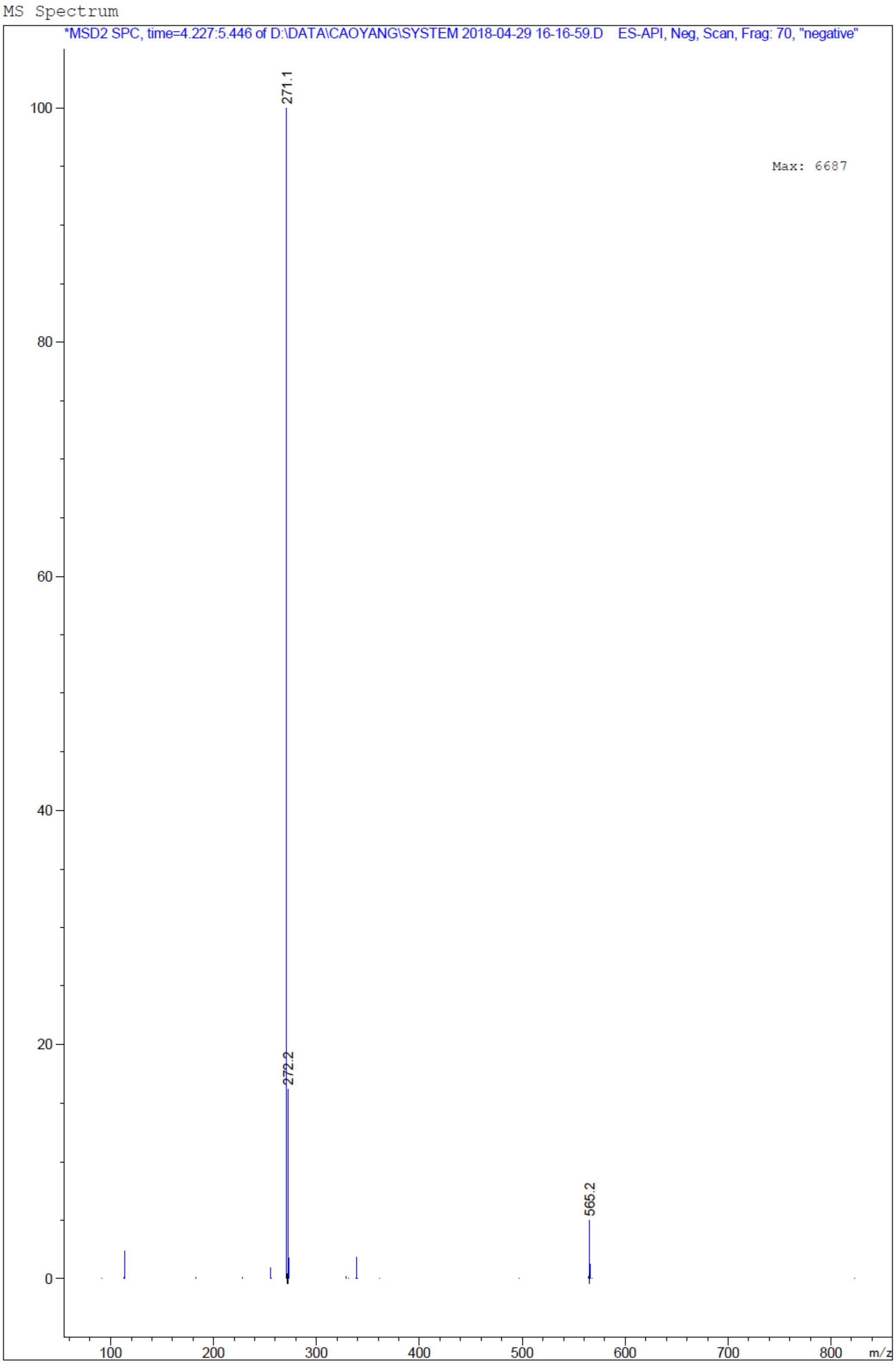

Figure S86. Mass spectrum (negative ionization) of **21f**

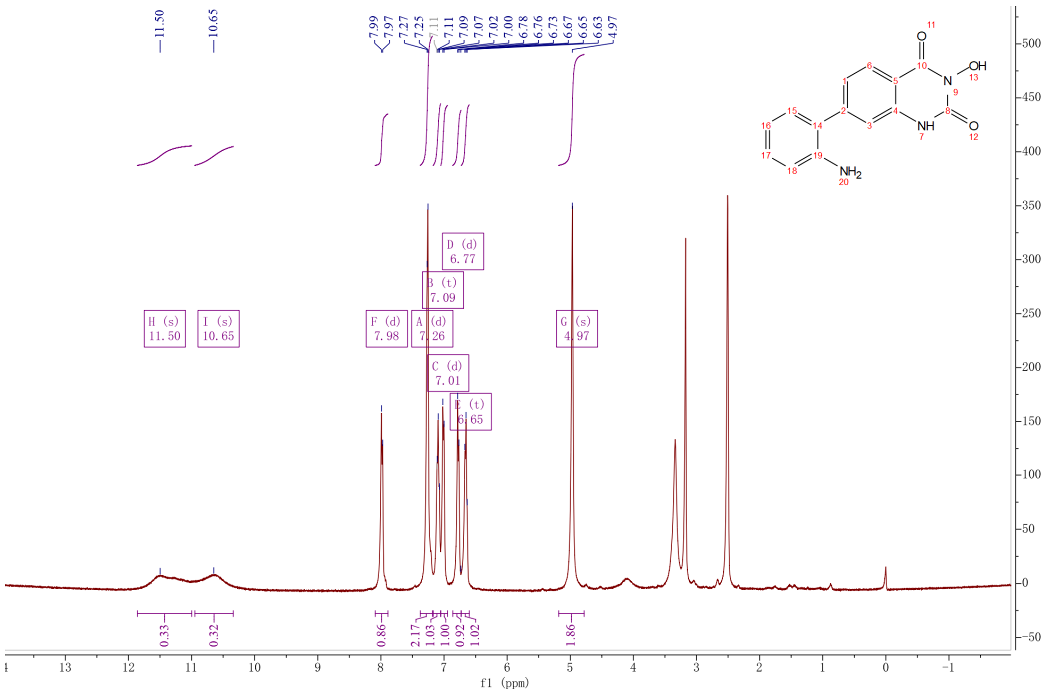

Figure S87. ^{1}H NMR (400 MHz, DMSO-d_6) spectrum of **21g**

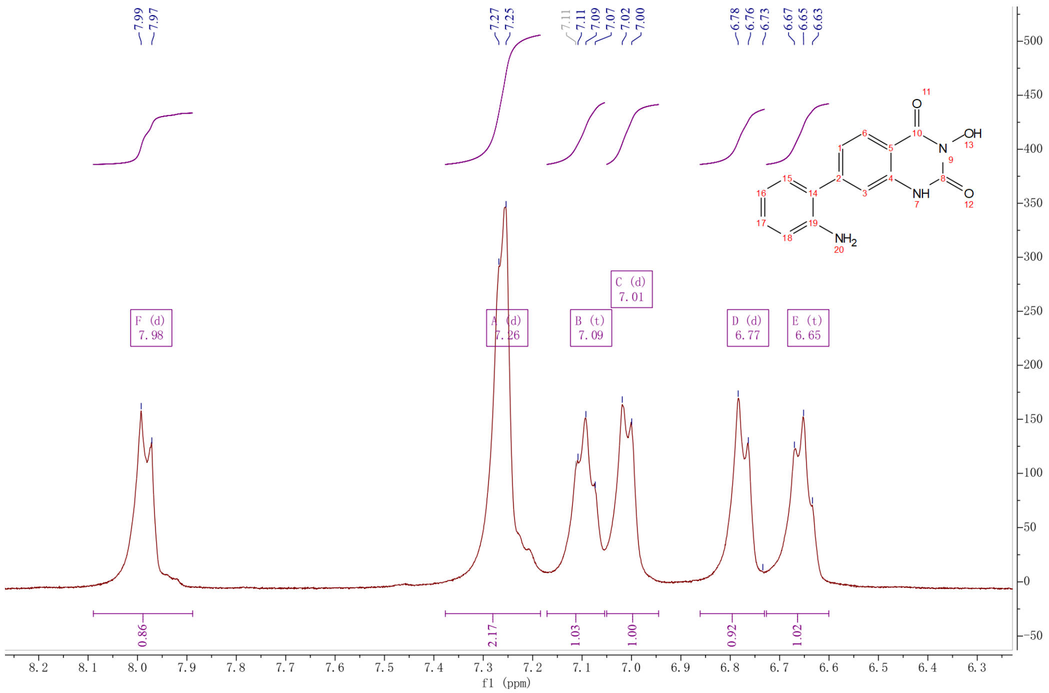

Figure S88. Magnified ^{1}H NMR (400 MHz, DMSO-d_6) spectrum fragments of **21g**

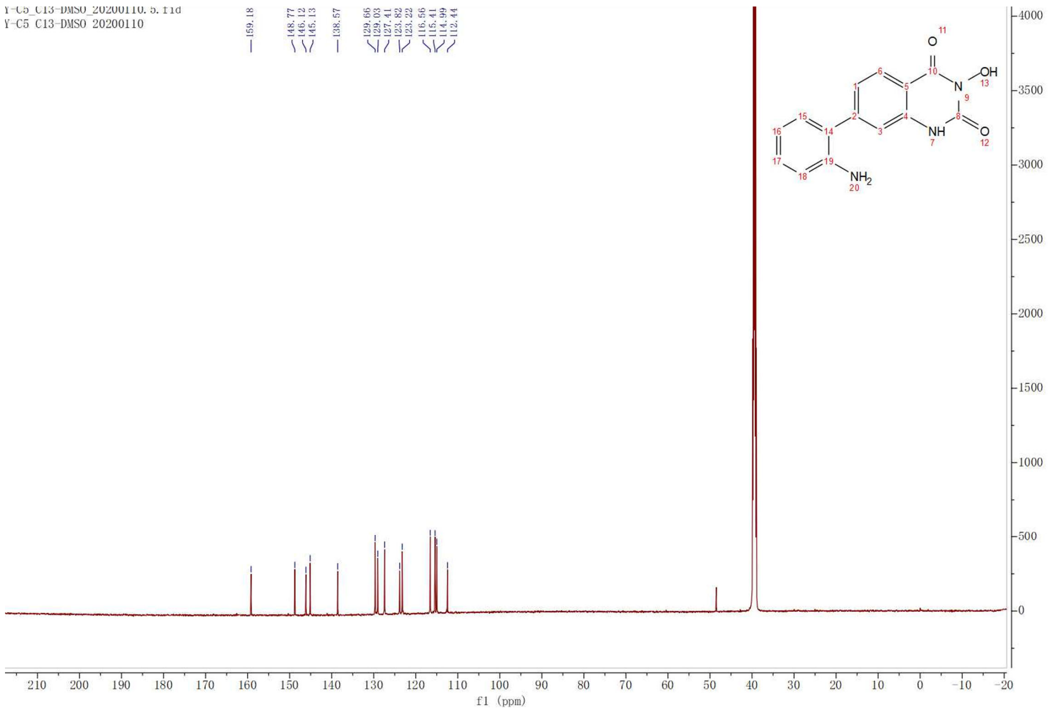

Figure S89. ^{13}C NMR (151 MHz, DMSO-d_6) spectrum of **21g**

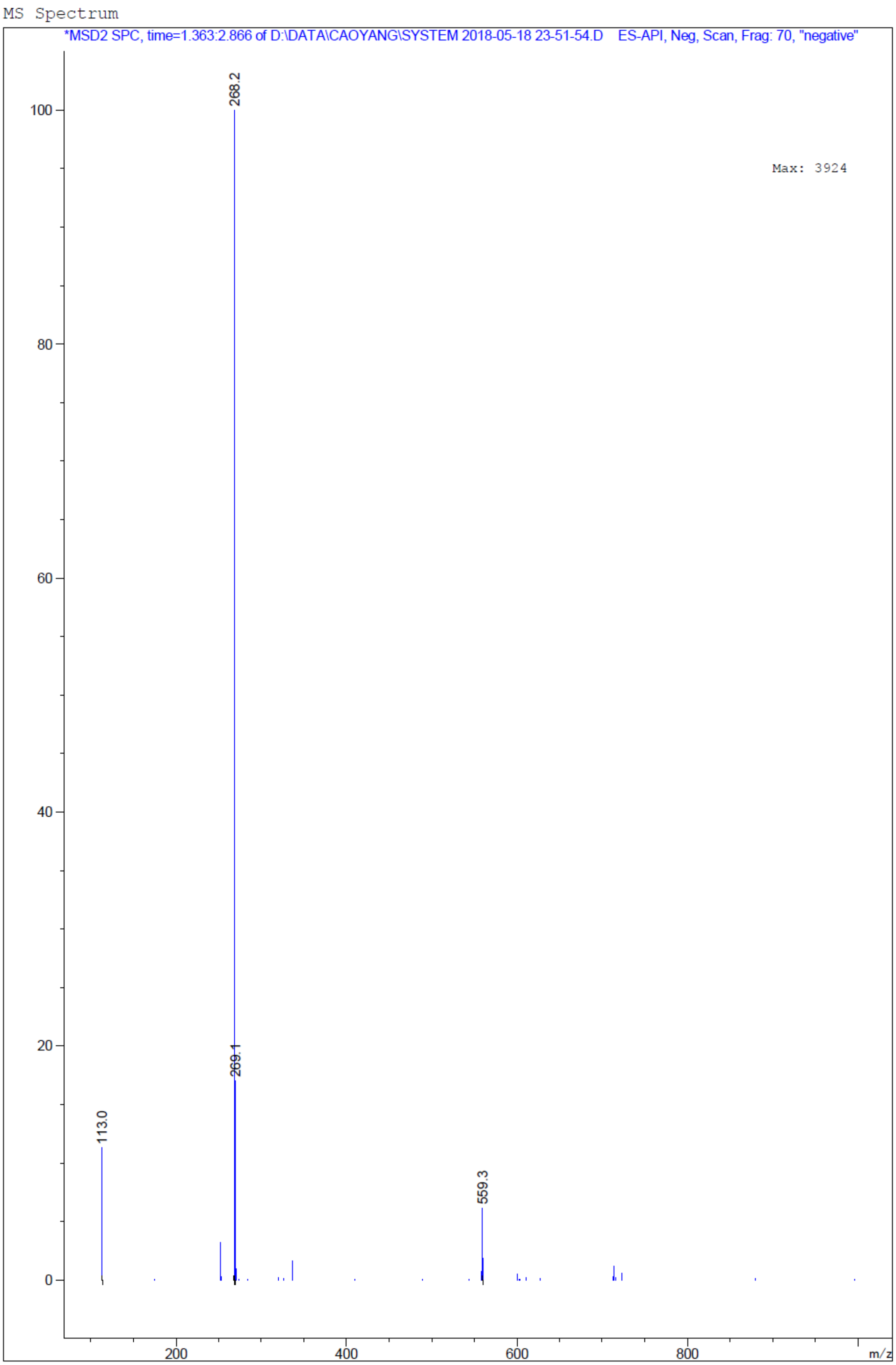

Figure S90. Mass spectrum (negative ionization) of **21g**

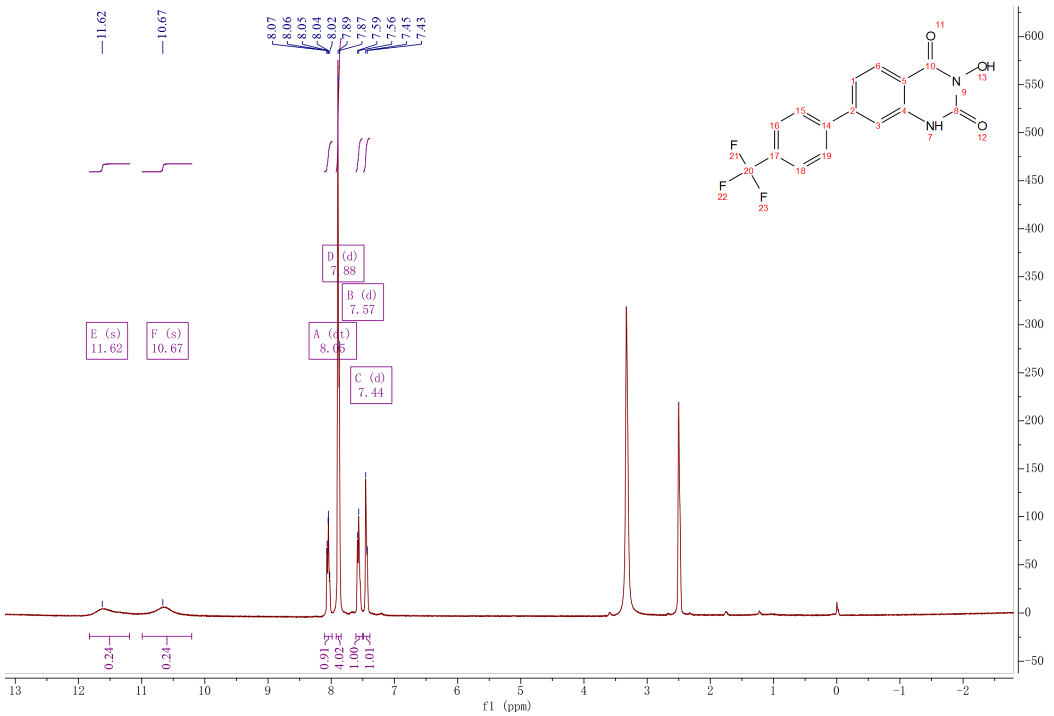

Figure S91. ^{1}H NMR (400 MHz, DMSO-d_6) spectrum of **21h**

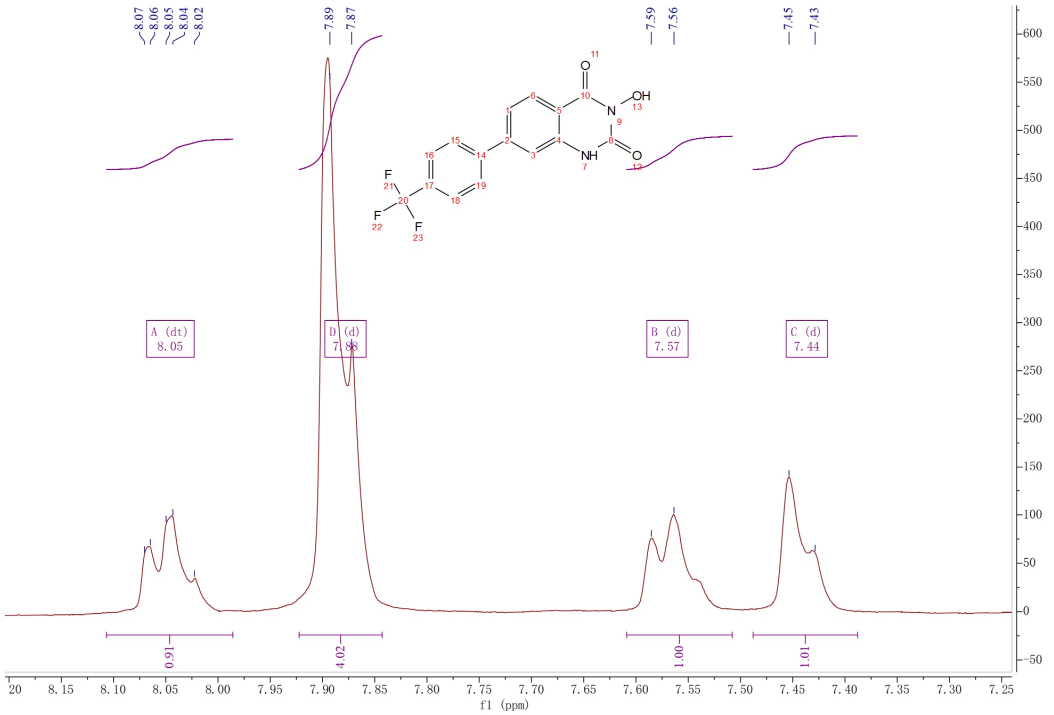

Figure S92. Magnified ^{1}H NMR (400 MHz, DMSO-d_6) spectrum fragments of **21h**

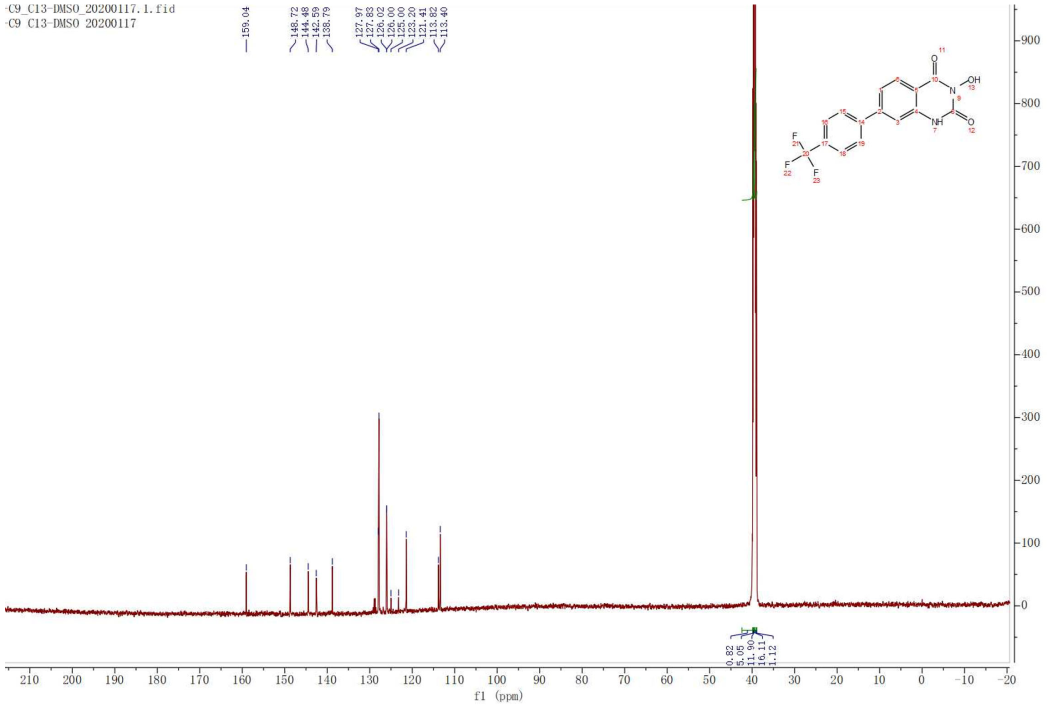

Figure S93. ^{13}C NMR (151 MHz, DMSO-d_6) spectrum of **21h**

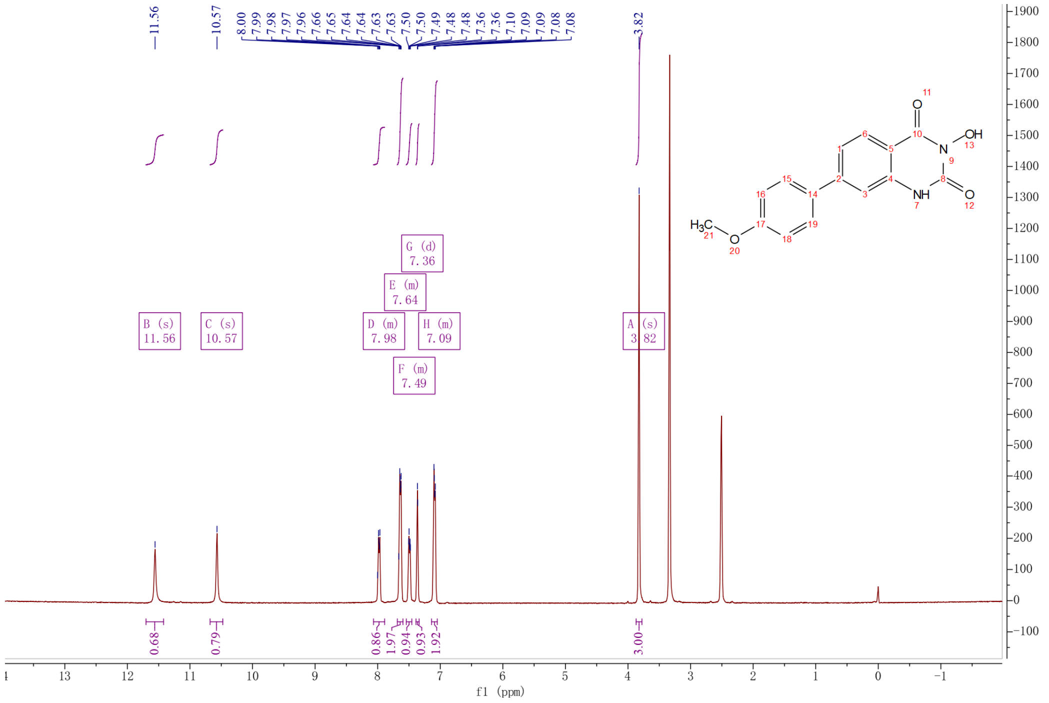

Figure S94. ^{1}H NMR (400 MHz, DMSO-d_6) spectrum of **21i**

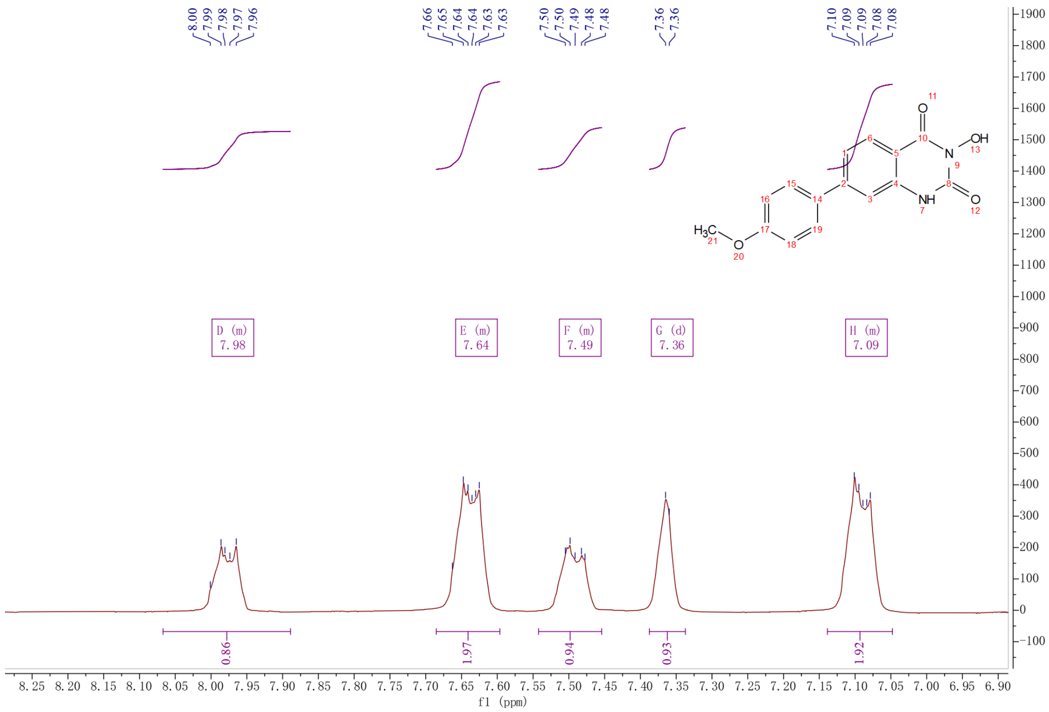

Figure S95. Magnified ^{1}H NMR (400 MHz, DMSO-d_6) spectrum fragments of **21i**

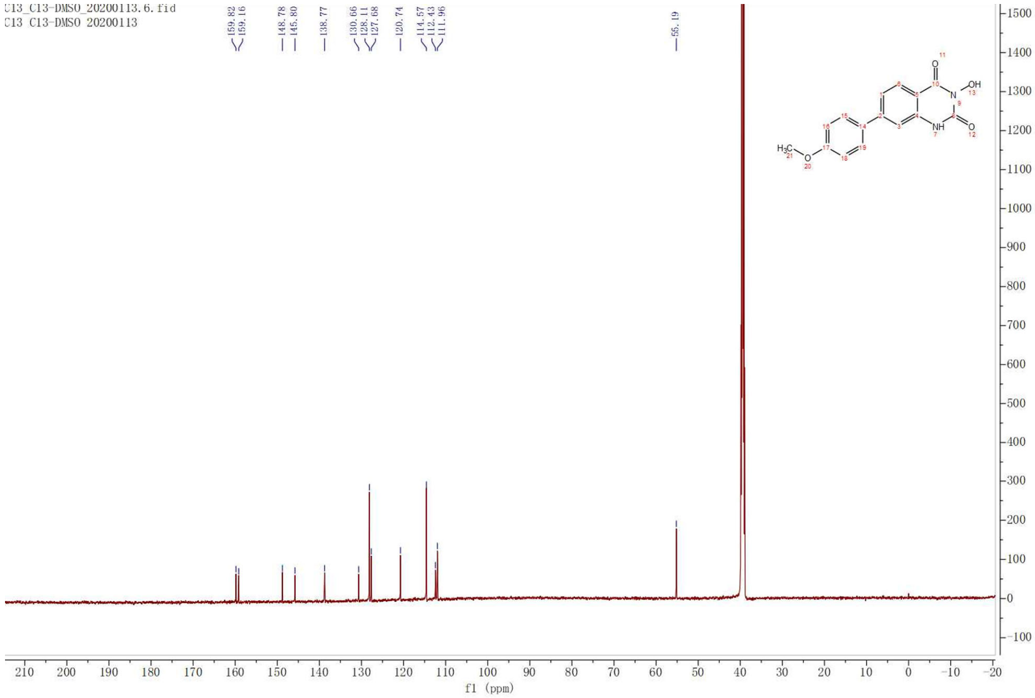

Figure S96. ^{13}C NMR (151 MHz, DMSO-d_6) spectrum of **21i**

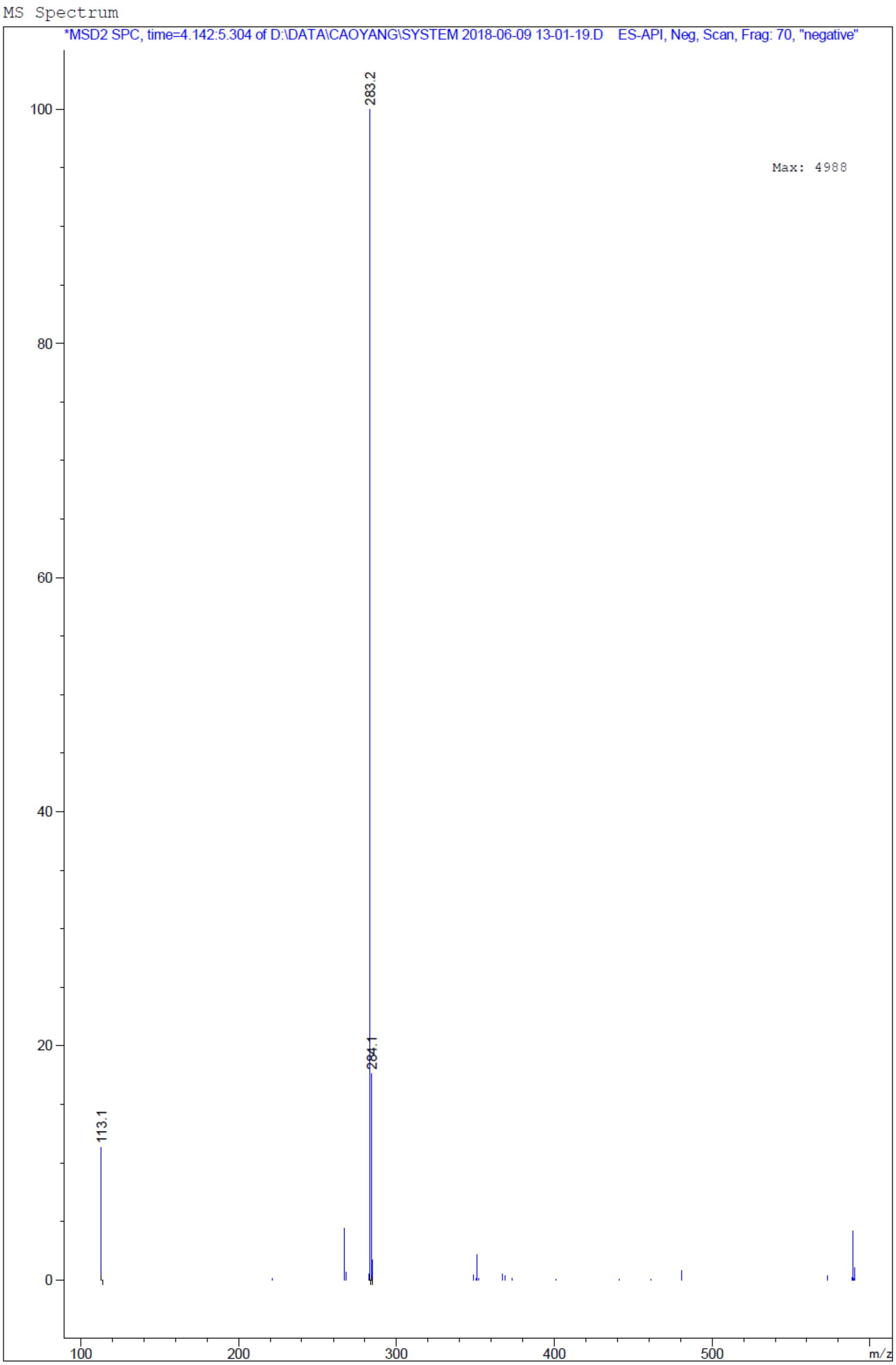

Figure S97. Mass spectrum (negative ionization) of **21i**

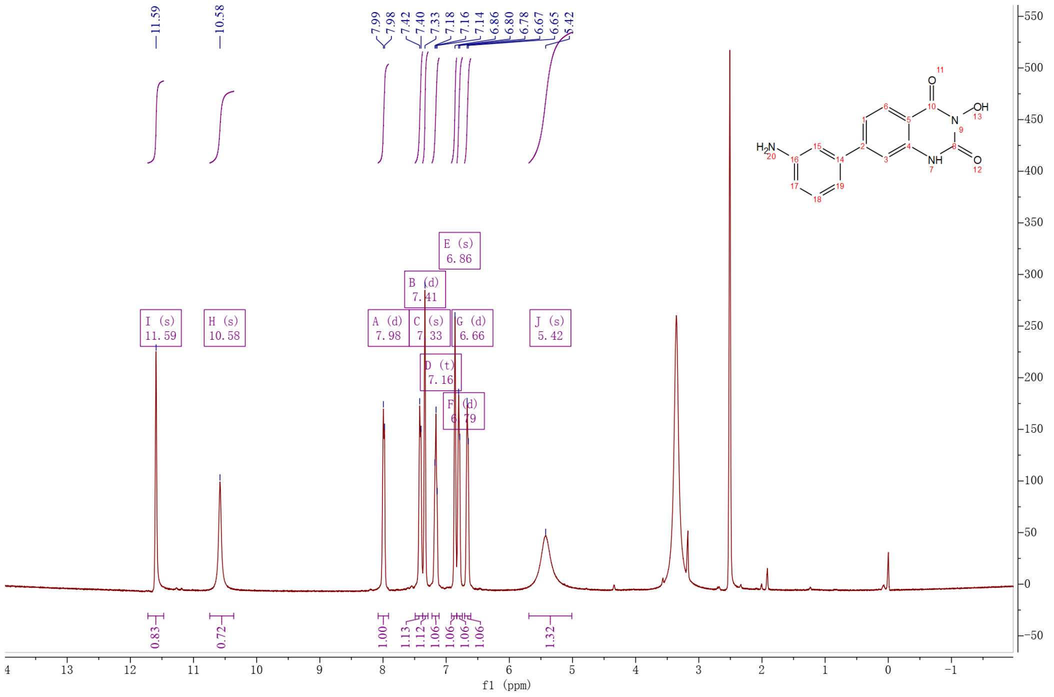

Figure S98. ^{1}H NMR (400 MHz, DMSO-d_6) spectrum of **21j**

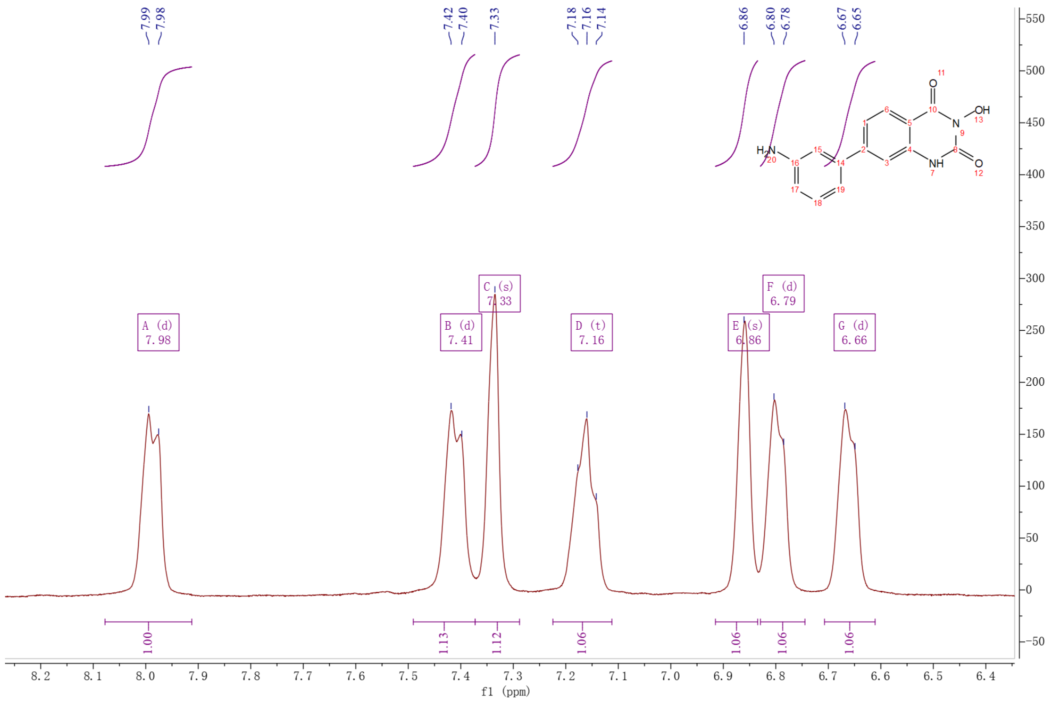

Figure S99. Magnified ^{1}H NMR (400 MHz, DMSO-d_6) spectrum fragments of **21j**

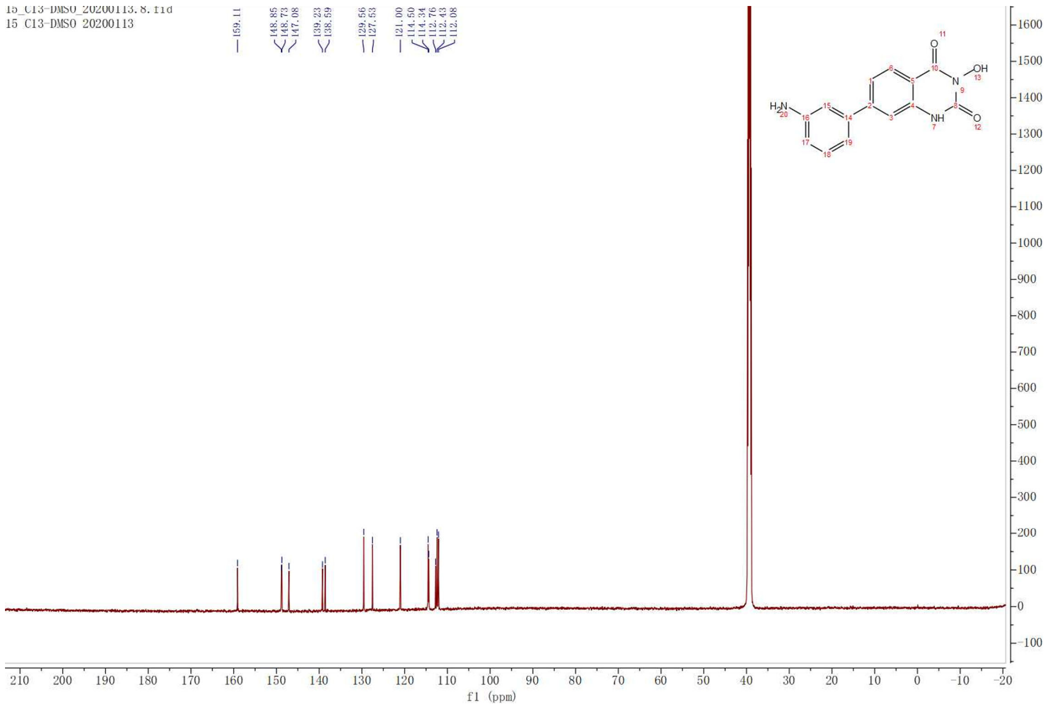

Figure S100. ^{13}C NMR (151 MHz, DMSO-d_6) spectrum of **21j**

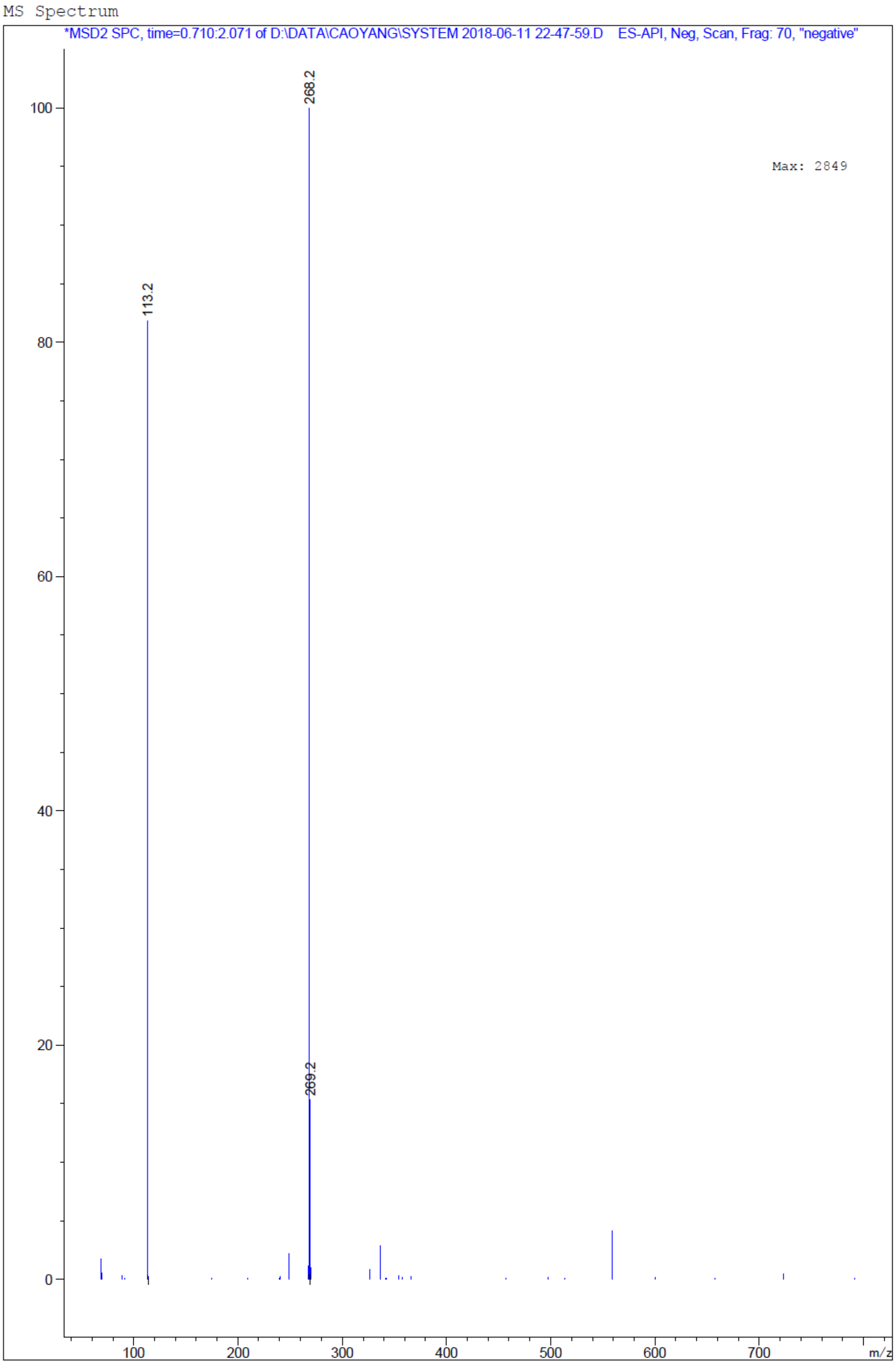

Figure S101. Mass spectrum (negative ionization) of **21j**

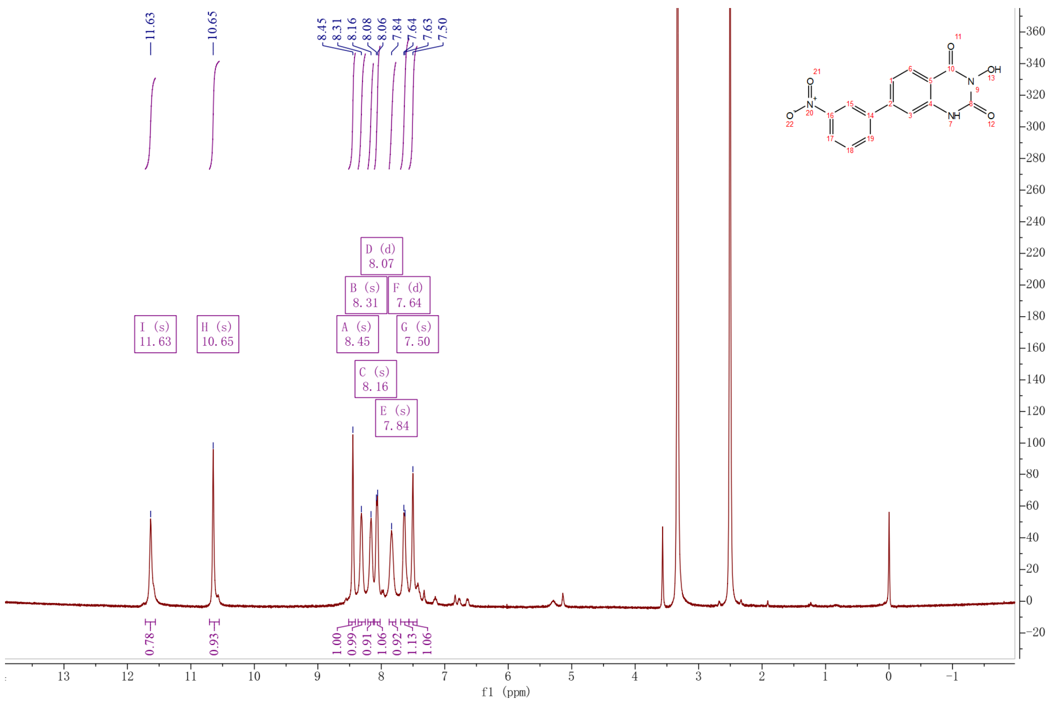

Figure S102. ^{1}H NMR (400 MHz, DMSO-d_6) spectrum of **21k**

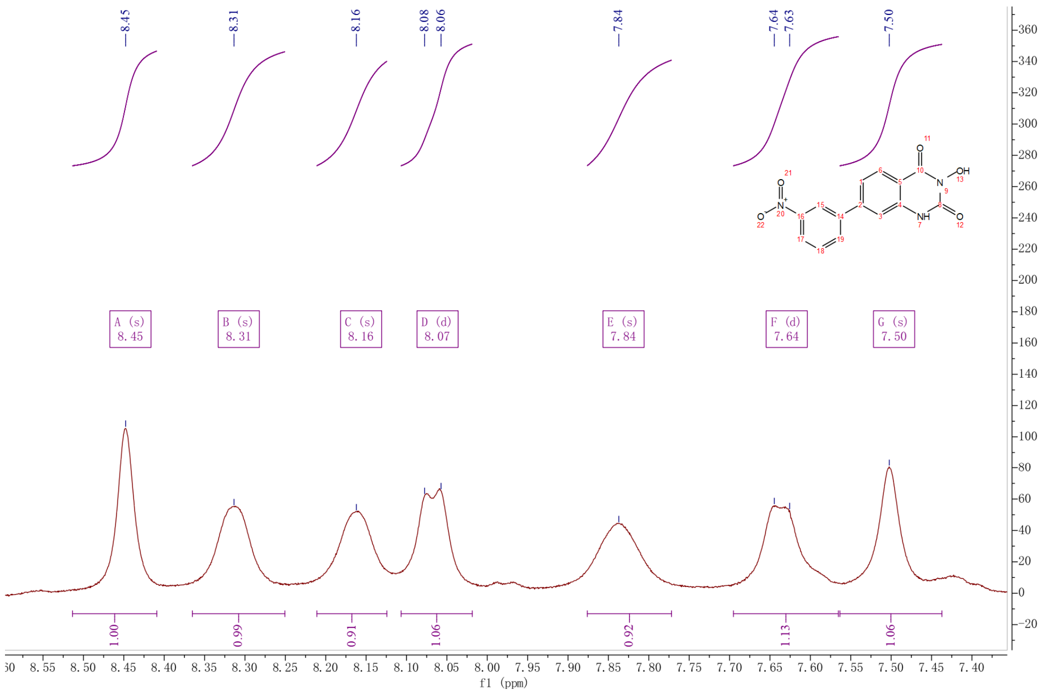

Figure S103. Magnified ^{1}H NMR (400 MHz, DMSO-d_6) spectrum fragments of **21k**

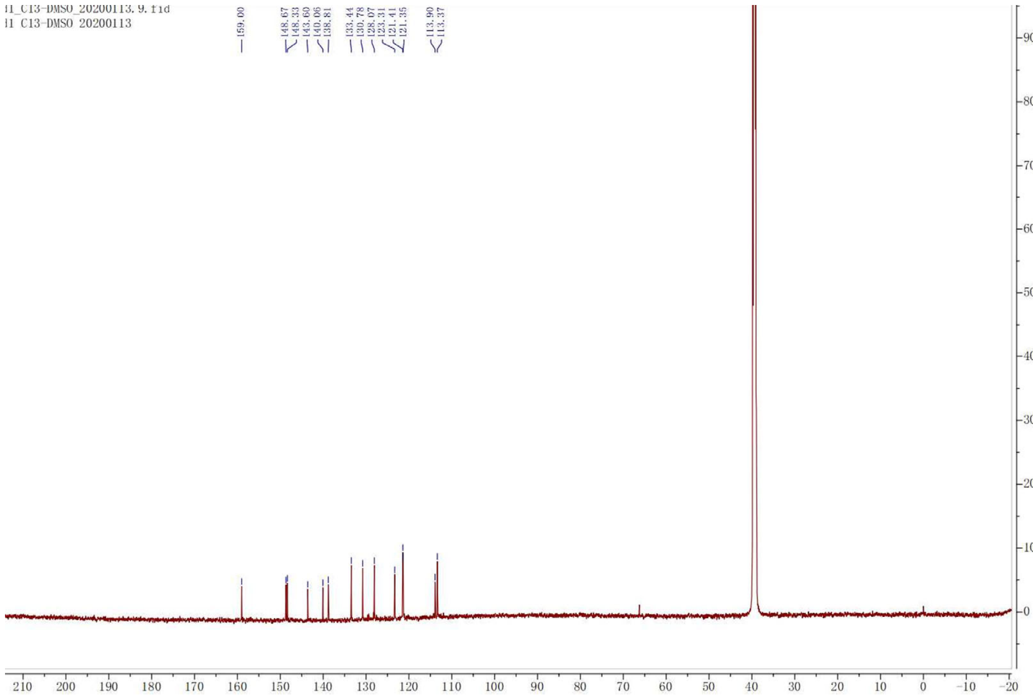

Figure S104. ^{13}C NMR (151 MHz, DMSO-d_6) spectrum of **21k**

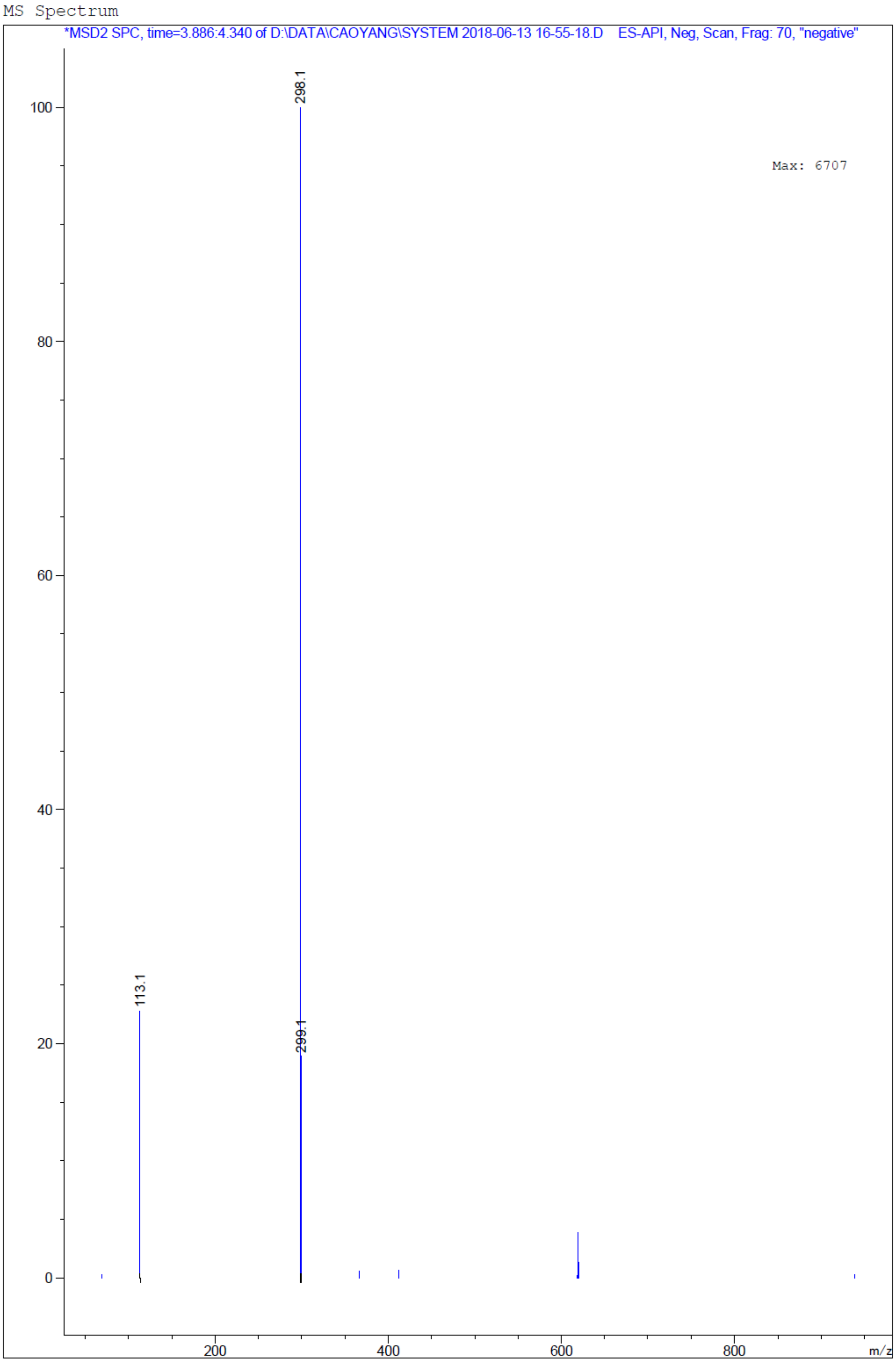

Figure S105. Mass spectrum (negative ionization) of **21k**

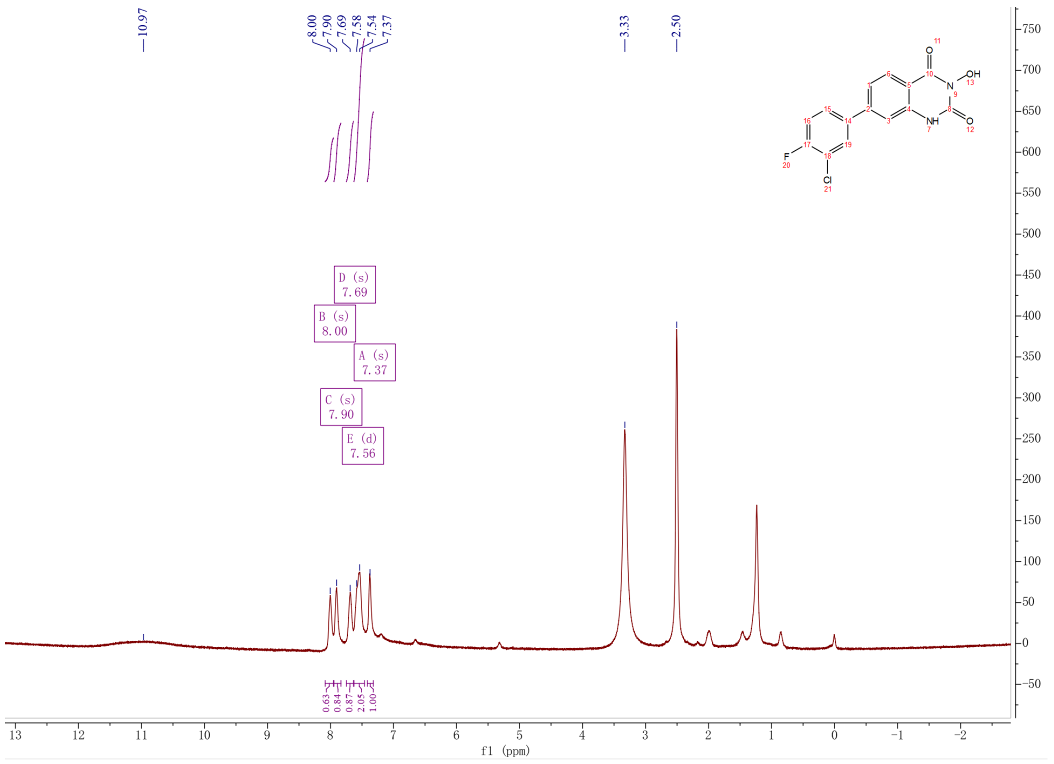

Figure S106. ^{1}H NMR (400 MHz, DMSO-d_6) spectrum of **21l**

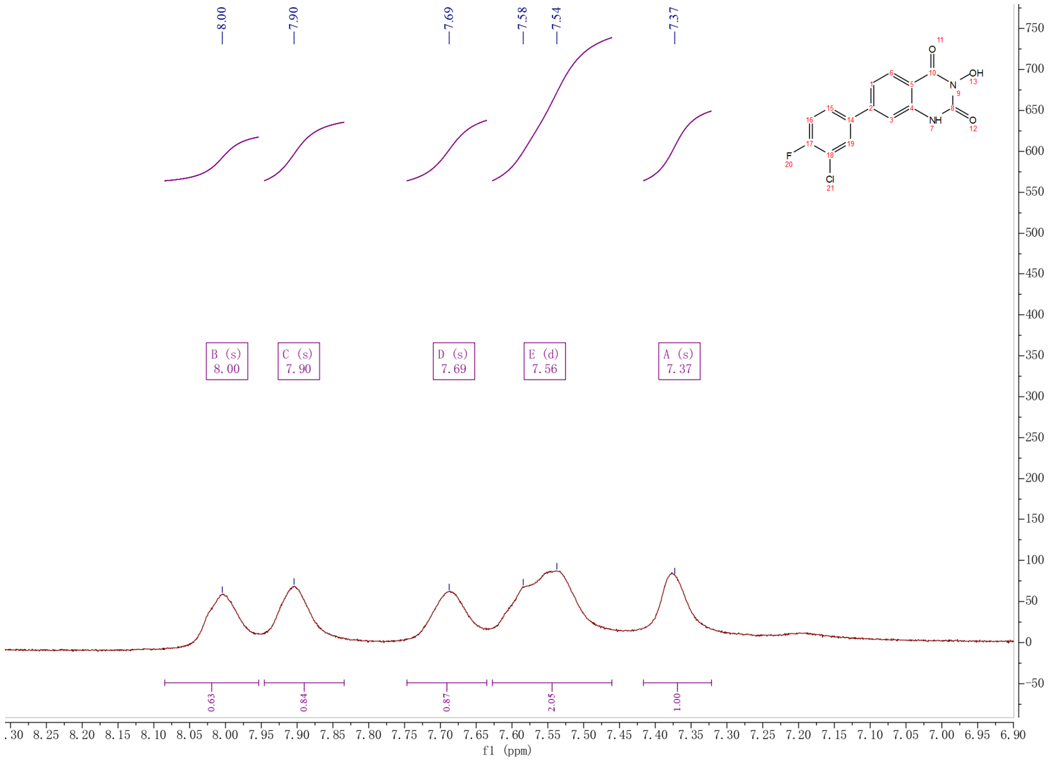

Figure S107. Magnified ^{1}H NMR (400 MHz, DMSO-d_6) spectrum fragments of **21l**

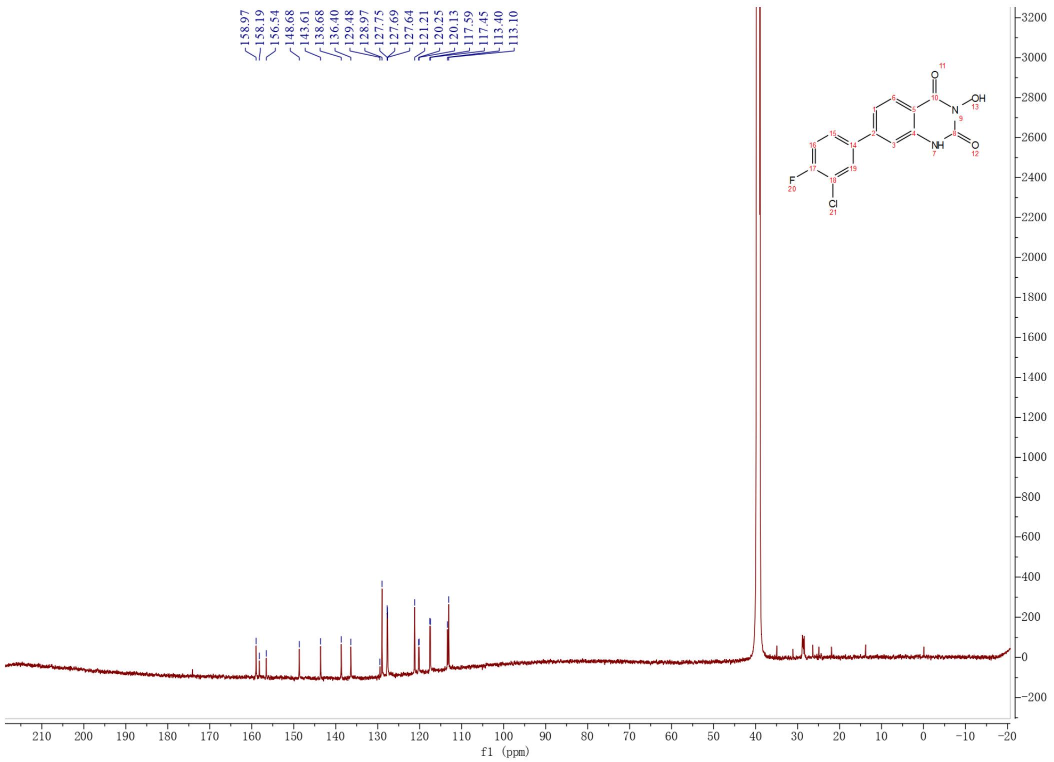

Figure S108. ^{13}C NMR (151 MHz, DMSO-d_6) spectrum of **21l**

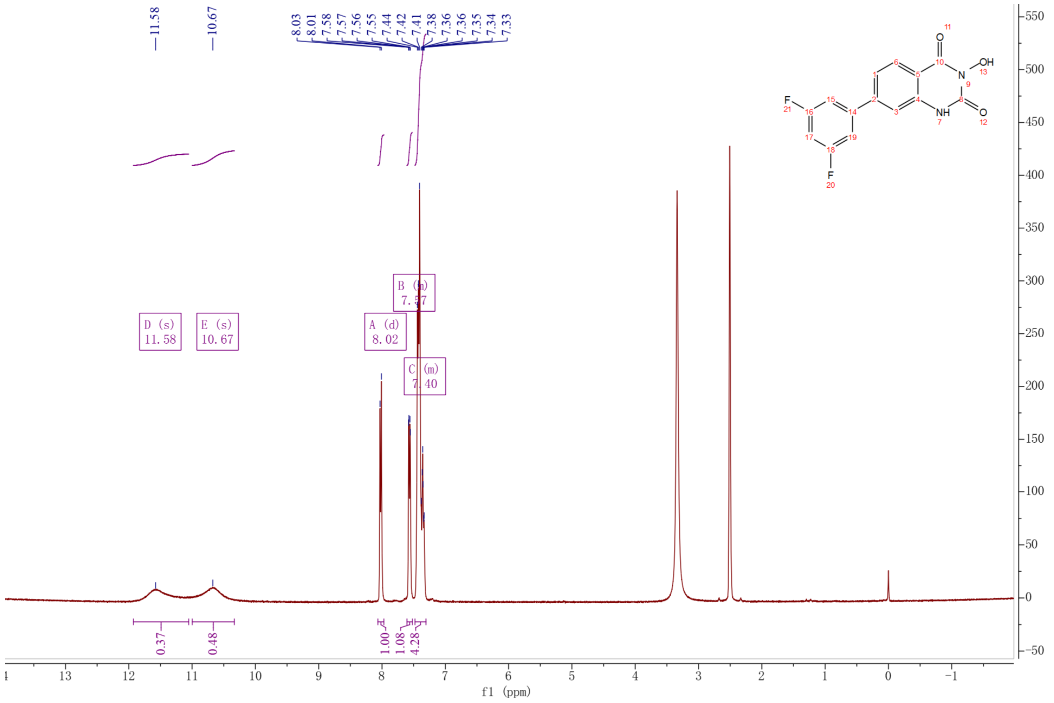

Figure S109. ^{1}H NMR (400 MHz, DMSO-d_6) spectrum of **21m**

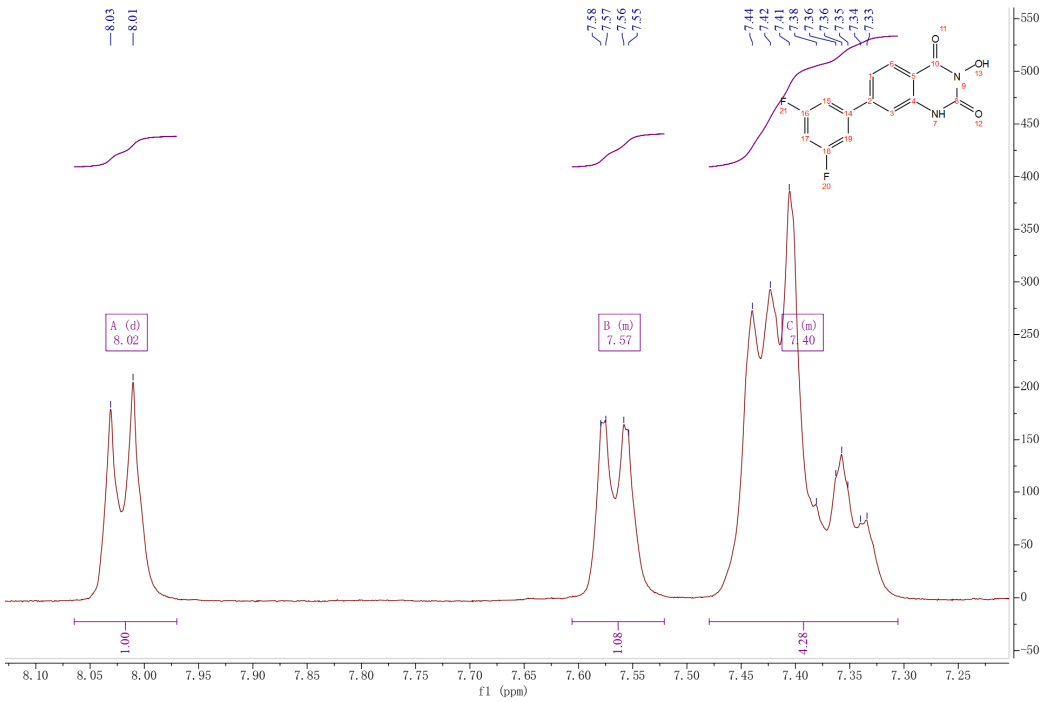

Figure S110. Magnified ^{1}H NMR (400 MHz, DMSO-d_6) spectrum fragments of **21m**

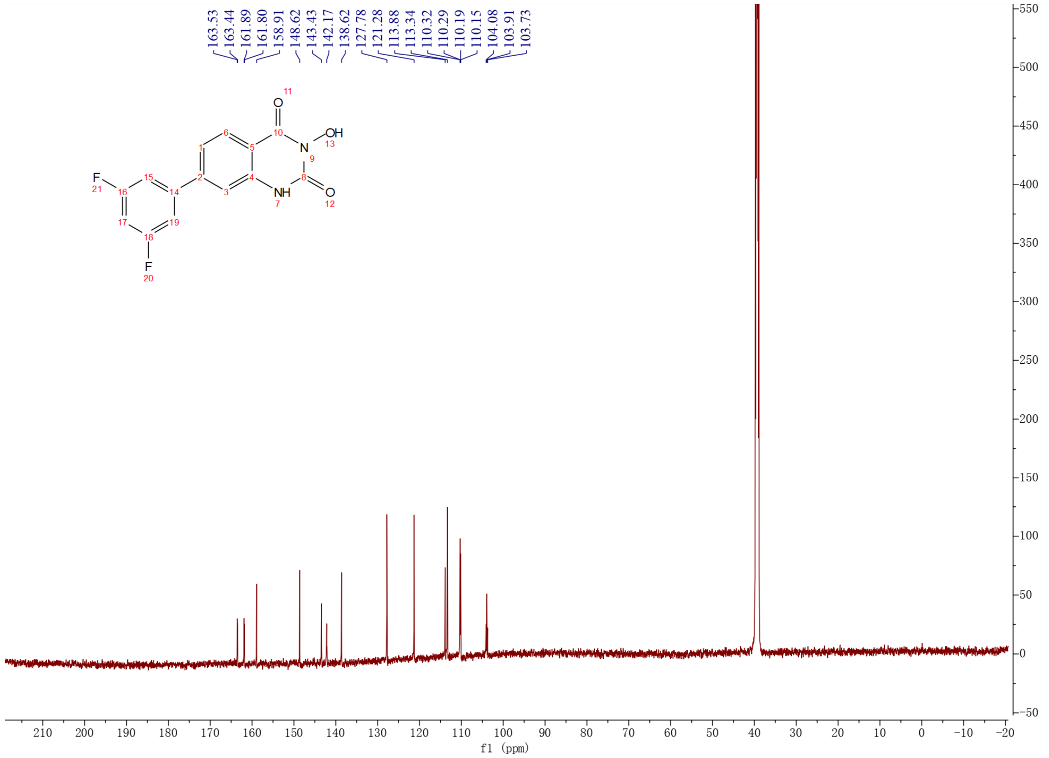

Figure S111. ^{13}C NMR (151 MHz, DMSO-d_6) spectrum of **21m**

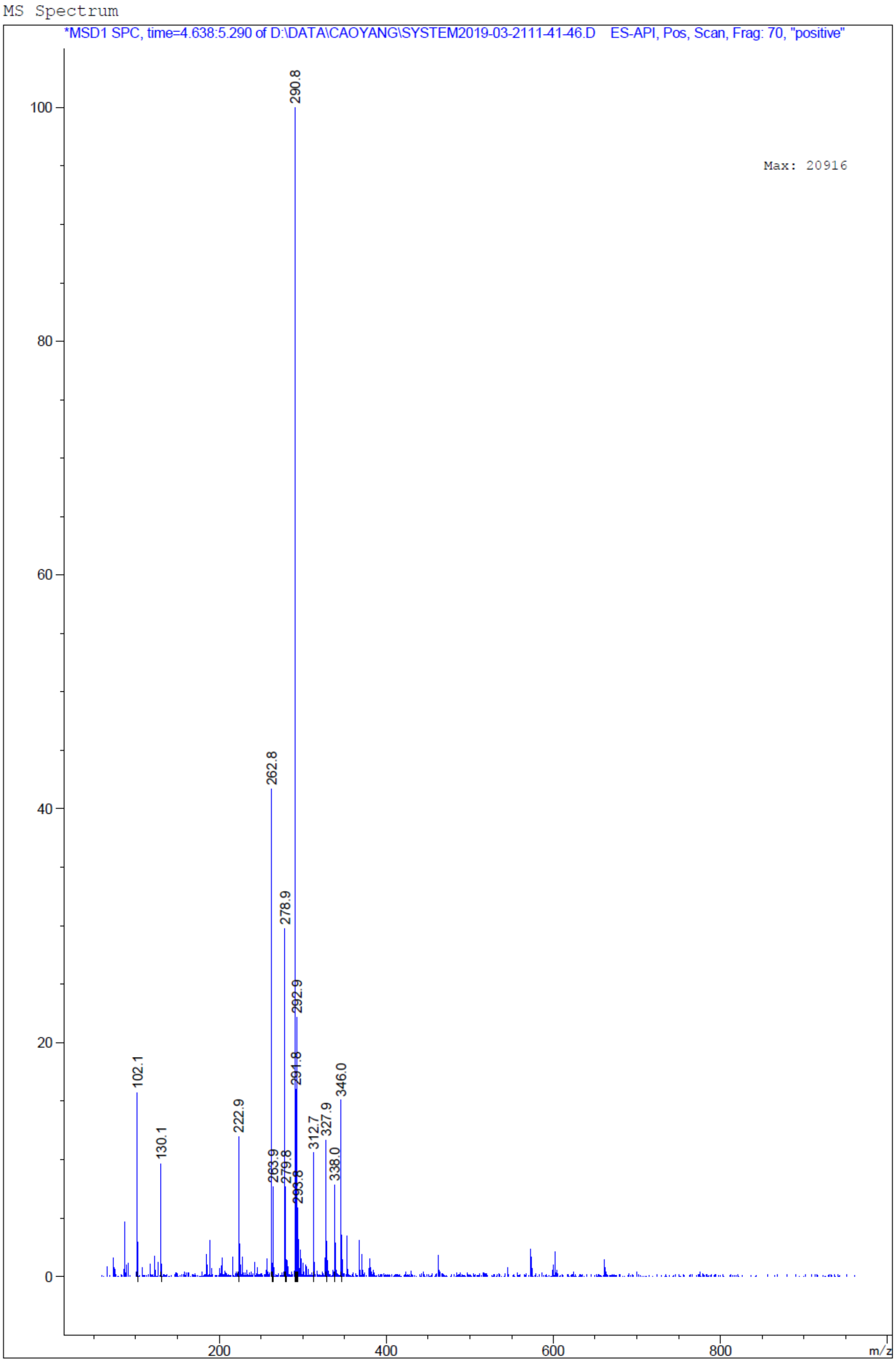

Figure S112. Mass spectrum (positive ionization) of **21m**

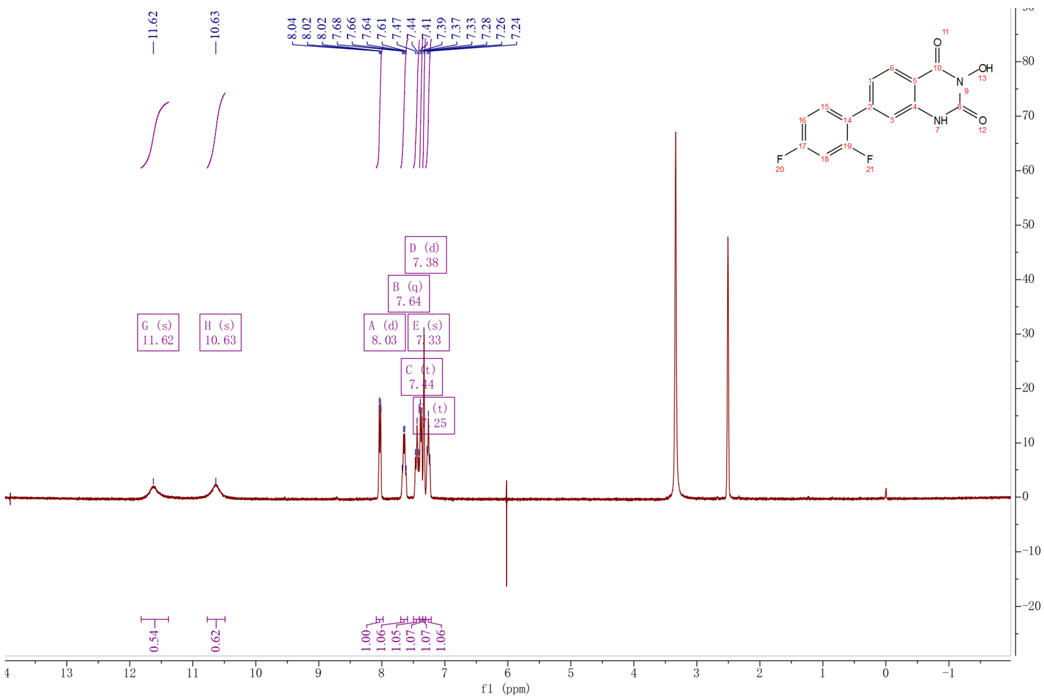

Figure S113. ^{1}H NMR (400 MHz, DMSO-d_6) spectrum of **21n**

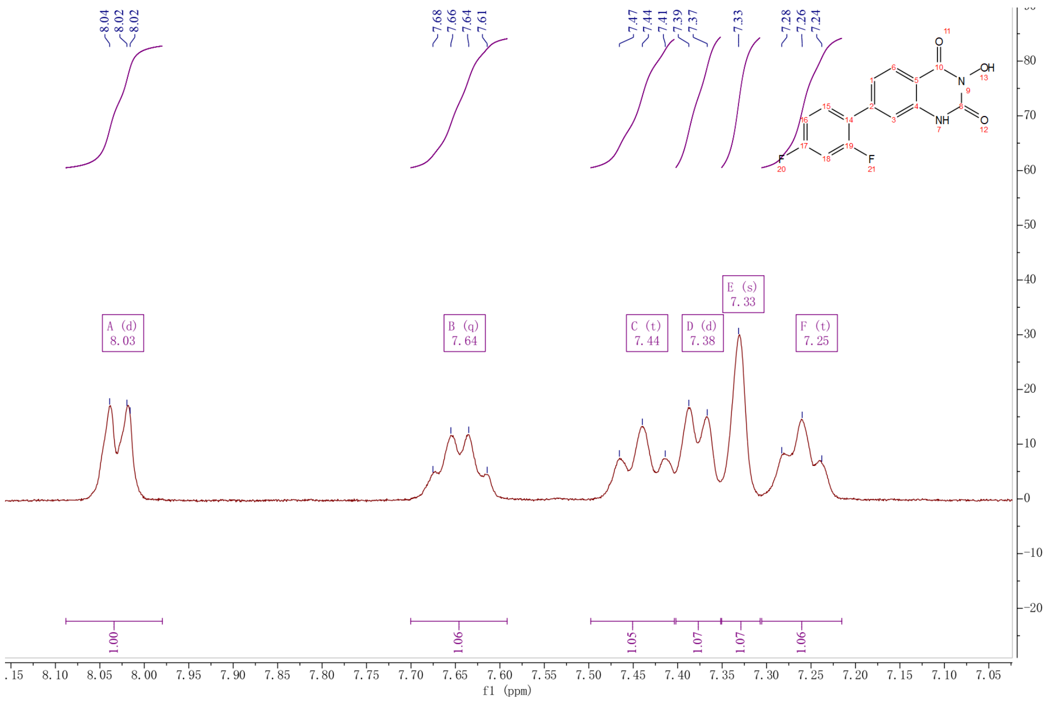

Figure S114. Magnified ^{1}H NMR (400 MHz, DMSO-d_6) spectrum fragments of **21n**

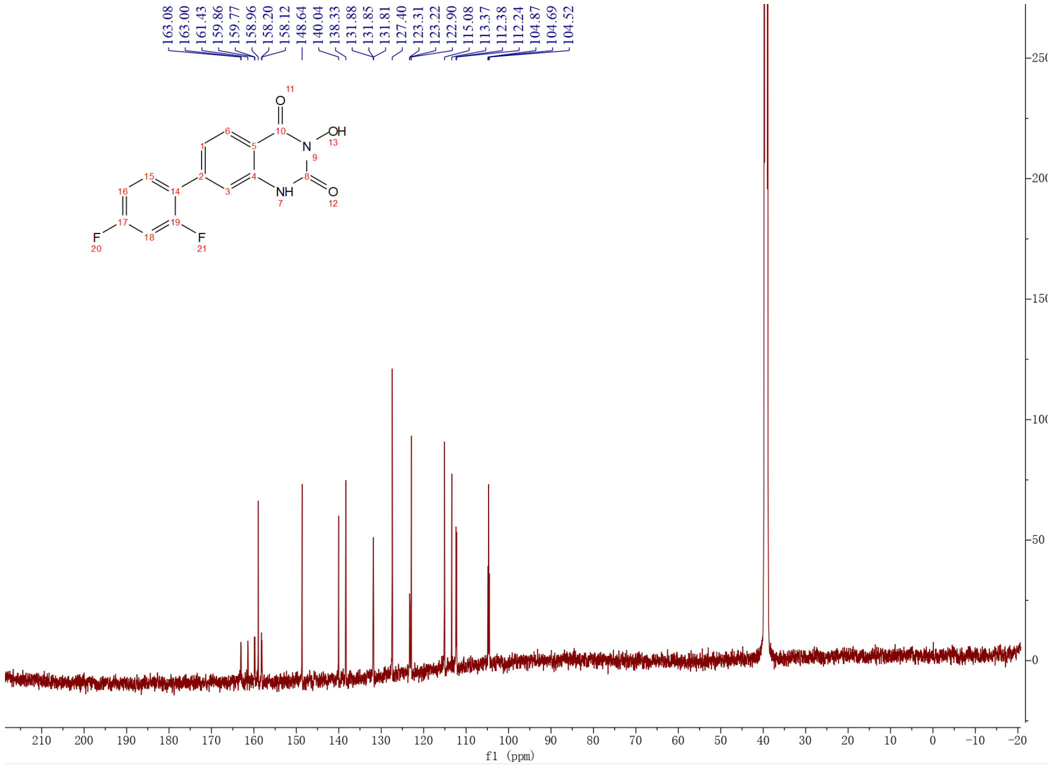

Figure S115. ^{13}C NMR (151 MHz, DMSO-d_6) spectrum of **21n**

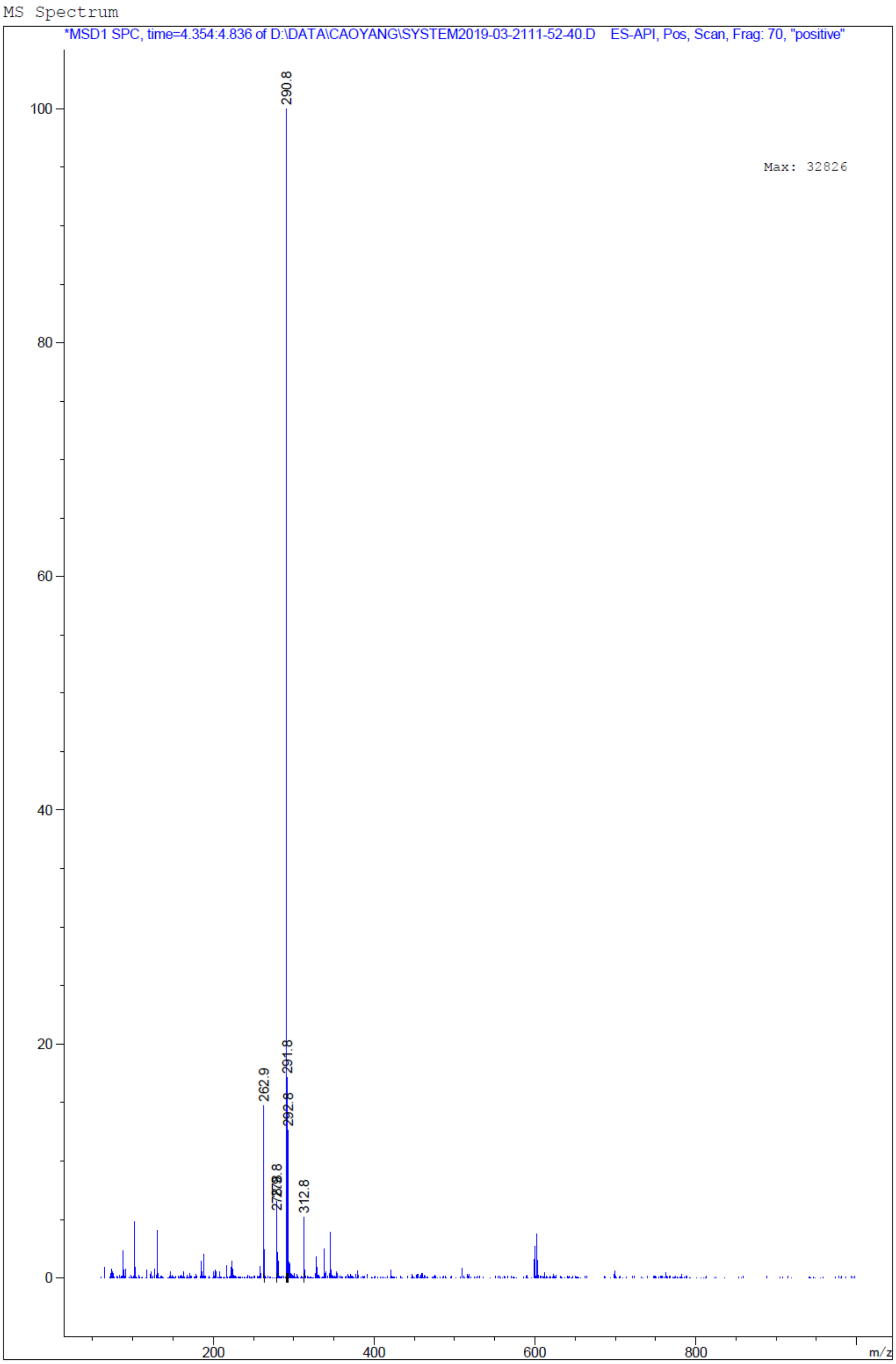

Figure S116. Mass spectrum (positive ionization) of **21n**

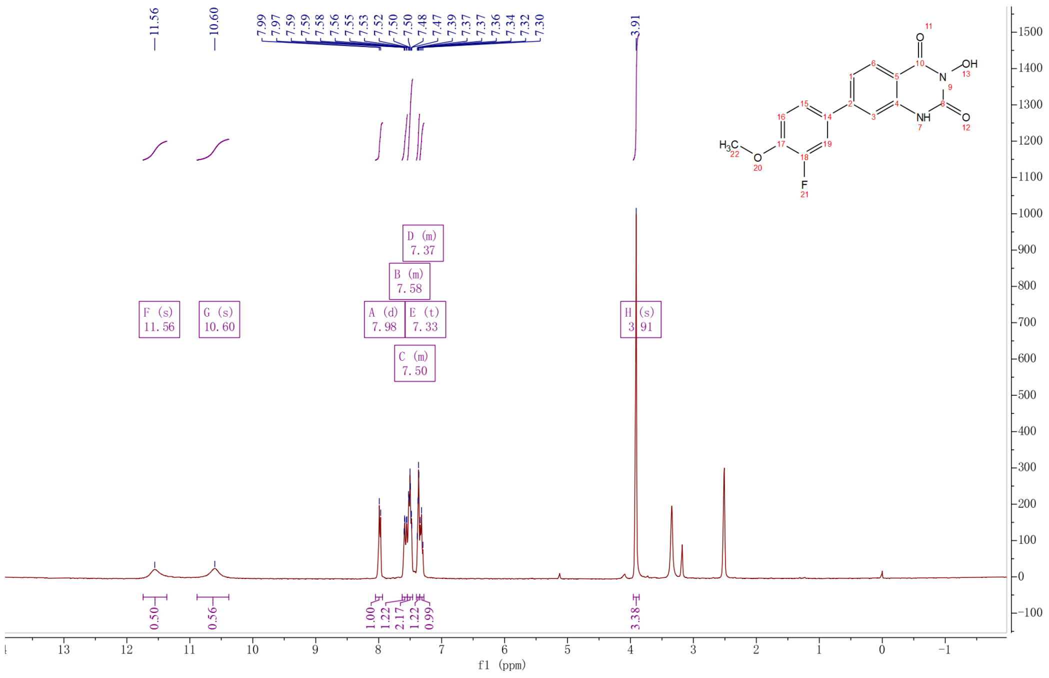

Figure S117. ^{1}H NMR (400 MHz, DMSO-d_6) spectrum of **21o**

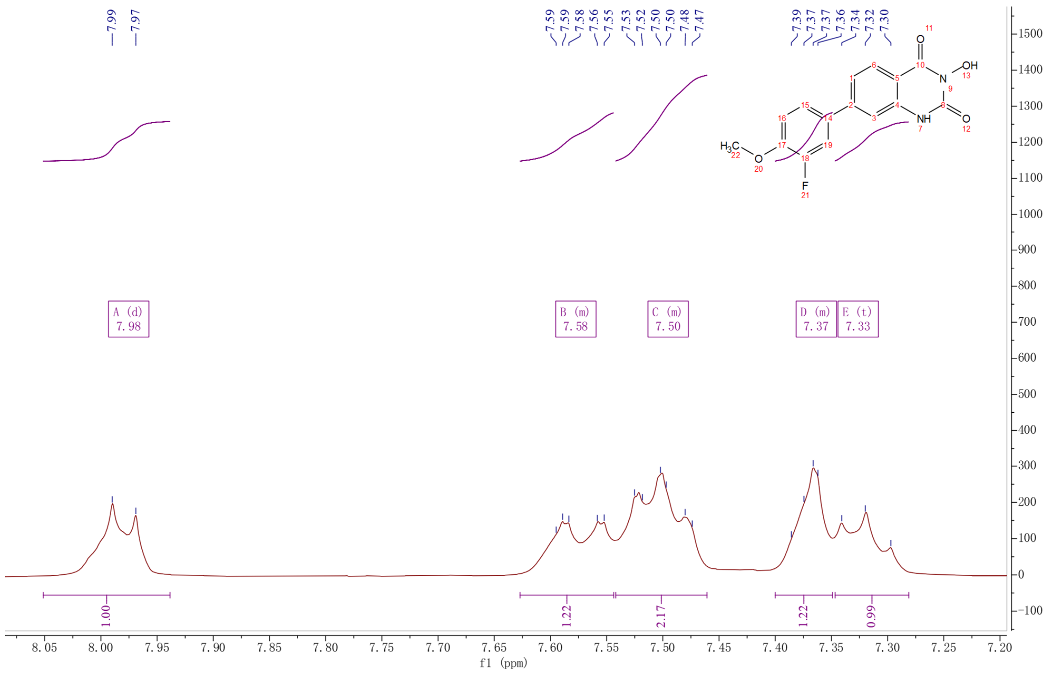

Figure S118. Magnified ^{1}H NMR (400 MHz, DMSO-d_6) spectrum fragments of **21o**

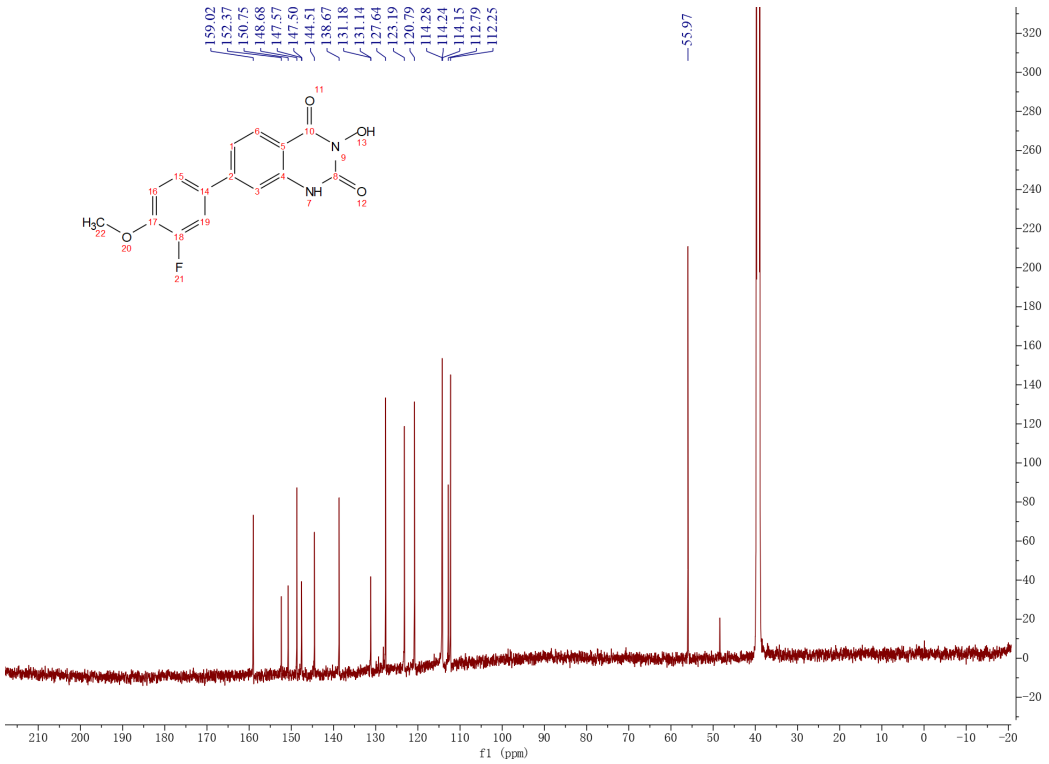

Figure S119. ^{13}C NMR (151 MHz, DMSO-d_6) spectrum of **21o**

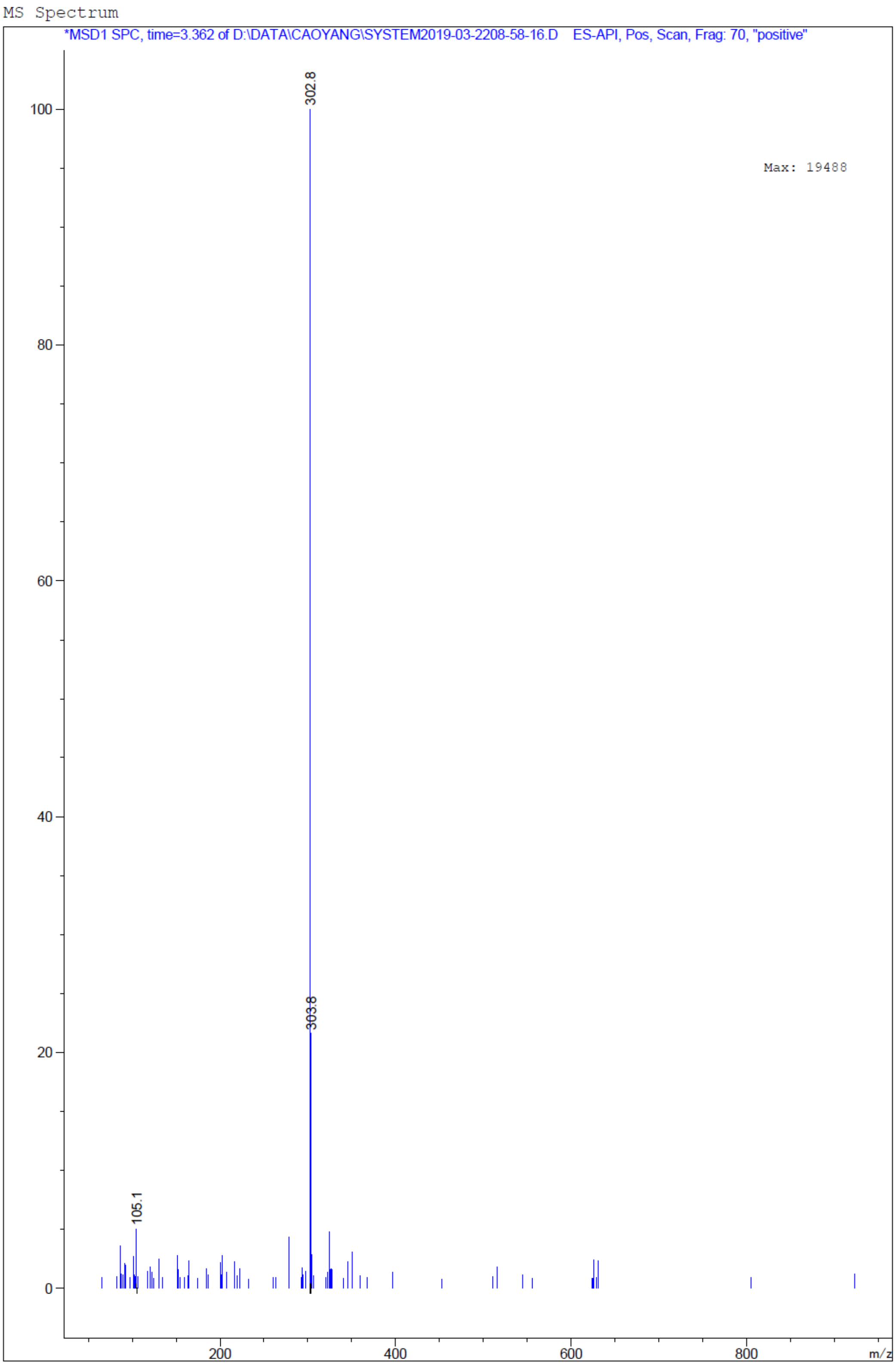

Figure S120. Mass spectrum (positive ionization) of **21o**

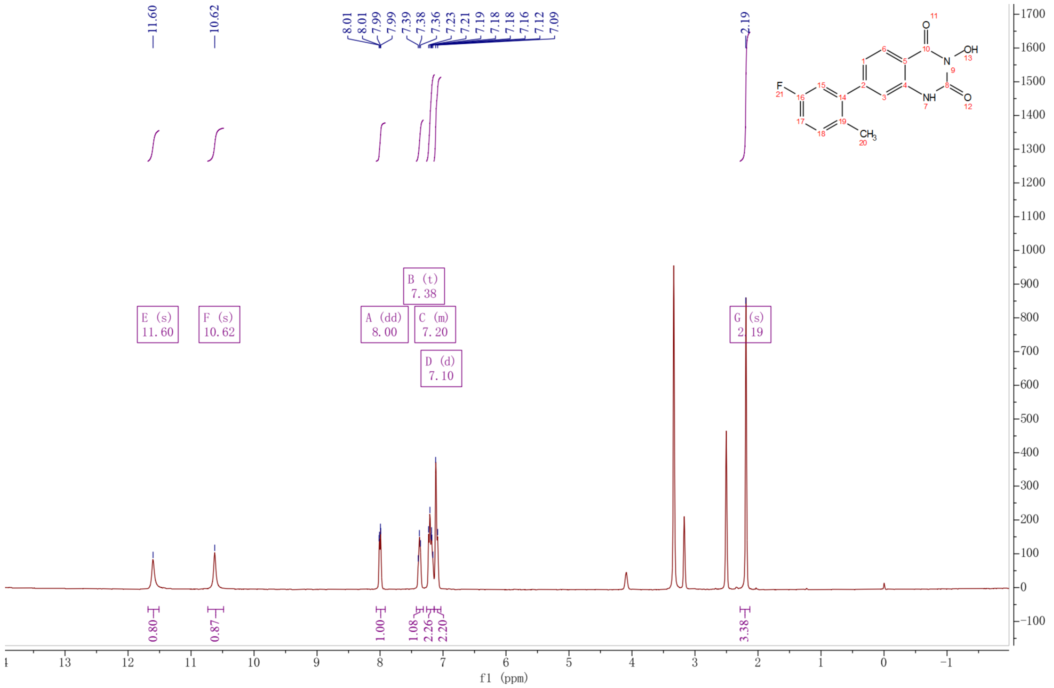

Figure S121. ^{1}H NMR (400 MHz, DMSO-d_6) spectrum of **21p**

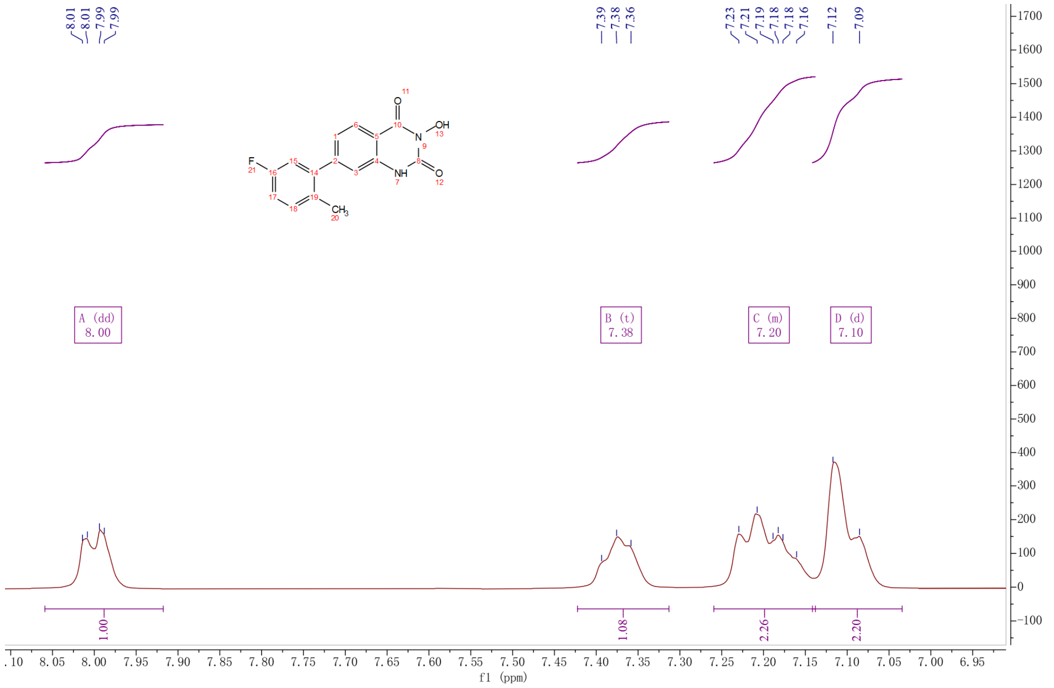

Figure S122. Magnified ^{1}H NMR (400 MHz, DMSO-d_6) spectrum fragments of **21p**

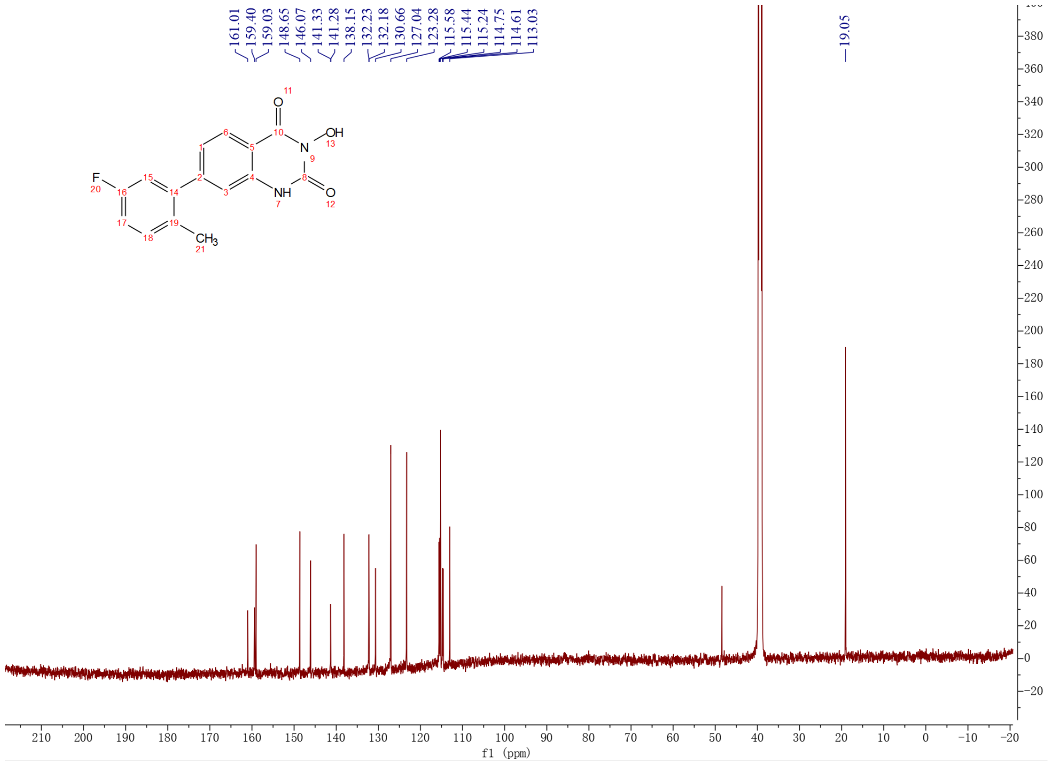

Figure S123. ^{13}C NMR (151 MHz, DMSO-d_6) spectrum of **21p**

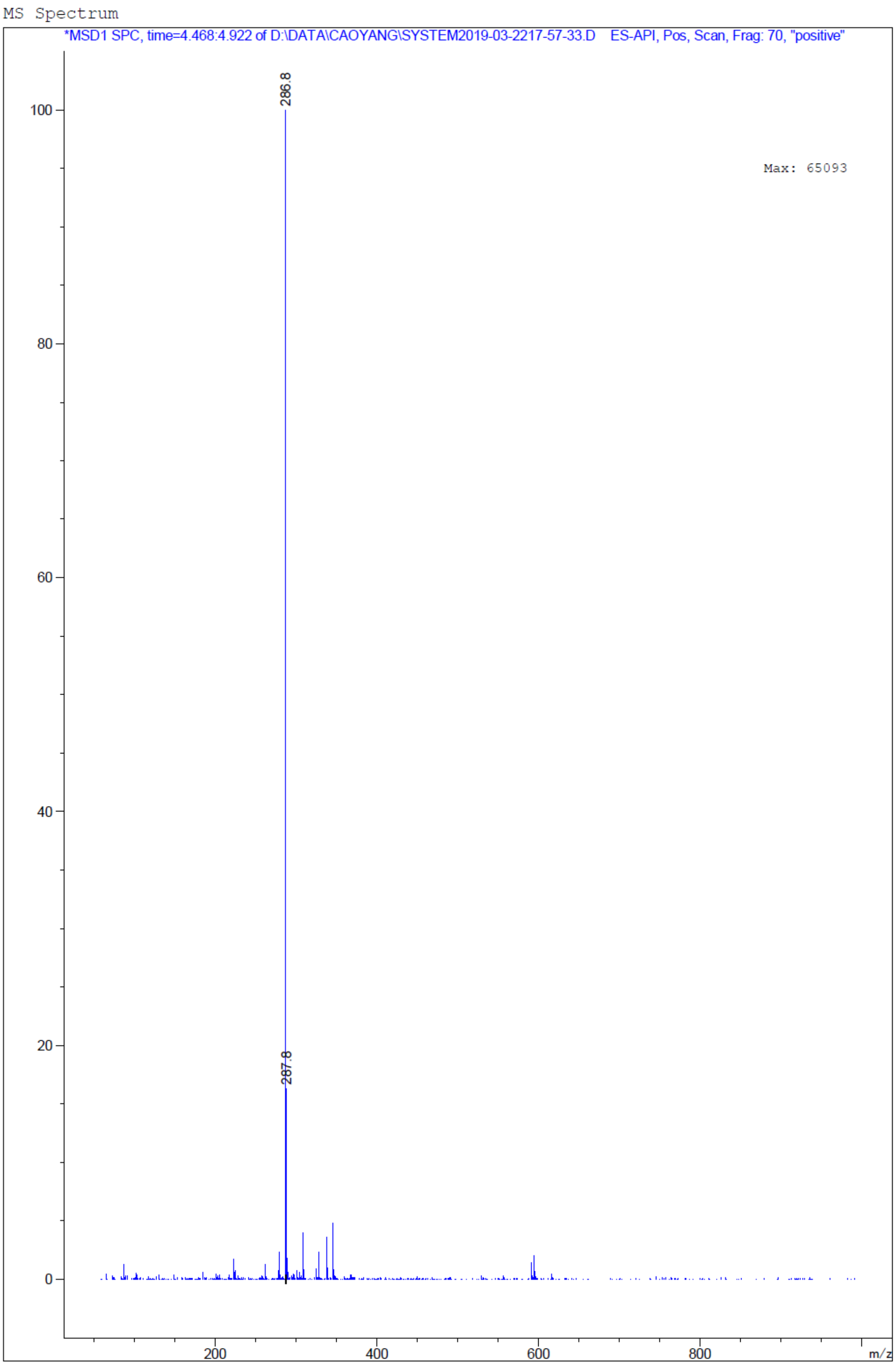

Figure S124. Mass spectrum (positive ionization) of **21p**

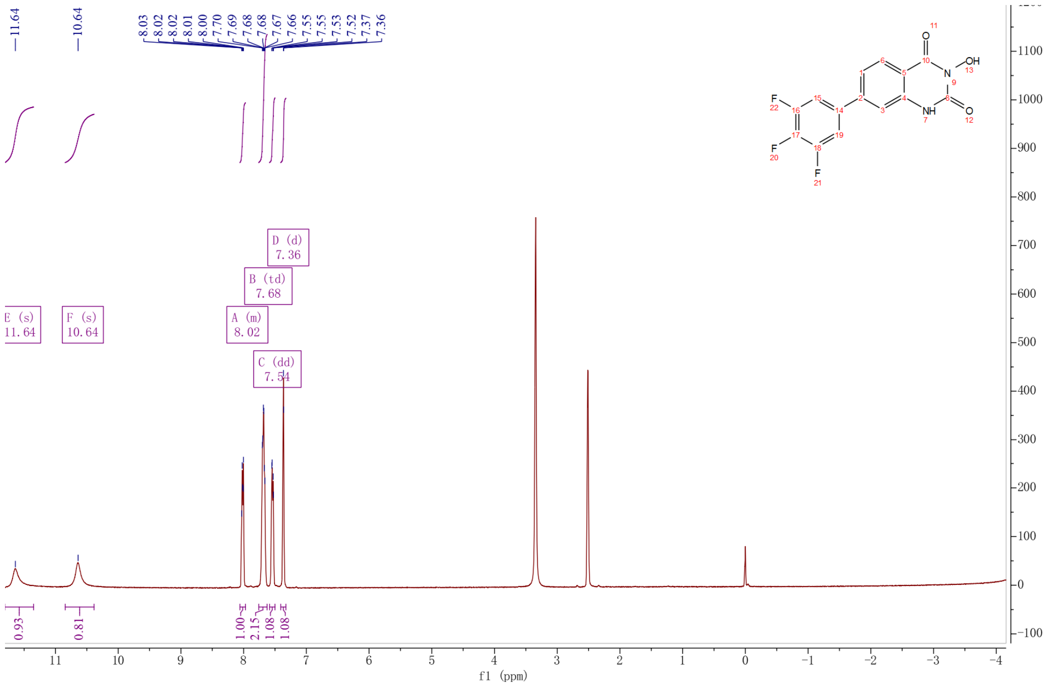

Figure S125. ^{1}H NMR (400 MHz, DMSO-d_6) spectrum of **21q**

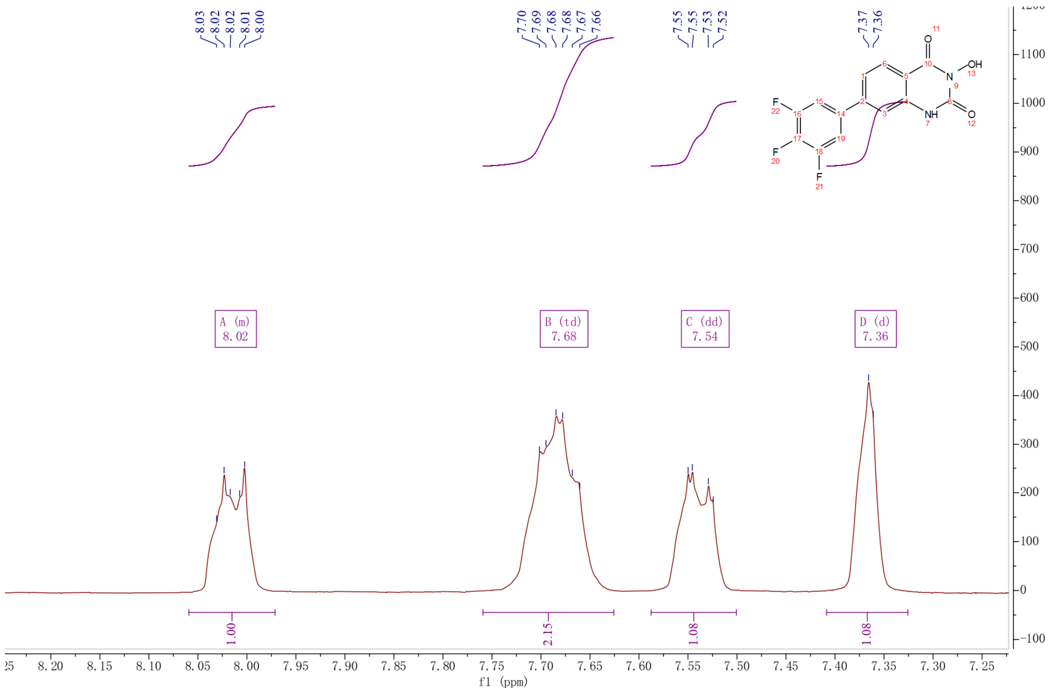

Figure S126. Magnified ^{1}H NMR (400 MHz, DMSO-d_6) spectrum fragments of **21q**

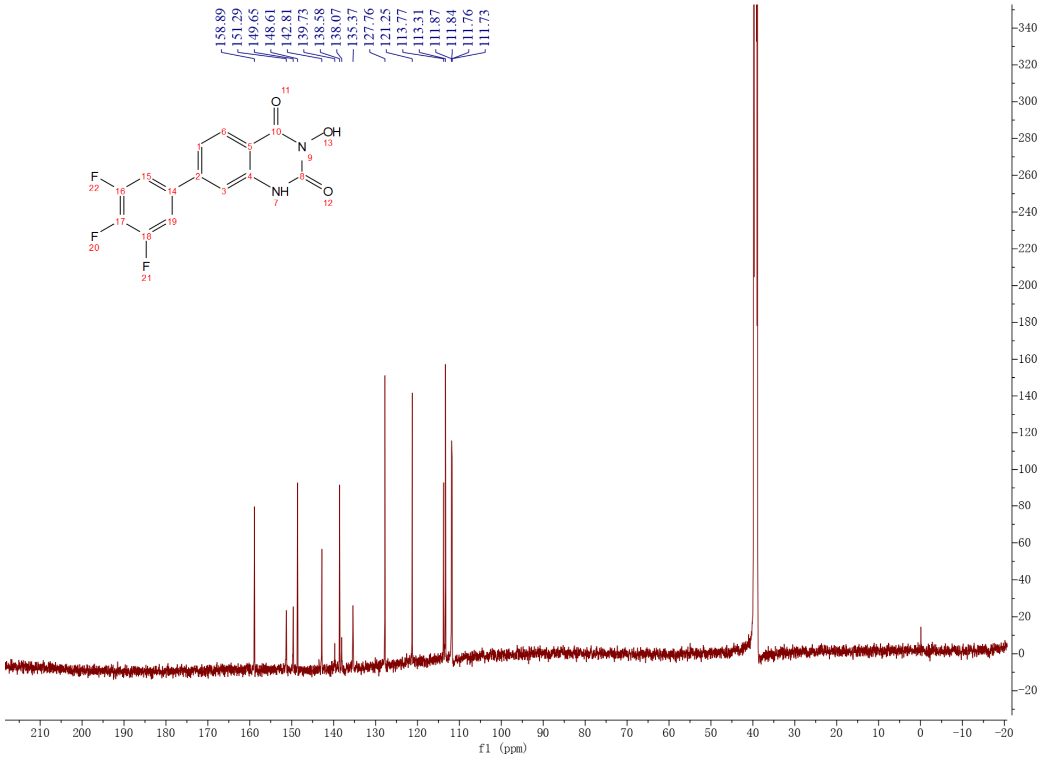

Figure S127. ^{13}C NMR (151 MHz, DMSO-d_6) spectrum of **21q**

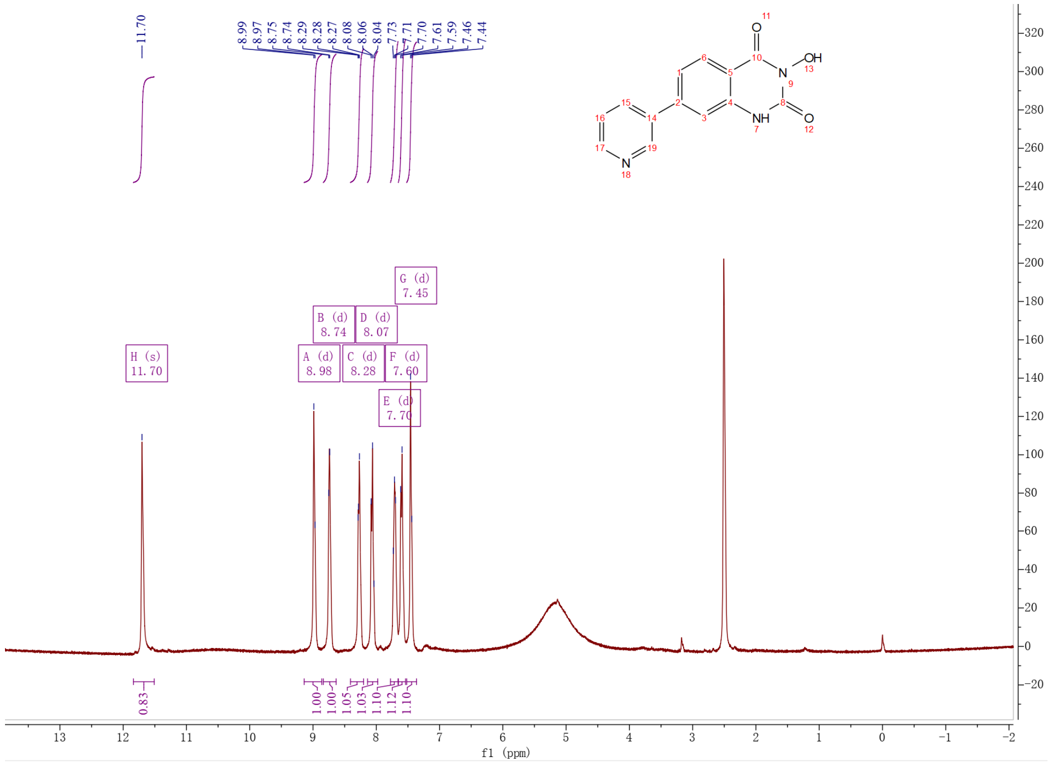

Figure S128. ^{1}H NMR (400 MHz, DMSO-d_6) spectrum of **21r**

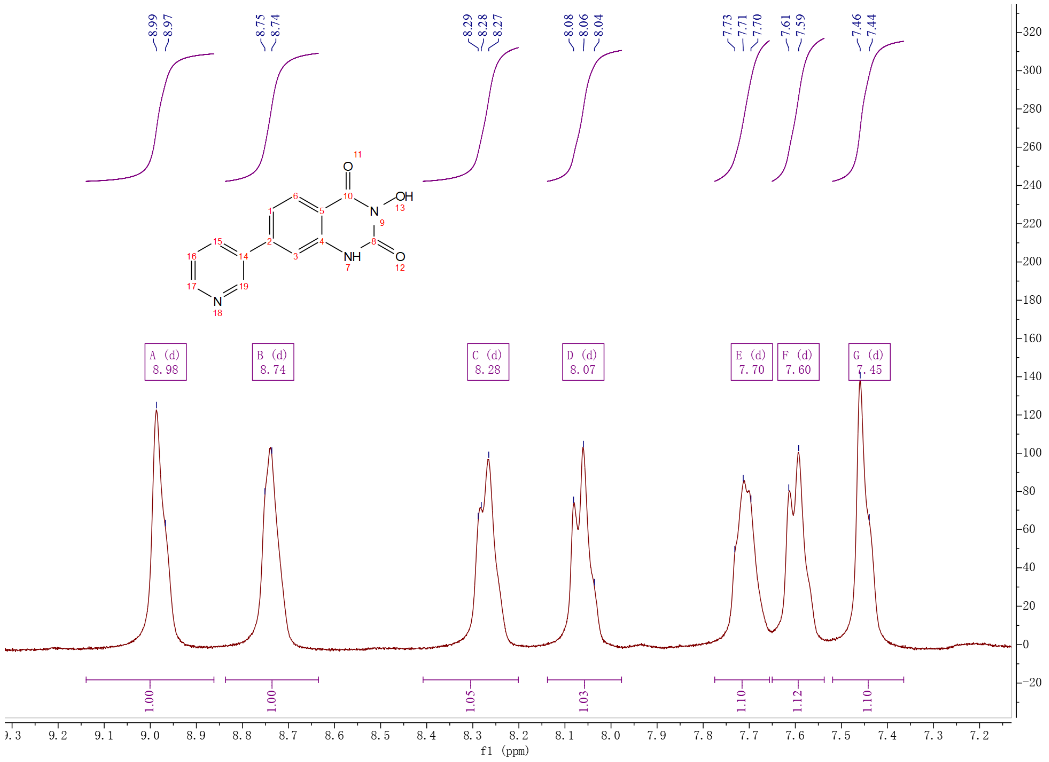

Figure S129. Magnified ^{1}H NMR (400 MHz, DMSO-d_6) spectrum fragments of **21r**

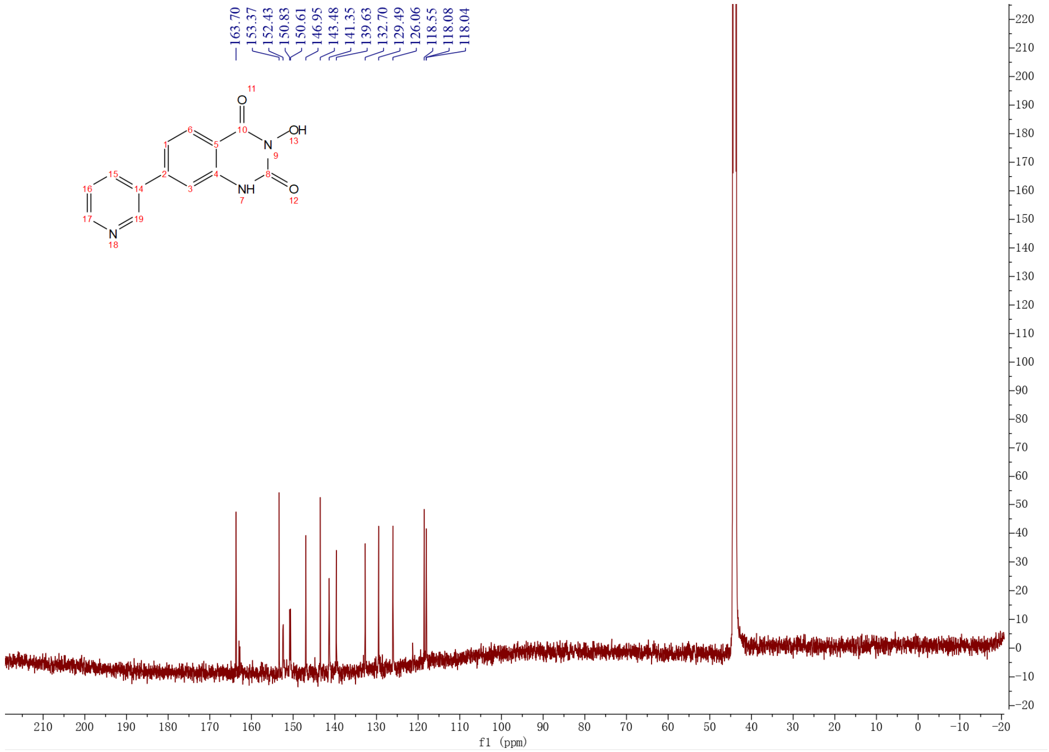

Figure S130. ^{13}C NMR (151 MHz, DMSO-d_6) spectrum of **21r**

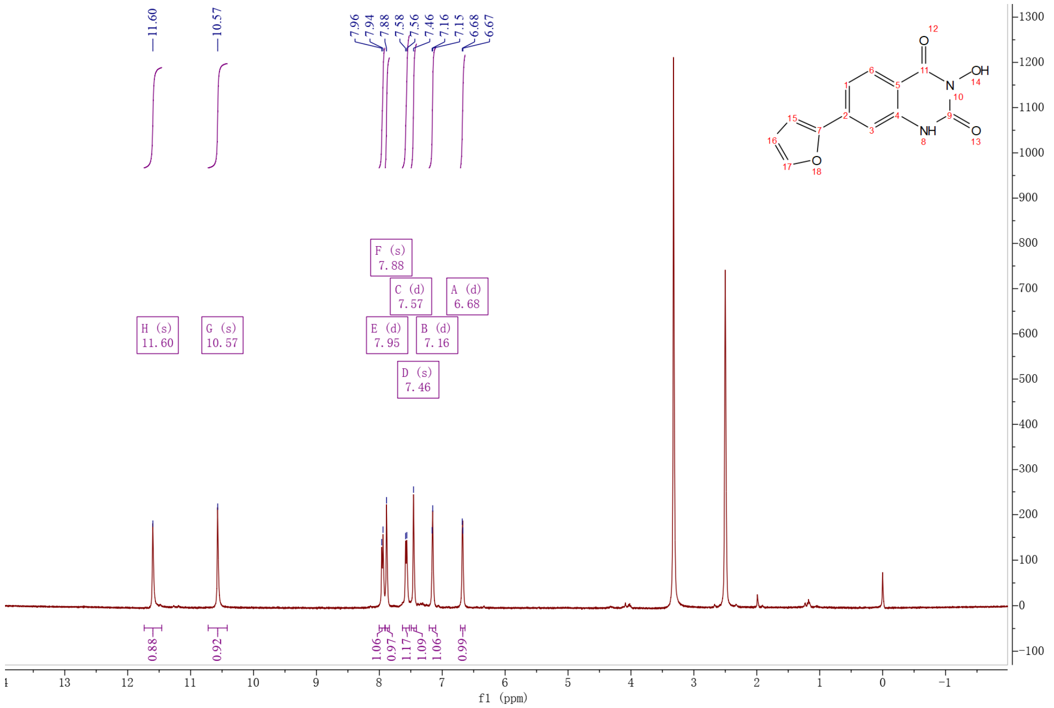

Figure S131. ^{1}H NMR (400 MHz, DMSO-d_6) spectrum of **21s**

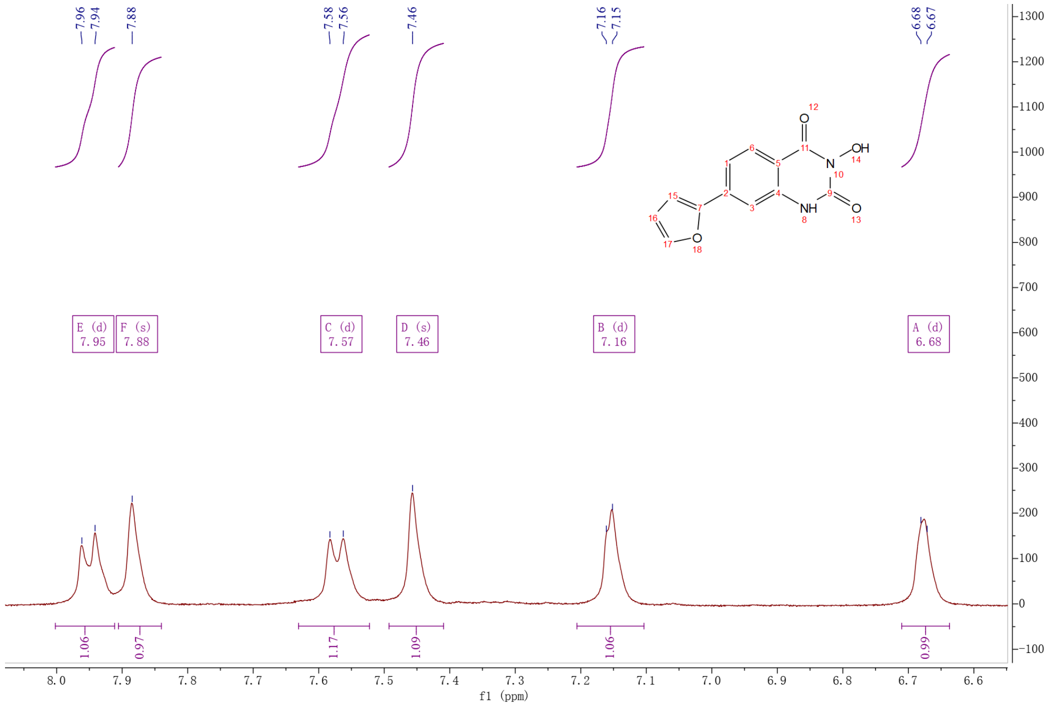

Figure S132. Magnified ^{1}H NMR (400 MHz, DMSO-d_6) spectrum fragments of **21s**

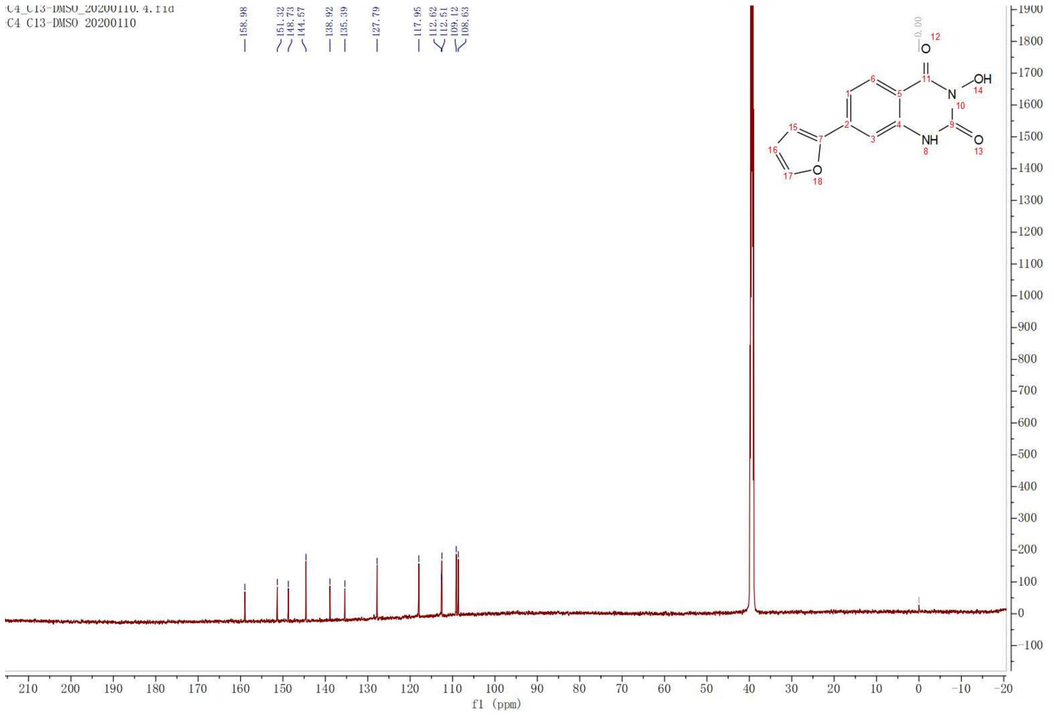

Figure S133. ^{13}C NMR (151 MHz, DMSO-d_6) spectrum of **21s**

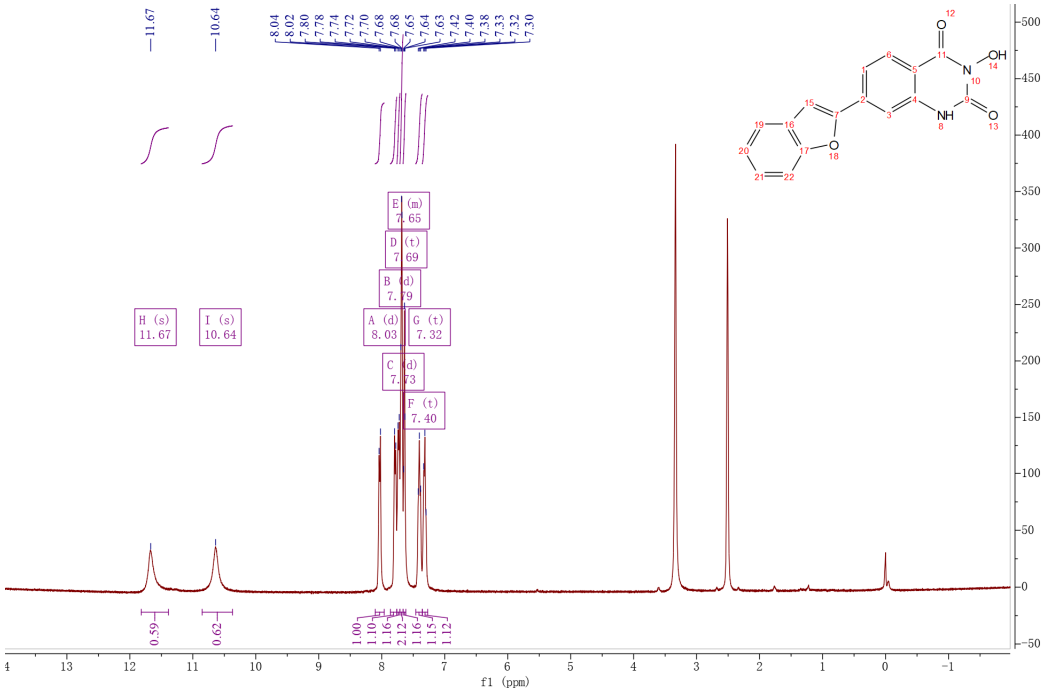

Figure S134. ^{1}H NMR (400 MHz, DMSO-d_6) spectrum of **21t**

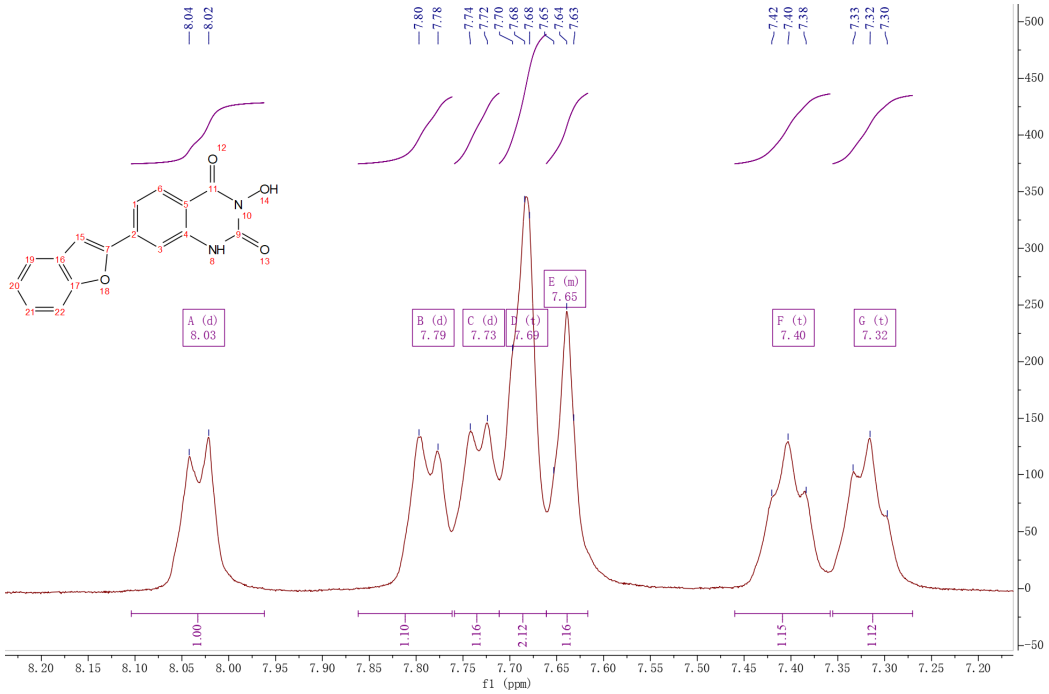

Figure S135. Magnified ^{1}H NMR (400 MHz, DMSO-d_6) spectrum fragments of **21t**

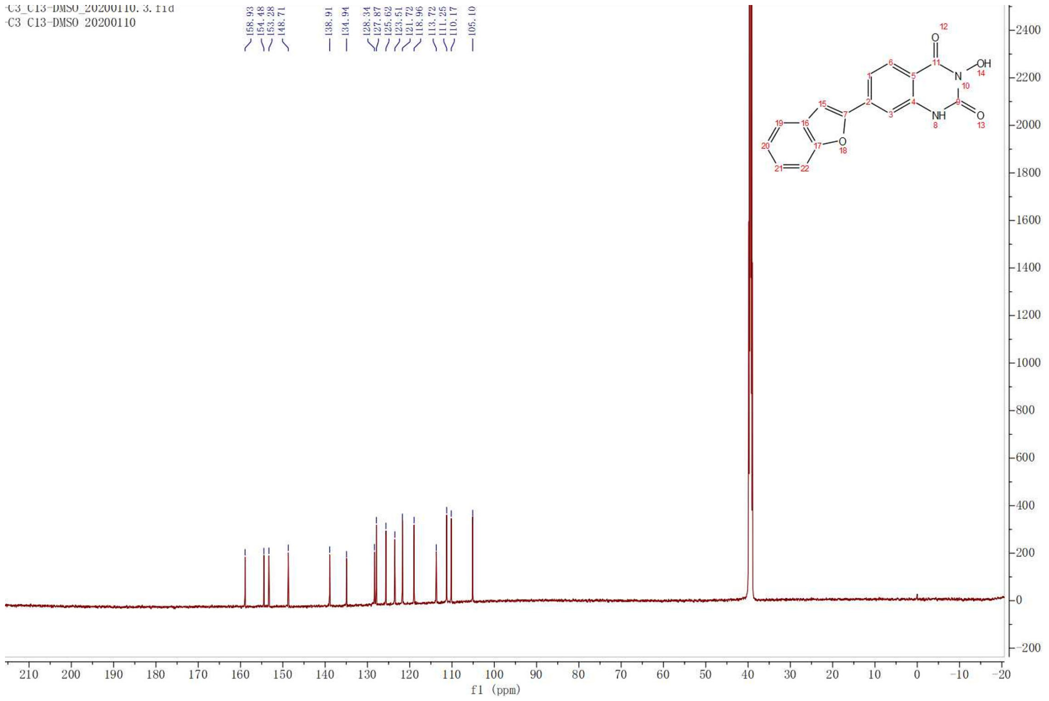

Figure S136. ^{13}C NMR (151 MHz, DMSO-d_6) spectrum of **21t**

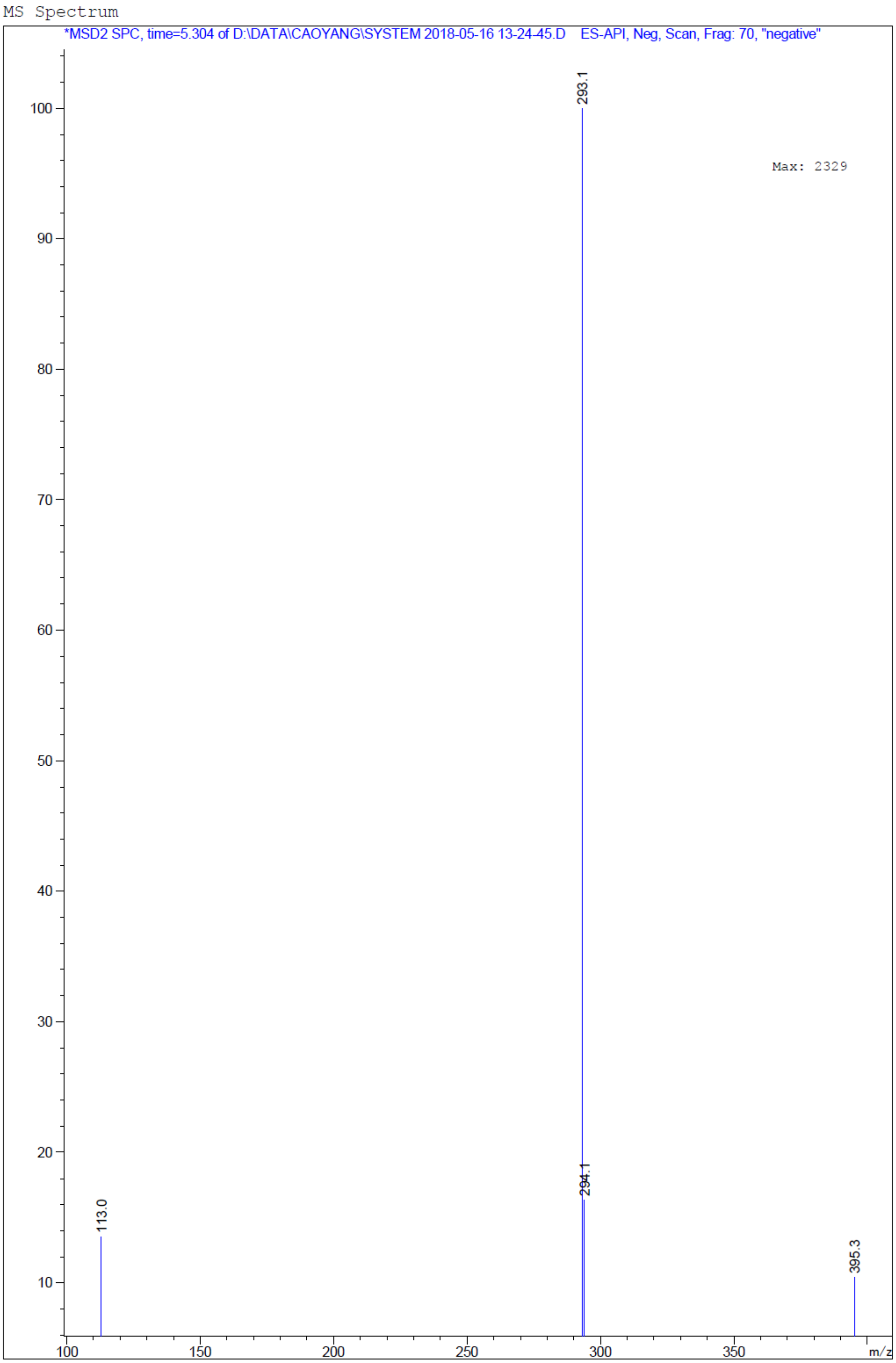

Figure S137. Mass spectrum (negative ionization) of **21t**

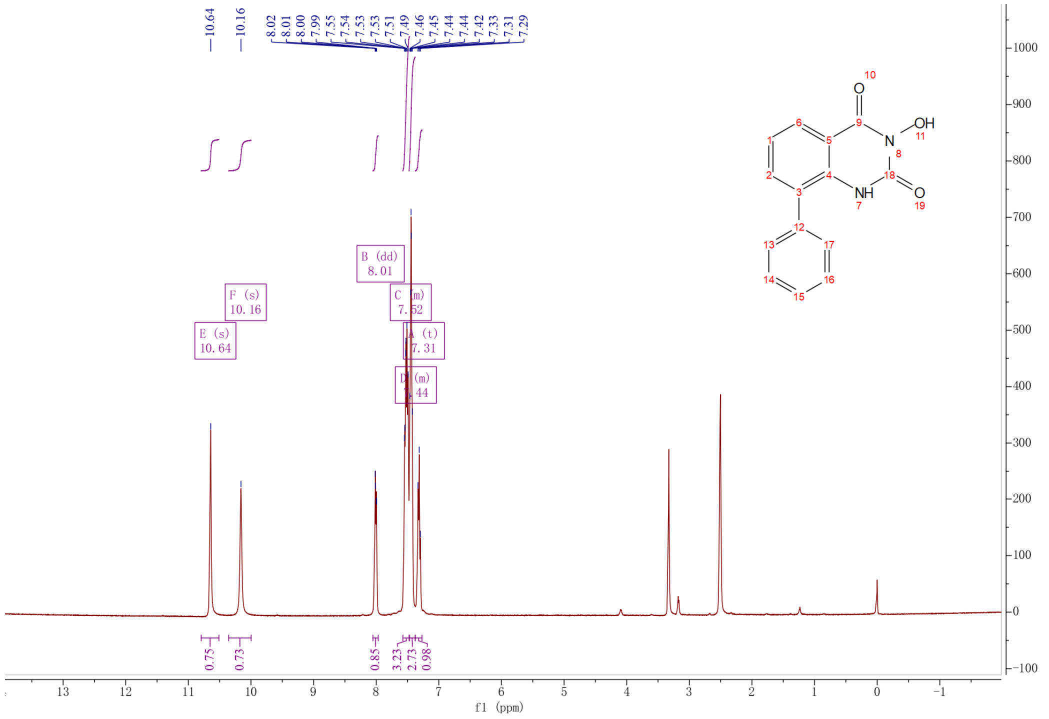

Figure S138. ^{1}H NMR (400 MHz, DMSO-d_6) spectrum of **21u**

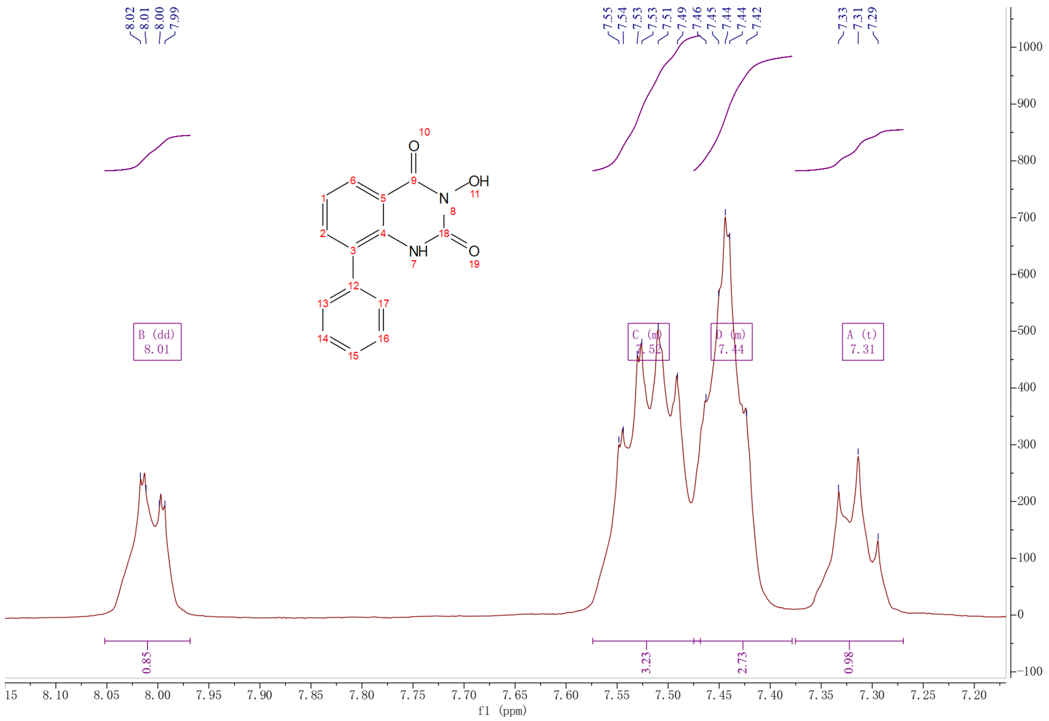

Figure S139. Magnified ^{1}H NMR (400 MHz, DMSO-d_6) spectrum fragments of **21u**

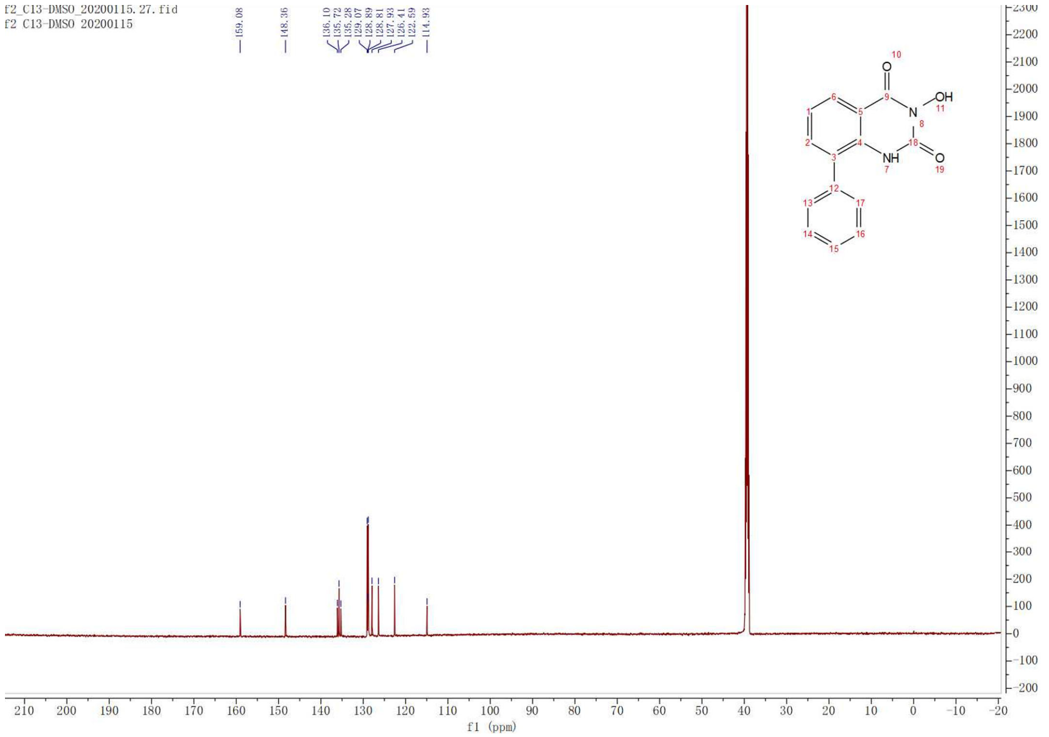

Figure S140. ^{13}C NMR (151 MHz, DMSO-d_6) spectrum of **21u**

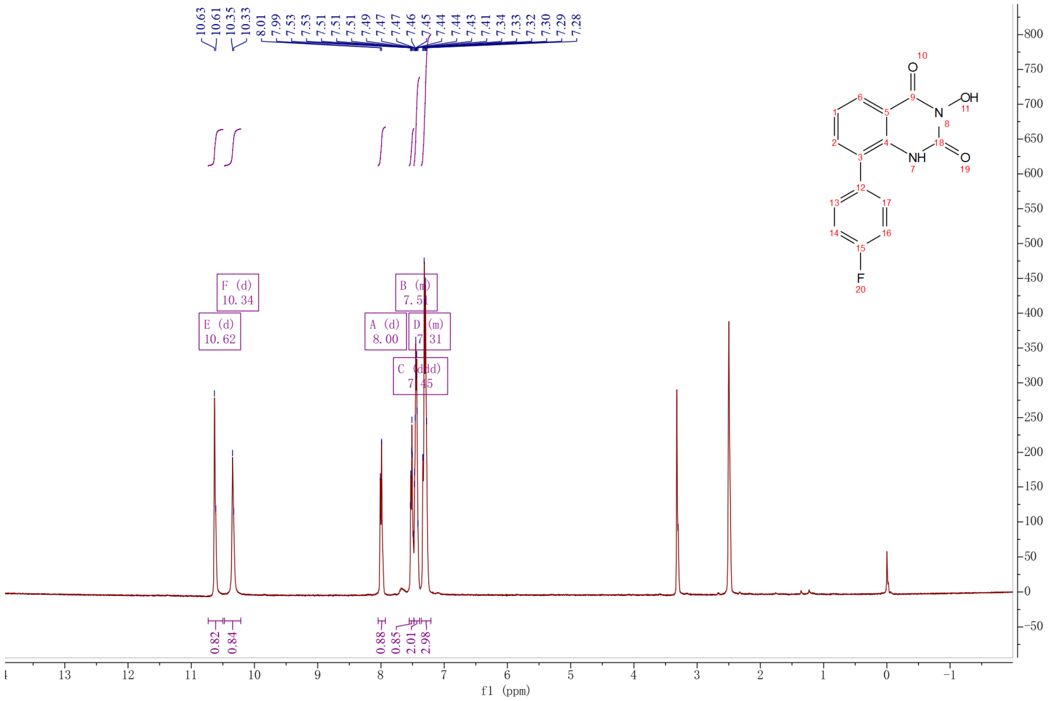

Figure S141. ^{1}H NMR (400 MHz, DMSO-d_6) spectrum of **21v**

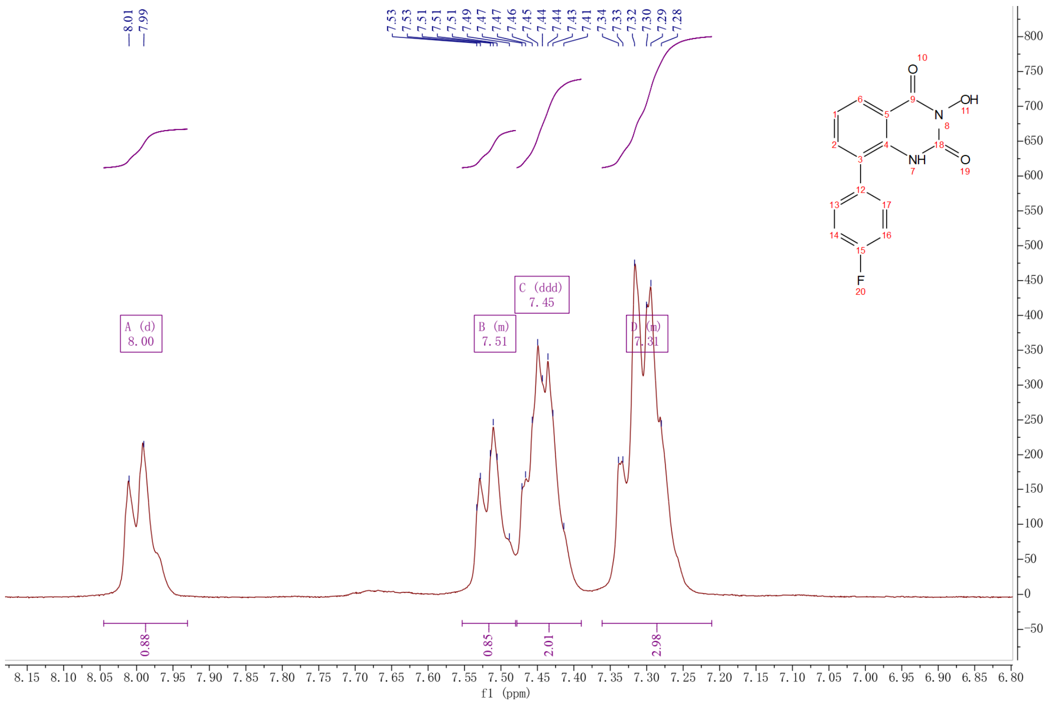

Figure S142. Magnified ^{1}H NMR (400 MHz, DMSO-d_6) spectrum fragments of **21v**

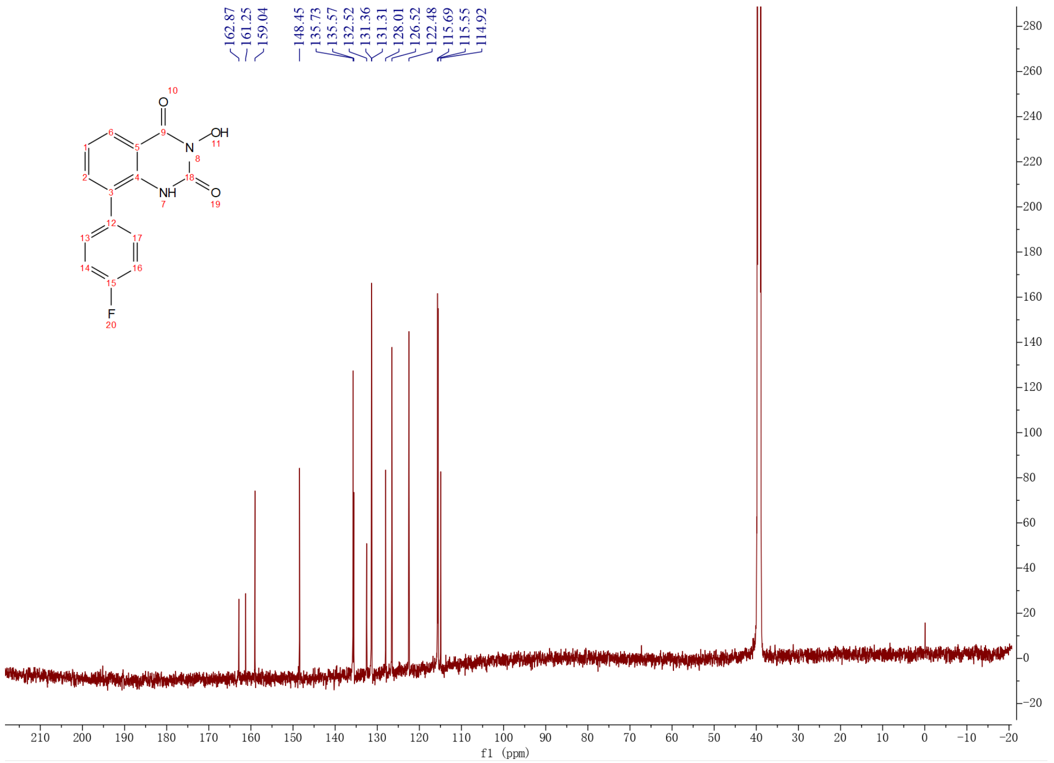

Figure S143. ^{13}C NMR (151 MHz, DMSO-d_6) spectrum of **21v**

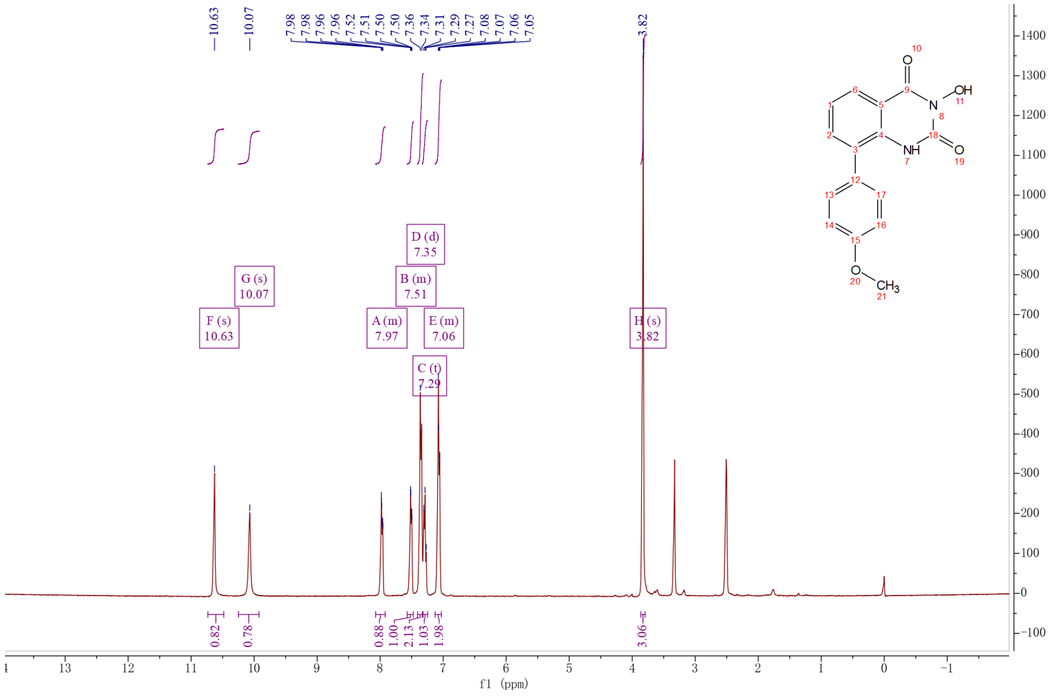

Figure S144. ^{1}H NMR (400 MHz, DMSO-d_6) spectrum of **21w**

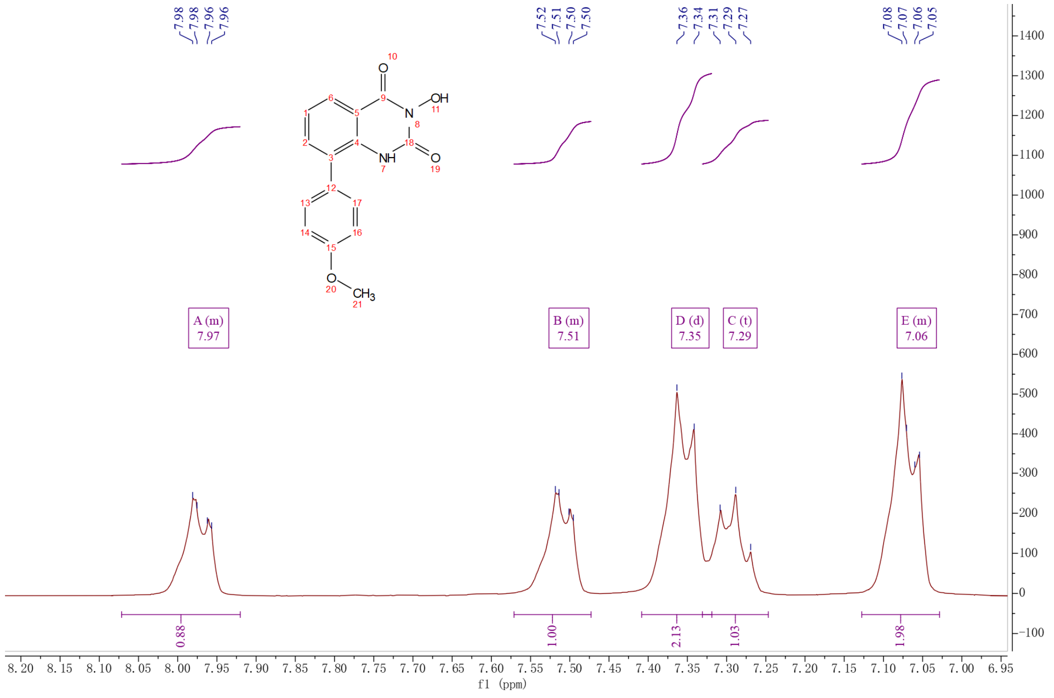

Figure S145. Magnified ^{1}H NMR (400 MHz, DMSO-d_6) spectrum fragments of **21w**

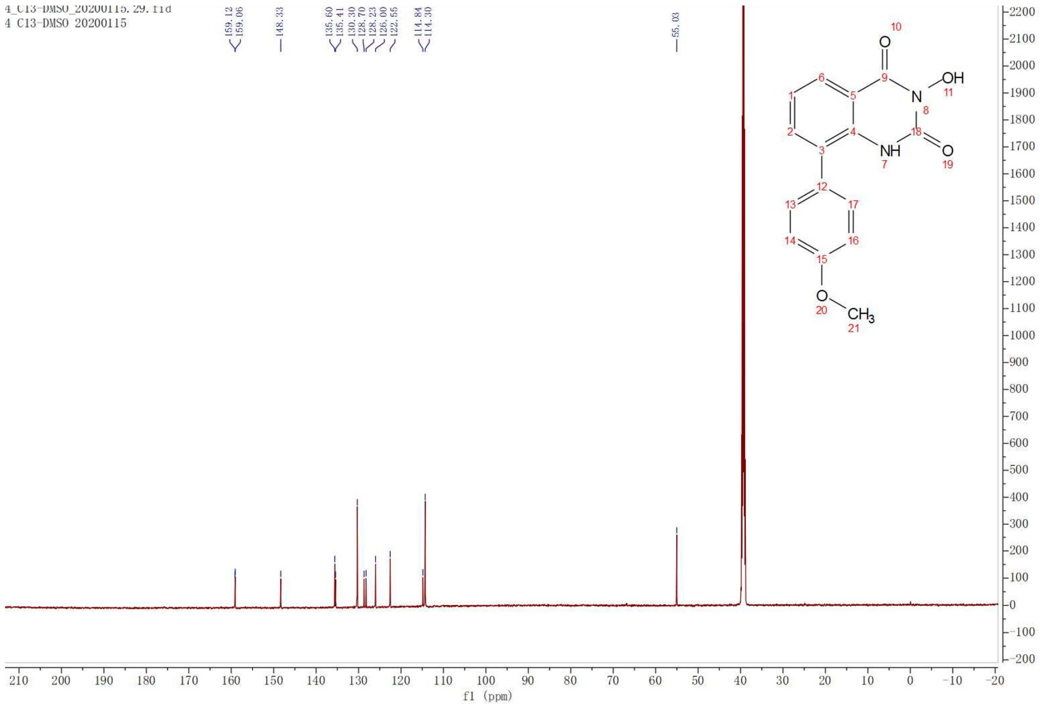

Figure S146. ^{13}C NMR (151 MHz, DMSO-d_6) spectrum of **21w**

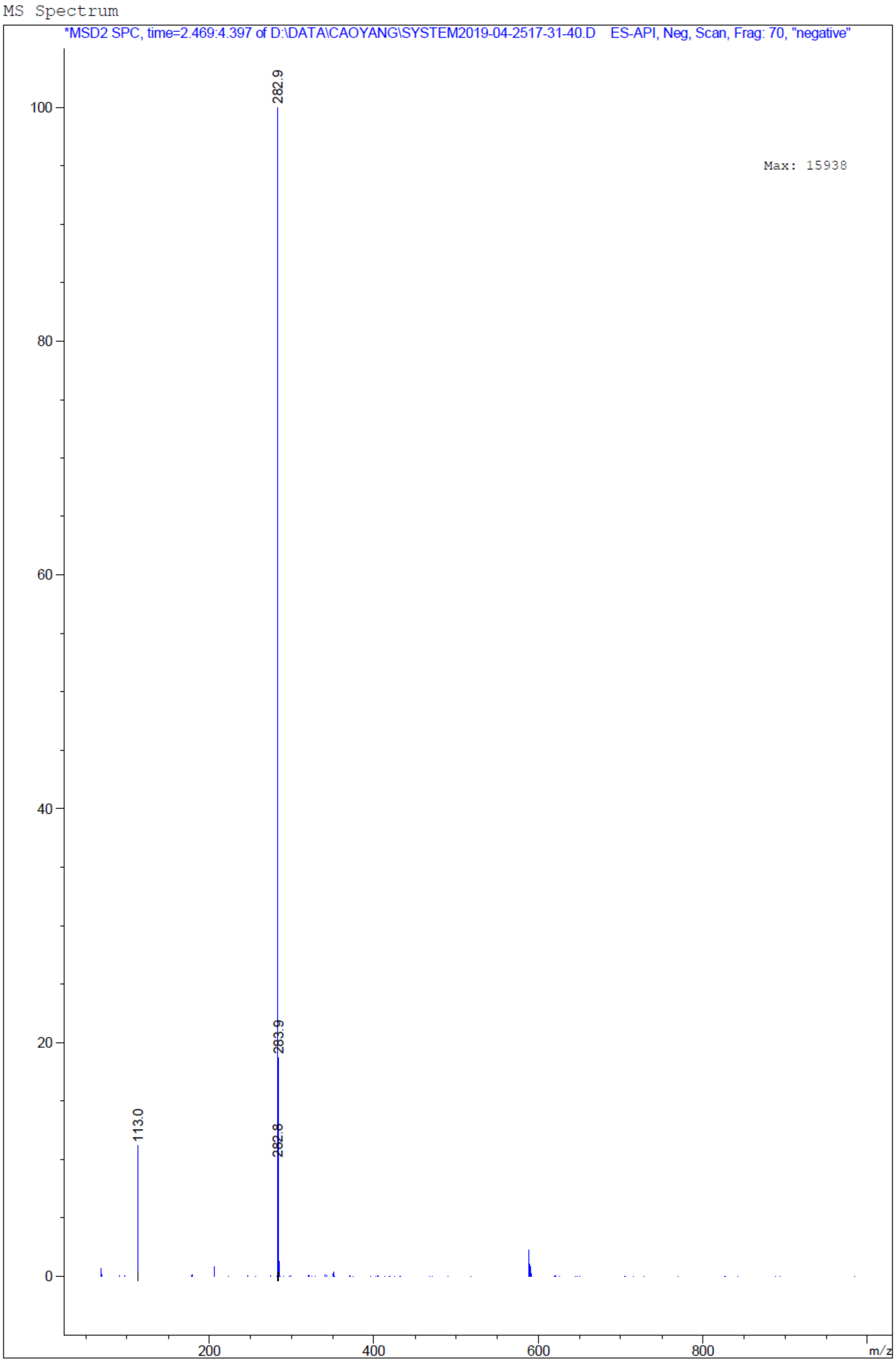

Figure S147. Mass spectrum (negative ionization) of **21w**

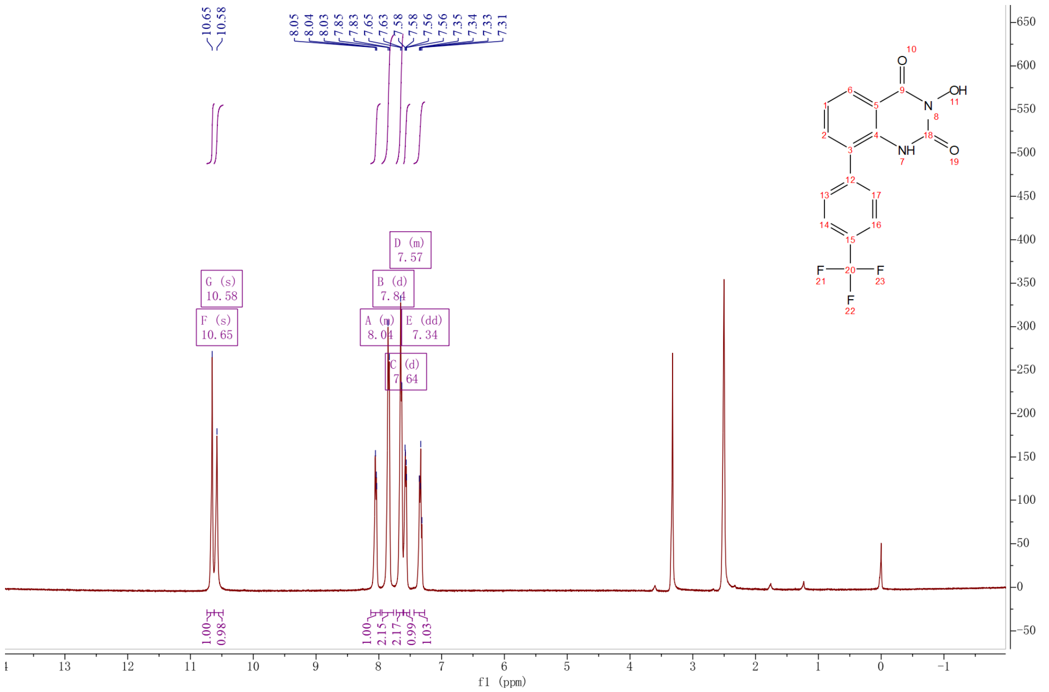

Figure S148. ^{1}H NMR (400 MHz, DMSO-d_6) spectrum of **21x**

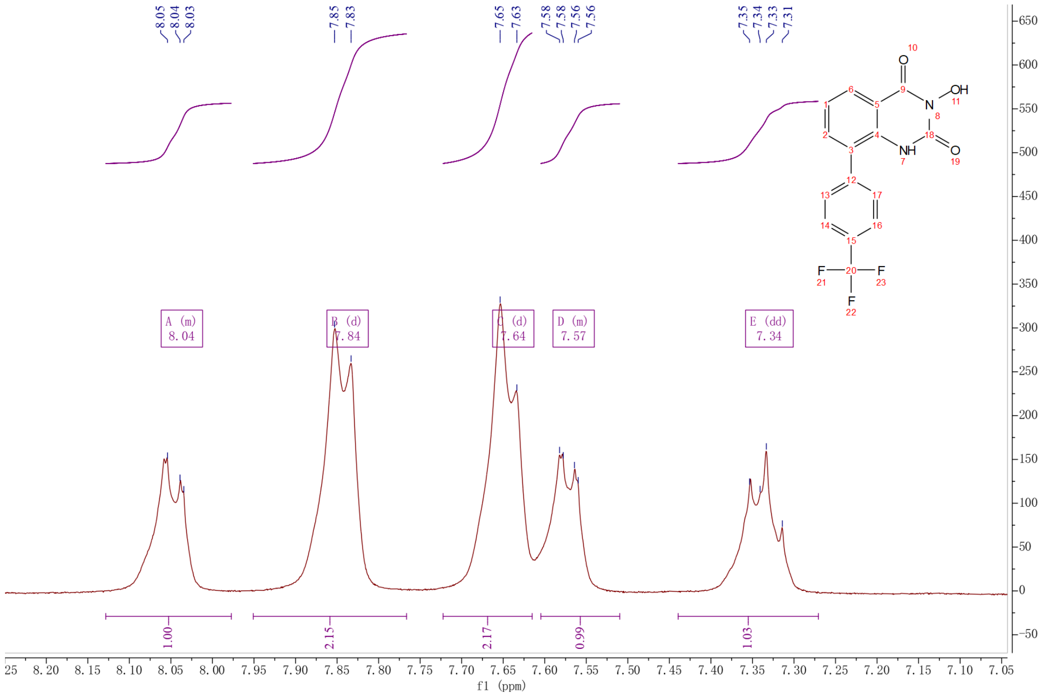

Figure S149. Magnified ^{1}H NMR (400 MHz, DMSO-d_6) spectrum fragments of **21x**

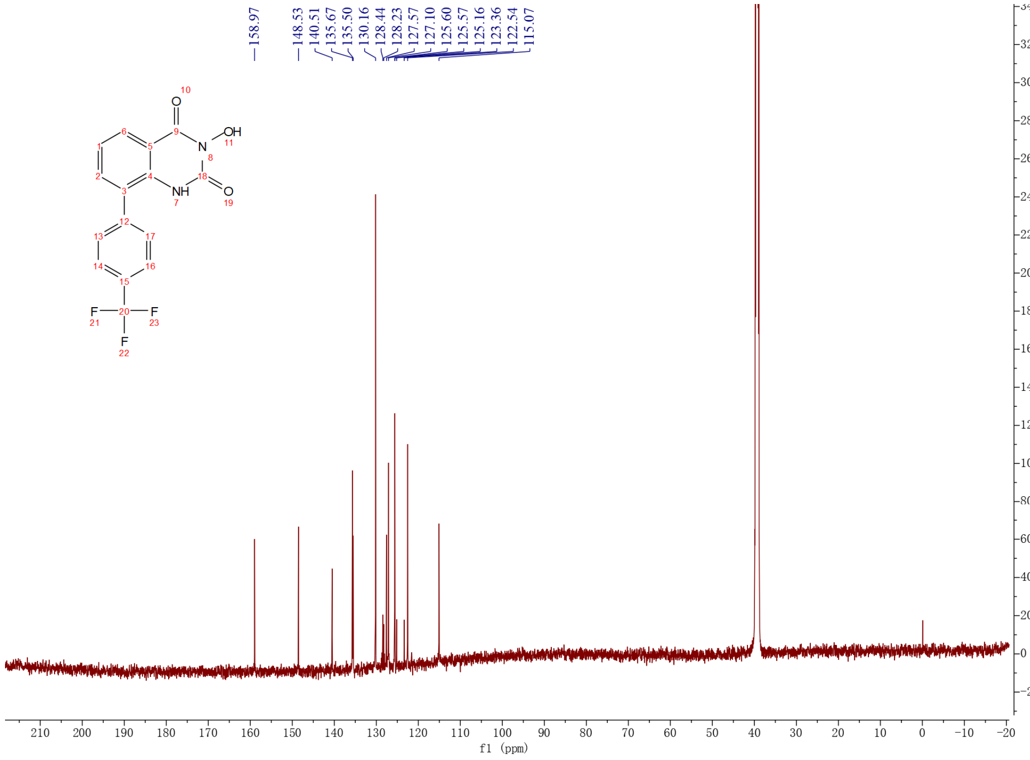

Figure S150. ^{13}C NMR (151 MHz, DMSO-d_6) spectrum of **21x**

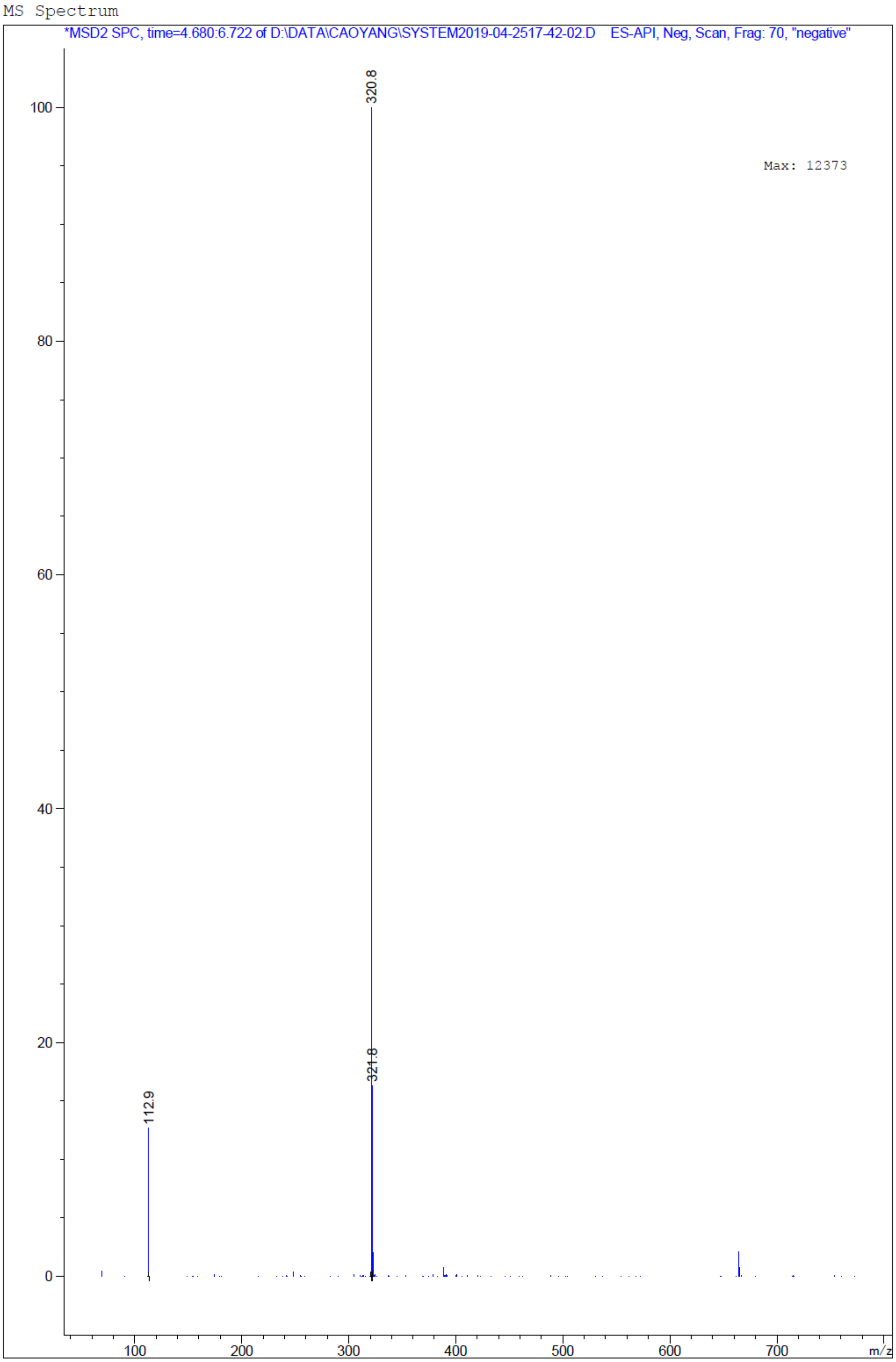

Figure S151. Mass spectrum (negative ionization) of **21x**

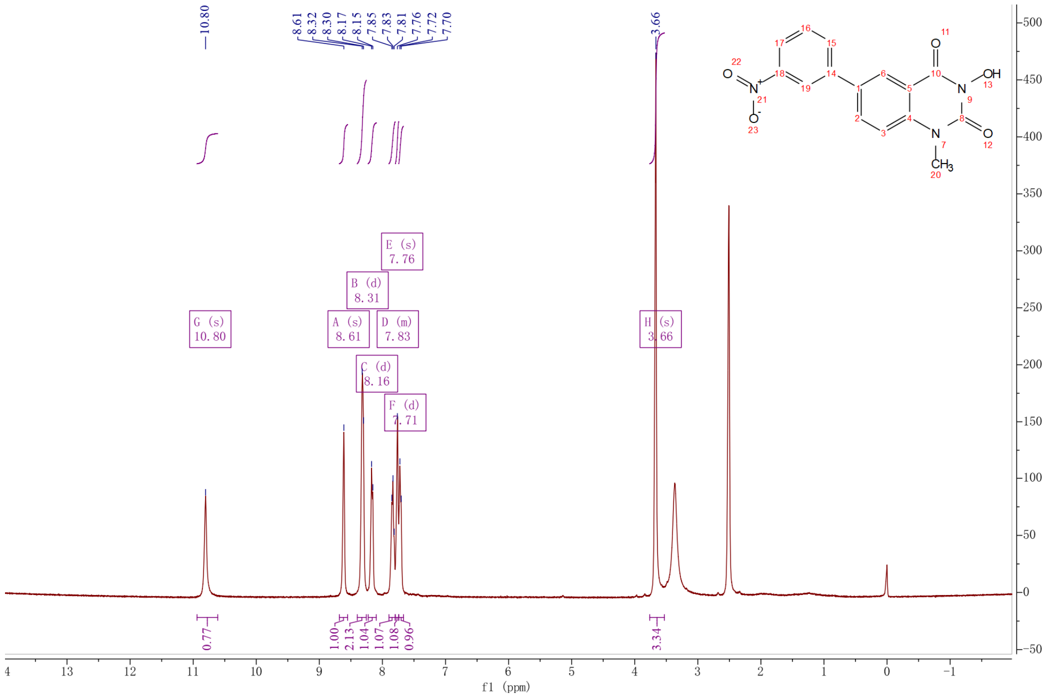

Figure S152. ^{1}H NMR (400 MHz, DMSO-d_6) spectrum of **23a**

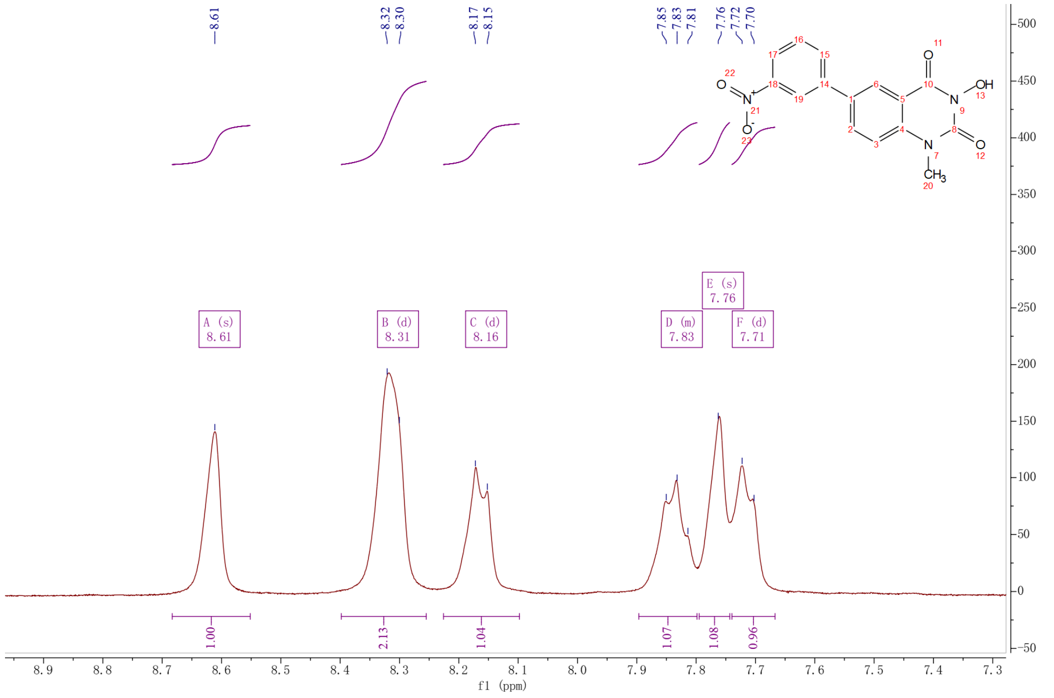

Figure S153. Magnified ^{1}H NMR (400 MHz, DMSO-d_6) spectrum fragments of **23a**

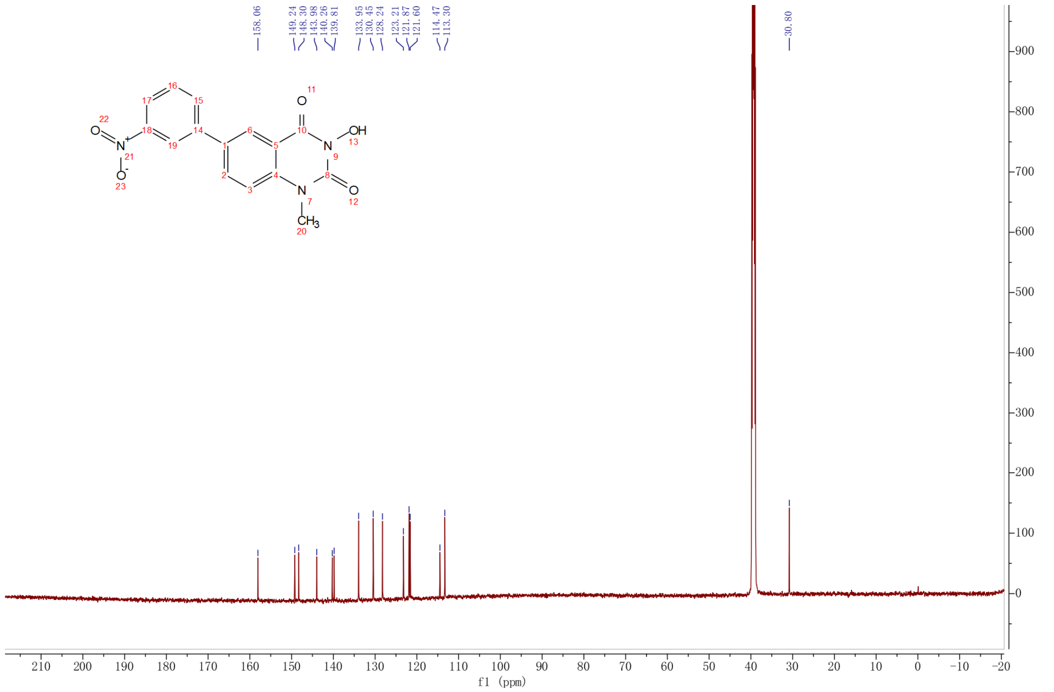

Figure S154. ^{13}C NMR (151 MHz, DMSO-d_6) spectrum of **23a**

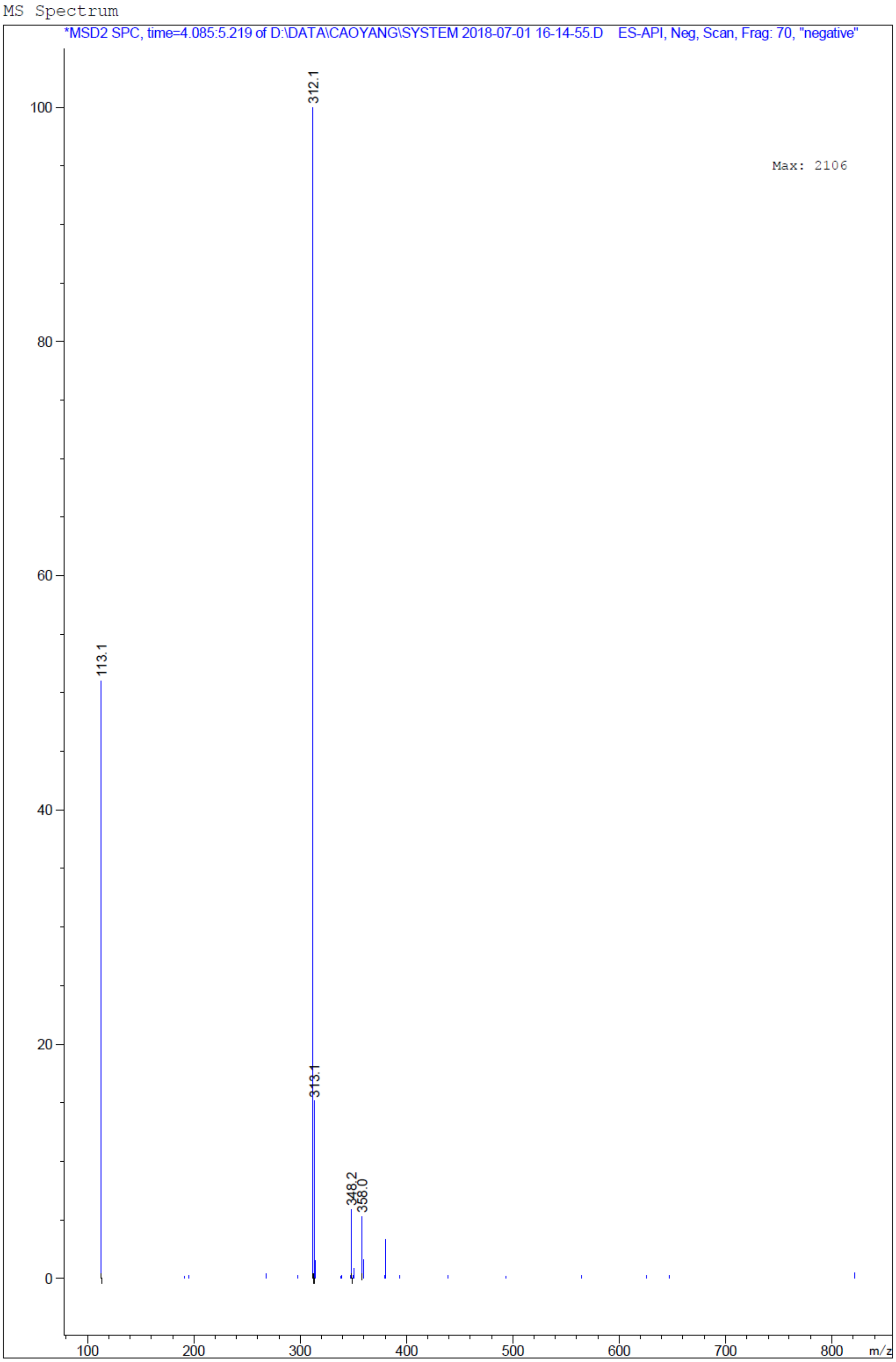

Figure S155. Mass spectrum (negative ionization) of **23a**

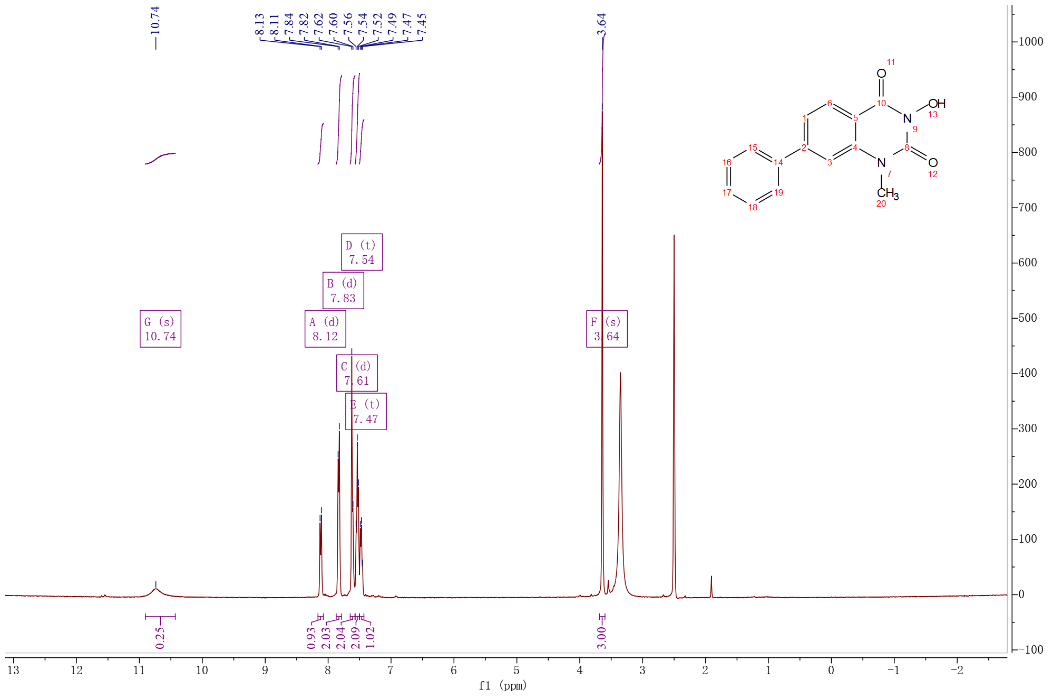

Figure S156. ^{1}H NMR (400 MHz, DMSO-d_6) spectrum of **23b**

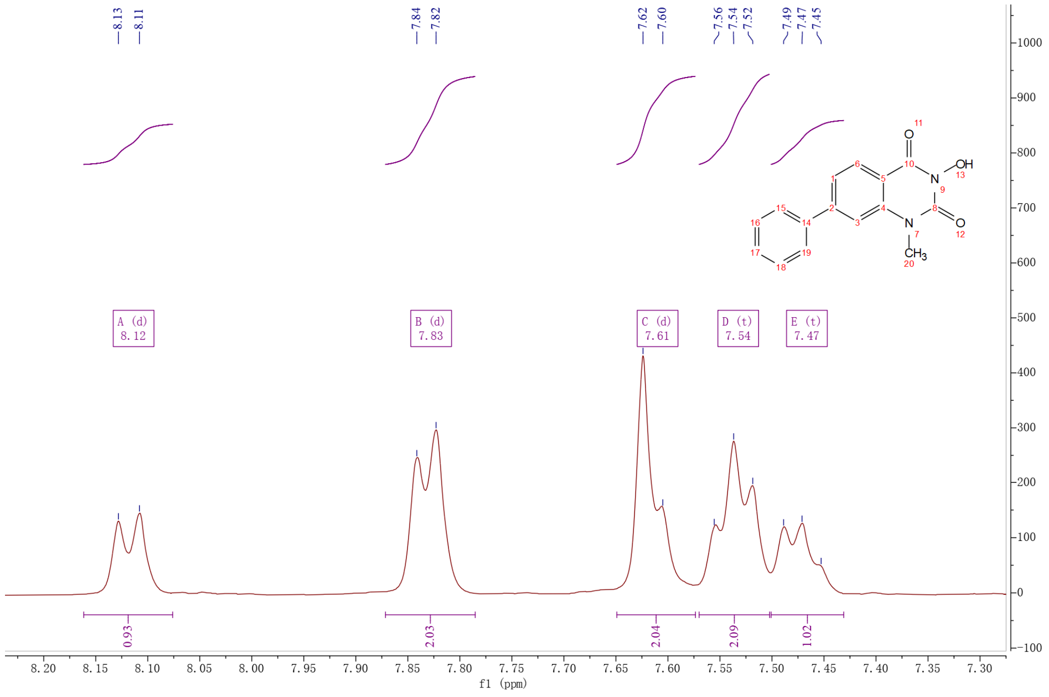

Figure S157. Magnified ^{1}H NMR (400 MHz, DMSO-d_6) spectrum fragments of **23b**

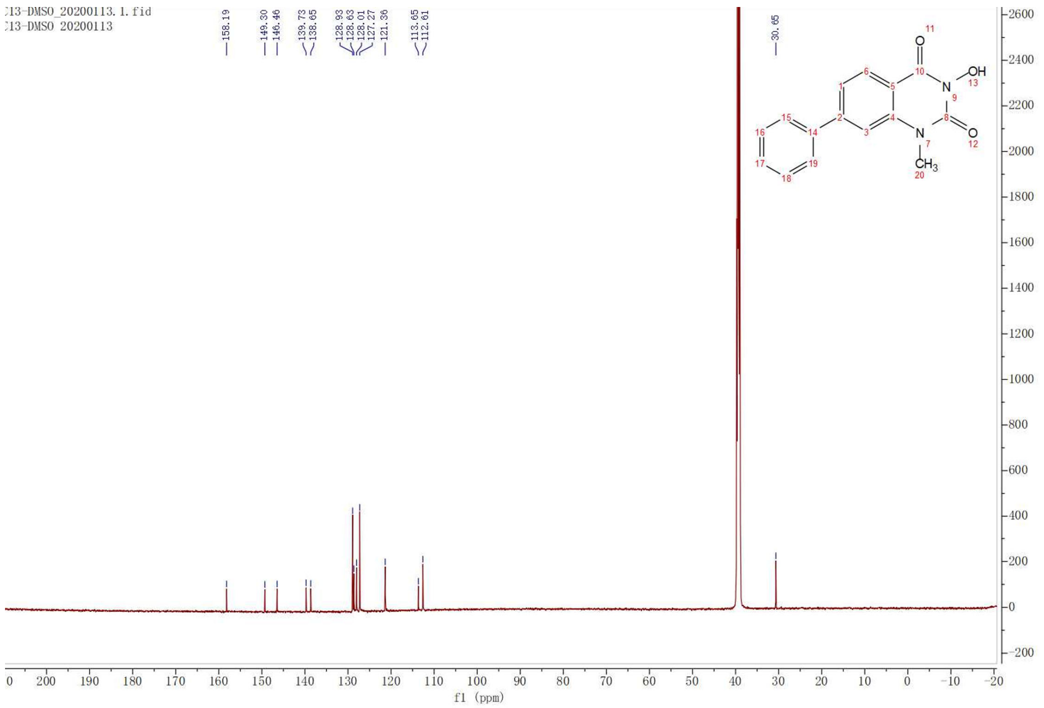

Figure S158. ^{13}C NMR (151 MHz, DMSO-d_6) spectrum of **23b**

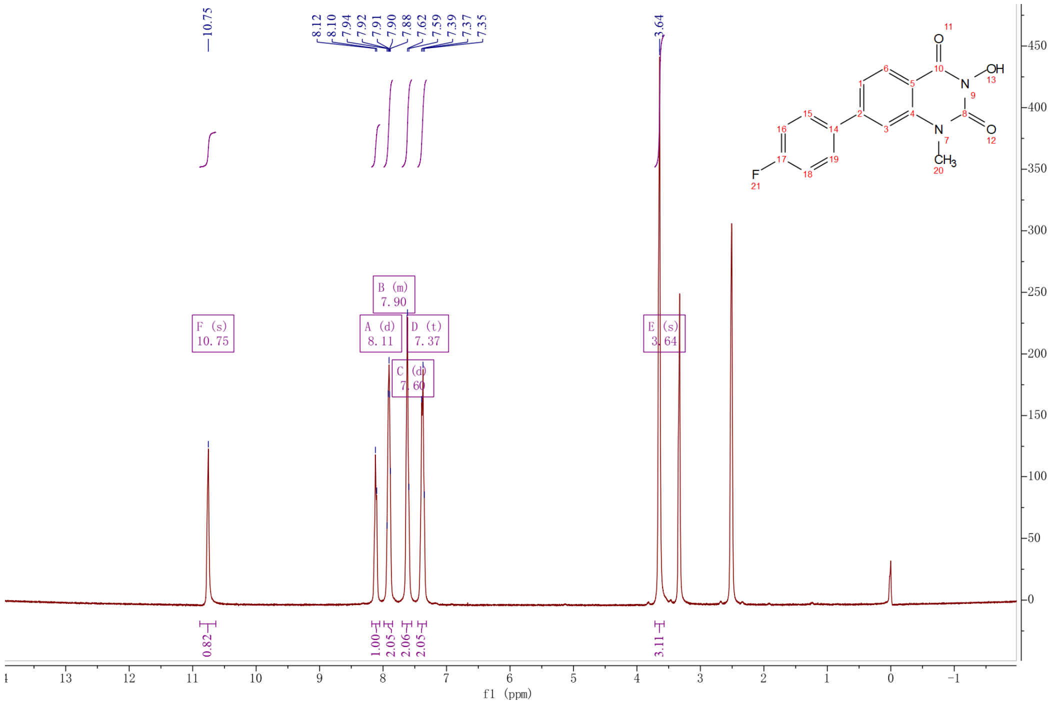

Figure S159. ^{1}H NMR (400 MHz, DMSO-d_6) spectrum of **23c**

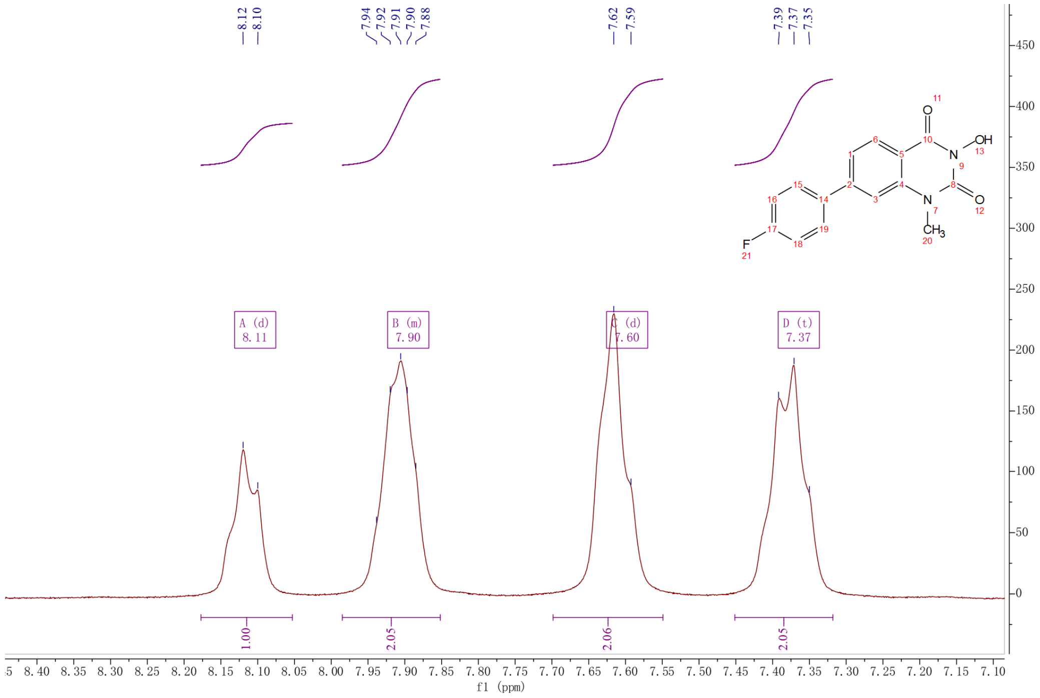

Figure S160. Magnified ^{1}H NMR (400 MHz, DMSO-d_6) spectrum fragments of **23c**

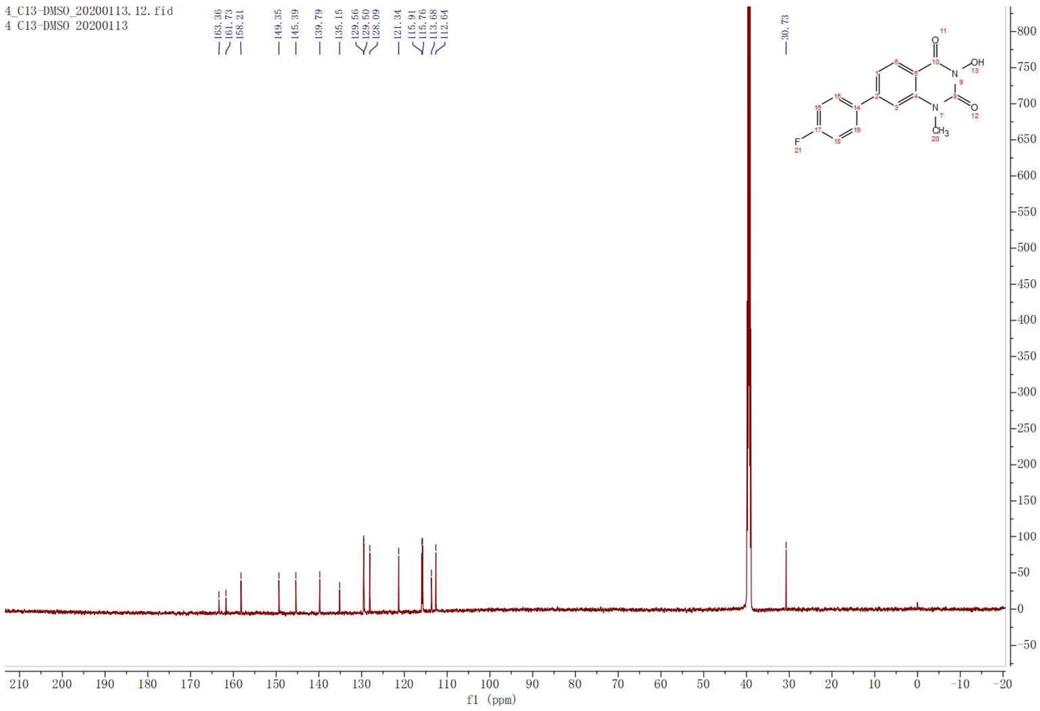

Figure S161. ^{13}C NMR (151 MHz, DMSO-d_6) spectrum of **23c**

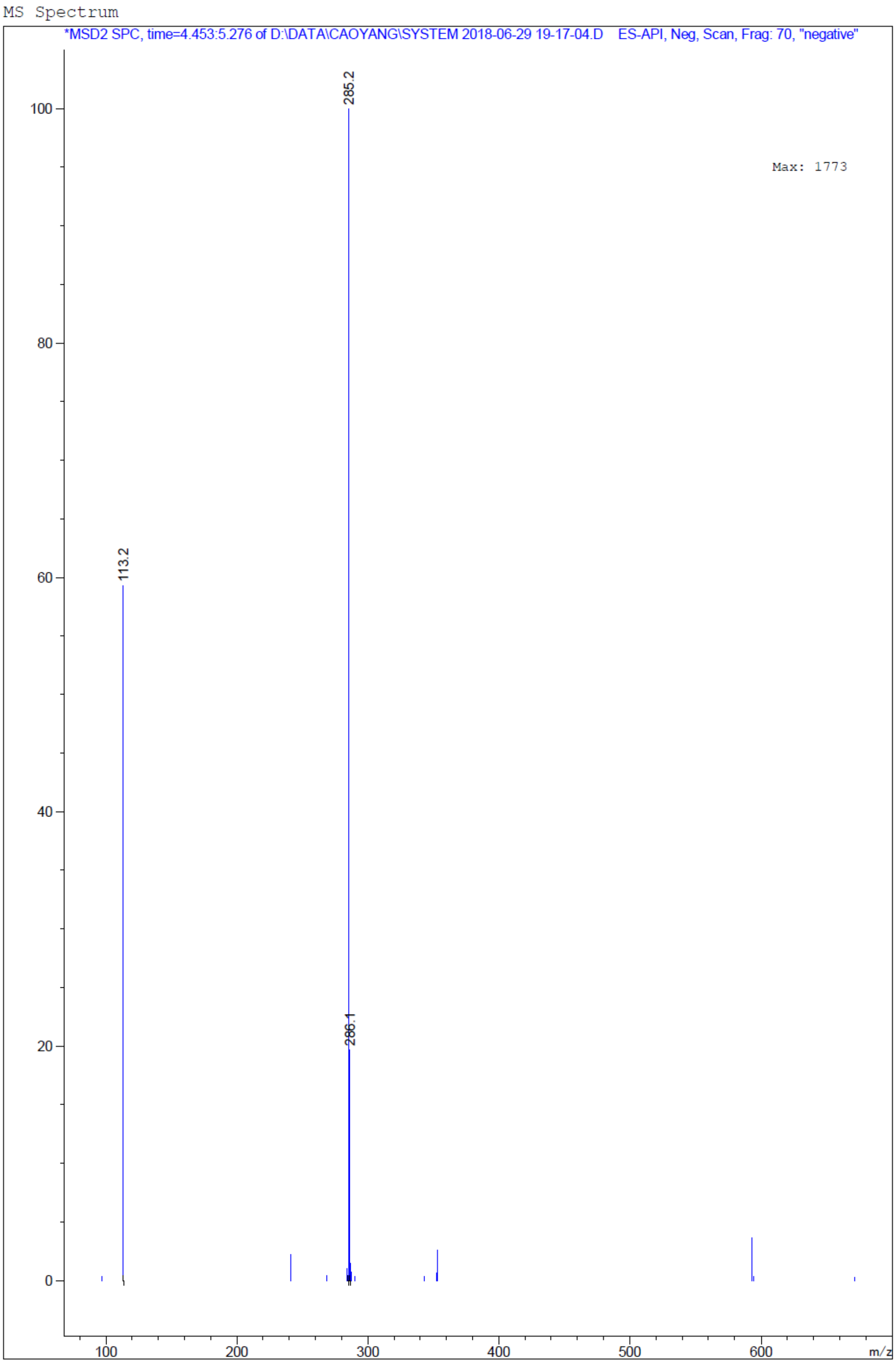

Figure S162. Mass spectrum (negative ionization) of **23c**

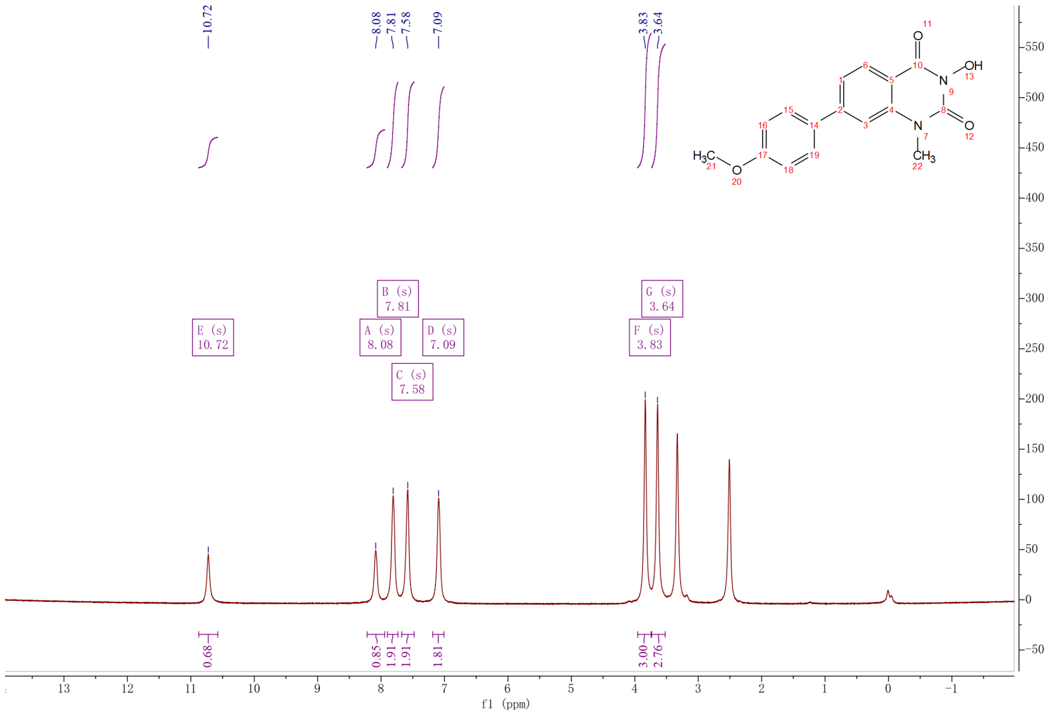

Figure S163. ^{1}H NMR (400 MHz, DMSO-d_6) spectrum of **23d**

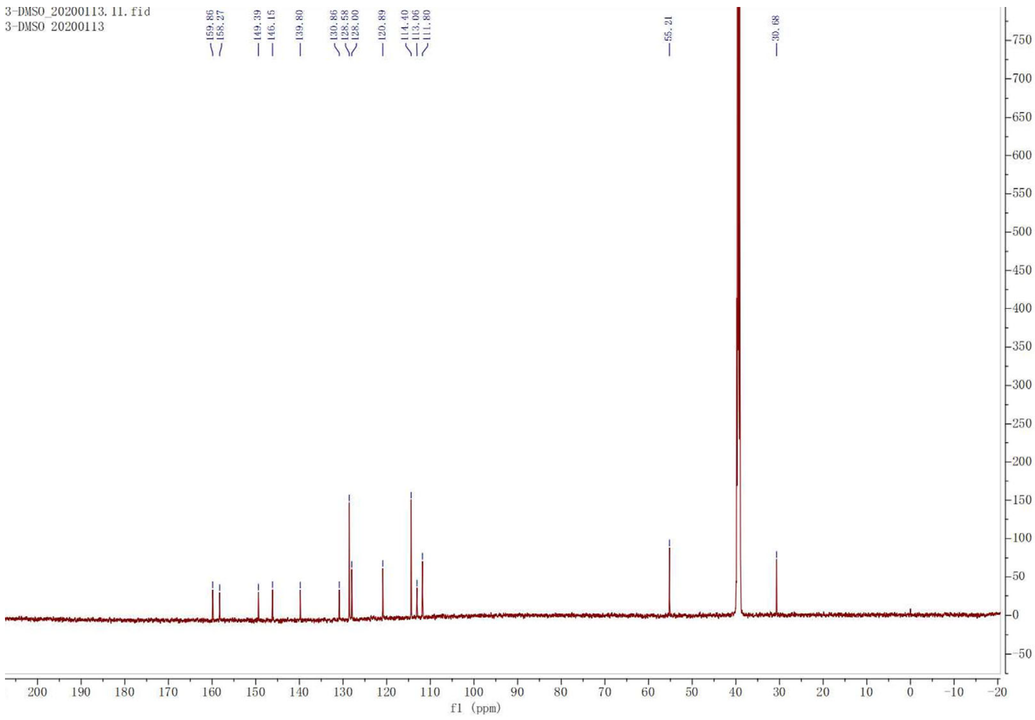

Figure S164. ^{13}C NMR (151 MHz, DMSO-d_6) spectrum of **23d**

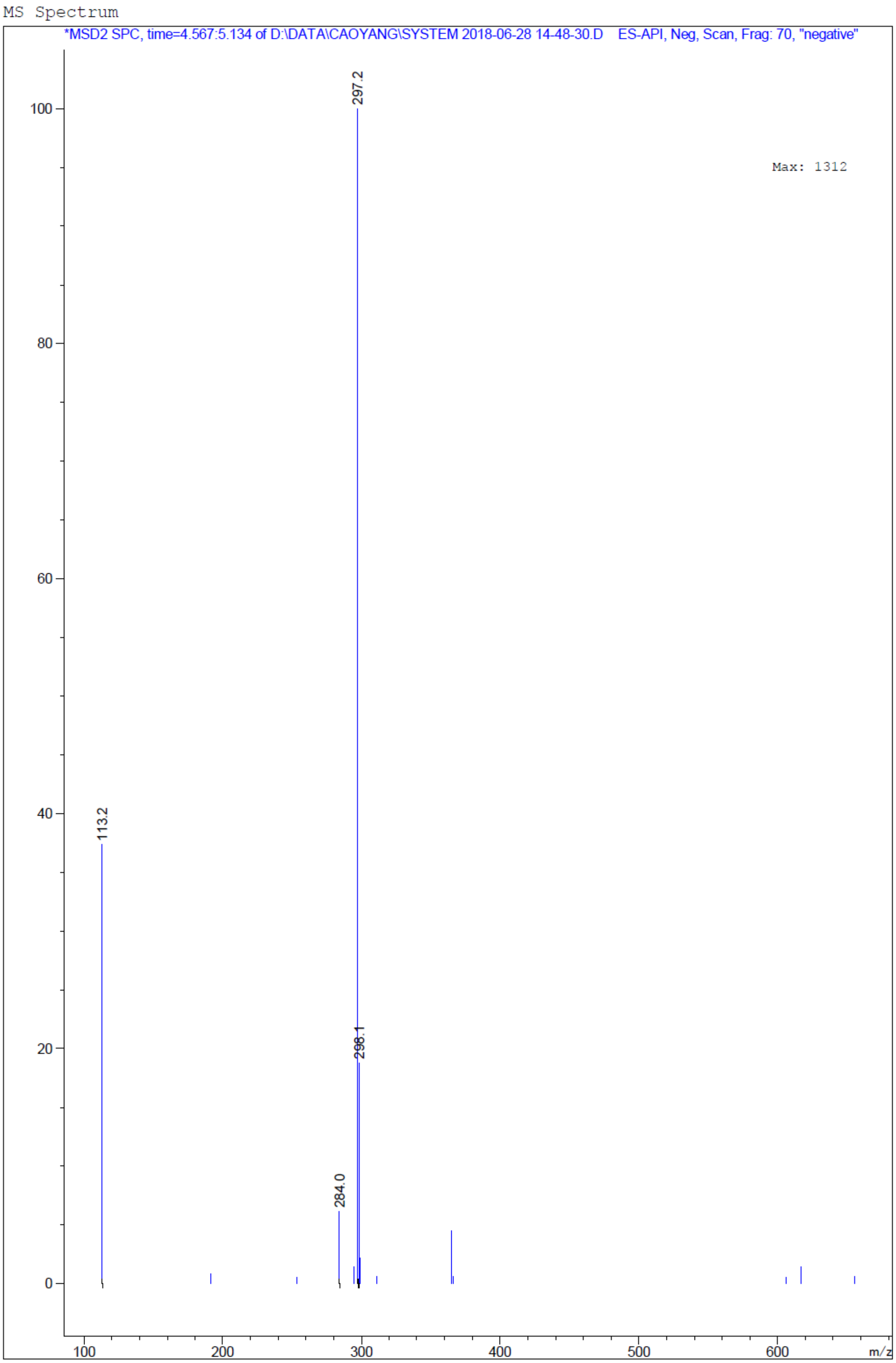

Figure S165. Mass spectrum (negative ionization) of **23d**

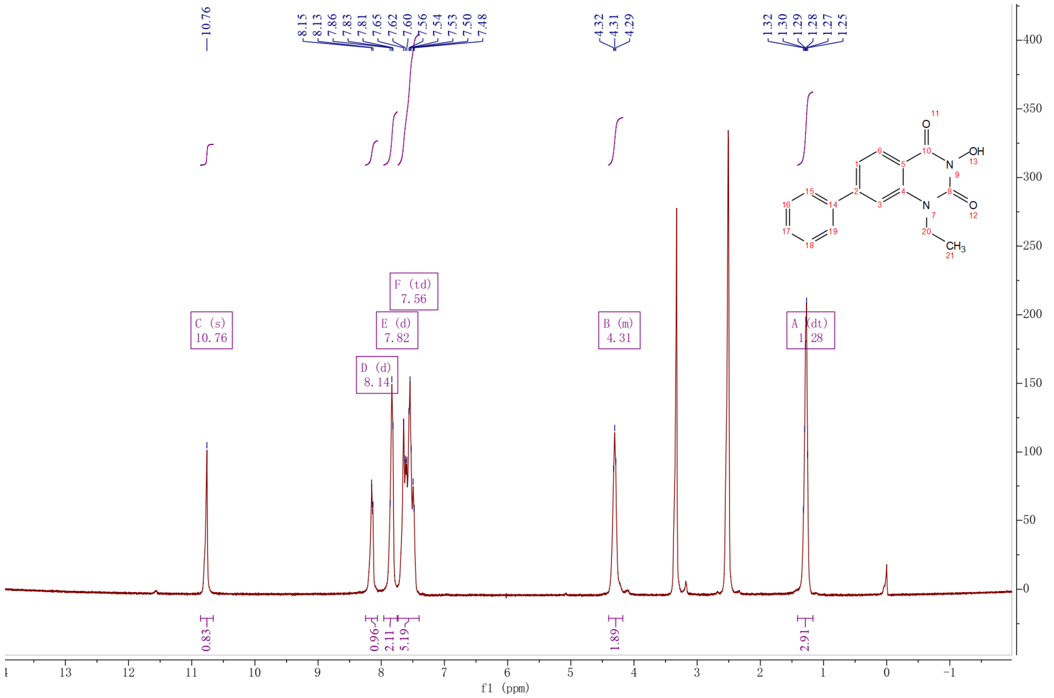

Figure S166. ^{1}H NMR (400 MHz, DMSO-d_6) spectrum of **23e**

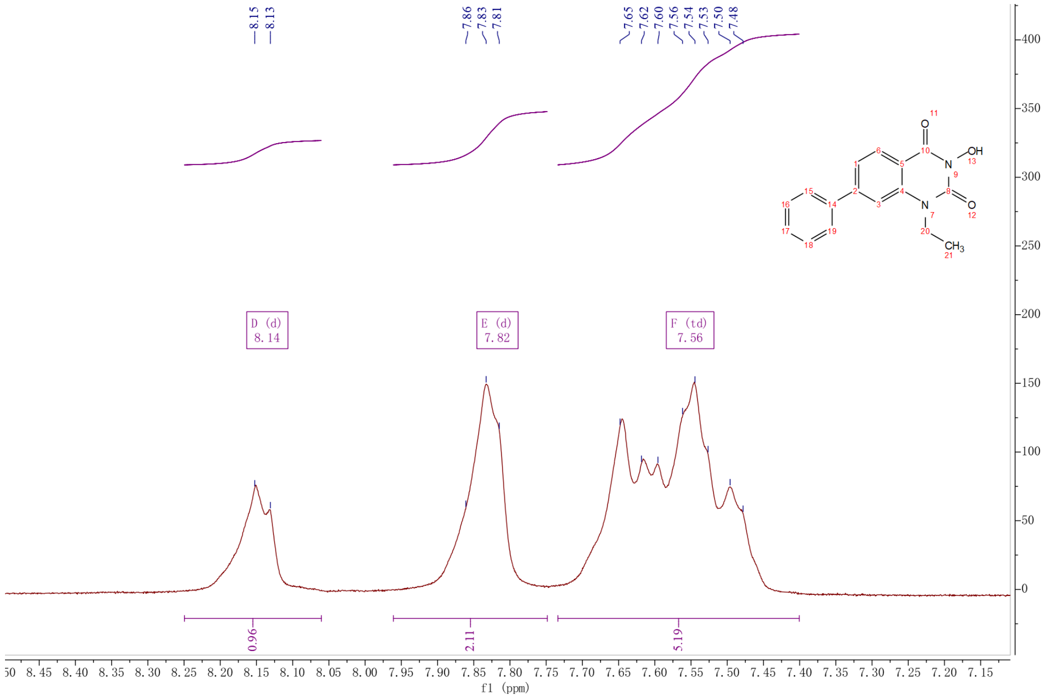

Figure S167. Magnified ^{1}H NMR (400 MHz, DMSO-d_6) spectrum fragments of **23e**

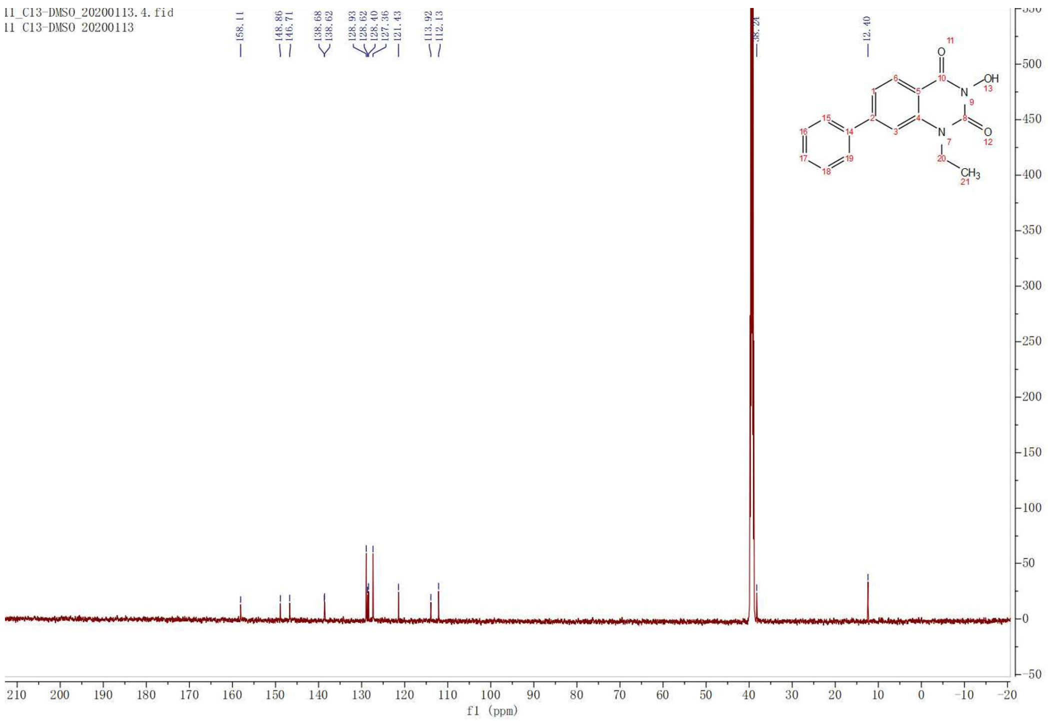

Figure S168. ^{13}C NMR (151 MHz, DMSO-d_6) spectrum of **23e**

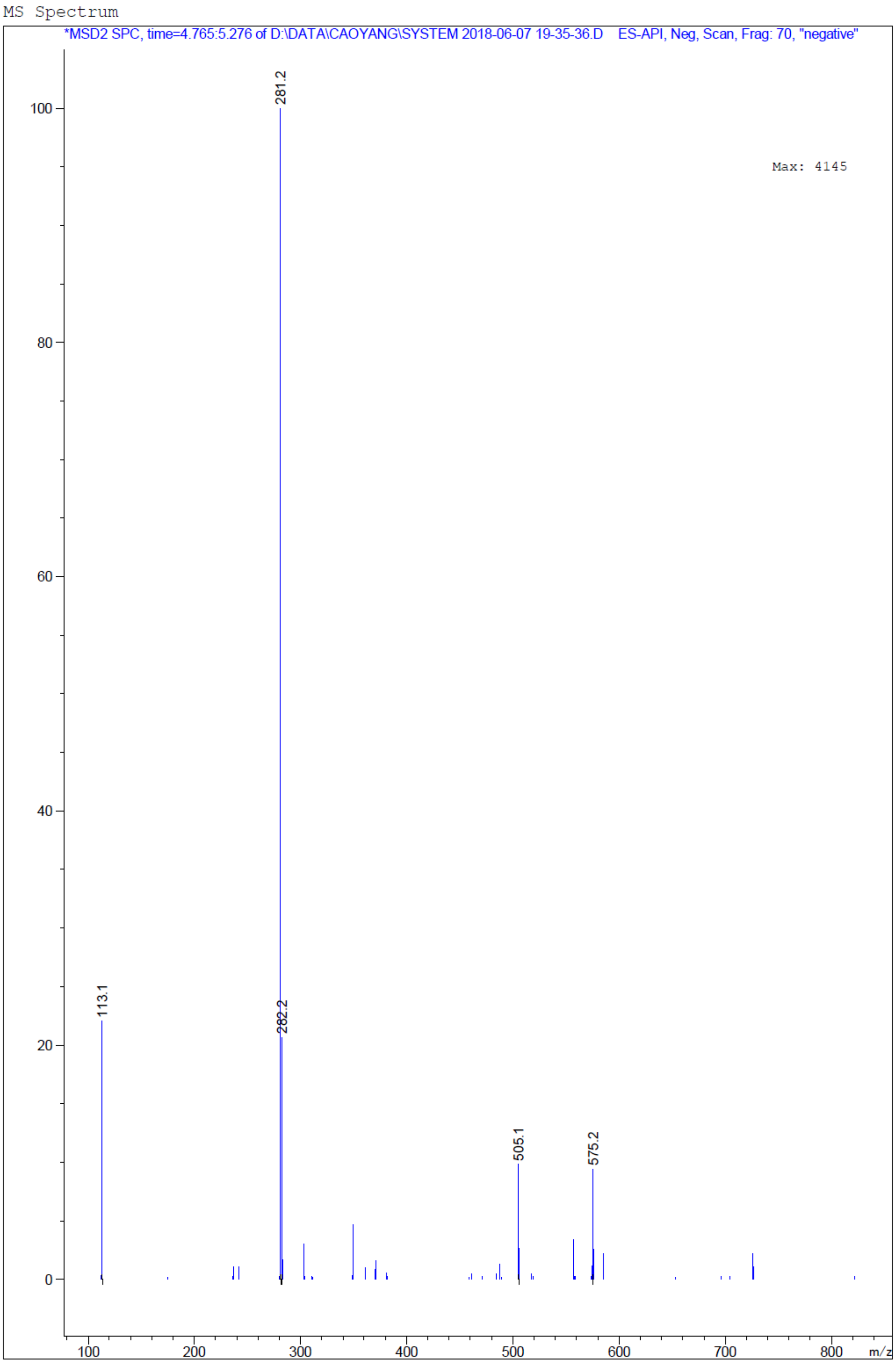

Figure S169. Mass spectrum (negative ionization) of **23e**

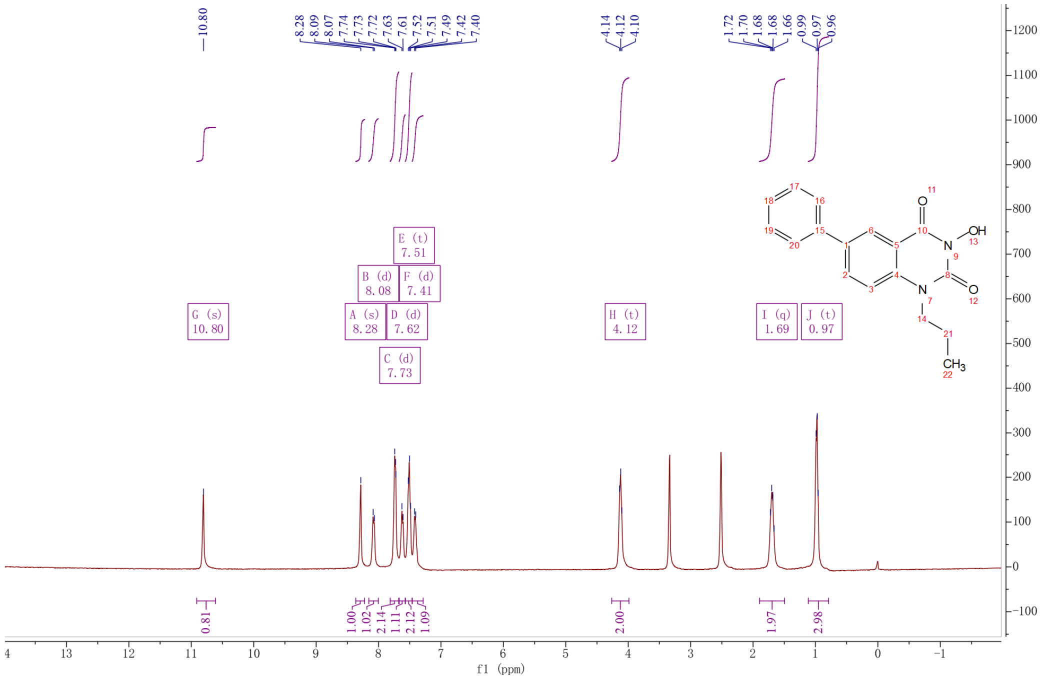

Figure S170. ^{1}H NMR (400 MHz, DMSO-d_6) spectrum of **23f**

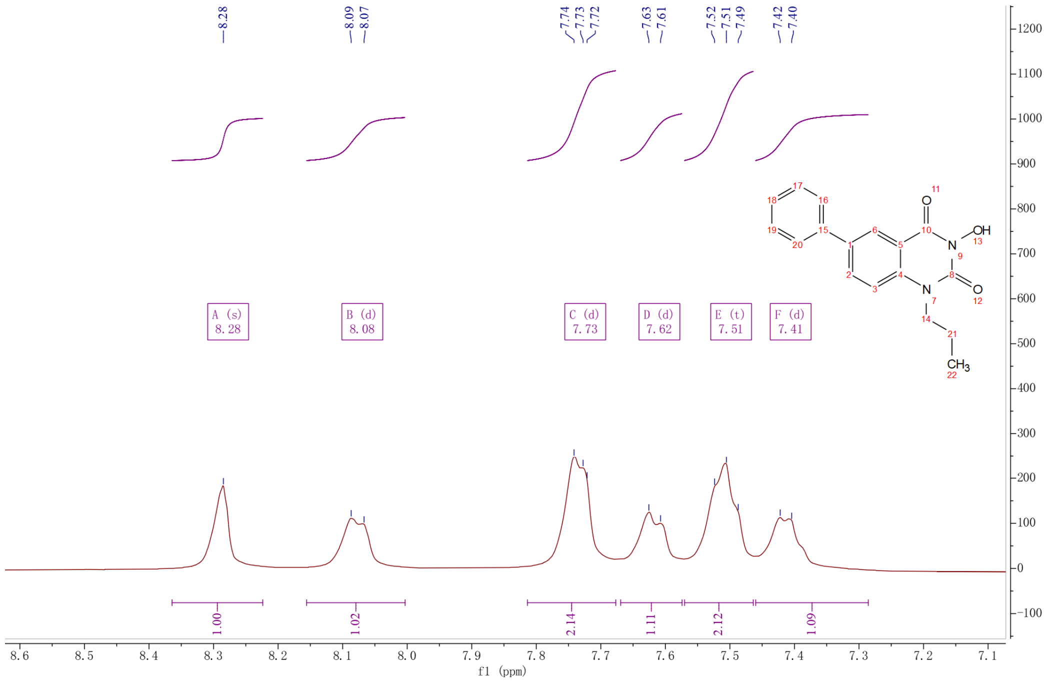

Figure S171. Magnified ^{1}H NMR (400 MHz, DMSO-d_6) spectrum fragments of **23f**

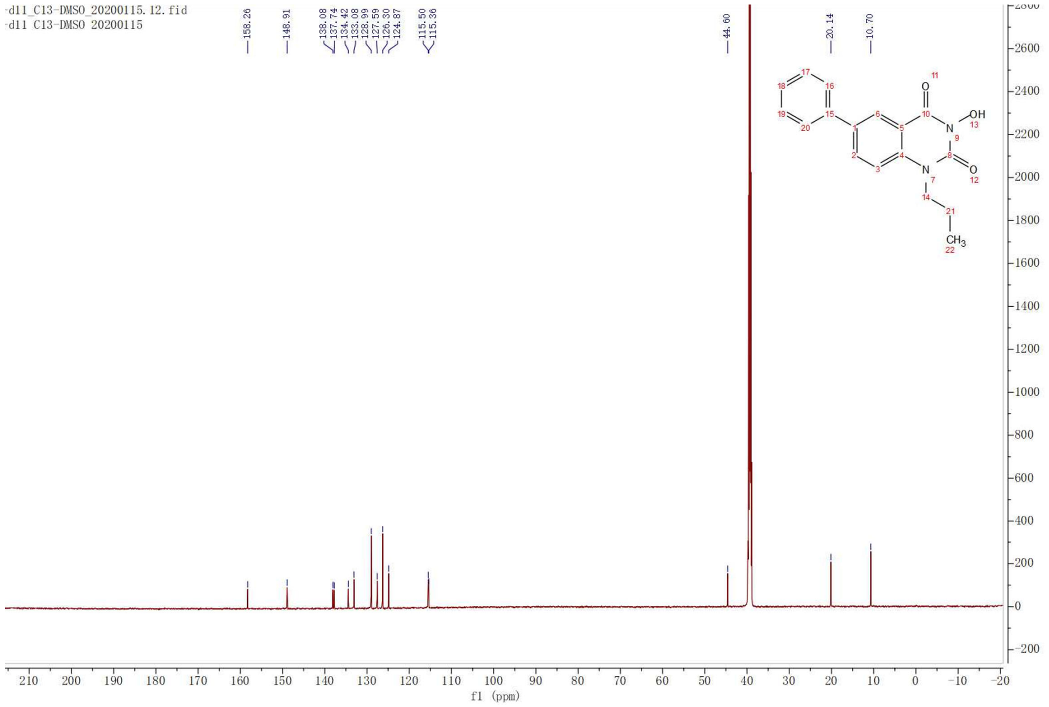

Figure S172. ^{13}C NMR (151 MHz, DMSO-d_6) spectrum of **23f**

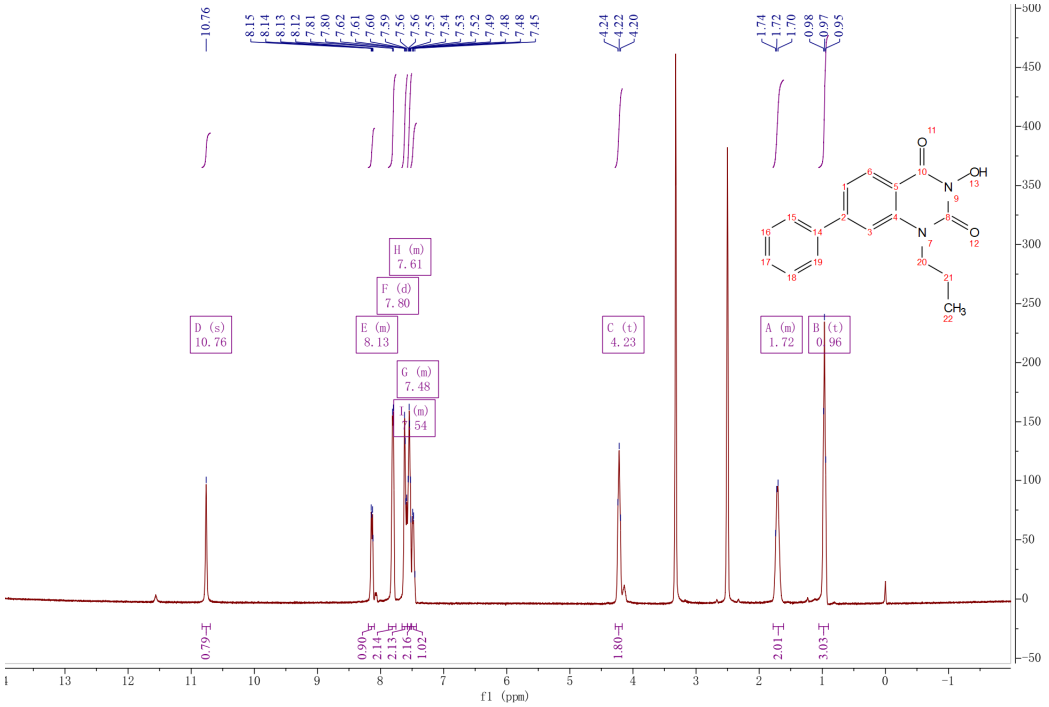

Figure S173. ^{1}H NMR (400 MHz, DMSO-d_6) spectrum of **23g**

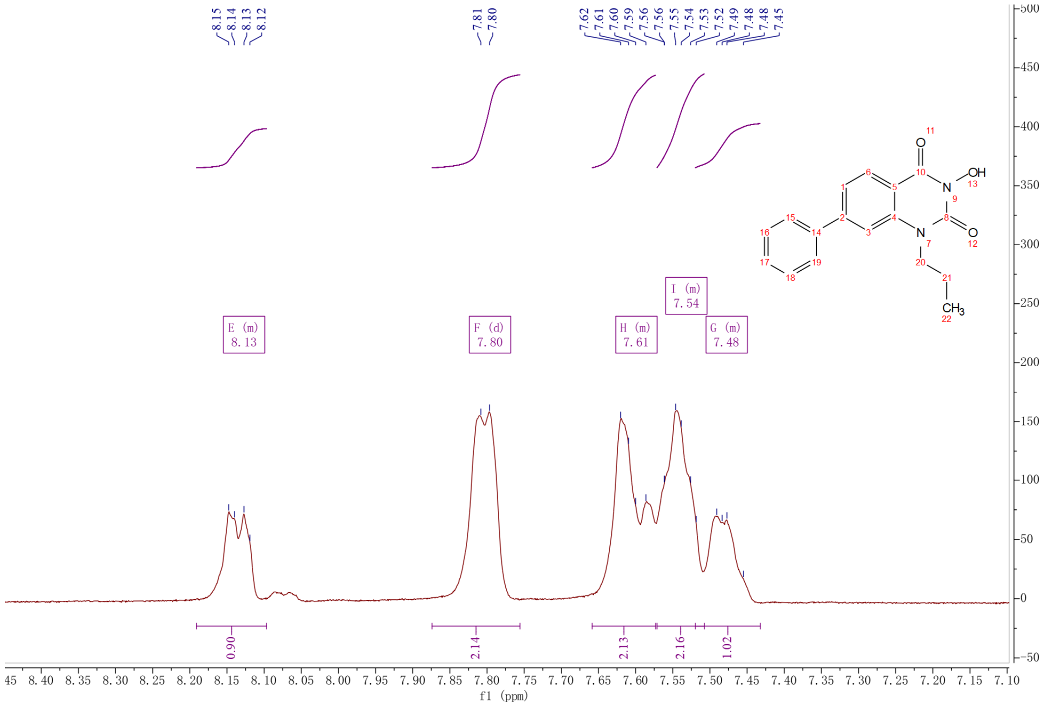

Figure S174. Magnified ^{1}H NMR (400 MHz, DMSO-d_6) spectrum fragments of **23g**

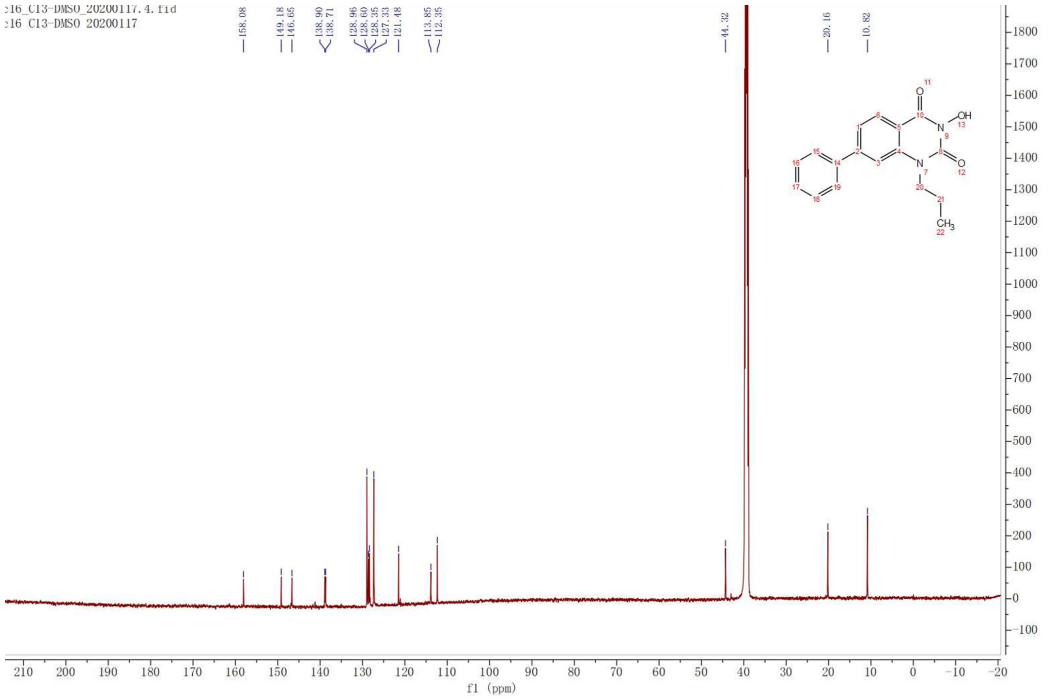

Figure S175. ^{13}C NMR (151 MHz, DMSO-d_6) spectrum of **23g**

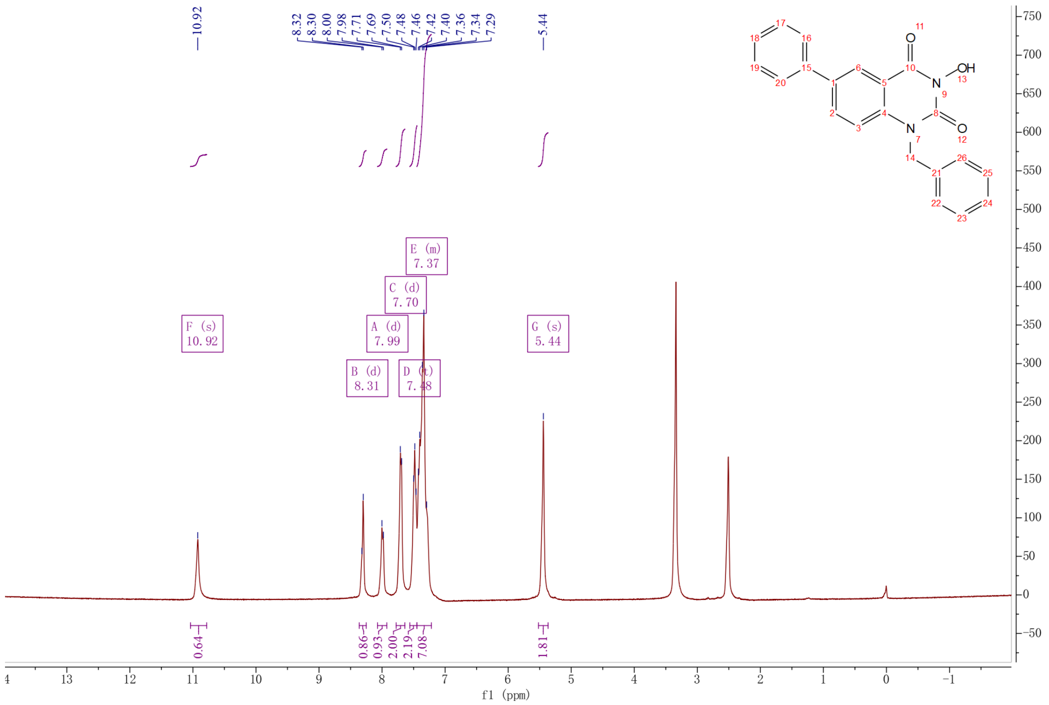

Figure S176. ^{1}H NMR (400 MHz, DMSO-d_6) spectrum of **23h**

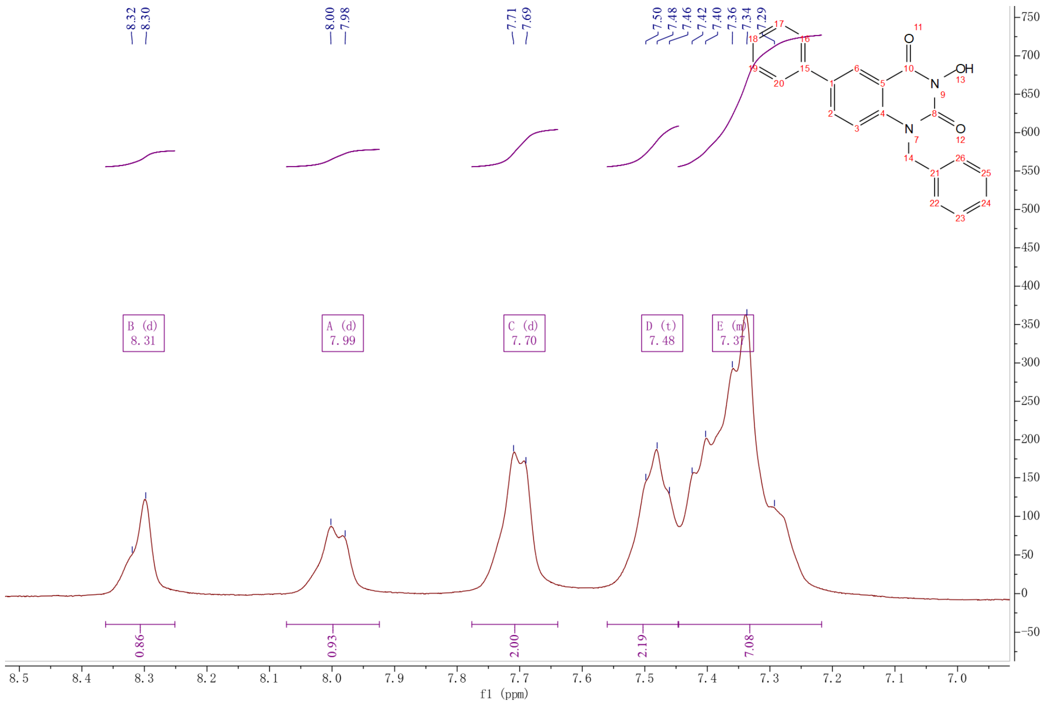

Figure S177. Magnified ^{1}H NMR (400 MHz, DMSO-d_6) spectrum fragments of **23h**

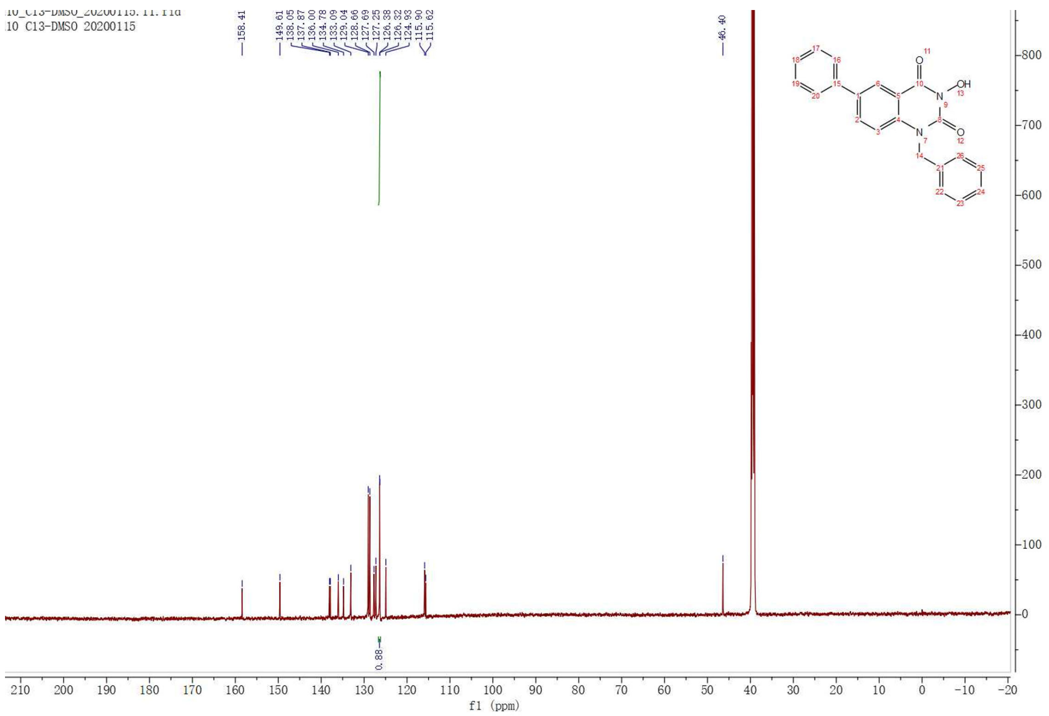

Figure S178. ^{13}C NMR (151 MHz, DMSO-d_6) spectrum of **23h**

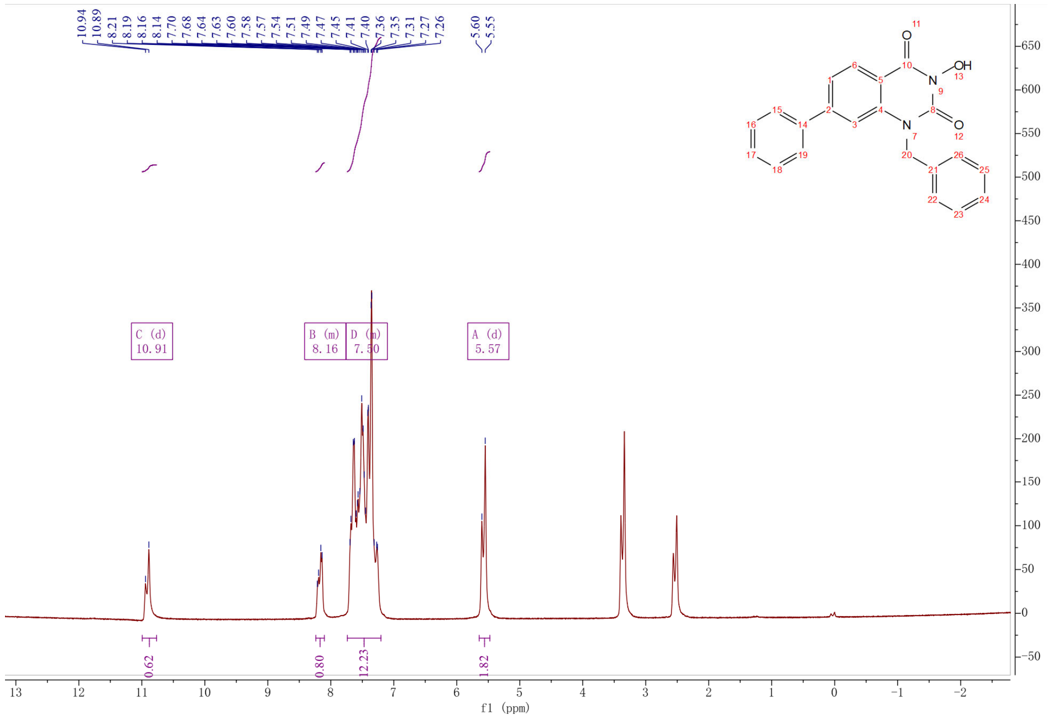

Figure S179. ^{1}H NMR (400 MHz, DMSO-d_6) spectrum of **23i**

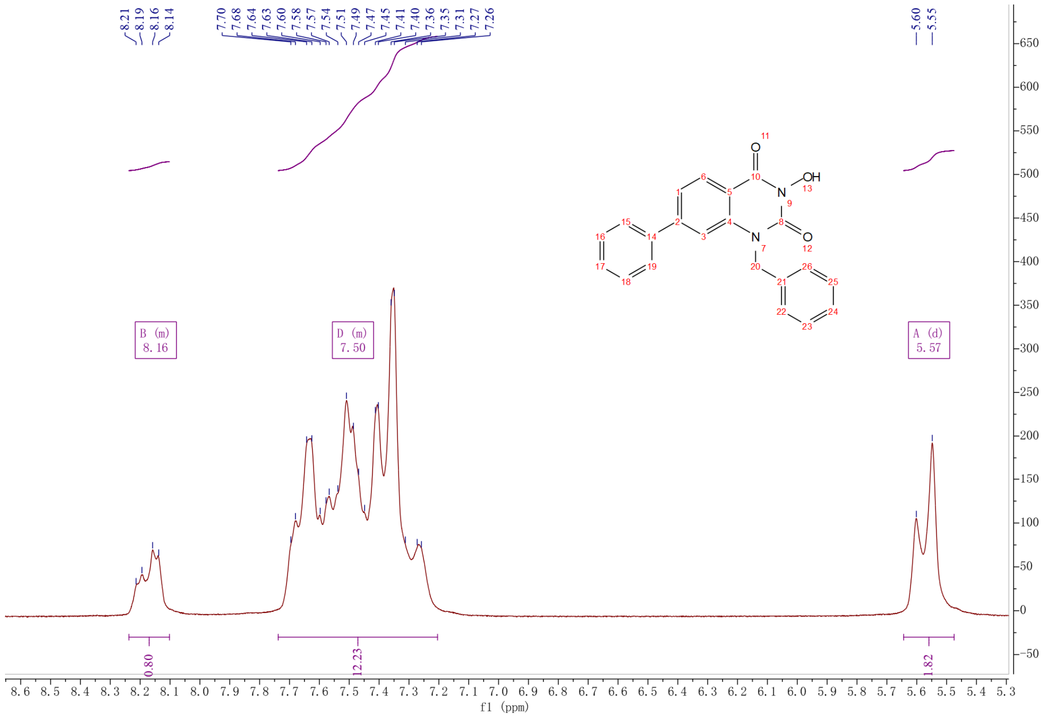

Figure S180. Magnified ^{1}H NMR (400 MHz, DMSO-d_6) spectrum fragments of **23i**

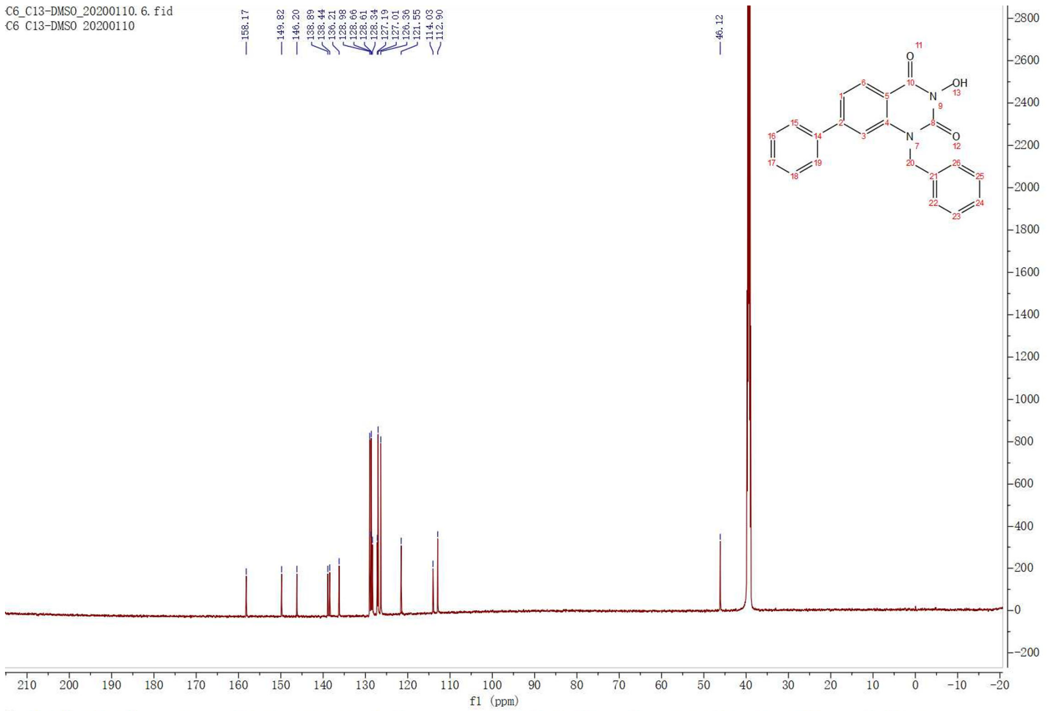

Figure S181. ^{13}C NMR (151 MHz, DMSO-d_6) spectrum of **23i**

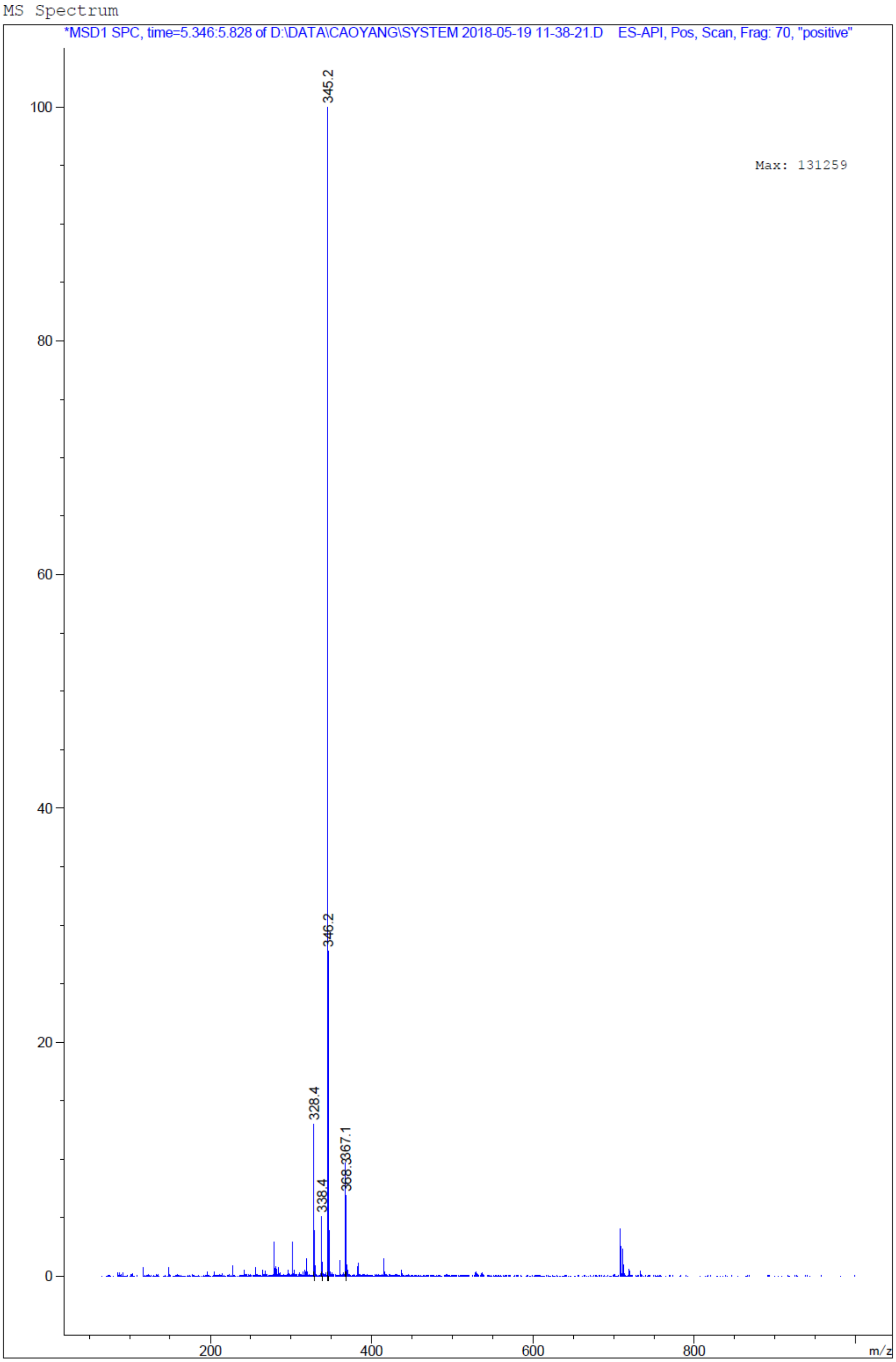

Figure S182. Mass spectrum (positive ionization) of **23i**

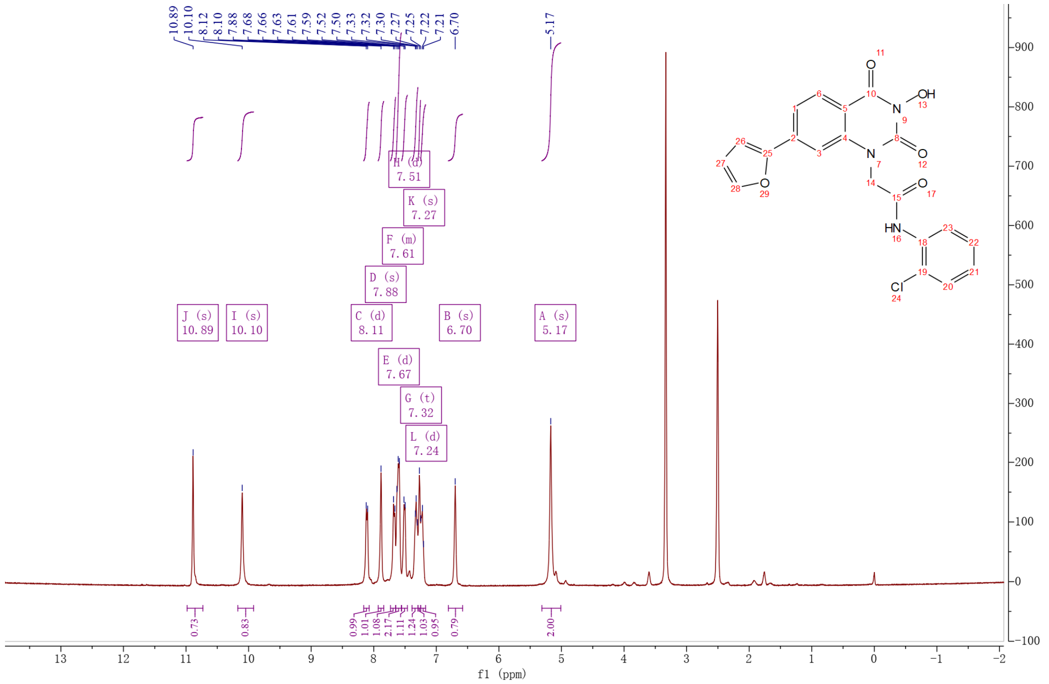

Figure S183. ^{1}H NMR (400 MHz, DMSO-d_6) spectrum of **23j**

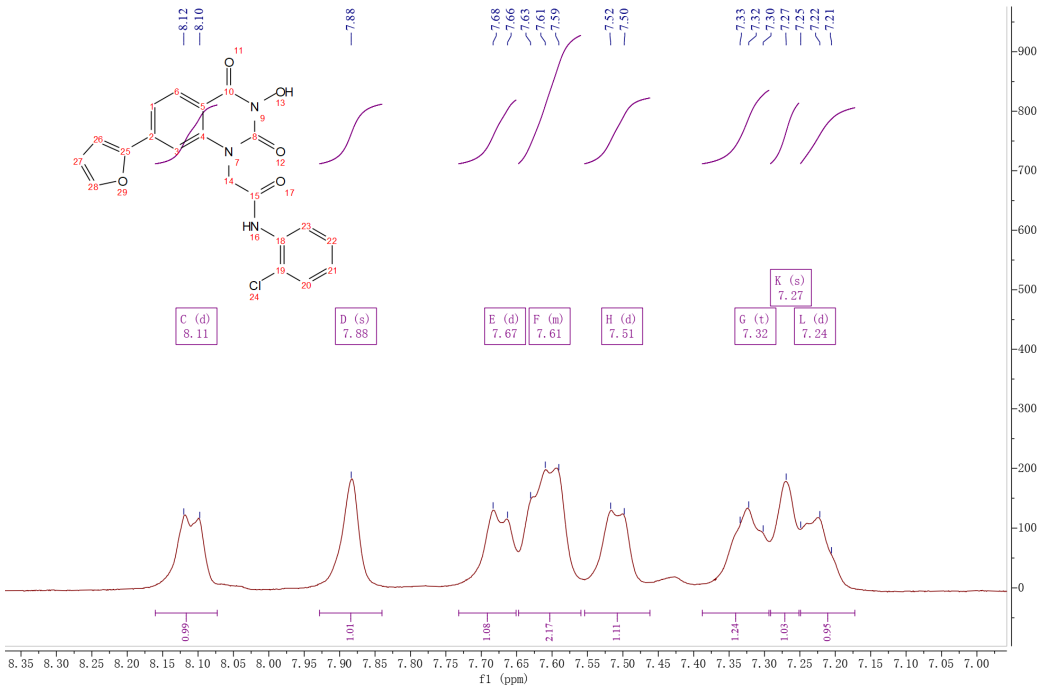

Figure S184. Magnified ^{1}H NMR (400 MHz, DMSO-d_6) spectrum fragments of **23j**

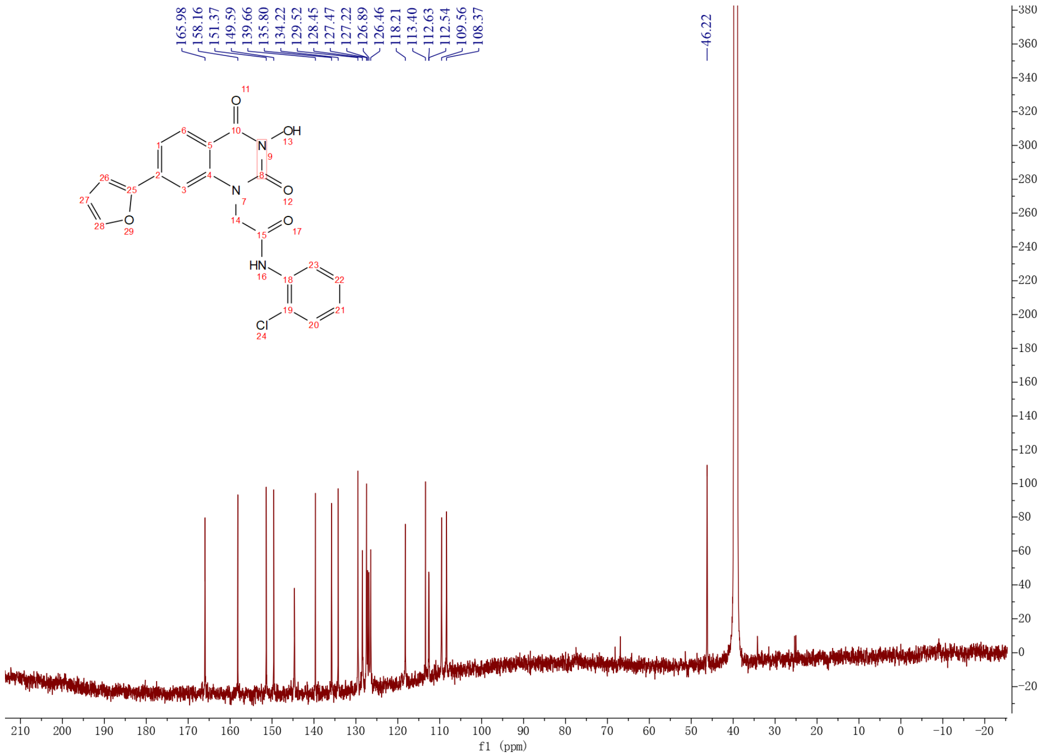

Figure S185. ^{13}C NMR (151 MHz, DMSO-d_6) spectrum of **23j**
